# Electric-Field Catalysis of Diels–Alder Reactions

**DOI:** 10.1021/acscentsci.6c00852

**Published:** 2026-07-24

**Authors:** Bing-Ru Shao, Shen-Yi Guo, Miguel Paraja, Qing-Xia Zhang, Augustina Jozeliu̅naitė, Naomi Sakai, Stefan Matile

**Affiliations:** † Department of Organic Chemistry, 27212 University of Geneva, 1211 Geneva, Switzerland; ‡ National Centre of Competence in Research (NCCR) Molecular Systems Engineering, 4002 Basel, Switzerland

## Abstract

The dream to catalyze reactions with externally applied electric fields is so obvious and so promising that it has attracted immense scientific attention. The Diels–Alder (DA) reaction has become the primary reaction to elaborate on this dream, but electric-field catalysis (EFC) of DA reactions under practical organic synthesis conditions remained impossible. The objective of this study was to challenge the emerging principles of scalable EFC in microfluidic capacitors with this holy grail in the field. Functional Helmholtz layers are assembled to translate applied electric fields into effective electric fields that are, according to chronoamperometry, strong enough for the EFC. Yields increase nonlinearly from 0% up to 90% with applied voltage. In chiral cationic Helmholtz layers assembled from polyarginines, benzylated quinines, or ammonium binaphthyls, enantioselectivity up to 31% *ee* is obtained. These results demonstrate that the emerging principles of EFC in microfluidic capacitors pass the ultimate test in the field with distinction: Besides realizing DA-EFC with externally applied electric fields under practical conditions, this study also introduces chiral Helmholtz layers as (i) the primary catalysts, with an impressive efficiency of EC_50_ = 0.2 mol %, and (ii) the unique source of chirality for asymmetric EFC.

## Graphical abstract

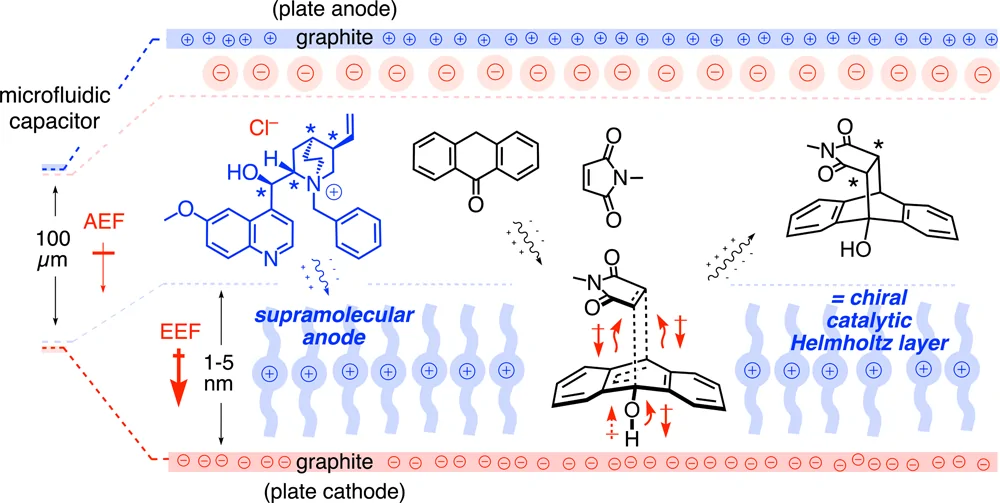

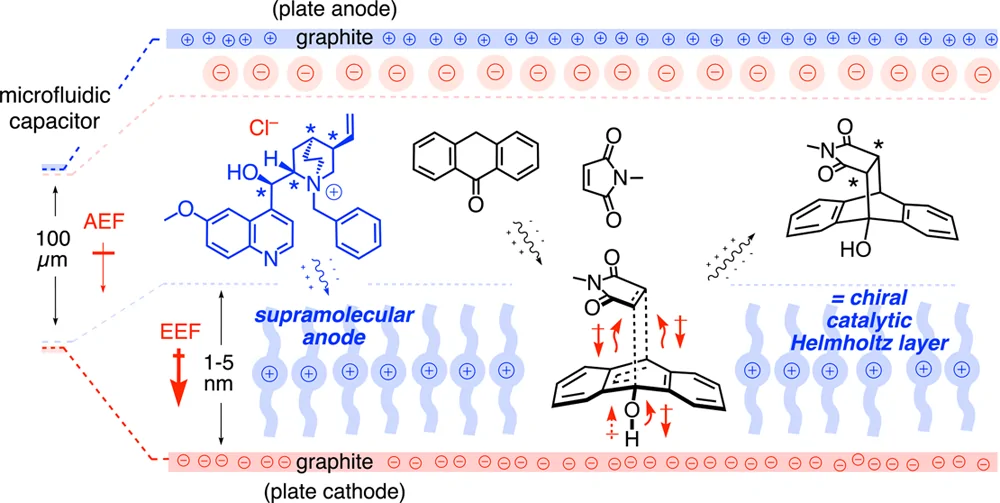

## Introduction

Every chemical reaction involves the movement of electrons from one place to another. The dream to enable, accelerate, and direct these charge displacements with externally applied electric fields (AEFs) has inspired chemists for decades.^1–4^ It is supported by studies in elegant model systems,^5–28^ by the local electric fields that enzymes have evolved to accomplish their performance,^29–34^ and by numerous theoretical investigations.^1–4,35–41^ The elephant in the room, however, is its incompatibility with practical organic synthesis conditions.

For electric-field catalysis (EFC), the Diels–Alder reaction has emerged as the primary reaction.^1–4,14–16,19–22,36–41^ In normal electron demand Diels–Alder (NEDDA) reactions, the charge transfer *Q*_CT_ occurs from the HOMO of the electron-rich diene to the LUMO of the electron-poor dienophile (Figure 1A). In their pioneering computational analysis in 2010, Shaik and co-workers showed already that effective electric fields (EEFs) applied along this *z*-axis either accelerate (+*z*, + EEF_*z*_) or inhibit the NEDDA (−*z*), depending on the direction.^36^ For inverse electron demand Diels–Alder (IEDDA) reactions, inverse *Q*_CT_ from the HOMO of the electron-rich dienophile to the LUMO of the electron-poor diene is catalyzed and inhibited by EEFs of the opposite direction. Only recently, Ramanan and co-workers computed that strong EEFs in decelerating −*z* orientation can cause EFC because the reaction mechanisms change from NEDDA to IEDDA.^37^ This concept has been further expanded to Lewis acid catalyzed DA reactions.^38^ Polarization of the substrates in the *z* direction by +*z* EEFs, visualized best in molecular electrostatic potential surfaces, induces macrodipoles μ_*z*_ that support the reaction,^39^ and can proceed to charge-transfer complexes between the substrates as reactive intermediates. The original computational study by Shaik and co-workers also predicted that EEFs applied along the *y* axis should influence *endo*/*exo* selectivity, and that EEFs could change from concerted to stepwise mechanisms with zwitterionic intermediates.^36^ Subsequent studies further predicted that EEFs in the *x* axis should influence the enantioselectivity of DA reactions.^40,41^

**Figure 1:**
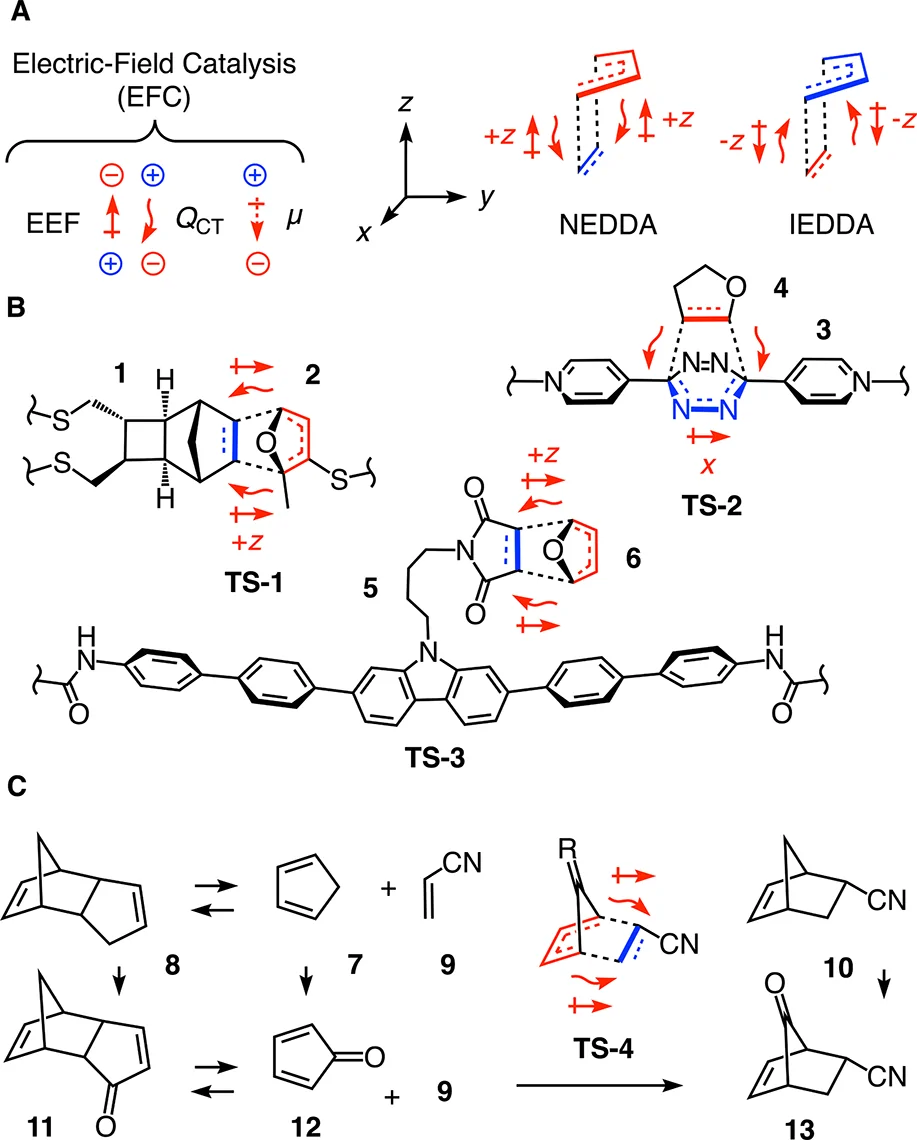
(A) The Diels–Alder reaction as primary reaction in theoretical studies of EFC. Effective electric fields (EEFs) oriented along the *z* axis of the charge transfer *Q*_CT_ during normal (NEDDA) and inverse electron demand Diels–Alder (IEDDA) cycloadditions catalyze or inhibit depending on their direction (+,−); μ defines the notation used also for macrodipoles. (B, C) Previous experimental studies of DA-EFC. Single-molecule break junction experiments with favorably (**TS-1**), unfavorably (**TS-2**), and flexibly oriented transition states (**TS-3**), and in situ MS detection experiments that disfavor operational EFC in water and MeCN microdroplets (**TS-4**).

Experimental support for these computational predictions first emerged 10 years ago from single-molecule studies with scanning tunneling microscopy break junctions (STM-BJs, Figure 1B).^14^ Norbornyl-derived dienophile **1** was covalently attached to a flat gold surface, and diene **2** was attached to a golden STM tip. In this construct, voltage applied between the gold tip and plate produces an AEF that is oriented in the *z* axis of the NEDDA reaction with transition state **TS-1**. The occurrence of a reaction can be detected from the tunneling current across the molecular bridge formed. In STM break junctions, single-molecule current reporting NEDDA reactions is blinking because after the reaction, the S–Au bonds of junctions break rapidly. NEDDA reactions were thus characterized as the blinking frequency. This blinking frequency increased linearly with +*z*-oriented AEFs up to saturation around 500 mV, while inverse −*z*-oriented AEFs did not catalyze the reaction.

Three years later, the complementary IEDDA with AEFs in the *x* orientation was reported using mechanically controllable break junctions (MCBJs).^15^ The pyridyl termini of the tetrazine **3** were placed to bind to the gold electrodes of the MCBJ and thus orient the diene in the *x*-direction of the AEF. The IEDDA with dihydrofuran **4** along **TS-2** is followed by retro-DA to release N_2_ and ring-opening aromatization. All intermediates were characterized by their characteristic single-molecule currents, which allowed monitoring of different reaction kinetics by counts in the respective current histograms. Measured for 1 mM substrates in mesitylene/dichloromethane 4:1, AEFs catalyzed the final ring-opening aromatization with a linear dependence on applied voltage until saturation characterized by effective voltages around 20 mV and saturation around 200 mV. Consistent with theory, the IEDDA was not catalyzed by the AEFs in the *x* orientation.

Two years later, in 2021, graphene–molecule–graphene single-molecule junctions (GMG-SMJs) were used to explore NEDDA-EFC with flexible substrates.^16^ The terminal amino groups of the rigid-rod scaffold of **5** were reacted with carboxylates of oxygen plasma etched graphene. This tethered the maleimide dienophile flexibly along the scaffold for NEDDA with furan **6** along **TS-3**. Single-molecule reactions with furan as vapor and in toluene/DMSO solution were characterized by stochastic switching between six conductive states in *I-t* curves. In the histograms, they were assigned to the single substrate **5**, *exo*/*endo* reactant (charge-transfer) complexes with furan, and *exo*/*endo* products appearing at increasing temperature. Stochastic spikes observed only at high temperatures (>95 °C, furan vapor) and voltage were assigned to a zwitterionic Michael addition intermediate with a large macrodipole. Their reversible conversion into DA products, directly observed in single-molecule *I-t* curves, supported a mechanistic crossover that was predicted to occur at EEFs > 2.6 V/nm. Increasing DA products with decreasing polarity of toluene/DMSO mixtures at 300 mV was considered as support for operational EFC. Single-molecule *I-t* curves provided rates for reversible conversion of all intermediates and thus equilibria for the concerted DA mechanism. Lowered activation energies from single-molecule Arrhenius plots for forward and reverse cycloaddition with 300 mV compared to bulk solution supported the occurrence of DA-EFC with furan **6** in toluene at 25 °C, while equilibria depended on temperature and solvent polarity. Compared to single-molecule DA-EFC with oriented substrates **1** and **2**, these results with flexible substrates **5** and **6** were particularly important in the context of the present study because they supported that self-orientation of freely tumbling substrates in an AEF is sufficient for EFC to occur. In other words, the results imply that fixed substrate orientation in oriented AEFs is not a prerequisite for EFC of Diels–Alder reactions.

Originating from ultrafast reactions observed during electrospray mass spectrometry (ESI-MS), possible EFC in microdroplets is a topic that causes intense current controversy. As early as 2017, the Diels–Alder cyclization of 3,5-hexadienyl acrylate ester catalyzed by indium­(III) triflate was shown to work in bulk water but, according to MS detection, not in water microdroplets.^19^ In the same year, the same Zare group reported that, again according to in situ MS detection, the Sharpless [2σ + 2σ + 2π] cycloaddition between the strained quadricyclane and diethyl azodicarboxylate occurs in water microdroplets and attributed the observed rate enhancement to concentrating “on-water” effects originating from poor solubility.^20^ In 2024, the DA reaction between a furan derivative and *ortho*-diiodotetrafluorobenzene in water microdroplets, detected by MS in situ, has been explained with reactive oxygen species that produce *ortho*-diiodotetrafluorobenzene radical anions as “quasi-benzyne” dienophiles, that is by electron transfer chemistry rather than EFC.^21^

In the same year 2024, Diels–Alder chemistry in water and MeCN microdroplets has been explored with cyclopentadiene **7**, produced in situ by retro-DA from dimer **8**, and acrylonitrile **9** (Figure 1C).^22^ This is an attractive choice because competition with oxidation of substrate **7**, substrate dimers **8**, and product **10** into ketones **11–13** provides an internal control for the occurrence of radical redox chemistry instead of EFC. According to in situ MS detection, oxidation products were the main products under all of the conditions. They were proposed to originate from reactive oxygen species generated during vaporization, possibly involving EEFs. The ratio of DA against oxidation products decreased with increasing spray potential up to 5000 V, that is, increasing EEFs. Increasing DA product ratios with droplet flight time, nebulizing gas pressure, substrate concentrations, solvent apolarity, and apolar (but not polar) droplet size (spray solution flow rate) all supported that increasing effective concentrations by droplet evaporation and insolubility-related on-water confinement, rather than EEFs, catalyze DA reactions in microdroplets. These results were supported by computational simulations that highlighted concerns about ultrafast EEF fluctuations without orientation in the low picosecond time scale in water microdroplets.

The EFC of Diels–Alder reactions under organic synthesis conditions has not been reported so far. This is not surprising because, as stated at the beginning, the incompatibility with practical organic synthesis is the grand central problem with catalysis with externally applied electric fields. Extrapolating from early results with other electrochemical constructs,^5–9^ we^10^ and others^11^ have recently suggested that this challenge could be tackled with microfluidic capacitors. The key was the introduction of catalytic Helmholtz layers that translate intrinsically insufficient AEFs into effective electric fields (EEFs) that are strong enough to directly catalyze reactions.^42–44^ In this study, Helmholtz layer catalysis in microfluidic capacitors is used to realize the seemingly impossible and catalyze Diels–Alder reactions with external electric fields under organic synthesis conditions. The functional chiral Helmholtz layers constructed for this purpose are the first example for engineered Helmholtz layers that function as (i) primary catalyst and (ii) only source of chirality in asymmetric EFC under practical conditions.

The chemistry of anthrone **14** was selected to elaborate on DA-EFC under organic synthesis conditions not only because cycloaddition to electron-poor dienophiles **15–17** gives products **18–20** with interesting chirality (Figure 2). Alternative Michael addition (MA) products **21–23** can serve as intrinsic chemoselectivity probes for mechanistic transitions from concerted DA toward the stepwise MA alternatives that were predicted to occur at high voltage in above single-molecule GMG-SMJ experiments with tethered dienophile **5**.^16^ Moreover, the oxidation product **24** can serve as an intrinsic probe for transitions from EFC to the radical redox chemistry that dominates with **11–13** in water microdroplets (Figure 2 vs [Fig fig1]C).^21,22^

**Figure 2:**
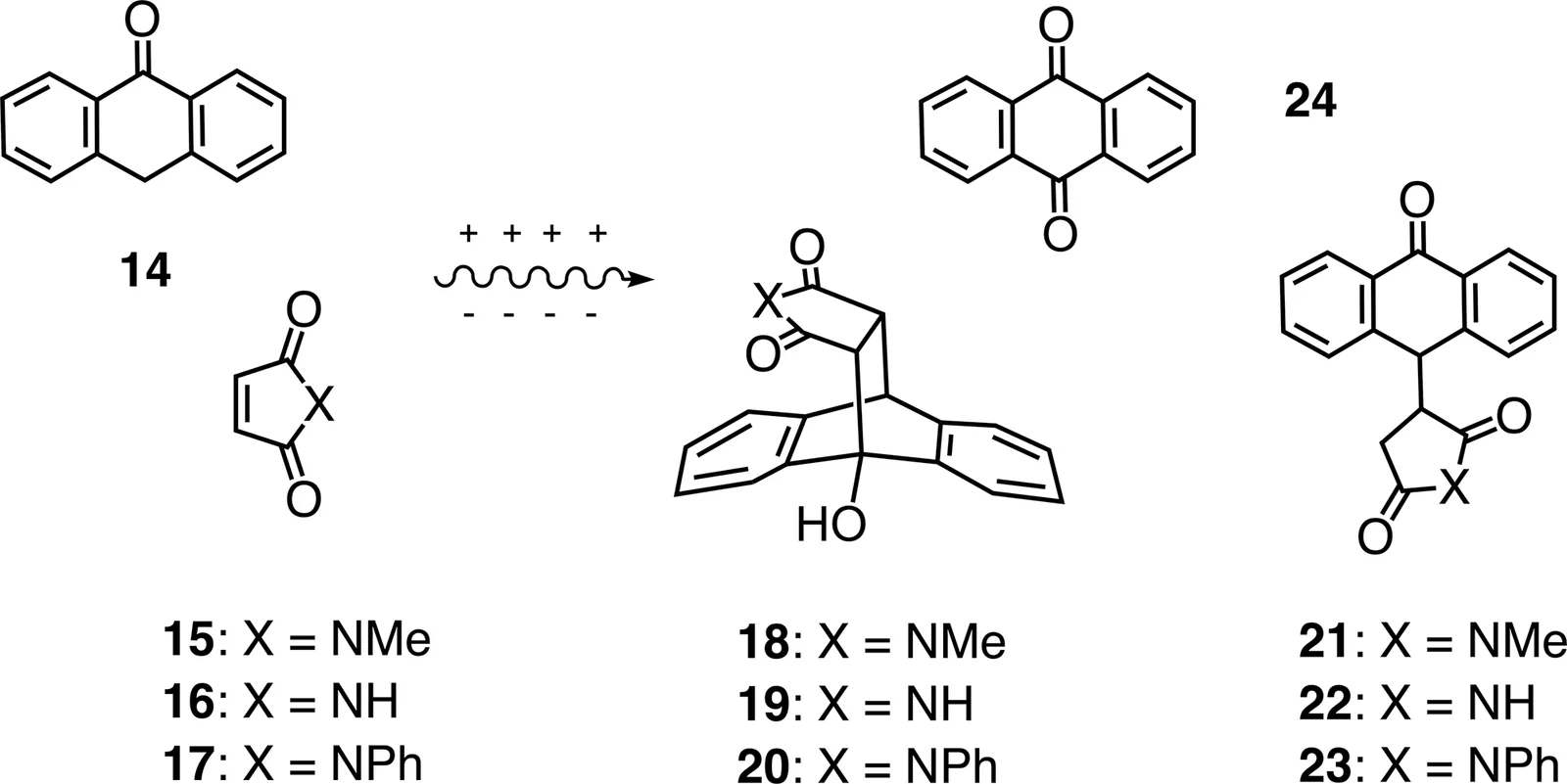
Current study on DA-EFC under organic synthesis conditions in microfluidic capacitors (charged wavy arrow) is realized with classical anthrone (**14**) chemistry because it offers products with interesting stereochemistry (**18–20**) and intrinsic probes for the occurrence of stepwise mechanisms (**21–23**) and radical redox chemistry (**24**).

Introduced in 1989 by Koerner and Rickborn,^45^ anthrone DA chemistry has attracted much attention for asymmetric base catalysis,^46–49^ first realized by Riant and Kagan.^50^ Michael addition products **21–23** have been observed along with DA product^51,52^ using Lewis acid^51^ and hydrogen-bonding catalysts,^47,52^ but apparently, not much Brønsted acid catalysis has been explored for anthrone DA chemistry.

## Results and Discussion

### Systems Design

The microfluidic reactors introduced to achieve electric-field catalysis compatible under practical organic synthesis conditions are commercially available (Figure 3A,B; photos: Figures S1–S3). They have received much recent attention for a completely different purpose.^53–57^ Namely, they are expected to make organic radical redox chemistry more controllable and safely scalable for use in industrial production. Intrigued by their properties, we thought that they could also be ideal to realize EFC in capacitor mode before the onset of electron transfer.

**Figure 3:**
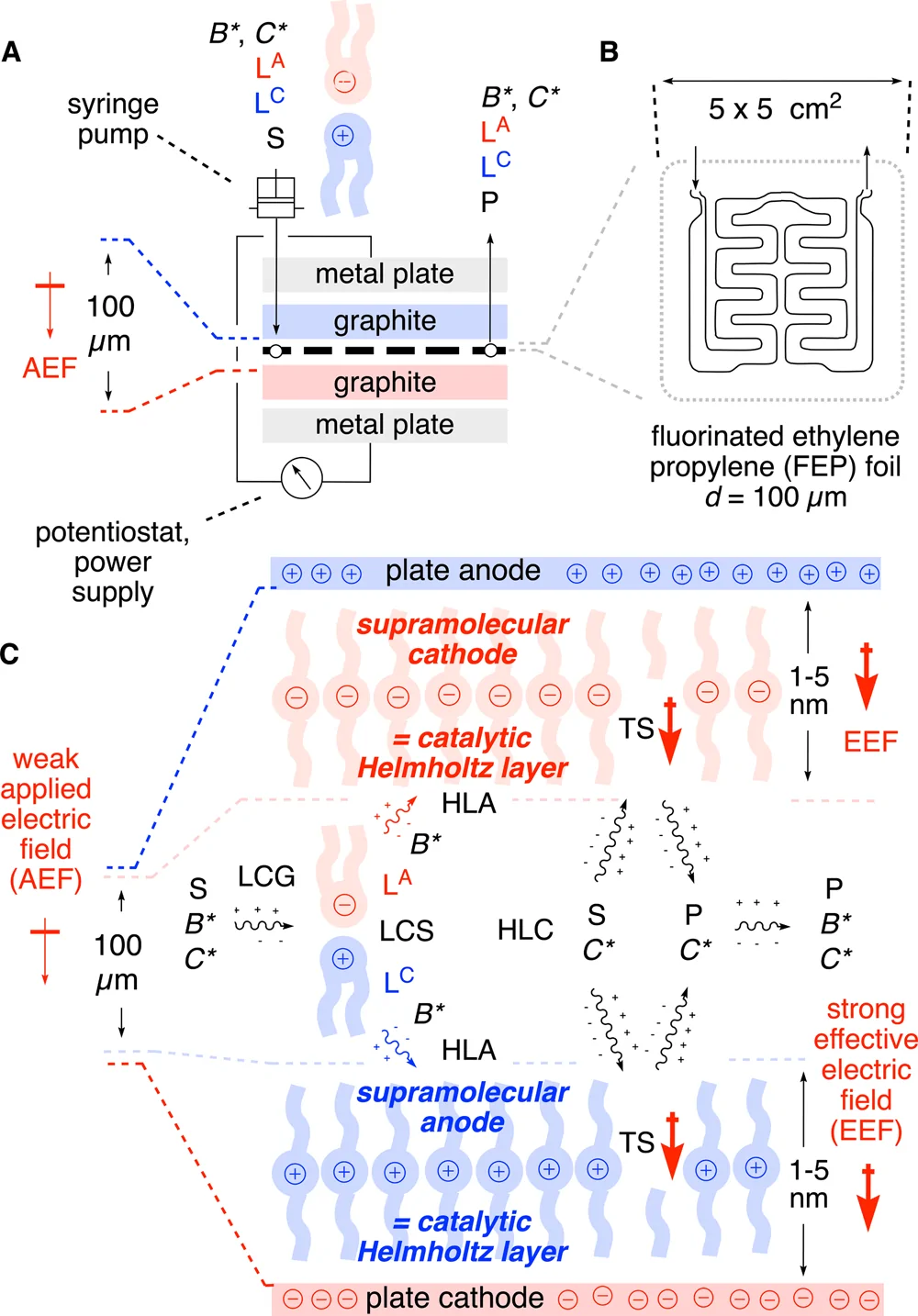
(A) Side view of microfluidic capacitors and (B) top view of the FEP foil with the flow system inbetween the graphite plate electrodes. (C) Emerging principles for EFC with externally applied fields under organic synthesis conditions: Layer component generation (LCG, in situ or by addition) is followed by layer component separation (LCS) in response to AEFs (optionally supported by breakers B*), layer components translocation and Helmholtz layer assembly (HLA) to translate weak AEFs into strong EEFs. These EEFs are strong enough for Helmholtz layer catalysis (HLC) by stabilizing the transition states (TS) from substrate (S) to products (P), supported by structural information transfer, also from (*) optional ion-pair breakers (B*) and catalysts (C*).

The selected microfluidic capacitors are composed of two 5 × 5 cm^2^ plate electrodes that are separated by a thin fluorinated ethylene propylene (FEP) foil with a flow channel. A thickness of *d* = 100–250 μm is commonly used; *d* < 100 μm is possible but increasingly problematic for routine work. This sandwich is then placed between metal plates and in the external housing that holds it all together. Graphite plates were the electrodes of choice because their surfaces show the least structural changes at high voltage. For maximal reproducibility, they were systematically polished on a dedicated machine before each experiment. The assembled capacitor was then connected to power supply or potentiostat to apply voltage and measure currents as a function of time. Finally, a syringe pump was connected to flow the reaction mixture through the capacitor at a controlled rate.

Electric fields are the applied voltage divided by the distance between the electrodes. According to theoretical predictions, EFC requires electric fields >1 V/nm to significantly stabilize transition states.^1–4,35–41^ These predictions imply that microfluidic capacitors with plate electrodes spaced >100 μm apart will not be capable of delivering strong enough AEFs.^42^ This conclusion called for a shift of attention from the AEFs as such to their consequences. In capacitors, these consequences are electric double layers (EDLs). Ion pairs present in apolar solvents separate and move to the respective electrode where they assemble into compact yet dynamic Helmholtz or Stern layers of uniformly charged ions, followed by less ordered diffuse layers with some ions of the opposite charge present as well.^58–62^ Polar solvents or substrates can also produce EDLs with Helmholtz layers composed of highly ordered, uniformly oriented neutral but polar molecules. With both ions or polar molecules, Helmholtz layers can be considered like supramolecular electrodes.^42^ The short distance to plate electrodes of opposite charge (1–5 nm) generates local effective electric fields (EEFs) that can exceed the AEFs by more than one million^42,44^ and are with >1 V/nm^11^ strong enough to directly catalyze reactions.

These considerations encouraged the rational design of Helmholtz layers components with functional motifs (Figure 3C). Their assembly into supramolecular 2D architectures would then position these functional motifs within the EEF they create to both enable EFC and contribute recognition motifs for information transfer to the product. Applying early lessons from cell penetration^63^ and sensing,^64–67^ the concept of catalytic Helmholtz layers was explored first with bioinspired ion pairs composed of polyarginine and hydrophobic anions for EFC of proline-catalyzed aldol condensations.^42,44^ Then it was validated and generalized with catalytic Helmholtz layers constructed from anionic Brønsted acids and ion-pair breakers.^43^ The results were compared to extract mechanistic principles for scalable EFC in catalytic Helmholtz layers (Figure 3C). Without going into details, the current understanding envisions a four-step mechanism composed of layer component generation (LCG), layer component separation (LCS), Helmholtz layer assembly (HLA), and Helmholtz layer catalysis (HLC).^42–44^ LCG can include added ion pairs,^42,43^ proton transfer,^44^ and even reactive intermediates (unpublished). LCS can be supported by optional ion-pair breakers,^43^ which then integrate into the Helmholtz layers, and, like optional organocatalysts,^42–44^ contribute to structural information transfer to the product. HLA of the separated components produces the EEFs needed for EFC. HLC can thus be defined as EFC with EEFs and (optional) structural information transfer from the compact layers of (engineered) EDLs.

### Diels–Alder Reactions in Catalytic Helmholtz Layers

Electric-field catalysis of Diels–Alder reactions in microfluidic capacitors was explored first with functional Helmholtz layers composed of the original, bioinspired polyarginine **25** (pR)^42,63–65^ as cationic layer component L^C^ and dodecyl sulfate (DS) **26** as anionic layer component L^A^ at 1 mM concentrations in CHCl_3_/DMSO 3:1 (Figure 4A). They were loaded together with 50 mM diene **14**, 75 mM dienophile **15**, and mesitylene as an internal standard into a syringe pump and flowed through the capacitor with a constant rate at room temperature (Figures 3, 4A). Yields *Y* after one passage through the microfluidic capacitor were determined by ^1^H NMR spectroscopy or chiral HPLC and described as microfluidic yields *Y*_m_. Microfluidic yields *Y*_m_^0^ report results without applied voltage. Under above conditions, *Y*_m_^0^ = 0% was recorded for DA product **18**; anthrone DA chemistry did not occur without electric fields (Figure 4B).

**Figure 4:**
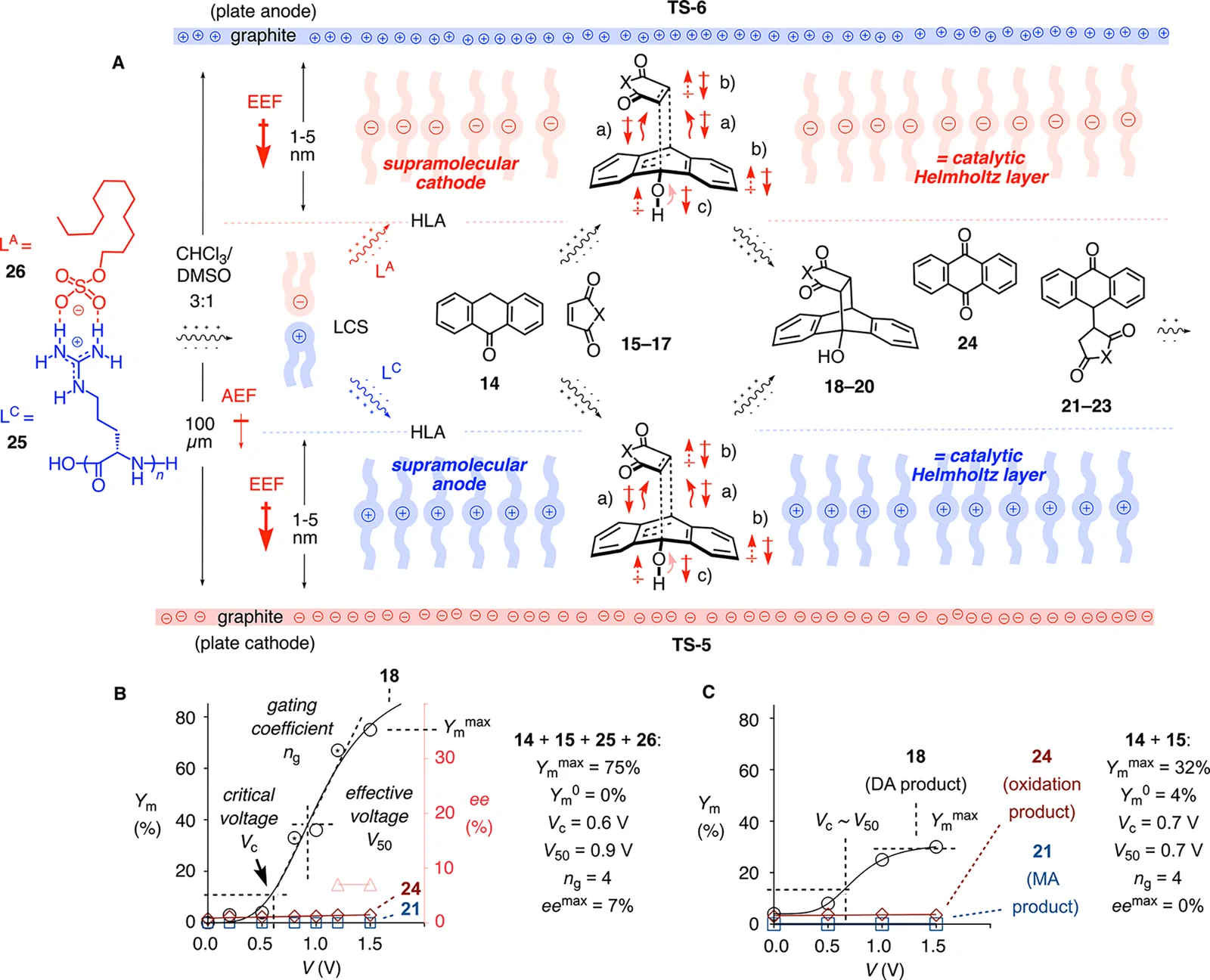
(A) DA-EFC in catalytic Helmholtz layers: Flowing into the AEF of the microfluidic capacitor, layer component separation (LCS) prepares for Helmholtz layer assembly (HCA) of L^C^ **25** and L^A^ **26** at the plate electrode of opposite charge to translate weak AEFs into EEFs. These EEFs are strong enough to catalyze NEDDA reactions of anthrone **14** with dienophiles **15–17** by (a) supporting intermolecular charge transfer along the reaction axis, (b) polarizing substrate complexes to induce supportive dipoles, perhaps up to charge transfer complex formation and inversion to IEDDA mechanisms, and (c) increasing phenol acidity up to deprotonation as formal invisible base catalysts, as outlined in transition states **TS-5** and **TS-6**. (B, C) YV curves (C) without and (B) with L^C^ **25** and L^A^ **26** (1 mM, 2 mol %): Dependence of the microfluidic yields *Y*_m_ (black, left, **18**: circles, **21**: squares, **24**: diamonds; *experimental replicates) and enantioselectivity (right, red, **18**: triangles) on the applied voltage *V* for **14** (50 mM) and **15** (75 mM) in CHCl_3_/DMSO 3:1, flow rate 5 μL/min, 100 μm foil, graphite electrodes, room temperature, with curve fit to Hill equation and indication of *Y*_m_^max^, *Y*_m_^0^, *V*_c_, *V*_50_, *n*_g_, and *ee* ^max^; X = NMe, NH, NPh.

The application of voltage afforded DA product **18**. Remarkably, *Y*_m_ did not increase linearly. Reminiscent of voltage-gated ion channels,^68^ sigmoidal YV curves^43^ were obtained instead (Figure 4B). The maximum experimental yield, *Y*_m_^max^ = 75%, was obtained at 1.5 V, and saturation could not be reached because DMSO as (co)­solvent is incompatible with high voltages. Saturation behavior in YV curves has been observed regularly and attributed to the assembly of the final catalytic Helmholtz layer whose activity cannot be further improved with increasing applied voltage.^42–44^ Curve fit to the Hill equation gave an effective voltage *V*_50_ = 0.9 V ((*Y*_m_^max^ + *Y*_m_^0^)/2), a critical voltage *V*_c_ = 0.6 V (*Y*_m_^0^ + 10%), and a gating (Hill) coefficient *n*_g_ = 4.

The removal of Helmholtz layer components L^C^ **25** and L^A^ **26** strongly reduced DA-EFC (Figure 4C). Most importantly, *Y*_m_^max^ dropped from *Y*_m_^max^ = 75% to *Y*_m_^max^ = 32%. Interestingly, YV curves remained voltage-gated with *n*_g_ = 4. The poor *Y*_m_^max^ and a minor *Y*_m_^0^ = 4% resulted in *V*_50_ = *V*_c_ = 0.7 V at *Y*_m_^50^ = 14%.

With and without L^C^ **25** and L^A^ **26**, EFC in microfluidic capacitors gave the DA product **18** almost exclusively (Figure 4B,C, circles). The oxidation product **24** was negligible and independent of AEFs (Figure 4B,C, diamonds). Contrary to disfavored DA reactions in microdroplets (Figure 1C),^22^ this selectivity supported the idea that successful DA reactions in catalytic Helmholtz layers occur by EFC without involving radical redox processes. The validity of this conclusion was supported by chronoamperometry (see below).

Significant amounts of MA product **21** were not obtained either (Figure 4B,C, squares). As described in the introduction, a transition from DA to MA products has been observed in single-molecule junctions at high temperature and high voltage (Figure 1B).^16^ Supported by high-level computational studies, this transition has been explained with the stabilization of the large macrodipole of the zwitterionic (rather than diradical) MA intermediate by strong electric fields. The absence of MA product **21** implied that in EEFs in catalytic Helmholtz layers, the formation of DA product **18** occurs by concerted cycloaddition and not by a stepwise mechanism.

### Mechanistic Considerations

These results were consistent with significant DA-EFC in functional Helmholtz layers composed of L^C^ **25** and L^A^ **26** that translate weak AEFs into strong EEFs of >1 V/nm. For EFC, these strong EEFs could support intermolecular charge transfer along the reaction axis in transition states **TS-5** and **TS-6** as expected from theoretical studies (Figure 4Aa vs [Fig fig1]A). Gradually increasing EEFs^58–62^ are expected to support diffusion of separate substrates or substrate complexes with strong macrodipoles across the diffuse into the compact catalytic Helmholtz layer. The dynamic, fluid 2D architectures also of this compact layer provide more than enough flexibility to adapt and integrate substrates spontaneously. Contributions from noncovalent interactions with Helmholtz layer components to support substrate integration and orientation are best documented by chirality transfer from chiral catalytic Helmholtz layers (Figure 4B, red triangles, see below for higher values).

Self-orientation of freely diffusing substrate complexes in EEFs has been supported experimentally for **TS-3** in single-molecule BJ studies^16^ and theoretically for **TS-4** (Figures 1B,C).^22^ Computational predictions also support that EEFs could further stabilize **TS-5** and **TS-6** by polarizing the substrates, inducing supportive dipoles (Figure 4Ab, 1A).^37,39^ Finally, EEFs could further catalyze the DA reaction by increasing phenol acidity up to deprotonation (Figure 4Ac). Here, EEFs would act as formal, invisible base catalysts, and base catalysis is well-established in anthrone DA chemistry.^46,50^

Weak anthrone DA chemistry without functional Helmholtz layers implied primary contributions from EFC under organic synthesis conditions (Figure 4B vs [Fig fig4]C). The residual, equally voltage-gated DA-EFC without functional Helmholtz layers (Figure 4C) was likely to occur in Helmholtz layers formed by uniformly oriented polar components of the solvent mixtures, here DMSO.^42^ In principle, proton transfer between the two substrates in response to AEFs^44^ could further produce cationic and anionic Helmholtz layers at the respective plate electrodes with reactant complexes activated by protonation and deprotonation, respectively. Although conceivable, such an intersubstrate proton transfer seemed rather unlikely with this system. Similar *V*_c_ values with and without dedicated Helmholtz components instead suggested that *V*_c_ reports mostly on the AEF needed to direct neutral polar substrate complexes toward either cationic or anionic Helmholtz layers to access **TS-5** and **TS-6**, respectively. The distinctly different *Y*_m_^max^ values with (75%) and without (32%) functional Helmholtz components would then support that the catalytic activity of Helmholtz layers including L^C^ **25** and L^A^ **26** exceeds Helmholtz layers only from presumably neutral, uniformly oriented substrates **14** and **15** in oriented solvent decisively. This difference evinced the catalytic activity of the engineered Helmholtz layers as the origin of EFC of Diels–Alder reactions under organic synthesis conditions. Quantitative support for this interpretation was obtained by chronoamperometry (see the following).

### Quininium Cations As Helmholtz Components

The dependence of DA-EFC on the nature of functional Helmholtz layers was explored first by replacing Helmholtz components L^C^ **25** and L^A^ **26** with *N*-benzylquininium chloride (QUIBEC, NBQ),^74,75^ which offers the quininium cation **27** as L^C^ and chloride as L^A^ (Figure 5).

**Figure 5:**
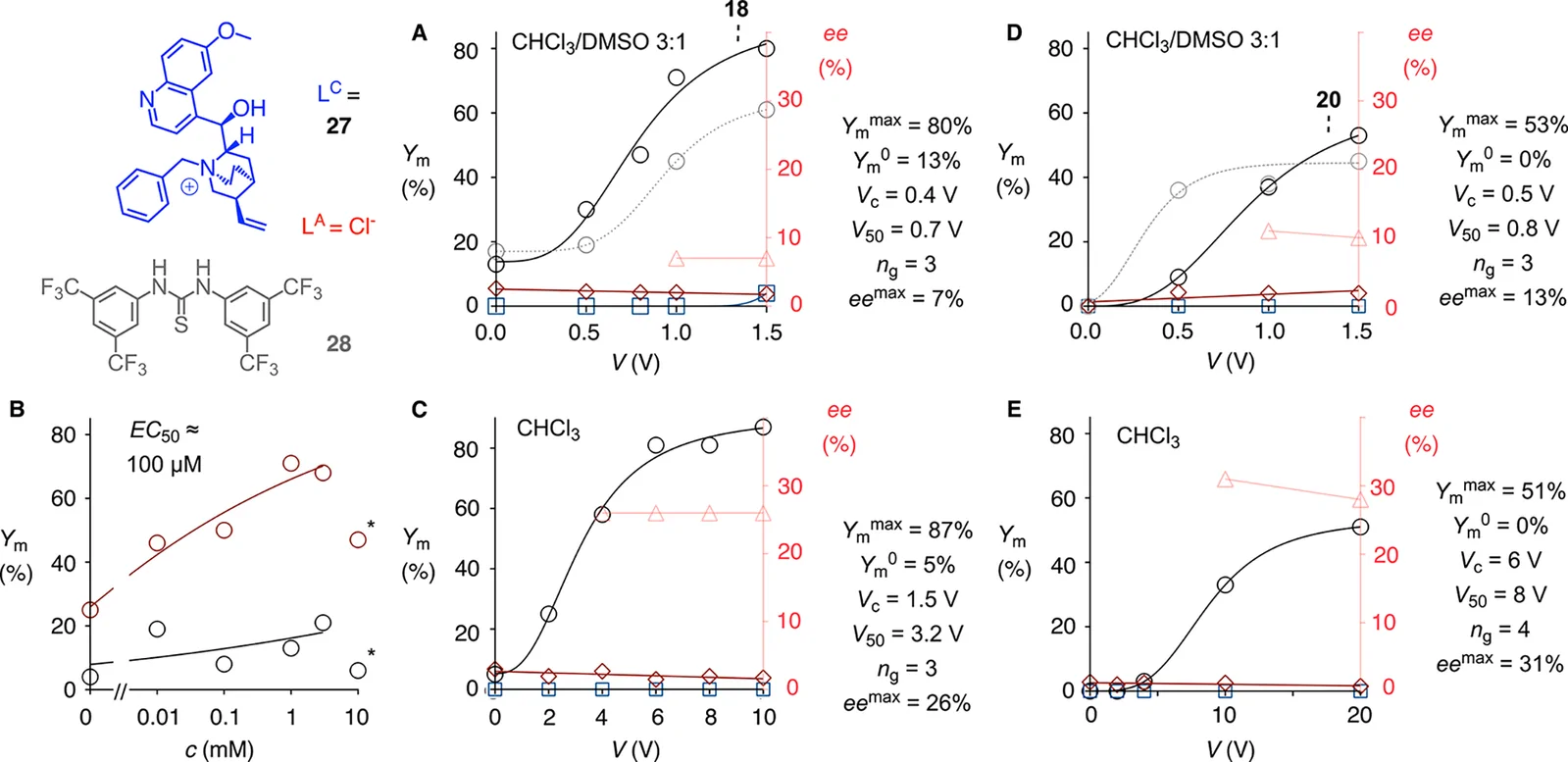
Dependence of DA-EFC on voltage (A, C–E) and concentration (B) of Helmholtz layer components (**27**), ion-pair breakers (**28**, A, D, gray), substrates (**17**, D, E), and solvents (CHCl_3_, C, E). YV curves for the reaction of diene **14** (50 mM) and dienophiles **15** (A–C) or **17** (D–E, 75 mM) in the presence of **27**-Cl (A, C–E: 1 mM; B: 0–10 mM) and **28** (A, D, gray, 2.5 mM) in CHCl_3_/DMSO 3:1 (A, B, D) or CHCl_3_ (C, E), reporting *Y*_m_ (left, **18/20**: circles, **21/23**: squares, **24**: diamonds) and enantioselectivity (right, red, **18/20**: triangles) as a function of the applied voltage *V* (A, C–E: varied; B: 0, black, or 1 V, dark red), flow rate 5 μL/min, 100 μm foil, graphite electrodes, room temperature, fit to Hill equation, affording *V*_c_, *V*_50_, and *n*_g_, *excluded from curve fit.

This Helmholtz layer substitution did not significantly disturb the DA-EFC between anthrone **14** and maleimide **15** (Figure 5A). The YV curve gave similar *Y*_m_^max^ (80%, 75%), *V*_c_ (0.4 V, 0.6 V), and *n*_g_ (3, 4), suggesting that the catalytic activity of the final Helmholtz layers did not change significantly. An increase of *Y*_m_^0^ from 0% to 13% implied weak contributions of the quininium cation to DA catalysis already without applied voltage. Preservation of the trace enantioselectivity supported, together with similar *Y*_m_^max^, that DA-EFC occurs preferably in the chiral cationic Helmholtz layer, the supramolecular anode, along **TS-5** rather than **TS-6** in the achiral, inorganic Helmholtz layers (Figure 4, see below).

### Catalyst Concentration Dependence

The concentration dependence of catalytic Helmholtz layers was determined for DA-EFC with 50 mM diene **14** and 75 mM dienophile **15** at 1 V for the quininium **27** as L^C^ and chloride as L^A^ in CHCl_3_/DMSO 3:1 (Figure 5B). The *Y*_m_^max^ of the reaction increased gradually to a maximum at around 1 mM, which was followed by a significant decrease at 10 mM. Hill analysis up to 3 mM afforded an EC_50_ ≈ 100 μM. Although dependent on conditions, particularly solubility in less polar solvent mixtures, Helmholtz components were used at the maximum at 1 mM. With the chiral Helmholtz layers serving as unique catalysts for DA-EFC, this calculated to 2 mol % and an EC_50_ = 0.2 mol % for formal catalyst loading, referring to monomeric L^C^ catalysts **27** relative to the anthrone substrate **14**.

Saturation behavior in the concentration dependence of catalytic Helmholtz layers was related to saturation behavior in the YV curves. Both were expected to mark completion of the assembly of the final catalytic Helmholtz layer, whose activity cannot be further improved with increasing voltage^42–44^ and even can decrease with too many Helmholtz components in the surrounding solution.

### Solvent Dependence

Solvent polarity is important for EFC under organic synthesis conditions in engineered Helmholtz layers for three main reasons. First, the onset of electron-transfer chemistry with certain, mostly polar solvents can limit access to high voltage. This is the case for DMSO above 2 V, for instance, but not for CHCl_3_.^42,44^ Second, in response to AEFs, polar solvents can orient uniformly, form EDLs, and generate EEFs that are strong enough for EFC.^42^ Intrinsic competition from polar solvents can complicate or even hinder the construction of catalytic Helmholtz layers with extrinsic components, which makes studies in apolar solvents preferable or at least less complicated. Third, the difficulty of ion-pair separation by AEFs for Helmholtz layer assembly increases with decreasing solvent polarity. With the current understanding of EFC, this should increase the critical voltage, that is, voltage gating, and the impact of ion-pair breakers.^43^

Contrary to the limitation of CHCl_3_/DMSO 3:1 to *V* < 2 V, YV curves in CHCl_3_ could thus be recorded up to *V* = 20 V without the emergence of oxidation products **24** (Figure 5C,E). The YV curves for substrates **14** and **15** with 2 mol % NBQ **27** in CHCl_3_ revealed preserved voltage gating (Figure 5C). With decreasing solvent polarity, product traces obtained without voltage decreased from *Y*_m_^0^ = 13% to *Y*_m_^0^ = 5%. This was as expected from the intrinsic reactivity in a stirred solution. Maximal microfluidic yields at high voltages increased slightly to a new record of *Y*_m_^max^ = 87%. A significant increase of the critical voltage with decreasing solvent polarity from *V*_c_ = 0.4 V to *V*_c_ = 1.5 V confirmed^43,76^ that *V*_c_ in less polar solvents reports on increasingly demanding ion-pair separation by the AEFs.

With decreasing solvent polarity, the enantioselectivity of DA-EFC of substrates **14** and **15** increased from *ee* ^max^ = 7% to *ee* ^max^ = 26% (Figure 5A,C, triangles).This result was important because it provided the first hints of asymmetric EFC with chiral information originating exclusively from the catalytic Helmholtz layers, rather than additional chiral organocatalysts such as prolines.^42^ This result thus supported the concept of asymmetric EFC by chirality transfer from chiral Helmholtz layers as realistic and worth further investigation.

### Substrate Dependence

DA-EFC with anthrone **14** in Helmholtz layers assembled from quininium **27** was compatible with maleimide **16** in place of maleimide **15** (Figure 2). Nearly identical YV curves were obtained in CHCl_3_/DMSO 3:1 (*Y*_m_^max^ = 73%; Figures S49, 5A). Removal of the quininium Helmholtz layers decreased DA-EFC with **16** as significantly as removal of pR Helmholtz layers decreased DA-EFC with **15** (*Y*_m_^max^ = 34%; Figures S51, 4C). The main difference was that the additional hydrogen-bond donor in **16** fully suppressed, rather than increased, the trace *ee* ^max^ = 7% with **15** (Figures S12, 5A).

In clear contrast, DA-EFC in the same chiral quininium Helmholtz layers **27**_**n**_ with anthrone **14** and phenyl maleimide **17** increased the stereoselectivity to *ee* ^max^ = 13% (Figure 5D). Compared with *Y*_m_^max^ = 80% for **18**, the yields of DA product **20** dropped to *Y*_m_^max^ = 53% (Figure 5D). This *Y*_m_^max^ = 53% was, however, impressive because yields without Helmholtz layers **27**_**n**_ were *Y*_m_^max^ = 13% with **17**, even lower than *Y*_m_^max^ = 32% with **15** (Figures S8 and S53).

In the less polar CHCl_3_, DA-EFC with maleimides **16** and **17** in place of maleimide **15** gave products **19** and **20** with up to 31% *ee* (Figures 5E and S23). Redox probes **24** were not obtained, also at high voltages (Figure 5E, diamonds). The best enantioselectivity *ee* ^max^ = 31% for DA-EFC was observed with phenyl maleimide **17** in CHCl_3_ at 10 V (Figure 5E, triangles). The recorded enantioselectivity with a phenyl substituent in maleimide **17** was consistent with theoretical predictions from the literature.^77^ For an asymmetric DA-EFC with up to *ee* ^max^ = 31%, the chiral catalytic quininium Helmholtz layers are the only possible origin of enantioselectivity (Figure 5E).

The dependence on flow rates was not remeasured because in previous studies with other systems, decreasing flow rates gave increasing yield, which was reasonable given the longer reaction times.^42–44^ Since parameters other than reaction time can contribute to changes, flow rates should not be considered as a single point in reaction kinetics without due caution. Poor accessibility to the reaction kinetics remains a technical limitation of microfluidic capacitors. The impact of alternating current and electrode material on previous systems was weak^42–44^ and thus not assessed in this study either.

### Chronoamperometry

Potential-step chronoamperometry (PS-CA) is routinely used to quantify EEFs in EDLs of capacitors from current decay kinetics and to probe for the occurrence of radical redox processes with currents at steady state.^11,69–73^ Applied to EFC under standard conditions, one of the two graphite electrodes was configured as working electrode, while the other served as both the counter and reference electrode. After the microfluidic reactor was filled with reaction solution containing quininium **27** as L^C^ and chloride as L^A^ together with substrates **14** and **15** in CHCl_3_/DMSO 3:1, PS-CA measurements were carried out while maintaining a continuous flow. A step potential was applied by imposing 1.0 V across the electrodes for 40 s to charge the EDL capacitor, followed by a step back to 0.0 V for 40 s to discharge (Figure 6Ab). The charging of the capacitor at 1.0 V produced a current that decreased rapidly, indicating the assembly of EDLs, including the catalytic Helmholtz layers (Figure 6Aa). The step change to 0.0 V caused a current to flow in the opposite direction to discharge the capacitor (applied first at the beginning in Figure 6A). The decay kinetics of this current report on EDL disassembly and should be similar to those of EDL assembly kinetics. This two-step procedure was repeated to probe the reproducibility.

**Figure 6:**
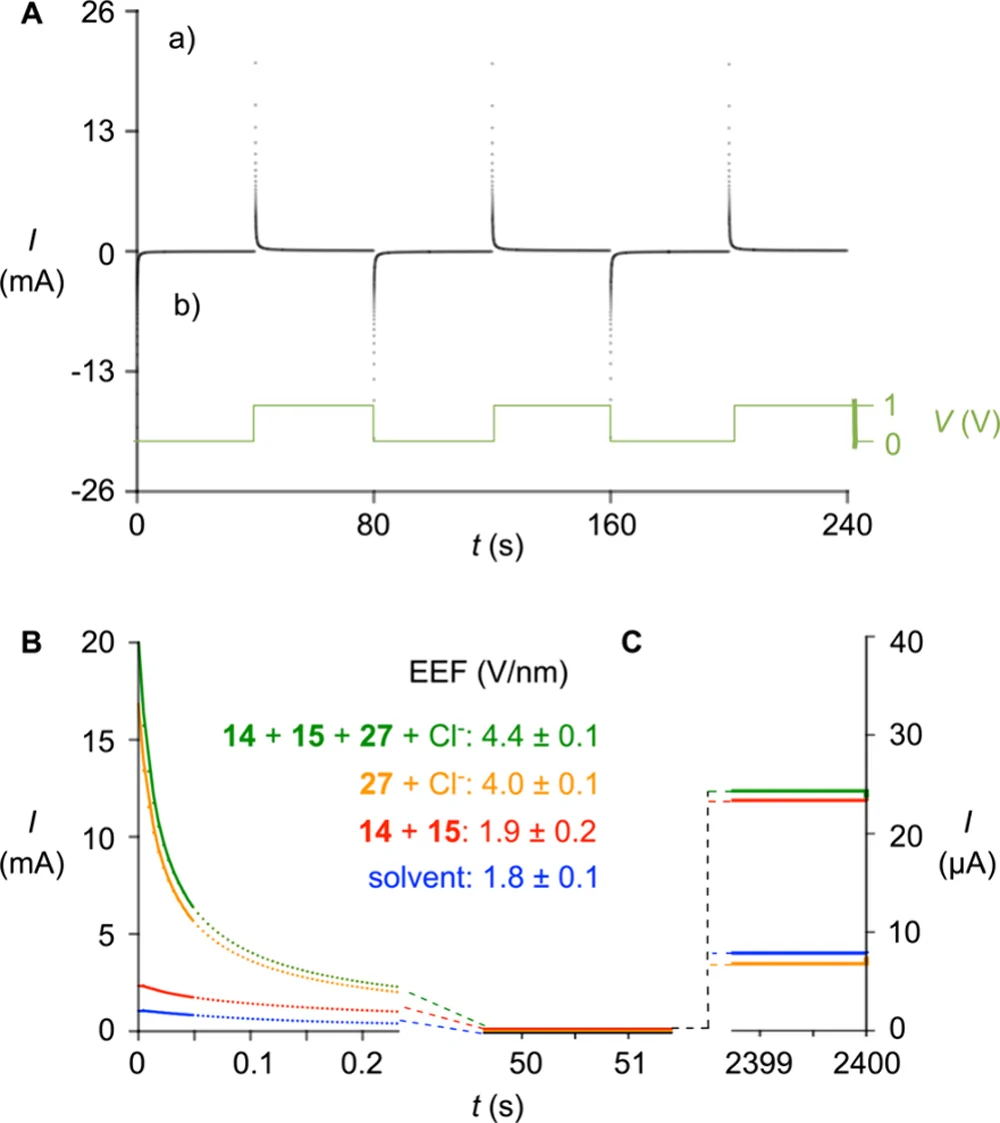
(A) Chronoamperogram with charge/discharge step potential changes of *V* = ±1.0 V in 40 s intervals (b) for **14** (50 mM), **15** (75 mM), and **27**-Cl (both 1 mM) in CHCl_3_/DMSO 3:1, flow rate 5 μL/min (a). (B, C) Representative charge profiles at (B) early and (C) late time points after application of *V* = +1.0 V at *t* = 0 s, without (blue) and with **14** and **15** (red), **27**-Cl (orange) and **14**, **15** and **27**-Cl (green) in CHCl_3_/DMSO 3:1. Very early data points (B) were fitted to monoexponential decay to calculate EEFs by applying the RC circuit model and Gauss law (SI, 3.6). Electrode area = 12 cm^2^. Errors represent standard errors of 3 charge kinetics analyses.

The initial charging/discharging kinetics of the capacitor were then fitted to an equivalent resistor-capacitor (RC) circuit model to determine the capacitance (for charging: *C* = 0.72 ± 0.04 mF, Figure 5B, green; for detailed procedures, see SI, 3.6, Equations S2–S6, Figures S57–S60).^69–71^ The EEF produced by the EDL was then estimated based on Gauss law, using mathematical average dielectric constants of the mixed solvent (Equation S7, Tables S27–S30).^11^ The EEF = 4.4 ± 0.1 V/nm obtained was consistent with the >1 V/nm needed to directly catalyze reactions. The absolute EEF values should however not be overinterpreted because of several fitting assumptions, such as unknown local dielectric constants (contributing to underestimates),^69–71^ ignored distance dependence, multiexponential decay due to complex EDLs, and so on.

Chronoamperograms were recorded for solutions containing anthrone **14** and maleimide **15** but not Helmholtz components **27**-Cl (Figures 6B, red, and S58). Smaller initial current and slower current decay revealed that the assembly of neutral EDLs with substrates **14** and **15** occurs but is less favorable than the one formed by charged Helmholtz components **27**-Cl (Figure 4A). This more reluctant assembly was consistent with neutral EDLs formed by oriented polar substrates **14** and **15** and substrate complexes. The EEF = 1.9 ± 0.2 V/nm from charge kinetics with substrate EDLs was much weaker than the EEF = 4.4 ± 0.1 V/nm obtained in the presence of the charged components **27**-Cl. This trend coincided with better DA-EFC (*Y*_m_^max^ = 80% vs *Y*_m_^max^ = 32%, Figures 5A, 4C) and thus provided corroborative quantitative evidence for both validity and significance of the concept of Helmholtz-layer catalysis.

The EEFs Helmholtz components **27**-Cl produced without substrates **14** and **15** were calculated to EEF = 4.0 ± 0.1 V/nm. These charged EEFs exceeded the neutral EEF = 1.9 ± 0.2 V/nm, although the concentration of ionic components was more than 50 times smaller (Figure 6B, red vs orange). The shape of chronoamperograms recorded for **27**-Cl did not depend strongly on the presence or the absence of substrates **14** and **15** (Figure 6B, orange vs green). These trends supported that substrates **14** and **15**, used at higher concentrations, insert via the diffuse layers into the still fluid, dynamic Helmholtz layers assembled by L^C^ **27** and proceed to cycloaddition as soon as their orientation aligns with the EEF produced by the catalytic Helmholtz layer. This alignment of reactant complexes along the EEF is supported intrinsically by their macrodipole and in addition by noncovalent interactions with catalytic Helmholtz layer, as evinced by chirality transfer.

The EEF = 1.8 ± 0.1 V/nm measured for the CHCl_3_/DMSO 3:1 solvent should originate from 2D assembly of uniformly oriented DMSO solvent molecules (Figure 6B, blue). Then, the dielectric constant of DMSO would probably be more appropriate to use, yielding an even smaller EEF (0.59 ± 0.04 V/nm).

After 50 s of EDL assembly at 1 V, very small microcurrents decreasing toward steady state were observed. After 40 min, which are usually allowed for equilibration before evaluating the reaction, decreasing presteady-state currents *I* ≈ 24 μA were obtained for architectures with substrates **14** and **15** with/without L^C^ **27**-Cl and *I* ≈ 7 μA for solvent with/without L^C^ **27**-Cl (Figure 6C). The slightly larger steady state currents *I* ≈ 24 μA with substrates **14** and **15** but without **27**-Cl were consistent with the more than 50 times higher concentrations used as well as their integration into EDL architectures. These presteady-state microcurrents <25 μA thus reported final small adjustments of the EDL architectures. Their smallness excluded significant contributions from radical redox chemistry to the found DA-EFC (Equation S8).^11,12,73^ Most importantly, presteady-state currents *I* ≈ 24 μA did not change in the presence of Helmholtz components **27**-Cl (Figure 6C, green vs orange), while the microfluidic yield of DA products **18** almost tripled from 26% to 71% at 1 V (Figures 5A, 4C).

Negligible contributions from faradaic processes were consistent with the absence of the oxidation product **24**. This result was important because it provided support for the existence and significance of the DA-EFC under organic synthesis conditions.

### Ion-Pair Breakers

Ion-pair breakers were introduced this year following the emergence of voltage-gated EFC.^43^ Ion-pair separation in response to AEFs in microfluidic capacitors was considered to account for the observed critical voltage needed to access EFC. For functional anions, neutral anion binders centered around Schreiner thiourea **28** ^78–81^ were thus considered to bind to these anions, separate them from their countercations, and integrate structural information into the anionic Helmholtz layers (Figure 3C). A significant decrease of critical voltages, an increase of yields, and changes in selectivity in the presence of ion-pair breakers provided experimental support for the concept.^43^

Bifunctional thiourea catalysis of anthrone DA chemistry was reported.^47^ However, the presence of the original ion-pair breaker **28** did not significantly change the YV curve for DA-EFC between anthrone **14** and maleimides **15** or **17** in Helmholtz layers made from quininium **27** and chloride in CHCl_3_/DMSO 3:1 (Figures 5A and 5D, black vs gray). Critical and effective voltages were affected but remained at comparably low values. This poor activity of ion-pair breaker **28** was meaningful because they were introduced for tighter ion pairs in less polar solvents characterized by *V*_c_ = 5.0 V.^43^ The weaker ion pairs in CHCl_3_/DMSO 3:1 naturally gave smaller *V*_c_ = 0.5 V. The poor activity of Schreiner thiourea **28** thus supported that for this DA-EFC in CHCl_3_/DMSO 3:1, ion-pair separation by AEFs is not the limiting process reported by the critical voltage *V*_c_. This finding provided corroborative support that in this system, the formation of oriented neutral reactant complexes in the catalytic Helmholtz layers determines the critical voltage *V*_c_.

### Helmholtz Component Screening

Scope and limitations of functional Helmholtz layer architectures to catalyze DA reactions with externally applied electric fields were explored under standard conditions with anthrone **14** and maleimide **15** in CHCl_3_/DMSO 3:1 at a flow rate of 5 μL/min and graphite electrodes separated by 100 μm (Figure 7). CHCl_3_/DMSO 3:1 was preferred over the more attractive, less polar CHCl_3_ to prevent that eventual solubility problems of the tested Helmholtz components would influence the results. Anionic Helmholtz layer components were varied first, while the original, bioinspired polyarginine **25** ^42,63^ was kept constant as cationic Helmholtz component (Figures 4B, 7A, Table 1). Polyarginine with chloride counterions was combined with different anions as sodium salts, which meant that chloride and sodium ions were present in all measurements as additional ions at constant concentrations limited by their solubility.

**Figure 7:**
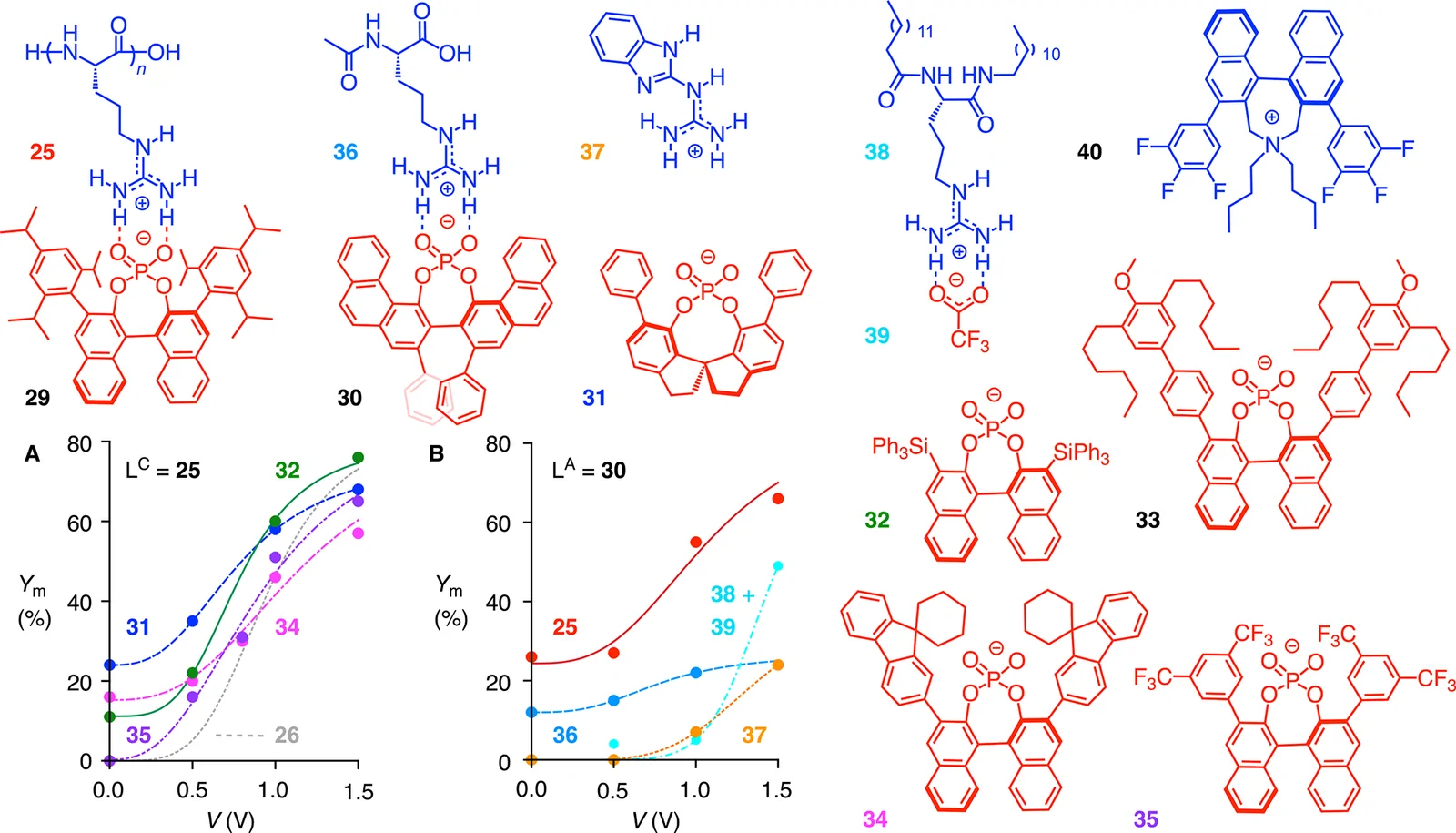
Helmholtz layer component screening for DA-EFC, with YV curve summary for (A) L^A^ and (B) L^C^ variations. YV curves for the reaction of diene **14** (50 mM) and dienophile **15** (75 mM) in the presence of components **25**, **26**, and **30–39** (1 mM, 2 mol %), reporting *Y*_m_ (**18**; *Y*_m_^max^ of **21** and **24** < 4%) as a function of the applied voltage *V* (CHCl_3_/DMSO 3:1, flow rate 5 μL/min, 100 μm foil, graphite electrodes, room temperature, fit to Hill equation), for (A) L^C^ = **25** with L^A^ = **26** (gray dotted line), **31** (blue dashed), **32** (green solid), **34** (magenta dash-dot), or **35** (purple dash-dot-dot), and (B) L^A^ = **30** with L^C^ = **25** (red solid), **36** (blue dashed), or **37** (orange dot) and L^A^ = **39** with L^C^ = **38** (cyan dash-dot-dot).

**Table 1:** Helmholtz Component Screening for DA-EFC^a^

entry	L^C^ ^b^	L^A^ ^c^	*Y*_m_^max^ (%)^d^	*Y*_m_^0^ (%)^e^	*V*_c_ (V)^f^	*V*_50_ (V)^g^	*n*_g_ ^h^	*ee* ^max^ (%)^i^
1	**25**	**26**	75	0	0.6	0.9	4*	7
2	–	–	32	4	0.7	0.7	4	0
3	**27**	Cl^–^	80	13	0.4	0.7	3*	7
4	**25**	**29**	72	24	0.6	0.7	6*	15
5	**25**	**30**	66	26	0.7	0.9	3*	15
6	**25**	**31**	68	24	0.5	0.7	3	7
7	**25**	**32**	76	11	0.5	0.8	4	0
8	**25**	**33**	63	7	0.7	0.9	7	0
9	**25**	**34**	57	16	0.6	0.9	3	0
10	**25**	**35**	65	0	0.4	0.8	3	0
11	Na^+^	**29**	61	16	0.6	0.8	3	0
12	**36**	**30**	22	12	1.0	1.0	3	0
13	**37**	**30**	24	0	1.1	1.2	5	0
14	**38**	**39**	49	0	1.1	1.3	8*	0
15	**40**	Br^–^	61	9	0.6	0.9	3*	11

a Results of Hill analysis for DA-EFC of diene **14** (50 mM) and dienophile **15** (75 mM) to **18** in the presence of **25–27** and **29–40** (1 mM, 2 mol %) in CHCl_3_/DMSO 3:1, flow rate 5 μL/min, 100 μm foil, graphite electrodes, at room temperature.

b Cationic layer component.

c Anionic layer component.

d Highest observed *Y*_m_ (%).

e *Y*_m_ (%) observed at 0 V.

f The critical voltage equals *Y*_m_ = *Y*_m_^0^ + 10%.

g The effective voltage is equal to *Y*_m_ = (*Y*_m_^max^ + *Y*_m_^0^)/2.

h Gating coefficients, estimated by Hill fits; uncertainties ≤ 1 unless marked with *.

i *ee* values, estimated by chiral HPLC analysis.

Replacement of the original DS **26** by (*S*)-TRIP **29**, a popular chiral binaphthylphosphodiester,^82–85^ caused the appearance of weak activity *Y*_m_^0^ = 24% without applied voltage (Table 1, entry 4, Figure S24). Present with *Y*_m_^0^ = 16% also in the absence of pR **25**, this observation was best explained with very weak base catalysis by (*S*)-TRIP **29** (entry 11). The YV curve with (*S*)-TRIP **29** and pR **25** showed a voltage-gated EFC with unchanged *V*_c_ = 0.6 V. Compared with DS **26**, lower *V*_50_ = 0.7 V and higher *n*_g_ = 6 suggested that (*S*)-TRIP **29** facilitated the assembly of catalytic Helmholtz layers. With *Y*_m_^max^ = 72%, the catalytic activity of the final Helmholtz layer was only marginally weaker compared to that of DS **26** (*Y*_m_^max^ = 75%, entry 1 vs 4). Replacement of an achiral DS-Helmholtz cathode **26**_**n**_ with a chiral (*S*)-TRIP assembly **29**_**n**_ increased the maximal enantioselectivity from *ee* ^max^ = 7% to *ee* ^max^ = 15%. These relatively small increases of *Y*_m_^max^ and *ee* ^max^ with chiral Helmholtz cathodes supported that DA-EFC mainly occurs in the chiral pR-Helmholtz anodes **25**_**n**_ along **TS-5**. A decrease to *Y*_m_^max^ = 61% and *ee* ^max^ = 0% upon replacement of this chiral pR anode **25**_**n**_ by inorganic sodium was consistent with this interpretation (entry 11).

The trends observed with (*S*)-TRIP **29** were overall preserved with chiral phosphodiesters **30** and **31** as partners of pR **25**, particularly with regard to the presence of weak enantioselectivity and weak base catalysis without voltage (Table 1, entries 5 and 6, Figures 7A, blue, S27, S29). This minor enantioselectivity fully disappeared, rather than increased, with more sophisticated chiral phosphodiesters **32–35** known or extrapolated (**33**, **34**) from asymmetric catalysis^82–85^ (entries 7–10, Figure 7A, green, magenta, purple, Schemes S5–S9). Moreover, yields for EFC at high voltage and base catalysis without voltage decreased down to *Y*_m_^max^ = 63% and *Y*_m_^0^ = 0%, respectively (entries 8, 10). This poor performance of more sophisticated chiral phosphodiesters confirmed that the emerging principles of scalable EFC at high voltage differ and can be almost contrary to established synthetic methodology.

The dependence on their nature demonstrated that anionic Helmholtz components contribute to DA-EFC at constant cationic Helmholtz components. However, the most important contributions to the yield and enantioselectivity of DA-EFC seemed to originate from the constant cationic Helmholtz component **25**. More sophisticated anionic Helmholtz components failed to improve much from DS **26** and at worst disturbed the catalytic Helmholtz layers. The conclusion that **TS-5** in catalytic Helmholtz layers accounts for most of DA-EFC was already supported by preserved top performance upon substitution of pR **25** with the quininium **27** and the poor activity of ion-pair breakers **28**, integrating into the anionic rather than the cationic Helmholtz layer (Figures 4A, 5A,D). This interpretation was supported by more important changes in response to substitution of the cationic rather than the anionic Helmholtz layer component. Arginine monomers **36** in place of pR **25** were incapable of significant EFC, although they were teamed up with phosphodiesters **30** that were impressive with **25** (Table 1, entries 5 vs 12, Figure 7B, blue). *Y*_m_^max^ = 22% was below *Y*_m_^max^ = 32% without charged catalytic Helmholtz layers. The presence of a carboxylic acid in the Helmholtz component **36** might contribute to this inactivity. However, also the carboxylate-free guanidinium variant **37**, together with the same phosphodiester **30**, performed poorly (*Y*_m_^max^ = 24%; entry 13 Figure 7B, orange).

The more hydrophobic arginine monomer **38** together with the TFA anion **39** showed an unusual YV curve with strong voltage gating (entry 14, Figure 7B, cyan). *V*_c_ = 1.1 V and *n*_g_ = 8 characterized a sudden turn-on of DA-EFC from *Y*_m_^0^ = 0% to *Y*_m_^max^ = 49% around *V*_50_ = 1.3 V (*Y*_m_^max^ = 75% at 2 V). This interesting cooperativity could originate from the assembly into cationic Helmholtz layers reminiscent of those of biomimetic lipid monolayers.

The Maruoka phase-transfer catalyst **40** ^86^ having an ammonium cation next to a chiral binaphthyl as in the phosphodiester series provided corroborative support that DA-EFC occurs preferably in the cationic Helmholtz layer along **TS-5** (Figure 4A). High *Y*_m_^max^ = 61% against nearly negligible *Y*_m_^0^ = 9% coincided with the reemergence of stereoselectivity at *ee* ^max^ = 11% (Table 1, entry 11, Figure S47). This weakly asymmetric DA-EFC was in the range of the best chiral cations pR **25** (*ee* ^max^ = 15%, entries 4 and 5) and quininium **27** (*ee* ^max^ = 7%, entry 3). However, while asymmetric DA-EFC with quininium Helmholtz layers **27_n_** maximized to *ee* ^max^ = 26–31% in less polar CHCl_3_ at high voltage (Figure 5E), a similar jump was not observed with Maruoka Helmholtz layers **40_n_**. Enantioselectivity remained at *ee* ^max^ = 15% in CHCl_3_ (*Y*_m_^max^ = 90%, Figure S55).

The strong responsiveness to variation of cationic Helmholtz layer components, exceeding the functional impact of anionic components by far, supported that DA-EFC occurs preferably in cationic Helmholtz layers along **TS-5** (Figure 4). This interpretation was interesting because EEFs acting like invisible base catalysts to activate the DA reaction by phenol deprotonation should perform better in anionic rather than cationic Helmholtz layers, that is, **TS-6**, contributing another anionic layer component (Figure 4c). DA-EFC occurring in cationic Helmholtz layers along **TS-5** thus supported that the EEFs catalyzed the cycloaddition directly by enabling the charge transfer along the reaction axis in reactant complexes, as described by theory.^1–4,14–16,19–22,36–41^ This interpretation implied that, also supported by theory,^22^ the oriented EEFs produced by the catalytic Helmholtz layers will orient integrated reactant complexes according to their dipole and thus install the directionality needed for EFC (Figures 1 and 4A).

## Conclusions

The Diels–Alder reaction has emerged as the primary reaction in electric field catalysis.^1–4,14–16,19–22,36–41^ Despite this central importance, electric-field catalysis (EFC) of Diels–Alder (DA) reactions has not been possible so far under practical organic synthesis conditions.^22^ In this study, this seemingly impossible is achieved. Within microfluidic capacitors, functional Helmholtz layers are installed to translate the applied electric fields (AEFs) into effective electric fields (EEFs) that are strong enough to directly catalyze the reactions. In these EEFs, the yield of DA products from maleimides and the classical anthrones increases nonlinearly with increasing applied voltage, i.e., shows voltage-gated EFC. Chronoamperometry measurements demonstrate the formation of EEFs in the catalytic Helmholtz layers. With up to 4.4 V/nm, they are strong enough to directly catalyze the reactions. Negligible steady-state currents exclude significant contributions from Faradaic processes under these conditions. These chronoamperometry data are valuable because they quantitatively demonstrate the occurrence and relevance of EFC under scalable bulk conditions in rationally designed functional Helmholtz layers.

We define Helmholtz-layer catalysis (HLC) as EFC with EEFs and (optional) structural information transfer from the compact layers of (engineered) electric double layers (EDLs). Beyond realizing scalable DA-EFC, this study marks the first time that HLC is achieved without the presence of additional small-molecule catalysts that are first activated by EEFs to then accelerate the reaction of interest. In this study, the functional Helmholtz layer is the only catalyst. Enantioselectivity up to 31% *ee*, which originates exclusively from these chiral catalytic Helmholtz layers, is equally unprecedented. Together with the strong dependence on layer components, this unprecedented chirality transfer from chiral catalytic Helmholtz to the transition state of the reaction highlights how noncovalent interactions from engineered Helmholtz layers can contribute to catalysis beyond their EEFs. The understanding of the assembly of catalytic Helmholtz layers on the molecular level thus emerges as a primary topic for supramolecular chemistry experts to contribute to the development of a conceptual framework for an organic synthesis with externally applied electric fields under practical synthesis conditions. The promises, from basic organic chemistry to sustainable industrial production and the understanding of the origin of life, are tantalizing.

## Supplementary Material



## Data Availability

The data that support the findings of this study are openly available in Zenodo at 10.5281/zenodo.20810151.

## Notes

Material and methods, including abbreviations, synthesis of reference compounds and Helmholtz components (Schemes S1–S9), construction of microfluidic capacitors, including photos (Figures S1–S3), general procedures for DA-EFC, with Helmholtz layer assembly, HPLC and NMR analysis (Scheme S10, Equation S1, Figures S4–S5), all YV curves with original data and data analysis (Figures S6–S56, Tables S1–S26), chronoamperometry procedures and analysis (RC circuit, Gauss’s law, Equations S2–S8, Figures S57–S60, Tables S27–S30), radical scavenger experiments (Figures S61–S62, Tables S31), supporting references, and original ^1^H NMR spectra for all new compounds (Figures S63–S75) (PDF)

## References

[1] Shaik S. (2024). My Vision
of Electric-Field-Aided Chemistry in 2050. ACS
Phys. Chem. Au.

[2] Ciampi S., Darwish N., Aitken H. M., Díez-Pérez I., Coote M. L. (2018). Harnessing Electrostatic Catalysis in Single Molecule,
Electrochemical and Chemical Systems: A Rapidly Growing Experimental
Tool Box. Chem. Soc. Rev..

[3] Yang C., Guo Y., Zhang H., Guo X. (2025). Utilization of Electric Fields to
Modulate Molecular Activities on the Nanoscale: From Physical Properties
to Chemical Reactions. Chem. Rev..

[4] Boxer S. G., Head-Gordon T. (2025). Introduction:
Electric Fields in Chemistry and Biology. Chem.
Rev..

[5] Gorin C. F., Beh E. S., Kanan M. W. (2012). An Electric Field-Induced Change
in the Selectivity of a Metal Oxide-Catalyzed Epoxide Rearrangement. J. Am. Chem. Soc..

[6] Gorin C. F., Beh E. S., Bui Q. M., Dick G. R., Kanan M. W. (2013). Interfacial
Electric Field Effects on a Carbene Reaction Catalyzed by Rh Porphyrins. J. Am. Chem. Soc..

[7] Akamatsu M., Sakai N., Matile S. (2017). Electric-Field-Assisted Anion-π
Catalysis. J. Am. Chem. Soc..

[8] Chat K., Maksym P., Kamiński K., Adrjanowicz K. (2022). Stereoregulation,
Molecular Weight, and Dispersity Control of PMMA Synthesized via Free-Radical
Polymerization Supported by the External High Electric Field. Chem. Commun..

[9] Tu W., Dzienia A., Maksym P., Duarte D. M., Unni A. B., Chat K., Kamiński K., Adrjanowicz K. (2022). Electric-Field
Assisted Ring-Opening Polymerization: On the Kinetics and Product
Properties of DGEBA/Aniline Model System. Polymer.

[10] Gutiérrez
López M. Á., Ali R., Tan M.-L., Sakai N., Wirth T., Matile S. (2023). Electric-Field-Assisted
Anion-π Catalysis on Carbon Nanotubes in Electrochemical Microfluidic
Devices. Sci. Adv..

[11] Sevim S., Sanchis-Gual R., Franco C., Aragonès A. C., Darwish N., Kim D., Picca R. A., Nelson B. J., Ruiz E., Pané S., Díez-Pérez I., Puigmartí-Luis J. (2024). Electrostatic
Catalysis of a Click
Reaction in a Microfluidic Cell. Nat. Commun..

[12] Westendorff K. S., Hülsey M. J., Wesley T. S., Román-Leshkov Y., Surendranath Y. (2024). Electrically
Driven Proton Transfer Promotes Brønsted
Acid Catalysis by Orders of Magnitude. Science.

[13] Dinakar B., Westendorff K. S., Torres J. F., Dakhchoune M., Groenhout K., Ewell N., Surendranath Y., Dincă M., Román-Leshkov Y. (2025). Elucidating Electric
Field-Induced Rate Promotion of Brønsted Acid-Catalyzed Alcohol
Dehydration. J. Am. Chem. Soc..

[14] Aragonès A. C., Haworth N. L., Darwish N., Ciampi S., Mannix E. J., Wallace G. G., Diez-Perez I., Coote M. L. (2016). Electrostatic Catalysis
of a Diels-Alder Reaction. Nature.

[15] Huang X., Tang C., Li J., Chen L.-C., Zheng J., Zhang P., Le J., Li R., Li X., Liu J., Yang Y., Shi J., Chen Z., Bai M., Zhang H.-L., Xia H., Cheng J., Tian Z.-Q., Hong W. (2019). Electric Field-Induced
Selective Catalysis of Single-Molecule Reaction. Sci. Adv..

[16] Yang C., Liu Z., Li Y., Zhou S., Lu C., Guo Y., Ramirez M., Zhang Q., Li Y., Liu Z., Houk K. N., Zhang D., Guo X. (2021). Electric Field-Catalyzed
Single-Molecule Diels-Alder Reaction Dynamics. Sci. Adv..

[17] Zhan G., Cai Z.-F., Martínez-Abadía M., Mateo-Alonso A., De Feyter S. (2020). Real-Time Molecular-Scale Imaging
of Dynamic Network Switching between Covalent Organic Frameworks. J. Am. Chem. Soc..

[18] Aziz M., Prindle C. R., Lee W., Zhang B., Schaack C., Steigerwald M. L., Zandkarimi F., Nuckolls C., Venkataraman L. (2024). Evaluating
the Ability of External Electric Fields to Accelerate Reactions in
Solution. J. Phys. Chem. B.

[19] Banerjee S., Gnanamani E., Yan X., Zare R. N. (2017). Can All Bulk-Phase
Reactions Be Accelerated in Microdroplets?. Analyst.

[20] Bain R. M., Sathyamoorthi S., Zare R. N. (2017). “On-Droplet” Chemistry:
The Cycloaddition of Diethyl Azodicarboxylate and Quadricyclane. Angew. Chem., Int. Ed..

[21] Chen H., Wang R., Chiba T., Foreman K., Bowen K., Zhang X. (2024). Designer “Quasi-Benzyne”: The Spontaneous Reduction
of Ortho-Diiodotetrafluorobenzene on Water Microdroplets. J. Am. Chem. Soc..

[22] Gong K., Nandy A., Song Z., Li Q.-S., Hassanali A., Cassone G., Banerjee S., Xie J. (2024). Revisiting the Enhanced
Chemical Reactivity in Water Microdroplets: The Case of a Diels-Alder
Reaction. J. Am. Chem. Soc..

[23] Zhang F., Tian Y., Wei H., Yue T., Gao Y., Li X., Zare R. N., Zhang X., Wang Z. (2025). Interfacial Electric
Fields Modulate Redox Reactions in Abiological Coacervates. J. Am. Chem. Soc..

[24] Dong J., Xu J., Meng Z.-D., Nan Z.-A., Li W., Zare R. N., Tian Z.-Q., Fan F. R. (2025). Microdroplet Cascade Catalysis for
Highly Selective Production of Propylene Glycol under Ambient Conditions. J. Am. Chem. Soc..

[25] Chen H., Li X., Li B., Chen Y., Ouyang H., Li Y., Zhang X. (2025). Microdroplet Chemistry with Unactivated Droplets. J. Am. Chem. Soc..

[26] Yan X., Bain R. M., Cooks R. G. (2016). Organic Reactions in Microdroplets:
Reaction Acceleration Revealed by Mass Spectrometry. Angew. Chem., Int. Ed..

[27] Li J., Xu J., Song Q., Zhang X., Xia Y., Zare R. N. (2024). Methane
C­(Sp3)-H Bond Activation by Water Microbubbles. Chem. Sci..

[28] Gao X., Zhou P., Yuan X., Li X., Li B., Xia Y., Meng Y., Zare R. N., Zheng T., Zhang X. (2025). Peeling Tape
Produces Strong Electric Fields via Stick-Slip Friction That Drive
Chemical Reactions. Proc. Natl. Acad. Sci. U.
S. A..

[29] Warshel A., Sharma P. K., Kato M., Xiang Y., Liu H., Olsson M. H. M. (2006). Electrostatic
Basis for Enzyme Catalysis. Chem. Rev..

[30] Vaissier
Welborn V., Head-Gordon T. (2019). Computational Design of Synthetic
Enzymes. Chem. Rev..

[31] Kamerlin S. C. L., Sharma P. K., Prasad R. B., Warshel A. (2013). Why Nature Really Chose
Phosphate. Q. Rev. Biophys..

[32] Léonard N. G., Dhaoui R., Chantarojsiri T., Yang J. Y. (2021). Electric Fields
in Catalysis: From Enzymes to Molecular Catalysts. ACS Catal..

[33] Kast P., Asif-Ullah M., Jiang N., Hilvert D. (1996). Exploring
the Active
Site of Chorismate Mutase by Combinatorial Mutagenesis and Selection:
The Importance of Electrostatic Catalysis. Proc.
Natl. Acad. Sci. U. S. A..

[34] Fried S. D., Boxer S. G. (2017). Electric Fields and Enzyme Catalysis. Annu. Rev. Biochem..

[35] Piejko M., Alfonso-Ramos J. E., Moran J., Stuyver T. (2025). Abiotic Ribonucleoside
Formation in Aqueous Microdroplets: Mechanistic Exploration, Acidity,
and Electric Field Effects. ChemistryEurope.

[36] Meir R., Chen H., Lai W., Shaik S. (2010). Oriented Electric Fields
Accelerate Diels-Alder Reactions and Control the Endo/Exo Selectivity. ChemPhysChem.

[37] Gopakumar K., Shaik S., Ramanan R. (2023). Two-Way Catalysis in a Diels-Alder
Reaction Limits Inhibition Induced by an External Electric Field. Angew. Chem., Int. Ed..

[38] Swain L., Paul E., Gopakumar K., Ramanan R. (2025). Electric Fields Interrupt
with Lewis Acidity and Reverse Back to Lewis Base Function. Chem.—Asian J..

[39] Yu S., Vermeeren P., Hamlin T. A., Bickelhaupt F. M. (2021). How Oriented
External Electric Fields Modulate Reactivity. Chem. - Eur. J..

[40] Wang Z., Danovich D., Ramanan R., Shaik S. (2018). Oriented-External Electric
Fields Create Absolute Enantioselectivity in Diels-Alder Reactions:
Importance of the Molecular Dipole Moment. J.
Am. Chem. Soc..

[41] Shaik S., Danovich D., Kalita S., Dubey K. D. (2025). Oriented Electric
Fields—Universal Catalysts. Acc. Chem.
Res..

[42] Guo S.-Y., Paraja M., Jozeliu̅naitė A., Gallardo-Villagrán M., Zhang Q.-X., Marsalek A., Sakai N., Matile S. (2025). Organocatalytic
Microfluidic Double-Layer Capacitors. Angew.
Chem., Int. Ed..

[43] Paraja M., Matile S. (2026). Ion-Pair Breakers and Anionic Brønsted Acids for
Helmholtz-Layer Catalysis with External Electric Fields in Microfluidic
Capacitors. JACS Au.

[44] Guo S.-Y., Matile S. (2026). Catalysis of Aldol
Reactions with External Electric
Fields in Microfluidic Capacitors: Formation of Organocatalytic Electric
Double Layers with Prolinol Ethers and Nitrophenols. ChemistryEurope.

[45] Koerner M., Rickborn B. (1989). Anthrones as Reactive Dienes in Diels-Alder Reactions. J. Org. Chem..

[46] Shen J., Nguyen T. T., Goh Y.-P., Ye W., Fu X., Xu J., Tan C.-H. (2006). Chiral Bicyclic Guanidine-Catalyzed Enantioselective
Reactions of Anthrones. J. Am. Chem. Soc..

[47] Zea A., Valero G., Alba A.-N. R., Moyano A., Rios R. (2010). Bifunctional
Thiourea-Catalyzed Asymmetric Addition of Anthrones to Maleimides. Adv. Synth. Catal..

[48] Akalay D., Dürner G., Göbel M. W. (2008). A First Case of Asymmetric Catalysis
Induced by Metal-Free Bisoxazolines. Eur. J.
Org. Chem..

[49] Tokioka K., Masuda S., Fujii T., Hata Y., Yamamoto Y. (1997). Asymmetric
Cycloaddition of Anthrone with *N*-Substituted Maleimides
with *C*
_2_-Chiral Pyrrolidines. Tetrahedron Asymmetry.

[50] Riant O., Kagan H. B. (1989). Asymmetric Diels-Alder Reaction Catalyzed
by Chiral
Bases. Tetrahedron Lett..

[51] Baik W.-P., Yoon C.-H., Koo S.-H., Kim H.-K., Kim J.-H., Kim J.-R., Hong S.-D. (2004). Lewis Acid-Catalyzed
Reactions of
Anthrone: Preference for Cycloaddition Reaction over Conjugate Addition
Depending on the Functionality of α,β-Unsaturated Carbonyl
Compounds. Bull. Korean Chem. Soc..

[52] Bin P., Ke-Jun C., Da-Wei M. (2000). Chiral Guanidine
Catalyzed Michael
Addition Reaction and Diels-Alder Reaction of Anthrone and *N*-Methylmaleimide. Chin. J. Chem..

[53] Elsherbini M., Wirth T. (2019). Electroorganic Synthesis
under Flow Conditions. Acc. Chem. Res..

[54] Noël T., Cao Y., Laudadio G. (2019). The Fundamentals
Behind the Use of Flow Reactors in
Electrochemistry. Acc. Chem. Res..

[55] Bajada M. A., Sanjose-Orduna J., Di Liberto G., Tosoni S., Pacchioni G., Noel T., Vile G. (2022). Interfacing Single-Atom Catalysis
with Continuous-Flow Organic Electrosynthesis. Chem. Soc. Rev..

[56] Folgueiras-Amador A. A., Philipps K., Guilbaud S., Poelakker J., Wirth T. (2017). An Easy-to-Machine Electrochemical Flow Microreactor: Efficient Synthesis
of Isoindolinone and Flow Functionalization. Angew. Chem., Int. Ed..

[57] Smith B. P., Truax N. J., Pollatos A. S., Meanwell M., Bedekar P., Garrido-Castro A. F., Baran P. S. (2024). Total Synthesis of Dragocins A-C
through Electrochemical Cyclization. Angew.
Chem., Int. Ed..

[58] Helmholtz H. (1853). Ueber einige
Gesetze der Vertheilung elektrischer Ströme in körperlichen
Leitern mit Anwendung auf die thierisch-elektrischen Versuche. Ann. Phys..

[59] Schott C. M., Schneider P. M., Song K.-T., Yu H., Götz R., Haimerl F., Gubanova E., Zhou J., Schmidt T. O., Zhang Q., Alexandrov V., Bandarenka A. S. (2024). How to
Assess and Predict Electrical Double Layer Properties. Implications
for Electrocatalysis. Chem. Rev..

[60] Kilic M. S., Bazant M. Z., Ajdari A. (2007). Steric Effects
in the Dynamics of
Electrolytes at Large Applied Voltages. I. Double-Layer Charging. Phys. Rev. E.

[61] Kornyshev A. A. (2007). Double-Layer
in Ionic Liquids: Paradigm Change?. J. Phys.
Chem. B.

[62] Gebbie M.
A., Liu B., Guo W., Anderson S. R., Johnstone S. G. (2023). Linking
Electric Double Layer Formation to Electrocatalytic Activity. ACS Catal..

[63] Sakai N., Matile S. (2003). Anion-Mediated Transfer of Polyarginine
across Liquid
and Bilayer Membranes. J. Am. Chem. Soc..

[64] Butterfield S. M., Miyatake T., Matile S. (2009). Amplifier-Mediated
Activation of
Cell-Penetrating Peptides with Steroids: Multifunctional Anion Transporters
for Fluorogenic Cholesterol Sensing in Eggs and Blood. Angew. Chem., Int. Ed..

[65] Miyatake T., Nishihara M., Matile S. (2006). A Cost-Effective Method for the Optical
Transduction of Chemical Reactions. Application to Hyaluronidase Inhibitor
Screening with Polyarginine-Counteranion Complexes in Lipid Bilayers. J. Am. Chem. Soc..

[66] Takeuchi T., Matile S. (2009). DNA Aptamers as Analyte-Responsive Cation Transporters
in Fluorogenic Vesicles: Signal Amplification by Supramolecular Polymerization. J. Am. Chem. Soc..

[67] Takeuchi T., Montenegro J., Hennig A., Matile S. (2011). Pattern Generation
with Synthetic Sensing Systems in Lipid Bilayer Membranes. Chem. Sci..

[68] Sakai N., Houdebert D., Matile S. (2003). Voltage-Dependent Formation of Anion
Channels by Synthetic Rigid-Rod Push-Pull β-Barrels. Chem.—Eur. J..

[69] Schott C. M., Schneider P. M., Song K.-T., Yu H., Götz R., Haimerl F., Gubanova E., Zhou J., Schmidt T. O., Zhang Q., Alexandrov V., Bandarenka A. S. (2024). How to
Assess and Predict Electrical Double Layer Properties. Implications
for Electrocatalysis. Chem. Rev..

[70] Hou Y., Aoki K. J., Chen J., Nishiumi T. (2014). Solvent Variables Controlling
Electric Double Layer Capacitance at the Metal-Solution Interface. J. Phys. Chem. C.

[71] Wang X., Liu K., Wu J. (2021). Demystifying the Stern Layer at a Metal-Electrolyte
Interface: Local Dielectric Constant, Specific Ion Adsorption, and
Partial Charge Transfer. J. Chem. Phys..

[72] Rafiee M., Abrams D. J., Cardinale L., Goss Z., Romero-Arenas A., Stahl S. S. (2024). Cyclic Voltammetry
and Chronoamperometry: Mechanistic
Tools for Organic Electrosynthesis. Chem. Soc.
Rev..

[73] Fang J., Ji C., Meng Y., Ye P., Zhang H., Li W. (2025). Electrochemical
Polymerization of 1,2-Dithiolane Derivatives at Room Temperature. Angew. Chem., Int. Ed..

[74] Bos, M. E. ; Hashimoto, T. ; Maruoka, K. ; Smith, M. D. ; Badiola, K. N-Benzylquininium Chloride. In Encyclopedia of Reagents for Organic Synthesis; John Wiley & Sons, Ltd., 2016; pp 1–7.

[75] Fini F., Sgarzani V., Pettersen D., Herrera R. P., Bernardi L., Ricci A. (2005). Phase-Transfer-Catalyzed
Asymmetric Aza-Henry Reaction Using N-Carbamoyl
Imines Generated In Situ from α-Amido Sulfones. Angew. Chem., Int. Ed..

[76] Onsager L. (1934). Deviations
from Ohm’s Law in Weak Electrolytes. J. Chem. Phys..

[77] Maliekal P. J., Gulvi N., Badani P. M. (2022). Role of Non-Covalent Interactions
in Deciding the Fate of Product Formation in Bifunctional Thiourea-Assisted
Chiral Organic Reactions. Theor. Chem. Acc..

[78] Wang Y., Yu T.-Y., Zhang H.-B., Luo Y.-C., Xu P.-F. (2012). Hydrogen-Bond-Mediated
Supramolecular Iminium Ion Catalysis. Angew.
Chem., Int. Ed..

[79] Zhang Z., Schreiner P. R. (2009). (Thio)­Urea Organocatalysis—What Can Be Learnt
from Anion Recognition?. Chem. Soc. Rev..

[80] Sak M. H., Jacobsen E. N. (2025). Selective Noncovalent
Catalysis with Small Molecules. Chem. Rev..

[81] Kotke M., Schreiner P. R. (2006). Acid-Free,
Organocatalytic Acetalization. Tetrahedron.

[82] Terada M., Nakano M., Ube H. (2006). Axially Chiral
Guanidine as Highly
Active and Enantioselective Catalyst for Electrophilic Amination of
Unsymmetrically Substituted 1,3-Dicarbonyl Compounds. J. Am. Chem. Soc..

[83] Akiyama T. (2007). Stronger Brønsted
Acids. Chem. Rev..

[84] Betinol I. O., Kuang Y., Mulley B. P., Reid J. P. (2025). Controlling Stereoselectivity
with Noncovalent Interactions in Chiral Phosphoric Acid Organocatalysis. Chem. Rev..

[85] Luo N., Turberg M., Leutzsch M., Mitschke B., Brunen S., Wakchaure V. N., Nöthling N., Schelwies M., Pelzer R., List B. (2024). The Catalytic Asymmetric Polyene
Cyclization of Homofarnesol to Ambrox. Nature.

[86] Hashimoto T., Maruoka K. (2007). Recent Development
and Application of Chiral Phase-Transfer
Catalysts. Chem. Rev..

