# Supplementary material for: Electric-Field Catalysis of Diels–Alder Reactions
Source: ACS Cent Sci. 2026 Jul 24;12(8):1165–77. doi: 10.1021/acscentsci.6c00852 (PMC13523502; doi:10.1021/acscentsci.6c00852)
Supplement: Supplementary file 1 [file oc6c00852_si_001.pdf]

# Supporting Information

Electric-Field Catalysis of Diels-Alder Reactions

Bing-Ru Shao, Shen-Yi Guo, Miguel Paraja, Qing-Xia Zhang, Augustina Jozeliūnaitė, Naomi Sakai and Stefan Matile*

Department of Organic Chemistry, University of Geneva, 1211 Geneva, Switzerland

National Centre of Competence in Research (NCCR) Molecular Systems Engineering, 4002 Basel, Switzerland

*E-mail: stefan.matile@unige.ch

## Table of Contents


1. Material and Methods S3

2. Synthesis S4

   2.1. Synthesis of Reaction Products S4

   2.2. Synthesis of Helmholtz Components S5

3. Electric-Field Catalysis S11

   3.1. Microfluidic Capacitors S11

   3.2. Helmholtz Component Generation S13

   3.3. General Procedure for EFC of Diels-Alder Reactions S16

   3.4. HPLC Analysis S17

   3.5. NMR Analysis S18

   3.6. Chronoamperometry S96

   3.7. Radical Scavenger Control Experiments S106

4. Supporting References S110

5. NMR Spectra S111

## 1. Material and Methods

As described in reference S1. Reagents for synthesis were purchased from Fluka, Sigma-Aldrich, Alfa Aesar, TCI and Acros. Polyarginine hydrochloride (MW 5000-15000, **27**·Cl) and (*R*)-4,4-dibutyl-2,6-bis(3,4,5-trifluorphenyl)-4,5-dihydro-3H-dinaphtho[7,6,1,2-cde]azepinium bromide (**40**·Br) were purchased from Sigma-Aldrich. SDS was purchased from PanReac AppliChem. (*S*)-VAPOL phosphoric acid (**55**) and (11a*R*)-10,11,12,13-tetrahydro-5-hydroxy-3,7-diphenyl-diindeno[7,1-de:1',7'-fg][1,3,2]dioxaphosphocin-5-oxide (**56**) are commercially available and were purchased from Sigma-Aldrich and BLD Pharm. All chemicals were used as received. Column chromatography was carried out on silica gel 60 (SiliaFlash P60, 40-63 µm). Analytical (TLC) was performed on silica gel 60 (Merck, 0.2 mm). All $^{1}$H, $^{13}$C and $^{19}$F NMR spectra were recorded (as indicated) on a Bruker 300 MHz or 400 MHz spectrometer at room temperature (25 °C) and are reported as chemical shifts (δ) in ppm relative to TMS (δ = 0). Spin multiplicities are reported as a singlet (s), doublet (d), triplet (t) and quartet (q) with coupling constants (*J*) given in Hz, or multiplet (m). Broad peaks are marked as br. $^{1}$H and $^{13}$C resonances were assigned with the aid of additional information from 1D and 2D NMR spectra ($^{1}$H-$^{1}$H NOESY, $^{1}$H-$^{1}$H-COSY, DEPT 135, HSQC and HMBC). ESI-HRMS was measured on Xevo G2-S Tof (Waters). All mass data are reported as mass-per-charge ratio *m/z* (intensity in %, [assignment]). Chronoamperometry (CA) was performed with CHI660C electrochemical workstation using CHI660E software.

**Abbreviations**: BHT: Butylated hydroxytoluene; DBU: 1,8-Diazabicyclo[5.4.0]undecane-7-ene; DMF: N,N-Dimethylformamide; DMSO: Dimethyl sulfoxide; *ee*: Enantiomeric excess; EF: Electric field; EFC: Electric-field catalysis; Fmoc: 9-Fluorenylmethoxycarbonyl; HATU: 2-(7-Aza-1H-benzotriazole-1-yl)-1,1,3,3-tetramethyluronium hexafluorophosphate; I: Current; P: Product; Pbf: 2,2,4,6,7-Pentamethyldihydrobenzofuran-5-sulfonyl; pR: Poly-L-arginine; rt: Room temperature; SM: Starting material; TEA: Triethylamine; TFA: Trifluoroacetic acid.

## 2. Synthesis

### 2.1. Synthesis of Reaction Products

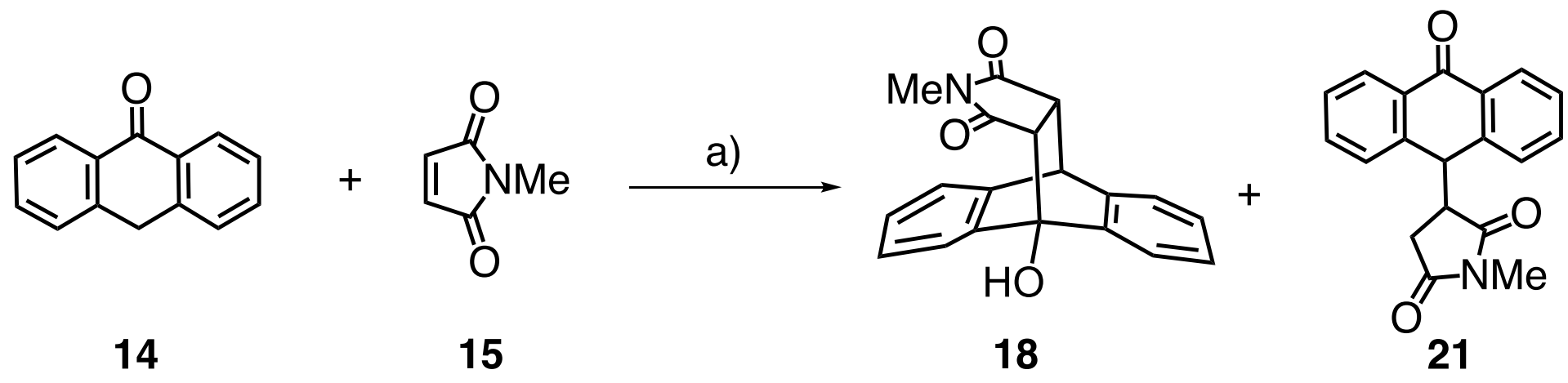


**Scheme S1.** (a) TEA, $CH_2Cl_2$, rt, 12 h, 45% (**18**) + 18% (**21**).

**Compounds 18 and 21** were prepared following the previously reported procedure.[S2]

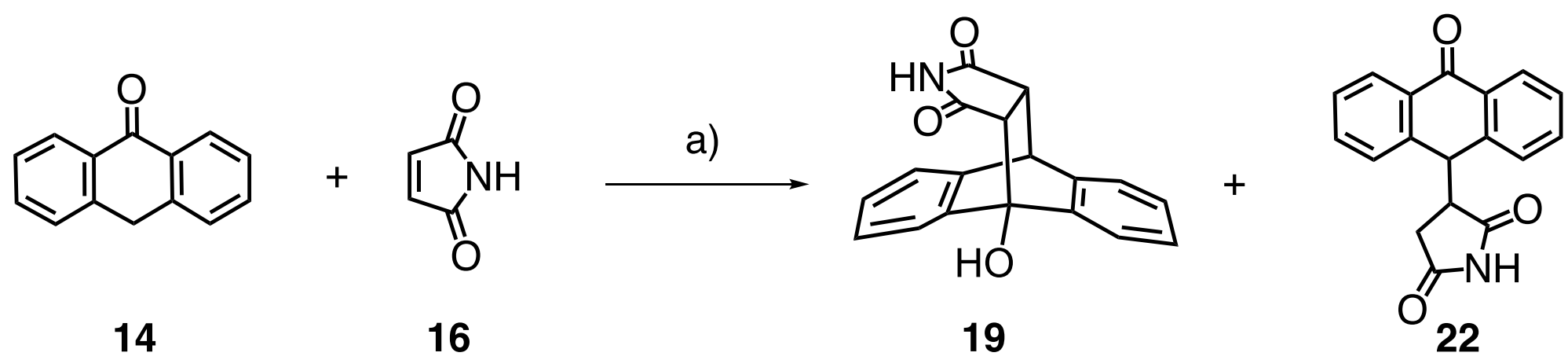


**Scheme S2.** (a) Xylene, 90 °C to rt, 72 h, 40% (**19**).

**Compound 19** was prepared following the previously reported procedure.[S3]

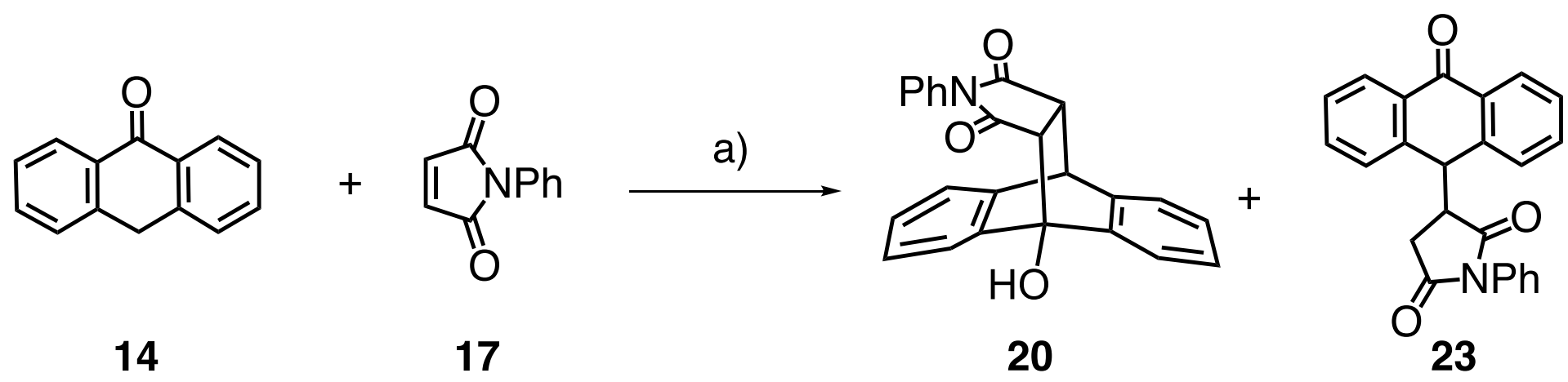


**Scheme S3.** (a) Xylene, 90 °C to rt, 72 h, 33% (**20**).

**Compound 20** was prepared following the previously reported procedure.[S3]

## 2.2. Synthesis of Helmholtz Components

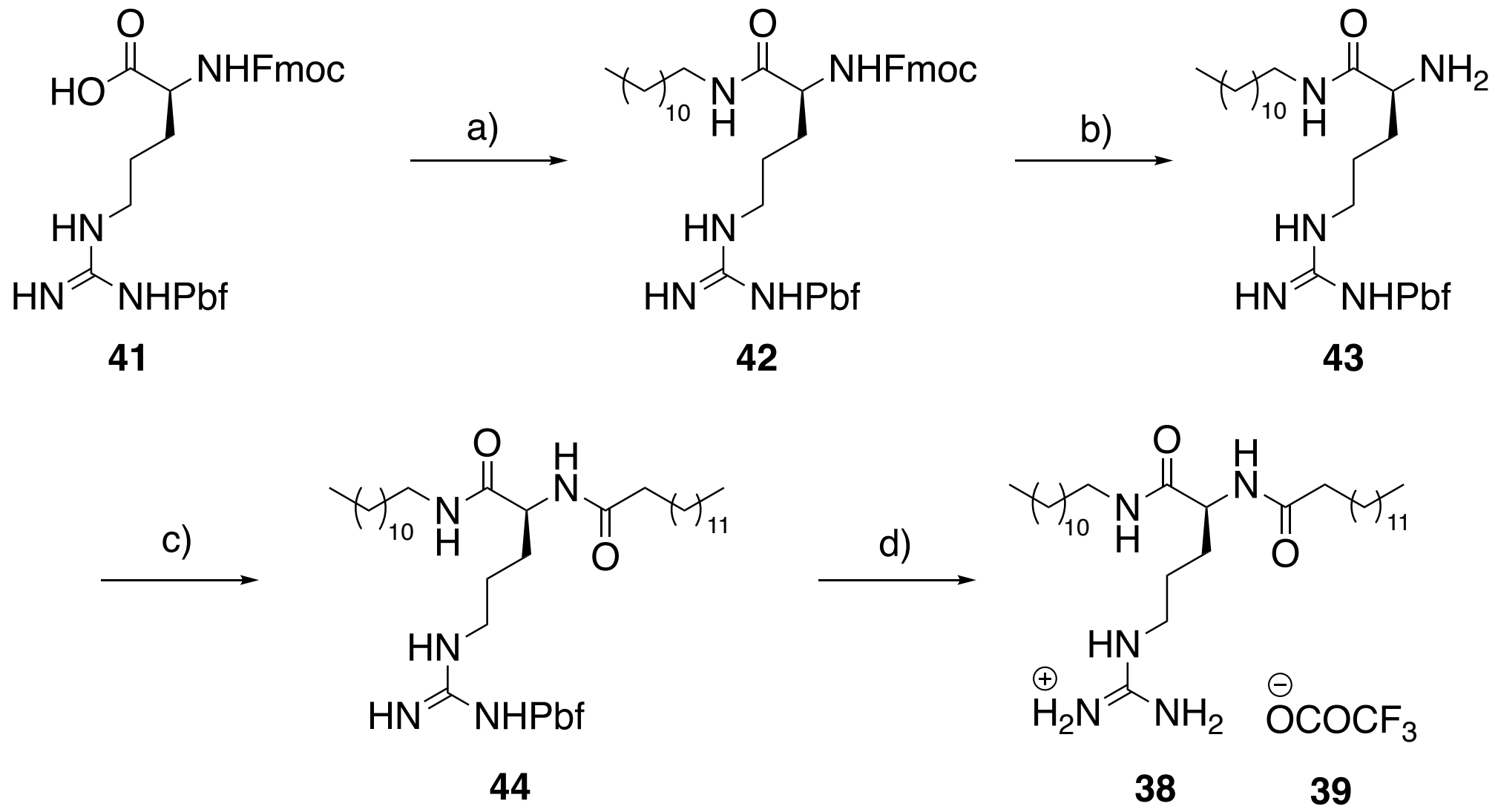

**Scheme S4.** a) Dodecylamine, HATU, TEA, DMF, 0 °C to rt, 4 h, 97%. b) DBU, $CH_2Cl_2$, rt, 3 h, 81%. c) Myristoyl chloride, TEA, $CH_2Cl_2$, rt, 2 h, 48%; d) TFA, $CHCl_3$, rt, 4 h, 56% (**38** and **39**).

**Compound 43** was prepared following the previously reported procedure.[S4]

**Compound 44**. To a solution of **43** (200 mg, 337 µmol) dissolved in dry $CH_2Cl_2$ (10 mL), myristoyl chloride was added (91.5 µL, 337 µmol) and the solution was stirred at rt. After 1.5 h, TEA (70.4 µL, 505 µmol) was added and the solution was stirred for 30 min at rt. The reaction mixture was concentrated under reduced pressure and purified by a flash column chromatography ($CH_2Cl_2$/MeOH 97:3 to 9:1, $R_f$ 0.4 in $CH_2Cl_2$/MeOH 9:1) to give **44** as a pale white solid (130 mg, 48%). The crude product was used for the next step without purification.

**Compounds 38 and 39**. Compound **44** (130 mg, 162 µmol) was dissolved in a solution of TFA/$CHCl_3$ 1:1 (8 mL). The solution was stirred at rt for 4 h and concentrated under reduced pressure followed by trituration in $Et_2O$ (50 mL). The solid was dried in vacuo overnight to give **38** and **39** as a colorless hygroscopic solid (60 mg, 56%). $[\alpha]_D^{20}$ + 0.5 (*c* 1.0, MeOH); Mp: 234-235 °C; IR (neat): 3302 (br, N-H), 3187 (br, N-H), 2955 (w, C-H), 2917 (s, C-H), 2849 (m, C-H), 1652 (m, C=O), 1635 (s, C=O), 1548 (m), 1519 (s, N–H /C-N), 1466 (m, C-H), 1279 (m, C-N), 1234 (m, C-N), 1197 (s, C-F), 1145 (s, C-F), 1048 (m), 1027 (s), 880 (m), 721 (m), 702 (m), 682 (m); $^1$H NMR (400 MHz,

$CD_3OD$): 4.29 (dd, $^3J_{H\text{-}H}$ = 8.3, 5.6 Hz, 1H), 3.24 - 3.10 (m, 4H), 2.27 - 2.22 (m, 2H), 1.81 - 1.56 (m, 6H), 1.51-1.46 (m, 2H), 1.29 (br, s, 36H), 0.89 (t, $^3J_{H\text{-}H}$ = 6.6 Hz, 6H); $^{13}$C NMR (101 MHz, $CD_3OD$): 175.0 (C), 172.5 (C), 157.2 (C), 52.9 (CH), 40.5 ($CH_2$), 39.0 ($CH_2$), 35.4 ($CH_2$), 31.6 ($CH_2$), 29.4 ($CH_2$), 29.4 ($CH_2$), 29.4 ($CH_2$), 29.3 ($CH_2$), 29.2, ($CH_2$), 29.1 ($CH_2$), 29.1 ($CH_2$), 29.0 ($CH_2$), 28.9 ($CH_2$), 28.8 ($CH_2$), 26.6 ($CH_2$), 25.6 ($CH_2$), 24.9 ($CH_2$), 22.3 ($CH_2$), 13.1 ($CH_3$); $^{19}$F NMR (282 MHz, $CD_3OD$): -77.27; HRMS (ESI, $[M]^+$) calcd for $C_{32}H_{66}N_5O_2$: 552.5212, found: 552.5199.

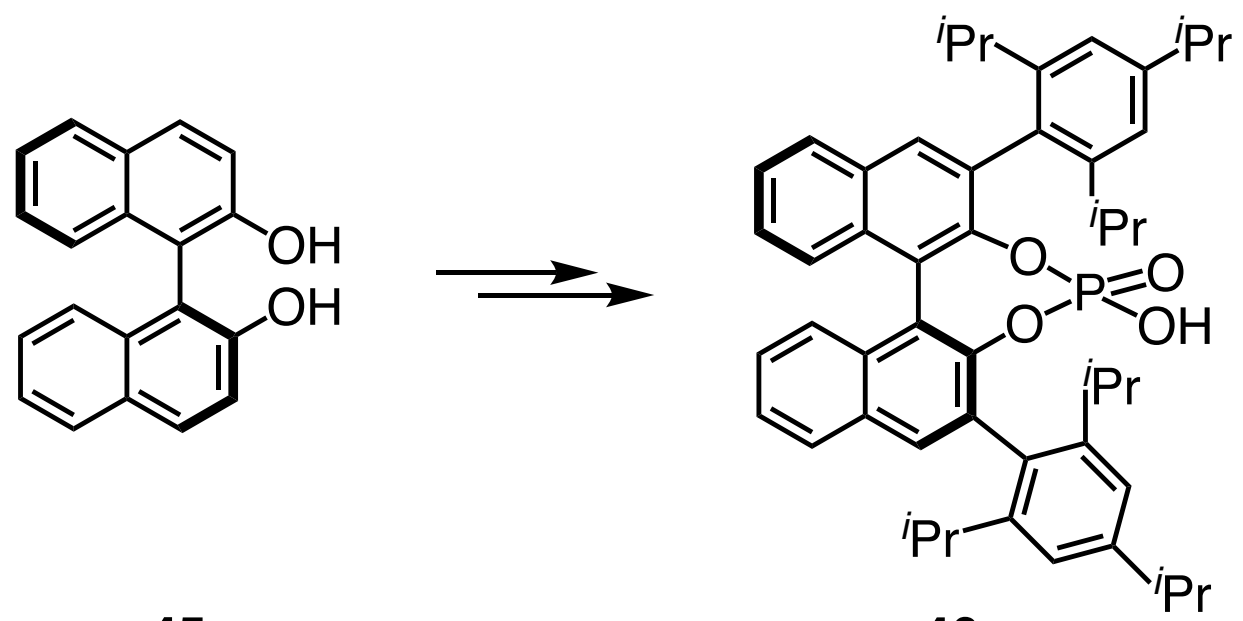


**45** **46**

**Scheme S5.** Synthesis of **46**.[S5]

**Compound 46** was prepared following the previously reported procedure.[S5]

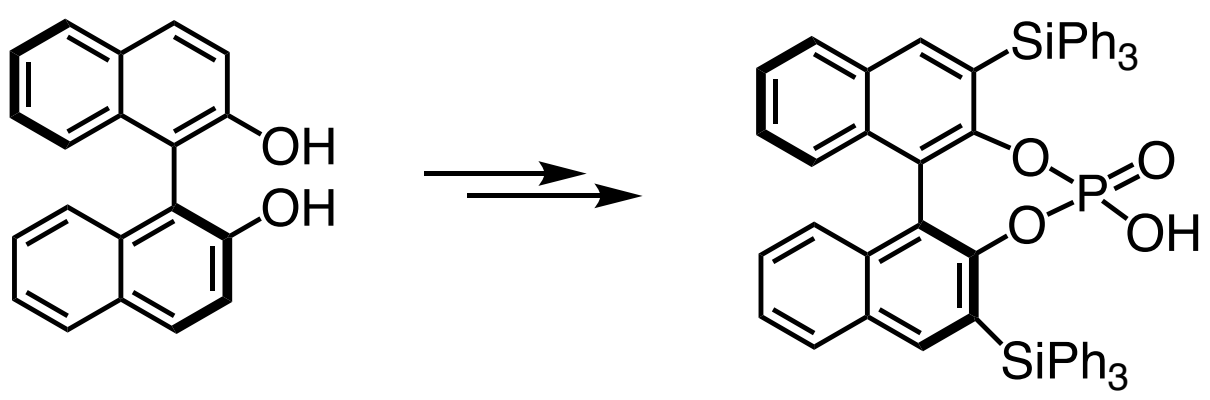


**45** **47**

**Scheme S6.** Synthesis of **47**.[S6]

**Compound 47** was prepared following the previously reported procedure.[S6]

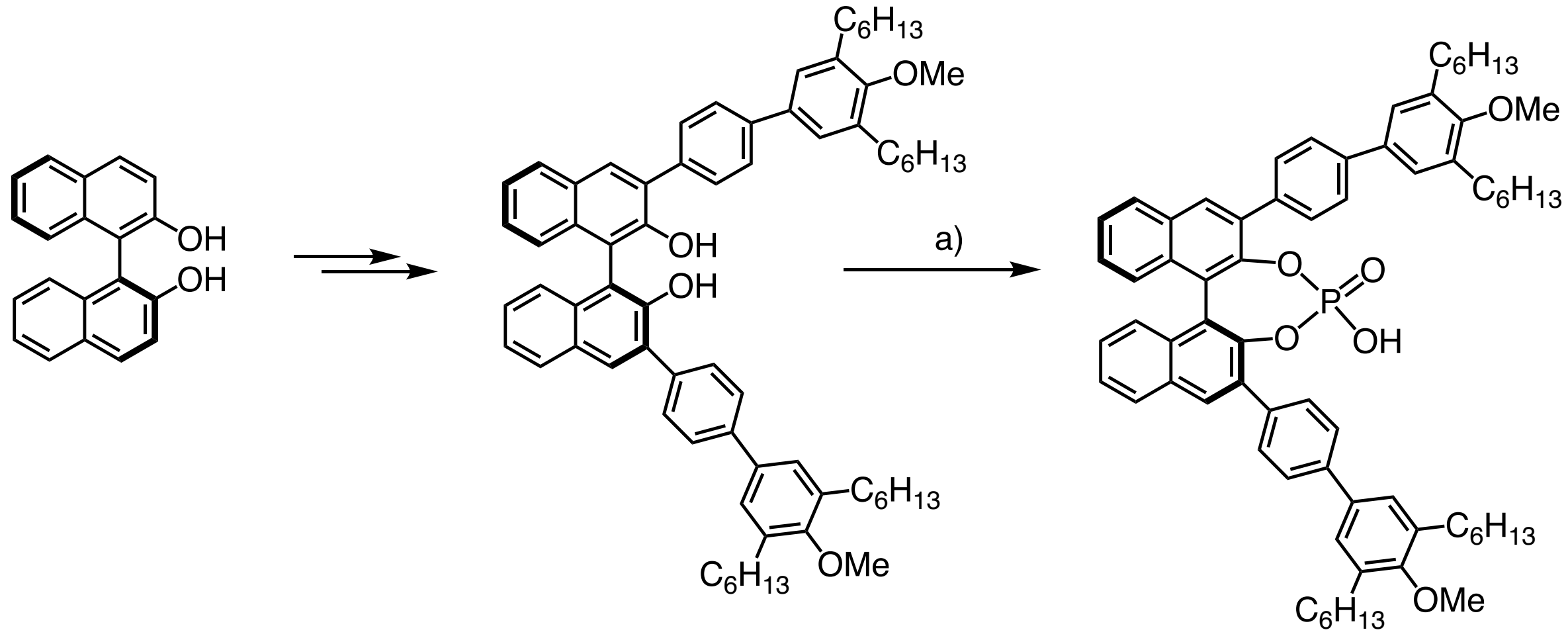

**45** **48** **49**

**Scheme S7.** (a) 1. $POCl_3$, pyridine, 95 °C, 4 h, 2. $H_2O$, 95 °C, 2 h, 87%.

**Compound 48** was prepared following the previously reported procedure.[S7]

**Compound 49.** Under inert atmosphere, $POCl_3$ (160 µL, 1.67 mmol) was added to a solution of **48** (550 mg, 0.56 mmol) in anhydrous pyridine (6.0 mL) and the reaction mixture was heated at 95 °C for 4 h. After cooling down to rt, $H_2O$ (750 µL) was added, and the mixture was heated at 95 °C for 2 h. After cooling down to rt, the reaction mixture was diluted with $CH_2Cl_2$ and washed with 1 M HCl aqueous solution. Aqueous solution was extracted with $CH_2Cl_2$, and the combined organic phase was dried over anhydrous $Na_2SO_4$, filtered and evaporated to dryness. The crude product was diluted with a minimal amount of EtOH, precipitated with 6 M HCl, filtered, washed with 6 M HCl, water, and a minimal amount of cold MeOH, and dried to afford **49** as a yellowish solid (510 mg, 87%). $[\alpha]_D^{20}$ = + 22 (*c* 1.0, $CH_2Cl_2$); Mp: 108-109 °C; IR (neat): 2953 (m, C - H), 2925 (s, C-H), 2855 (m, C - H), 1596 (w, C=C), 1518 (w, C=C), 1497 (m, C=C), 1471 (s, C=C/$CH_2$ bending), 1233 (s, P=O/P-O), 1194 (s, P-O-Ar), 1180 (s, P-O-Ar), 1151 (s, P-O-Ar), 1133 (m, P-O-Ar), 1015 (s, P-O-C), 997 (s, P-O-C), 962 (s), 888 (s), 841 (s), 831 (s), 749 (s, Ar-H), 724 (m, Ar-H), 708 (m, Ar-H), 653 (m, Ar-H); $^1$H NMR (500 MHz, $CDCl_3$): 8.05 (s, 2H), 7.96 (d, $^3J_{H-H}$ = 8.32 Hz, 2H), 7.65 (d, $^3J_{H-H}$ = 8.3 Hz, 4H), 7.54 - 7.43 (m, 6H), 7.39 - 7.27 (m, 4H), 7.15 (s, 4H), 3.66 (s, 6H), 2.50 (t, $^3J_{H-H}$ = 8.0 Hz, 8H), 1.49 (p, $^3J_{H-H}$ = 7.3 Hz, 8H), 1.30 - 1.09 (m, 24H), 0.88 - 0.73 (m, 12H); $^{13}$C NMR (126 MHz,

CDCl3) 156.0 (C), 144.6 (C), 144.6 (C), 140.6 (C), 136.3 (C), 135.8 (C), 135.2 (C), 133.6 (C), 133.6 (C), 131.9 (C), 131.7 (C), 131.3 (CH), 130.1(CH), 128.5 (CH), 127.1(CH), 126.7 (CH), 126.6 (CH), 126.5 (CH), 125.9 (C), 122.6 (C), 122.5 (C), 61.2 ($OCH_3$), 31.6 ($CH_2$), 30.9 ($CH_2$), 30.1 ($CH_2$), 29.5 ($CH_2$), 22.6 ($CH_2$), 14.1 ($CH_3$); $^{31}$P NMR (162 MHz, $CDCl_3$): 2.50; HRMS (ESI, [M-H]$^-$) calcd for $C_{70}H_{81}O_6P$: 1047.5697, found: 1047.5690.

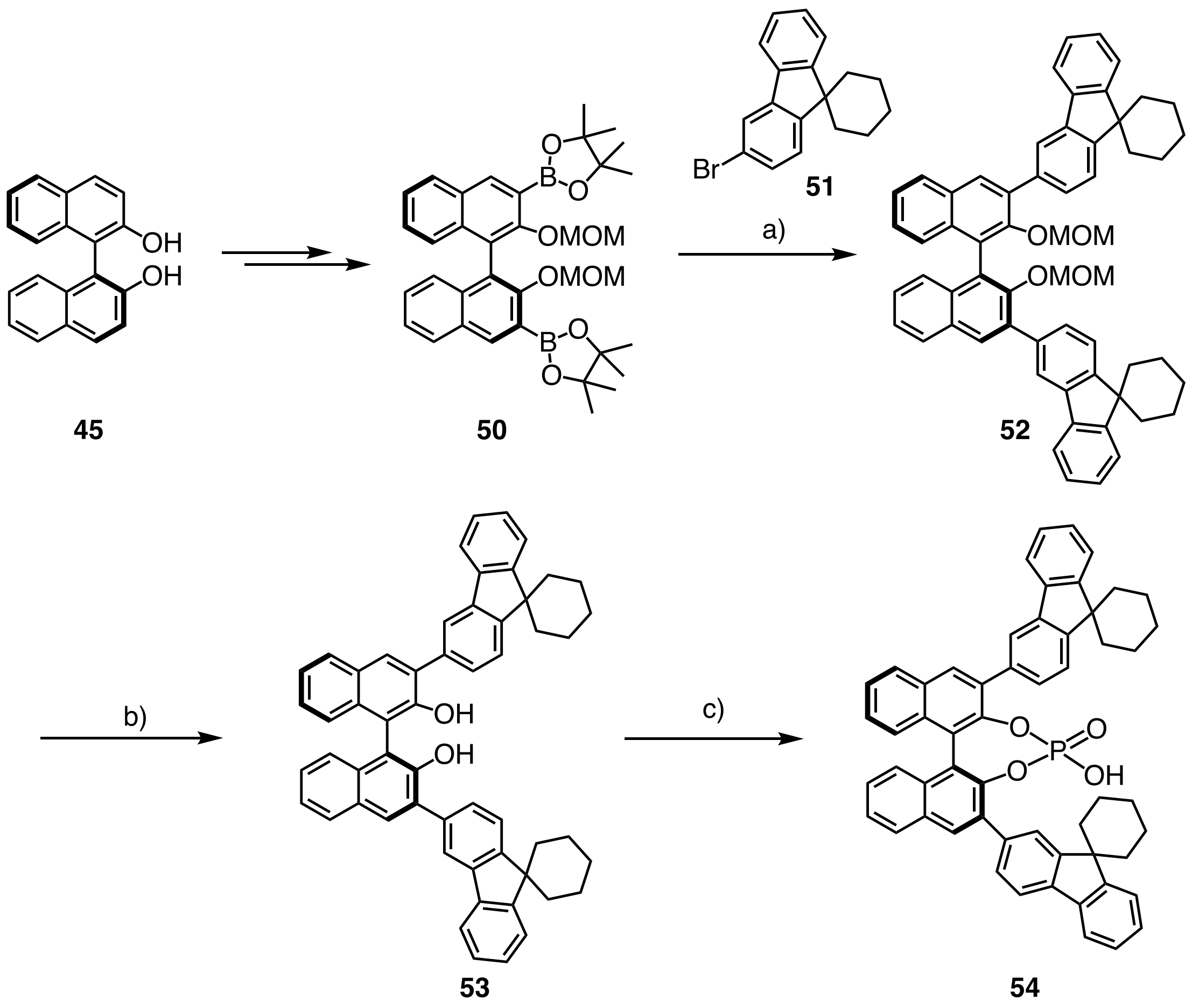


**Scheme S8.** (a) $K_2CO_3$, $Pd(PPh_3)_4$, toluene/EtOH/$H_2O$ 3:2:1, 110 °C, 24 h, 43%. (b) HCl, 1,4-dioxane, 60 °C, 24 h, 93%. (c) 1. $POCl_3$, pyridine, 95 °C, 4 h, 2. $H_2O$, 95 °C, 2 h, 66%.

**Compound 50** was prepared following the previously reported procedure.[S8]

**Compound 52.** Under inert atmosphere a mixture of **50** (2.68 g, 4.28 mmol), **51** (3.35 g, 10.7 mmol) and $K_2CO_3$ (2.96 g, 21.4 mmol) was dissolved in anhydrous toluene/EtOH/$H_2O$ 3:2:1 (54 mL) and the solution was sparged with argon for 30 min. Then $Pd(PPh_3)_4$ (494 mg, 0.04 mmol) and the reaction mixture was stirred at 110 °C for 24 h. After cooling down to rt, the mixture was diluted with

water and extracted with ethyl acetate. Combined organic phase was washed with water and brine, dried over anhydrous $Na_2SO_4$ and evaporated to dryness. The crude product was purified by column chromatography on silica gel (Pen/EtOAc 20:1, $R_f = 0.3$) to afford **52** as an amorphous solid (1.53 g, 43 %);$^1$H NMR (400 MHz, $CDCl_3$): 8.07 - 8.00 (m, 4H), 7.94 (d, $^3J_{H-H} = 8.2$ Hz, 2H), 7.86 - 7.75 (m, 6H), 7.72 (d, $^3J_{H-H} = 7.1$ Hz, 2H), 7.44 (ddd, $^3J_{H-H} = 8.1, 6.4, 1.7$ Hz, 2H), 7.39 (td, $^3J_{H-H} = 7.4, 1.2$ Hz, 2H), 7.36 - 7.27 (m, 6H), 4.51 - 4.43 (m, 4H), 2.40 (s, 6H), 2.09 - 1.70 (m, 20H); $^{13}$C NMR (101 MHz, $CDCl_3$): 153.6 (C), 153.5 (C), 151.7 (C), 139.6 (C), 138.8 (C), 137.9 (C), 136.2 (C), 133.8 (C), 131.1 (C), 130.6 (CH), 128.7 ($CH_2$), 128.0 (CH), 127.1 (CH), 126.9 (CH), 126.7 (C), 126.6 (CH), 126.4 (CH), 125.8 (CH), 125.3 (CH), 124.9 (CH), 120.1 (CH), 119.7 (CH), 98.7($CH_2$), 56.1 ($CH_3$), 50.6 (C), 35.9 (d, $J_{C-C} = 5.9$ Hz), 25.7 ($CH_2$), 22.8 (d, $J_{C-C} = 4.0$ Hz).

**Compound 53.** To a solution of **52** (1.53 g, 1.82 mmol) in 1,4-dioxane (35 mL) was added conc. HCl (3.65 mL, 24 equiv), and the reaction mixture was warmed to 60 °C and stirred for 2 days. After cooling to rt, the reaction mixture was diluted with $CH_2Cl_2$ and washed with saturated aqueous $NaHCO_3$. The aqueous phase was extracted with $CH_2Cl_2$, and the combined organic layer was washed with $NaHCO_3$ and brine, dried with $Na_2SO_4$, filtered and concentrated under reduced pressure. The crude was purified by column chromatography on silica gel (Pen/EtOAc 10:1, $R_f = 0.4$) to afford **53** as a yellow solid (1.28 g, 93%);$^1$H NMR (400 MHz, $CDCl_3$): 8.11 (s, 2H), 8.04 (d, $^3J_{H-H} = 1.7$ Hz, 2H), 7.96 (s, 2H), 7.86 (d, $^3J_{H-H} = 7.8$ Hz, 2H), 7.83 - 7.78 (m, 2H), 7.74 (dd, $J = 7.8, 1.6$ Hz, 2H), 7.70 (d, $J = 6.8$ Hz, 2H), 7.44 - 7.27 (m, 10H), 5.50 (s, 2H), 2.13 - 1.67 (m, 20H); $^{13}$C NMR (101 MHz, $CDCl_3$): 153.6 (d, $J_{C-C} = 3.0$ Hz), 150.48 (C), 139.5 (d, $J_{C-C} = 5.9$ Hz), 136.0 (C), 133.1 (C), 131.5 (CH), 131.3 (C), 129.7 (C), 128.6 (d, $J = 3.5$ Hz), 127.4 (CH), 127.1 (d, $J = 2.0$ Hz), 125.9 (CH), 124.7 (CH), 124.53 (d, $J_{C-C} = 5.4$ Hz), 120.1 (CH), 119.9 (CH), 112.8 (C), 50.6 (C), 35.8 (d, $J_{C-C} = 3.0$ Hz), 25.7 ($CH_2$), 22.9 ($CH_2$).

**Compound 54.** Under inert atmosphere to a solution of **53** (1.08 g, 1.44 mmol) in anhydrous pyridine (11.5 mL) was added $POCl_3$ (0.40 mL, 4.3 mmol) and reaction mixture was heated at 95 °C

for 4 hours. After cooling down to rt, $H_2O$ (1.5 mL) was added and heated at 95 ºC for another 2 hours. After cooling down to rt, the reaction mixture was diluted with $CH_2Cl_2$ and washed with 1 M HCl aqueous solution. The aqueous solution was extracted with $CH_2Cl_2$, the combined organic phase was dried over anhydrous $Na_2SO_4$, filtered and evaporated to dryness. The crude was diluted with minimal amount of THF and precipitated with 6 M HCl, filtered, washed with 6 M HCl, water, minimal amount of cold MeOH and dried to afford **54** as a yellowish solid (770 mg, 66%). $[\alpha]_D^{20}$ = + 20.6 (*c* 1.0, $CH_2Cl_2$); Mp: 283-284 °C; IR (neat): 3053 (w, Ar-H), 2927 (w, C-H), 2845 (w, C-H), 1598 (w, C=C), 1498 (m, C=C), 1467 (m, C=C), 1448 (m, C=C), 1263 (s, P=O), 1243 (s, P-O/P=O), 1194 (s, P-O-Ar), 1149 (s, P-O-Ar), 1129 (m, P-O-Ar), 1017 (s, P-O-C), 979 (m), 966 (m), 887 (m), 831 (m), 747 (s, Ar-H), 734 (s, Ar-H), 699 (m, Ar-H), 678 (m, Ar-H), 664 (m, Ar-H); $^1$H NMR (400 MHz, $CDCl_3$): 8.03 (s, 2H), 7.98 (d, $^3J_{H-H}$ = 8.2 Hz, 2H), 7.83 (d, $^4J_{H-H}$ = 1.5 Hz, 2H), 7.58 - 7.51 (m, 4H), 7.49 - 7.44 (m, 4H), 7.37 (t, $^3J_{H-H}$ = 6.5 Hz, 2H), 7.34 - 7.28 (m, 3H), 7.17 - 7.07 (m, 4H), 6.23 (s, 2H), 1.79 – 1.43 (m, 20H); $^{13}$C NMR (101 MHz, $CDCl_3$): 153.4 (C), 153.2 (C), 144.5 (d, $J_{C-P}$ = 9.2 Hz), 139.2 (d, $J_{C-P}$ = 7.3 Hz), 135.1 (C), 134.6 (d, $J_{C-P}$ = 3.2 Hz), 131.8 (C), 131.6 (C), 131.1 (C), 128.8 (CH), 128.3 (CH), 127.3 (C), 126.7 (CH), 126.4 (CH), 126.3 (CH), 125.9 (CH), 125.8 (CH), 124.1 (CH), 122.4 (d, $J_{C-P}$ = 2.4 Hz), 119.8 (CH), 119.5 (CH), 50.3 (C), 35.6 ($CH_2$), 35.2 ($CH_2$), 25.5 ($CH_2$), 22.6 (d, $J_{C-P}$ = 6.2 Hz); $^{31}$P NMR (162 MHz, $CDCl_3$): 3.58; HRMS (ESI, $[M-H]^-$) calcd for $C_{56}H_{45}O_4P$: 811.2982, found: 811.2987.

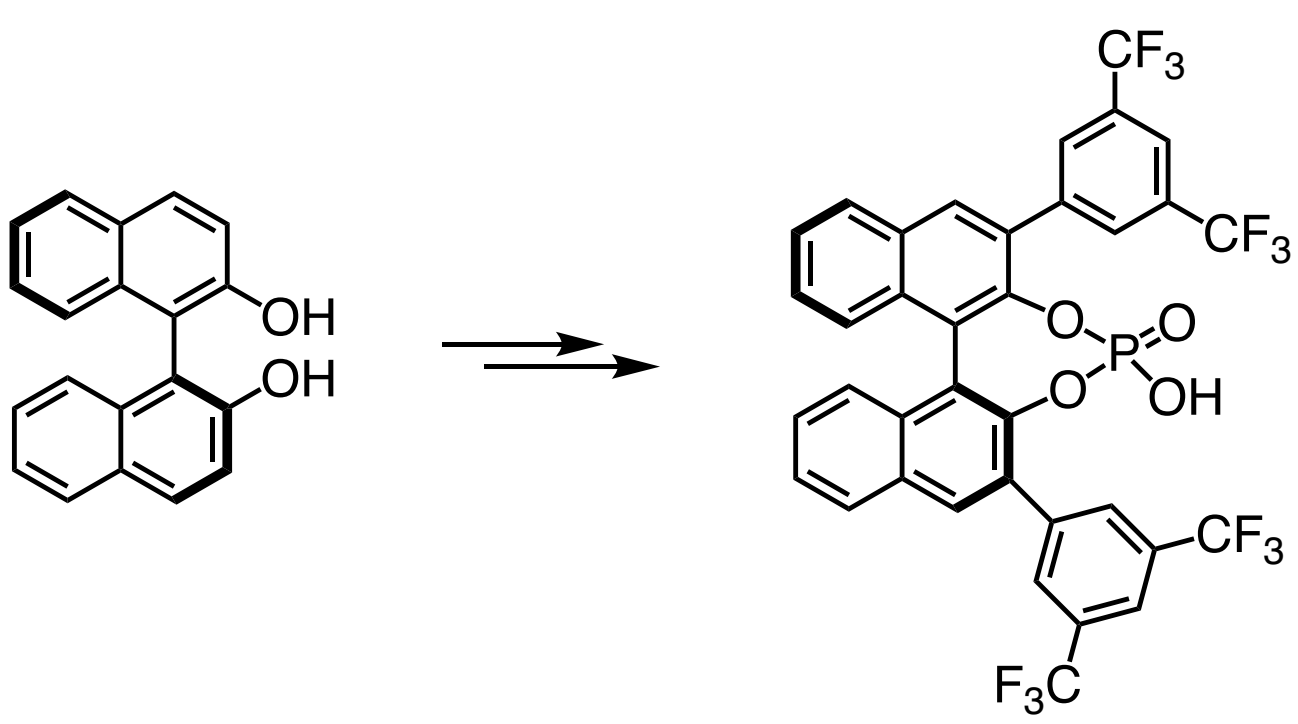


**45** **55**

**Scheme S9.** Synthesis of **55**.[S9]

**Compound 55** was prepared following the previously reported procedure.[S9]

## 3. Electric-Field Catalysis

### 3.1. Microfluidic Capacitors

Electric-field catalysis studies were performed using a setup similar to those described in reference S1 using a stand-alone Vapourtec Ion Electrochemical Reactor, with an Aim-TTi EX354RD Dual Power Supply from Thurlbym Thandar Instruments Ltd. Chemyx Fusion 100 Touch Syringe Pumps were used in the flow set-ups. The electrodes were fabricated from rigid graphite foil (Gr, Goodfellow, 99.95%, 1.0 mm thickness). The electrodes (5 x 5 $cm^2$) were separated by a 0.10 mm FEP spacer, resulting in a reactor volume of 0.12 mL, with an exposed electrode surface area of 12 $cm^2$.

The graphite plates were polished using a Presi Le Cube polishing machine, in sequential steps: 5 minutes with abrasive paper coated with 6 µm and diamond suspension, followed by 3 minutes with 3 µm, 3 minutes with 1 µm, and finally 5 minutes with 1/4 µm particles.

After every reaction was completed, the system was flushed with chloroform (5.0 mL) and DMSO (5 mL) at a flow rate of 0.4 mL/min. The electrodes and FEP spacer were then cleaned with acetone and ethyl acetate. The graphite electrodes were further immersed in chloroform overnight, followed by 30 minutes of sonication. Before the re-use, the electrodes were repolished to restore surface quality.

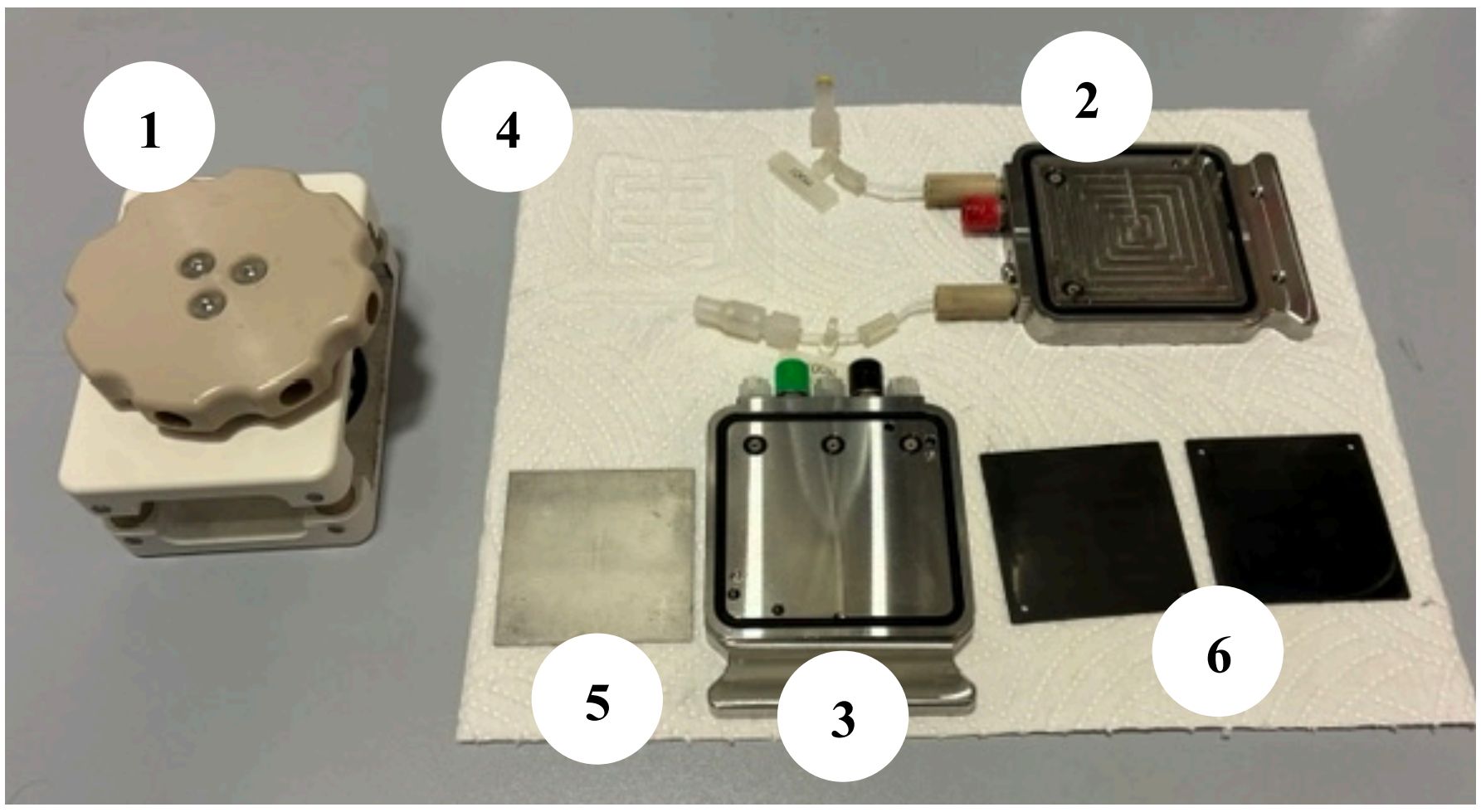


**Figure S1.** Disassembled microfluidic capacitor. 1. External housing. 2. Metallic bottom electrode holder. 3. Metallic top electrode holder. 4. 0.10 mm FEP spacer. 5. Metallic spacer. 6. Graphite electrodes. From reference S1.

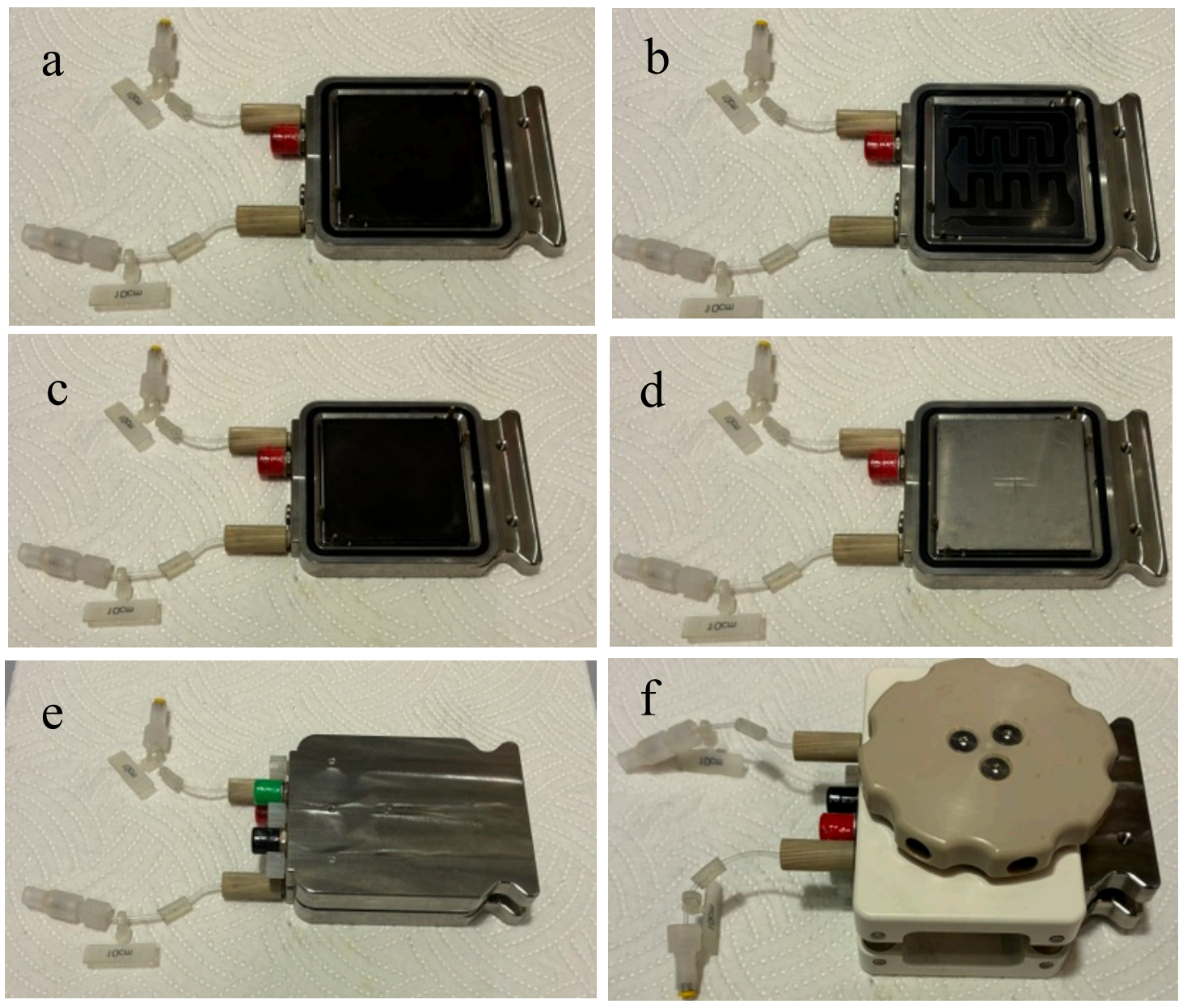


**Figure S2.** Assembly of the microfluidic capacitor. a) One of the graphite electrodes is placed on the metallic bottom electrode holder. b) The 0.10 mm FEP spacer is fitted on top of the graphite electrode. c) The second graphite electrode is placed on the FEP spacer. d) The metallic spacer is placed on top of the second graphite electrode. e) The metallic top electrode holder is fitted with the bottom part. f) The metallic unit is placed inside the housing, and pressure is applied until the capacitor is sealed. From referece S1.

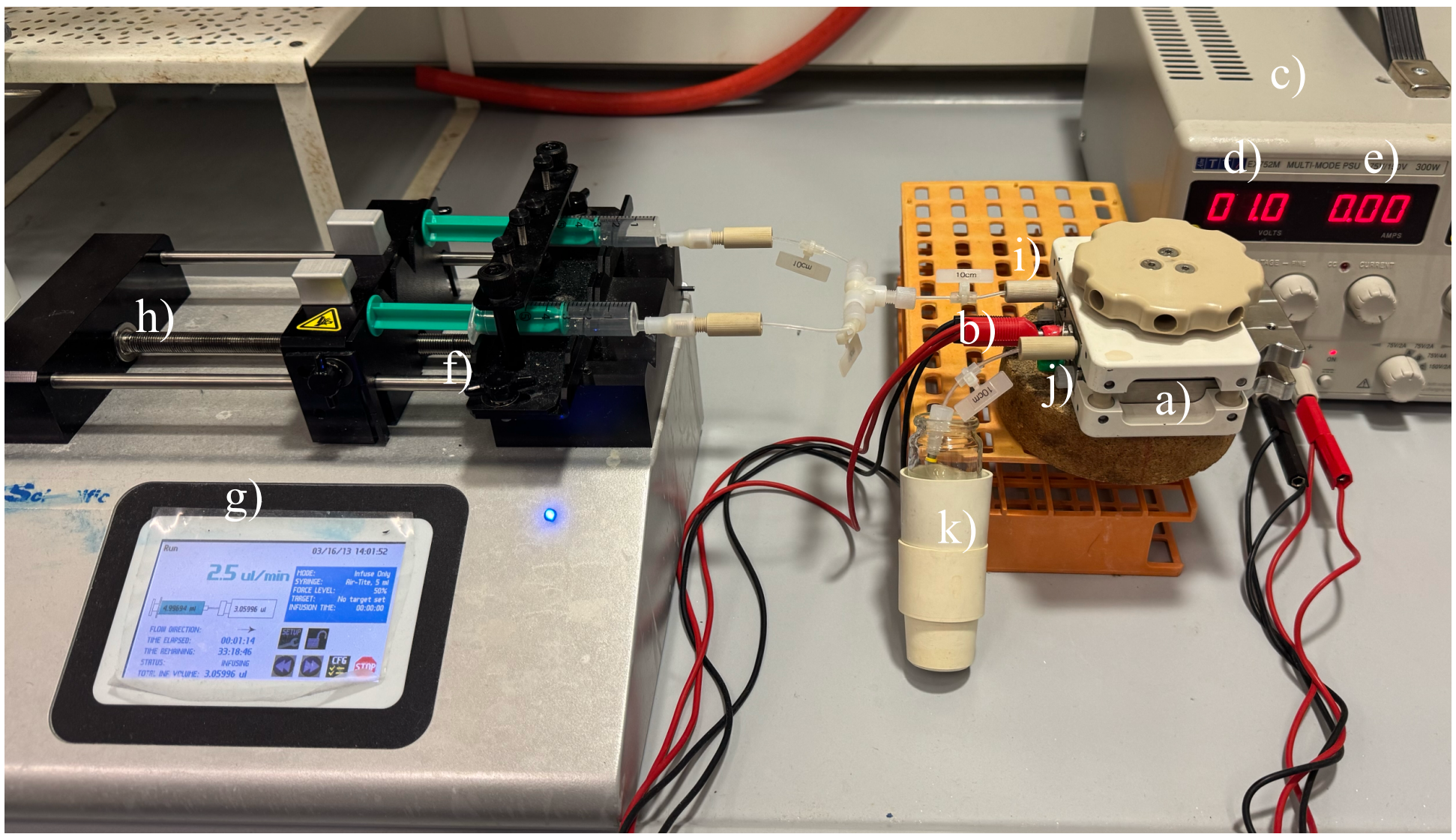


**Figure S3.** The microfluidic capacitor at work in its simplest possible configuration. Once assemFbled, the (a) microfluidic capacitor is (b) connected to (c) a power supply, where (d) the voltage is set and (e) the current is recorded. The (f) reaction mixture of interest in the solvent of interest is injected at (g) a set flowrate using (h) a syringe pump, into (i) the microfluidic capacitor. At (j), the solution exits the microfluidic capacitor, where a (k) vial is placed to collect the reaction mixture for analysis. The reaction solution is pumped through the capacitor at a fixed speed under applied constant voltage. The example shows one of the reactions at $V$ = 1 V reported in Figure S6 (d), with $I$ = 0.00 A (e) measured at steady state during the reaction.

### 3.2. Helmholtz Component Generation

**Components 25 and 26**: Polyarginine **25**·Cl (1.9 mg, 10 µmol monomer) was suspended in DMSO (0.9 mL), dissolved upon sonication for 10 min, and mixed with a solution of SDS (100 mM, 0.10 mL) in DMSO. The obtained solution (0.4 mL) was diluted to 4 mL with $CHCl_3$/DMSO 3.0:0.6 to give a solution of **25/26** (1 mM each) in $CHCl_3$/DMSO 3:1.

**Components 25 and 26 (General procedure):** CPA sodium salt **29** was prepared by neutralizing **46** (75 mg, 0.10 mmol) in MeOH (2.0 mL) with 1 M NaOH (0.10 mL), followed by dilution (with 5 mL water) and lyophilization. The obtained sodium salt **29** (3.0 mg, 4.0 µmol) was

dissolved in $CHCl_3$/DMSO 3:1 (4 mL), and polyarginine **25**·Cl (9.6 mg, 50 μmol arginine) was dissolved in DMSO (5.0 mL). Obtained stock solutions (10 mM, 0.4 mL each) were then mixed and diluted to 4 mL with $CHCl_3$/DMSO 3:0.6 (4 mL). The suspension was sonicated for 10 min. The final concentrations of each component were as follows: 1.0 mM **25/29** (based on monomer unit concentration).

**Components 25 and 31** were prepared following the general procedure. Namely, **56** was neutralized with equimolar NaOH to give **31**. The sodium salt **31** (2.0 mg, 4.0 μmol) was dissolved in $CHCl_3$/DMSO 3:1 (4 mL), and an aliquot (0.40 mL) was mixed with a solution of **25**·Cl (10 mM, 0.40 mL) in DMSO, diluted with $CHCl_3$/DMSO 3:0.6, and sonicated to give a solution of 1.0 mM **25/31** (based on monomer unit concentration) in $CHCl_3$/DMSO 3:1.

**Components 25 and 32** were prepared following the general procedure. Namely, **47** was neutralized with equimolar NaOH to give **32**. The sodium salt **32** (3.5 mg, 4.0 μmol) was prepared in $CHCl_3$/DMSO 3:1 (4 mL), and an aliquot (0.4 mL) was mixed with a solution of **25**·Cl (0.4 mL of 10 mM) in DMSO, diluted with $CHCl_3$/DMSO 3:0.6, and sonicated to give a solution of 1.0 mM **25/32** (based on monomer unit concentration) in $CHCl_3$/DMSO 3:1.

**Components 25 and 33** were prepared following the general procedure. Namely, **49** was neutralized with equimolar NaOH to give **33**. The sodium salt **33** (4.3 mg, 4.0 μmol) was prepared in $CHCl_3$/DMSO 3:1, (4 mL), and an aliquot (0.4 mL) was mixed with a solution of **25**·Cl (10 mM, 0.4 mL) in DMSO, diluted with $CHCl_3$/DMSO 3:0.6, and sonicated to give a solution of 1.0 mM **25/33** (based on monomer unit concentration) in $CHCl_3$/DMSO 3:1.

**Components 25 and 34** were prepared following the general procedure. Namely, **54** was neutralized with equimolar NaOH to give **34**. The sodium salt **34** (3.3 mg, 4.0 μmol) was prepared in $CHCl_3$/DMSO 3:1 (4 mL), and an aliquot (0.4 mL) was mixed with a solution of **25**·Cl (10 mM, 0.4 mL) in DMSO, diluted with $CHCl_3$/DMSO 3:0.6, and sonicated to give a solution of 1.0 mM **25/34** (based on monomer unit concentration) in $CHCl_3$/DMSO 3:1.

**Components 25 and 35** were prepared following the general procedure. Namely, **55** was neutralized with equimolar NaOH to give **35**. The sodium salt **35** (3.2 mg, 4.0 μmol) was prepared in $CHCl_3$/DMSO 3:1 (4 mL), and an aliquot (0.4 mL) was mixed with a solution of **25**·Cl (10 mM, 0.4 mL) in DMSO, diluted with $CHCl_3$/DMSO 3:0.6, and sonicated to give a solution of 1.0 mM **25/35** (based on monomer unit concentration) in $CHCl_3$/DMSO 3:1.

**Components 30 and 36** were prepared following the general procedure. Namely, **55** was neutralized with equimolar NaOH to give **30**. The sodium salt **30** (2.5 mg, 4.0 μmol) was prepared in $CHCl_3$/DMSO 3:1 (4 mL), and an aliquot (0.4 mL) was mixed with a solution of **36**·Cl (0.4 mL of 10 mM) in DMSO, diluted with $CHCl_3$/DMSO 3:0.6, and sonicated to give a solution of 1.0 mM **36/30** (based on monomer unit concentration) in $CHCl_3$/DMSO 3:1.

**Components 30 and 37** were prepared following the general procedure. Namely, **55** was neutralized with equimolar NaOH to give **30**. A solution of the 2-benzimidazolylguanidine **57** (700 mg, 4.0 mmol) in anhydrous $CH_2Cl_2$ (10 mL) was cooled to 0 °C. A solution of hydrogen chloride in dioxane (4 M, 1.0 mL) was added dropwise with stirring. The reaction mixture was allowed to warm to rt and stirred for 1-2 h. The resulting solid was collected by filtration, washed with cold diethyl ether (2-3 × 5 mL), and dried under reduced pressure to afford **37**·Cl. A stock solution (10 mM) of **37**·Cl was prepared by dissolving **37**·Cl (7.9 mg, 40.0 μmol) in DMSO (4 mL). An aliquot of this solution (0.4 mL) and an aliquot of the **30**·Na stock solution (0.4 mL) were diluted with $CHCl_3$/DMSO 3:0.2, and sonicated to give a solution of 1.0 mM **37/30** in $CHCl_3$/DMSO 3:1.

**Components 38 and 39**: **38/39** (2.60 mg, 4.0 μmol) was suspended in $CHCl_3$/DMSO 3:1. The suspension was dissolved after sonication for 10 min. The final concentrations of each component were as follows: 1.0 mM **38/39**.

**Components 40 and $Br^-$**: **40**·Br (3.0 mg, 4.0 μmol) was suspended in $CHCl_3$/DMSO 3:1 (4 mL). The suspension was dissolved after sonication for 10 min.

### 3.3. General Procedure for EFC of Diels-Alder Reactions

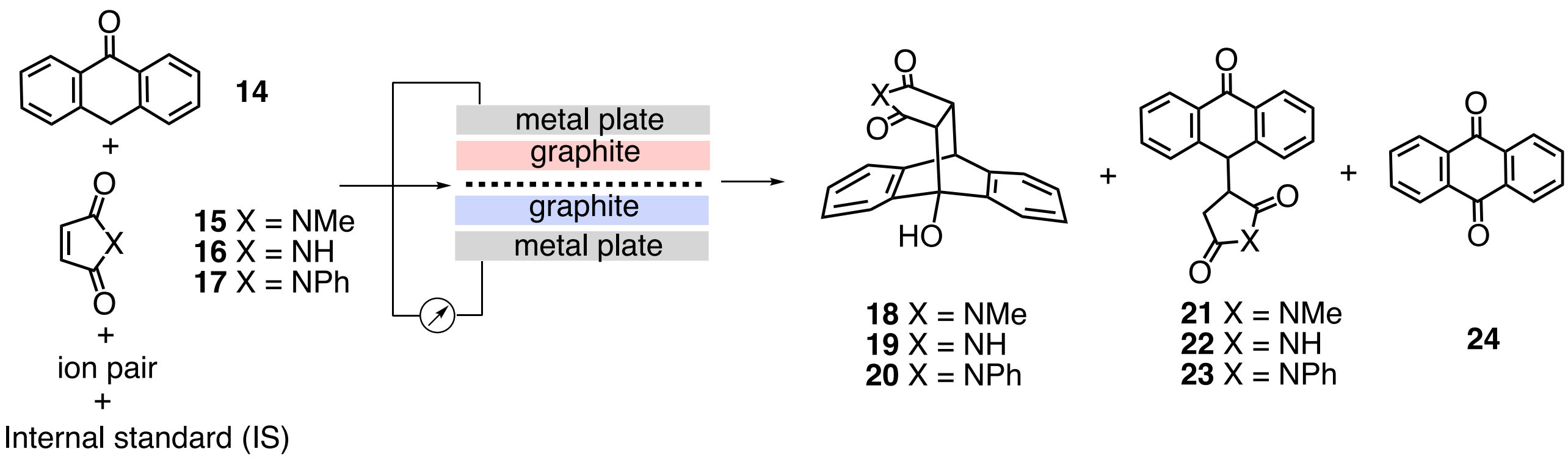


**Scheme S10.** Microfluidic DA-EFC with anthrone **14** and maleimides.

A stock solution of the ion pair (1 mM) and 1,3,5-tri-*tert*-butylbenzene as an internal standard (IS, 16.5 mM) was prepared in anhydrous $CHCl_3$/DMSO 3:1. Two aliquots of this solution, 2.0 mL each, were taken. Anthrone **14** (50 mM) was dissolved in the first aliquot, and maleimide **15**/**16**/**17** (75 mM) was dissolved in the second aliquot. The resulting two solutions were loaded separately into two syringes. The solution was injected at 5 µL/min using a syringe pump into the microfluidic capacitor under constant voltage. The initial 0.36 mL, corresponding to three reactor volumes, was discarded before collecting each sample to ensure that a steady state of the system had been reached at the desired voltage. Then, 150 µL of reaction mixture was collected over 30 min. Collected samples were diluted with additional $CDCl_3$ and the $^1$H NMR spectrum was recorded right after every experiment. Yields ($Y_m$) were determined by comparing the integrals of the pertinent product signals to that of the IS. All experiments were conducted under $I = 0.000$ A. The anhydrous $CHCl_3$ used for EFC was stabilized with 0.5-1% EtOH.

The resulting dependence of the microfluidic yield ($Y_m$) on the applied voltage ($V$) was plotted as *YV* curves and fitted with Equation S1 to retrieve the half effective voltage ($V_{50}$), the gating coefficient ($n_g$), the yield without the applied field ($Y_m^0$) and the theoretical maximum yield increase ($Y_m^\infty$).

$$Y_m = Y_m^{\infty} \cdot (V^{n_g} / (V_{50}^{n_g} + V^{n_g})) + Y_m^0 \quad \text{(Eq S1)}$$

Critical voltages $V_c$ were estimated from the fit curve as the voltage at which $Y_m = Y_m^0 + 10\%$ was observed.

### 3.4. HPLC Analysis

Enantioselectivities (*ee*'s) were estimated by HPLC analysis. Conditions for **18**: Chiralpak ID column (4.6 × 250 mm, *n*-hexane/*i*-PrOH 90:10, rt, 1.0 mL/min, 260 nm); conditions for **20**: Chiralpak IA column (4.6 × 250 mm, *n*-hexane/*i*-PrOH 80:20, rt, 0.8 mL/min, 260 nm).

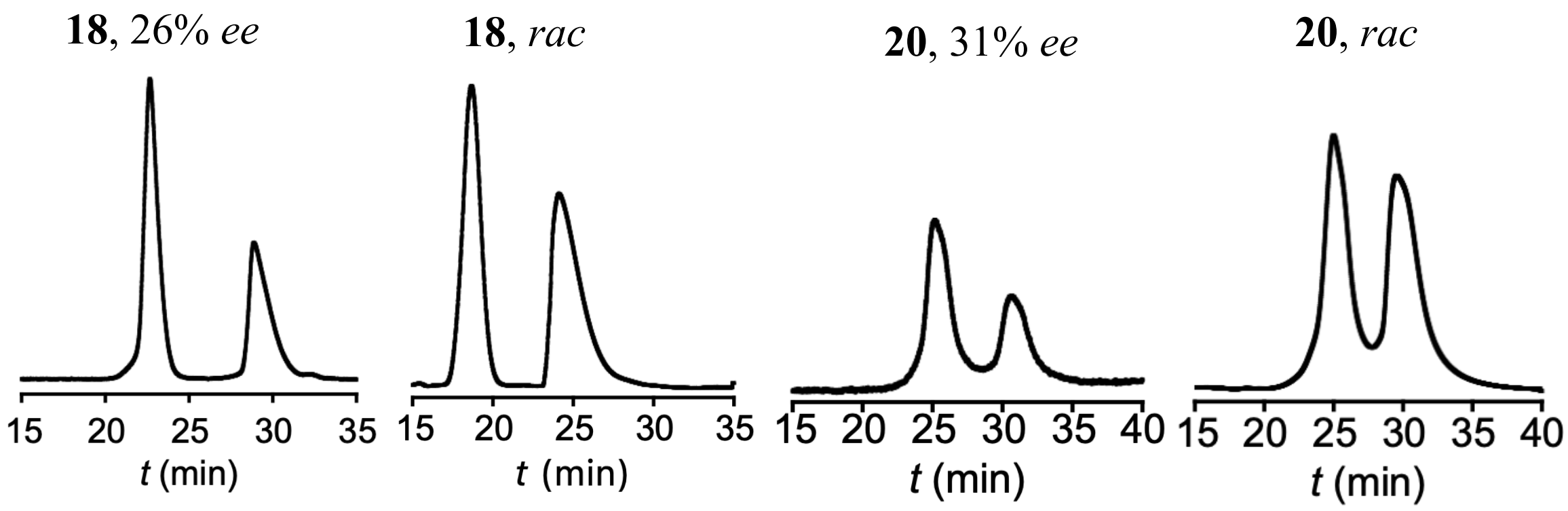


**Figure S4.** Representative HPLC traces of **18** and **20**.

## 3.5. NMR Analysis

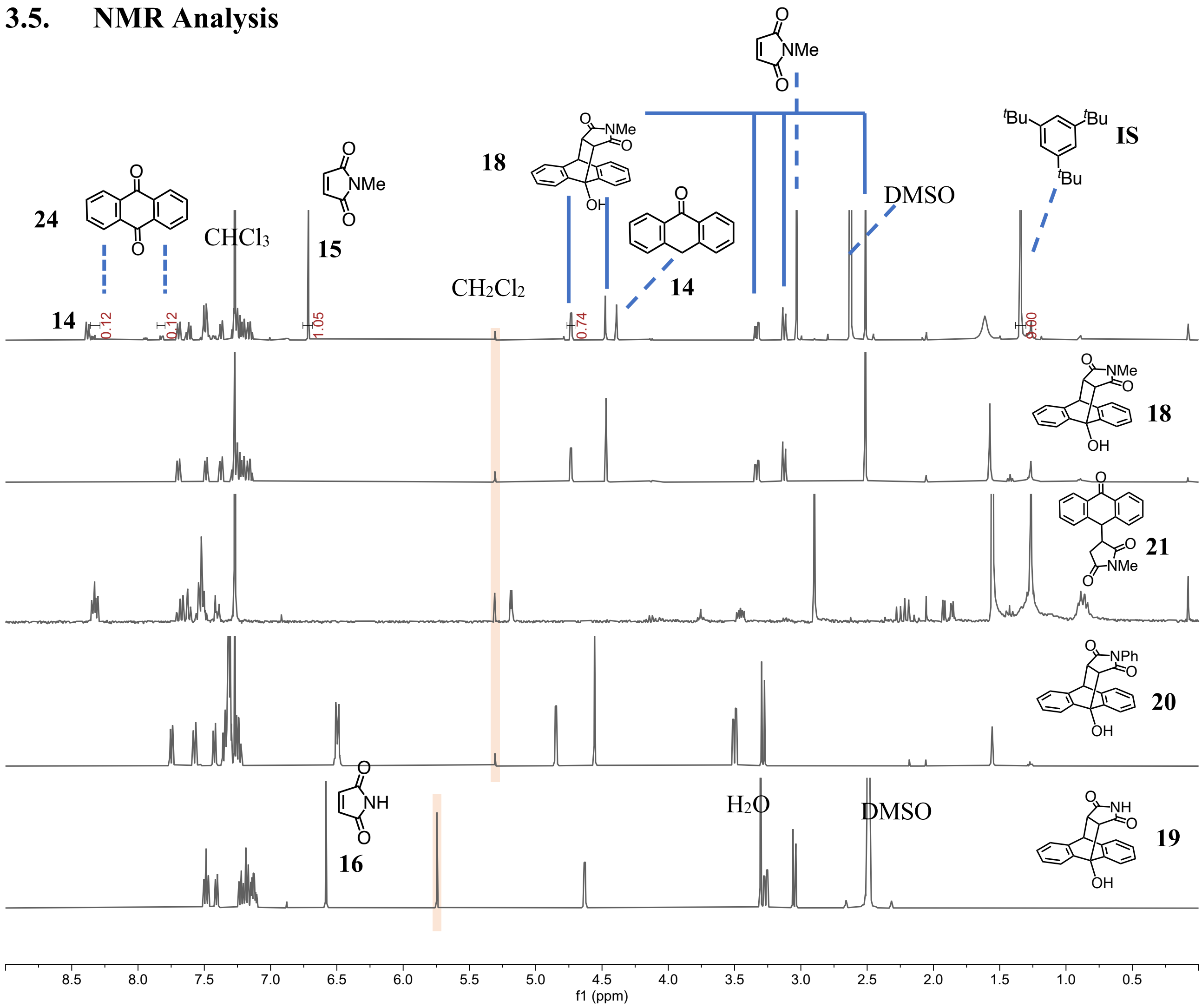


**Figure S5.** $^{1}$H NMR spectrum of a representative reaction mixture, containing starting materials **14** and **15, 16, 17**, oxidation product **24**, DA product **18,** and the internal standard **IS**, compared to the spectra of **18**, **21**, **20**, and **19** in $CDCl_3$ (top to bottom).

**Compound 24** was detected following the previously reported procedure.[S16]

### 3.6. Voltage Dependence of Diels-Alder Reactions

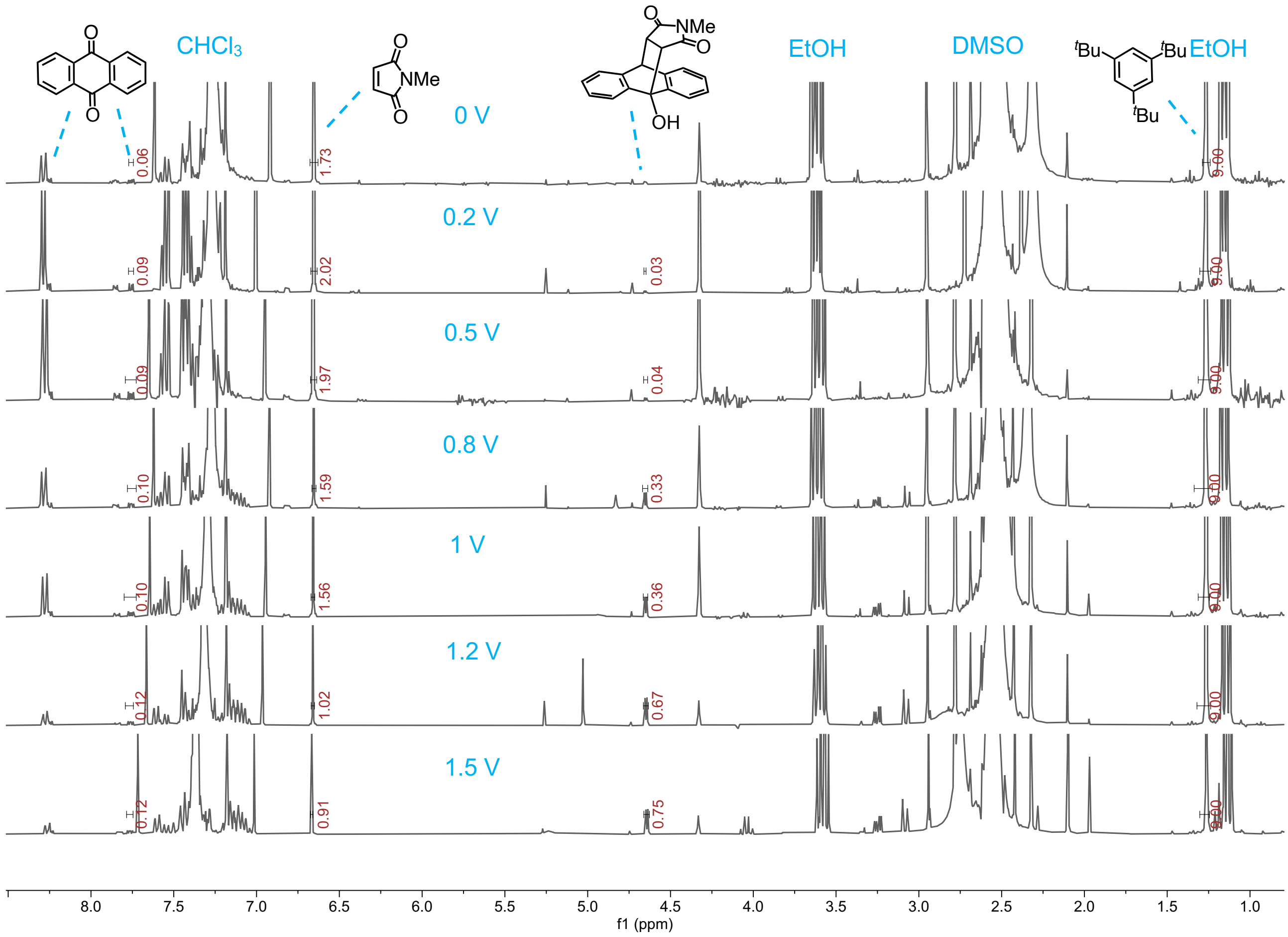


**Figure S6.** $^{1}$H NMR spectra (400 MHz, $CDCl_3$) of reaction mixtures obtained from **14** (50 mM), **15** (75 mM), **25** (1 mM) and **26** (1 mM) in $CHCl_3$/DMSO 3:1 (4 mL) after one passage through the microfluidic capacitor, flow rate 5 µL/min, 100 µm foil, graphite electrodes, with $V$ = 0.0, 0.2, 0.5, 0.8, 1.0, 1.2 and 1.5 V (from top to bottom) applied, at rt.

**Table S1.** EFC with substrates **14** (50 mM), **15** (75 mM) and Helmholtz components **25** (1 mM) and **26** (1 mM) in $CHCl_3$/DMSO 3:1.[a]

| Entry | $V$ (V)[b] | $I$ (A)[c] | $Y_m^{\mathbf{18}}$ (%)[d] | $Y_m^{\mathbf{21}}$ (%)[e] | $Y_m^{\mathbf{24}}$ (%)[f] | *ee* (%)[g] |
|---|---|---|---|---|---|---|
| 1 | 0 | 0.000 | 0 | 0 | 2 | - |
| 2 | 0.2 | 0.000 | 3 | 0 | 2 | - |
| 3 | 0.5 | 0.000 | 4 | 0 | 2 | - |
| 4 | 0.8 | 0.000 | 33 | 0 | 3 | - |
| 5 | 1.0 | 0.000 | 36 | 0 | 3 | - |
| 6 | 1.2 | 0.000 | 67 | 0 | 3 | 7 |
| 7 | 1.5 | 0.000 | 75 | 0 | 3 | 7 |

[a]In microfluidic capacitor, flow rate 5 µL/min, 100 µm foil, graphite electrodes, with 1,3,5-tri-*tert*-butylbenzene as internal standard (IS), rt, flowrate 5 µL/min. [b]Applied voltage, in volt. [c]Steady-state current measured, in ampere. [d]Microfluidic yield of DA product **18**, in percent. [e]Microfluidic yield of Michael product **21**, in percent. [f]Microfluidic yield of oxidation product **24**, in percent. [g]The *ee* value of DA product **18**, in percent.

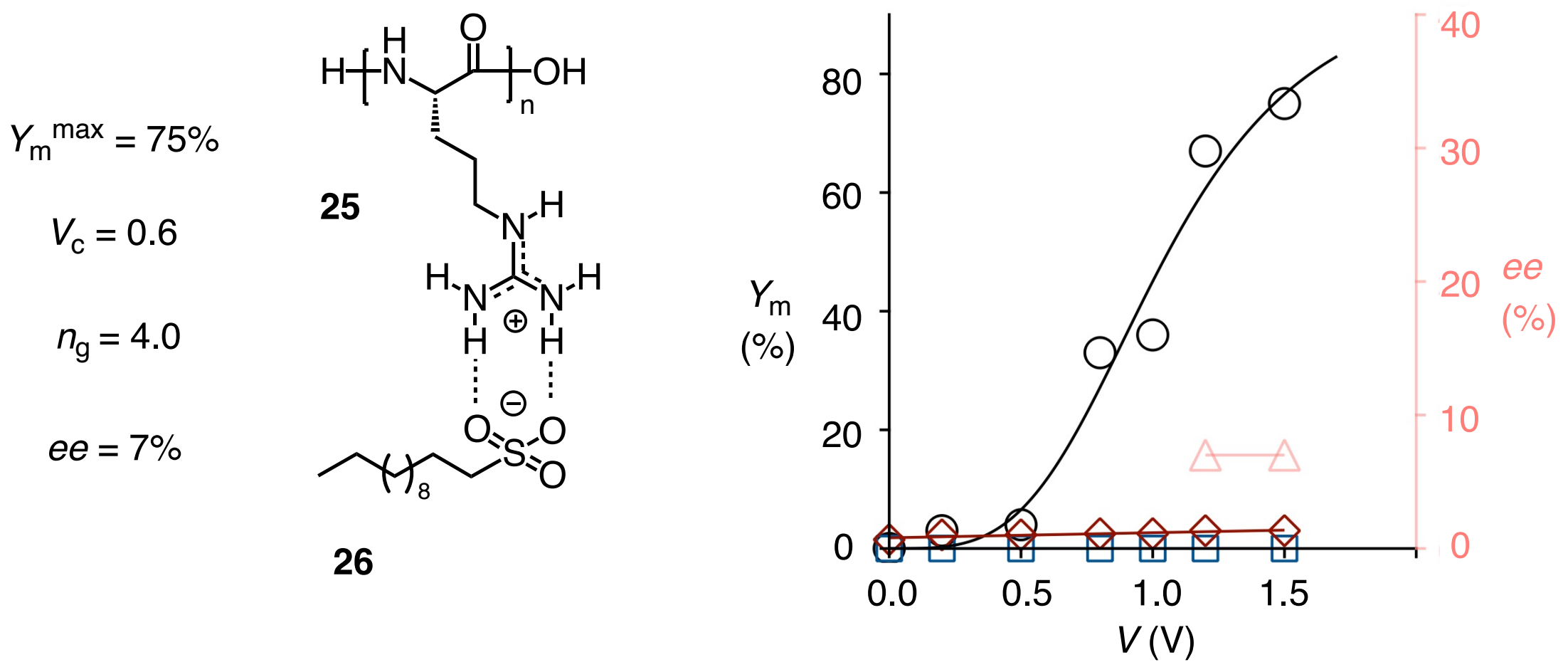


**Figure S7**. Dependence of microfluidic yield $Y_m$ (black, left, **18**: circles, **21**: squares, **24**: diamonds) and *ee* (right, red, **18**: triangles) on the applied voltage $V$ for substrates **14** (50 mM) and **15** (75 mM) with Helmholtz components **25** and **26** (1 mM, 2 mol%) in $CHCl_3$/DMSO 3:1, flow rate 5 µL/min, 100 µm foil, graphite electrodes, rt, with curve fit to Hill equation (circles only) and indication of $Y_m^{max}$ as maximal microfluidic yield, $V_c$ as critical voltage, $n_g$ as gating coefficient, and *ee* as enantioselectivity for **18**.

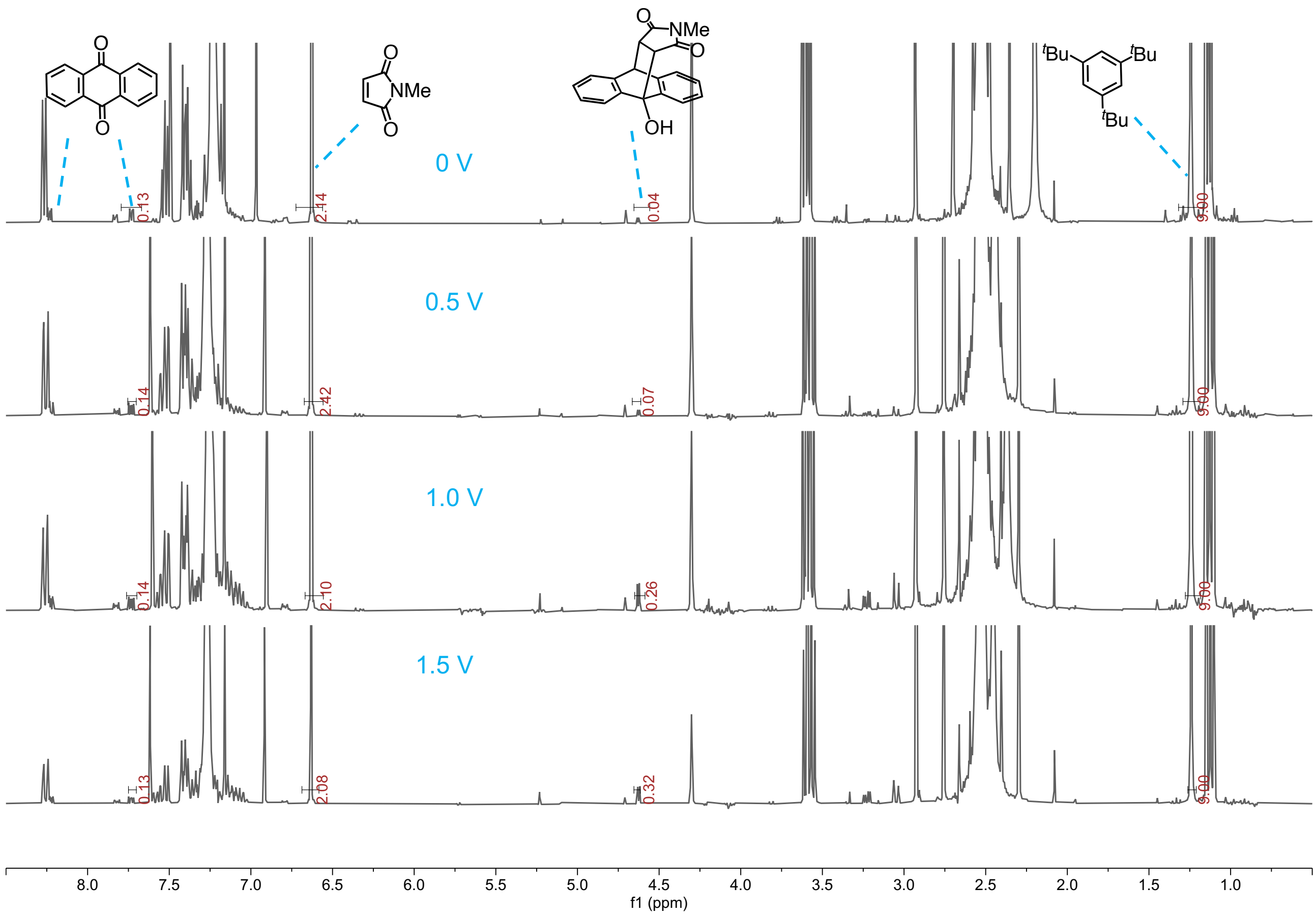


**Figure S8.** $^1$H NMR spectra (400 MHz, $CDCl_3$) of reaction mixtures obtained from **14** (50 mM) and **15** (75 mM) in $CHCl_3$/DMSO 3:1 (4 mL) after one passage through the microfluidic capacitor at 5 µL/min with $V$ = 0.0, 0.5, 1.0 and 1.5 V (from top to bottom) applied, at rt.

**Table S2.** EFC with **14** (50 mM) and **15** (75 mM).[a]

| Entry | *V* (V)[b] | *I* (A)[c] | $Y_m$**18** (%)[d] | $Y_m$**21** (%)[e] | $Y_m$**24** (%)[f] | *ee* (%)[g] |
|---|---|---|---|---|---|---|
| 1 | 0 | 0.000 | 4 | 0 | 3.3 | - |
| 2 | 0.5 | 0.000 | 7 | 0 | 3.5 | - |
| 3 | 1.0 | 0.000 | 26 | 0 | 3.5 | - |
| 4 | 1.5 | 0.000 | 32 | 0 | 3.3 | - |

[a]In microfluidic capacitor, flow rate 5 µL/min, 100 µm foil, graphite electrodes, with 1,3,5-tri-*tert*-butylbenzene as internal standard (IS), rt, flowrate 5 µL/min. [b]Applied voltage, in volt. [c]Steady-state current measured, in ampere. [d]Microfluidic yield of DA product **18**, in percent. [e]Microfluidic yield of Michael product **21**, in percent. [f]Microfluidic yield of oxidation product **24**, in percent. [g]The *ee* value of DA product **18**, in percent.

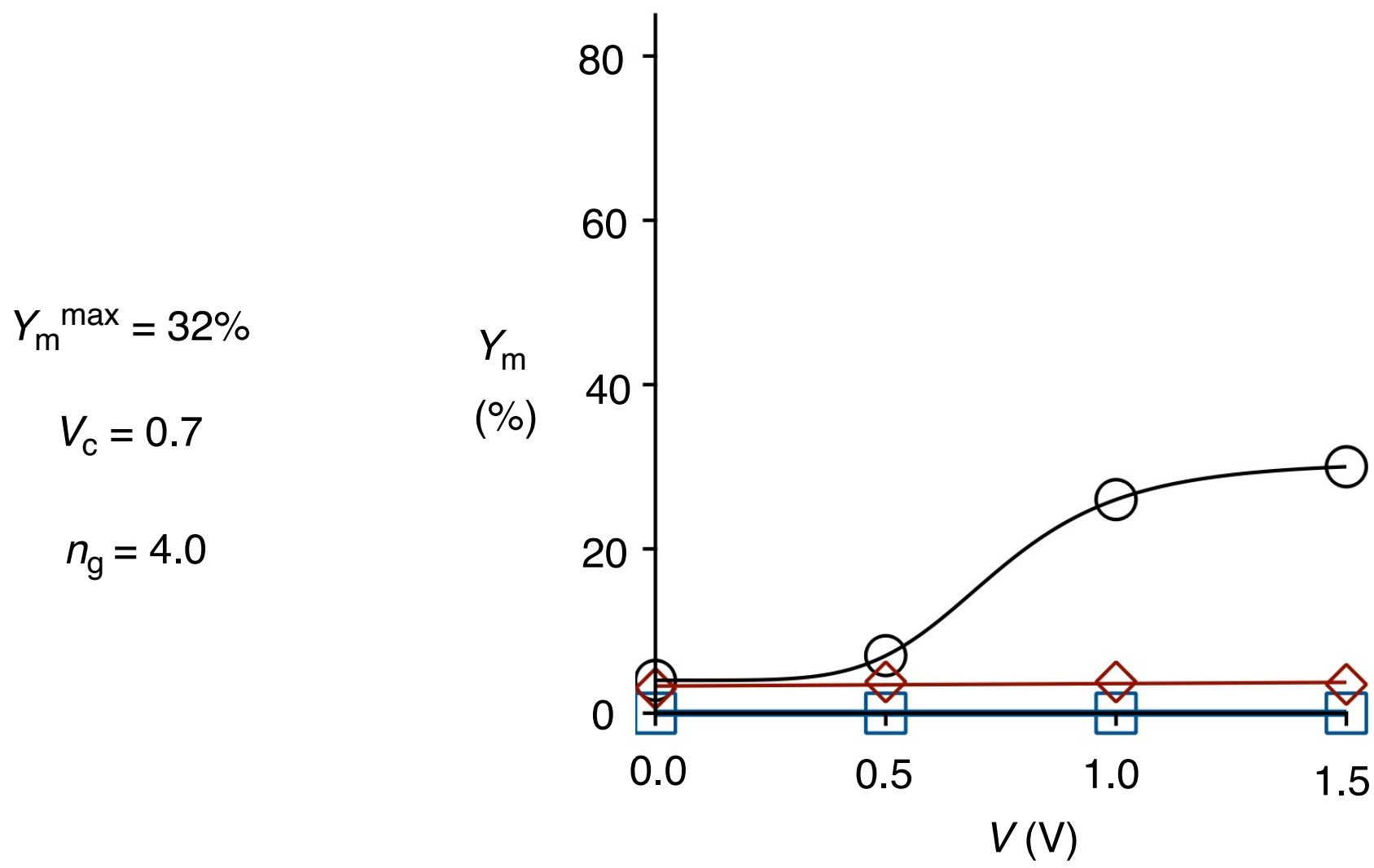


**Figure S9**. Dependence of microfluidic yield $Y_m$ (black, left, **18**: circles, **21**: squares, **24**: diamonds) on the applied voltage *V* for substrates **14** (50 mM) and **15** (75 mM) in $CHCl_3$/DMSO 3:1, flow rate 5 µL/min, 100 µm foil, graphite electrodes, rt, with curve fit to Hill equation (circles only) and indication of $Y_m^{max}$ as maximal microfluidic yield, $V_c$ as critical voltage, $n_g$ as gating coefficient.

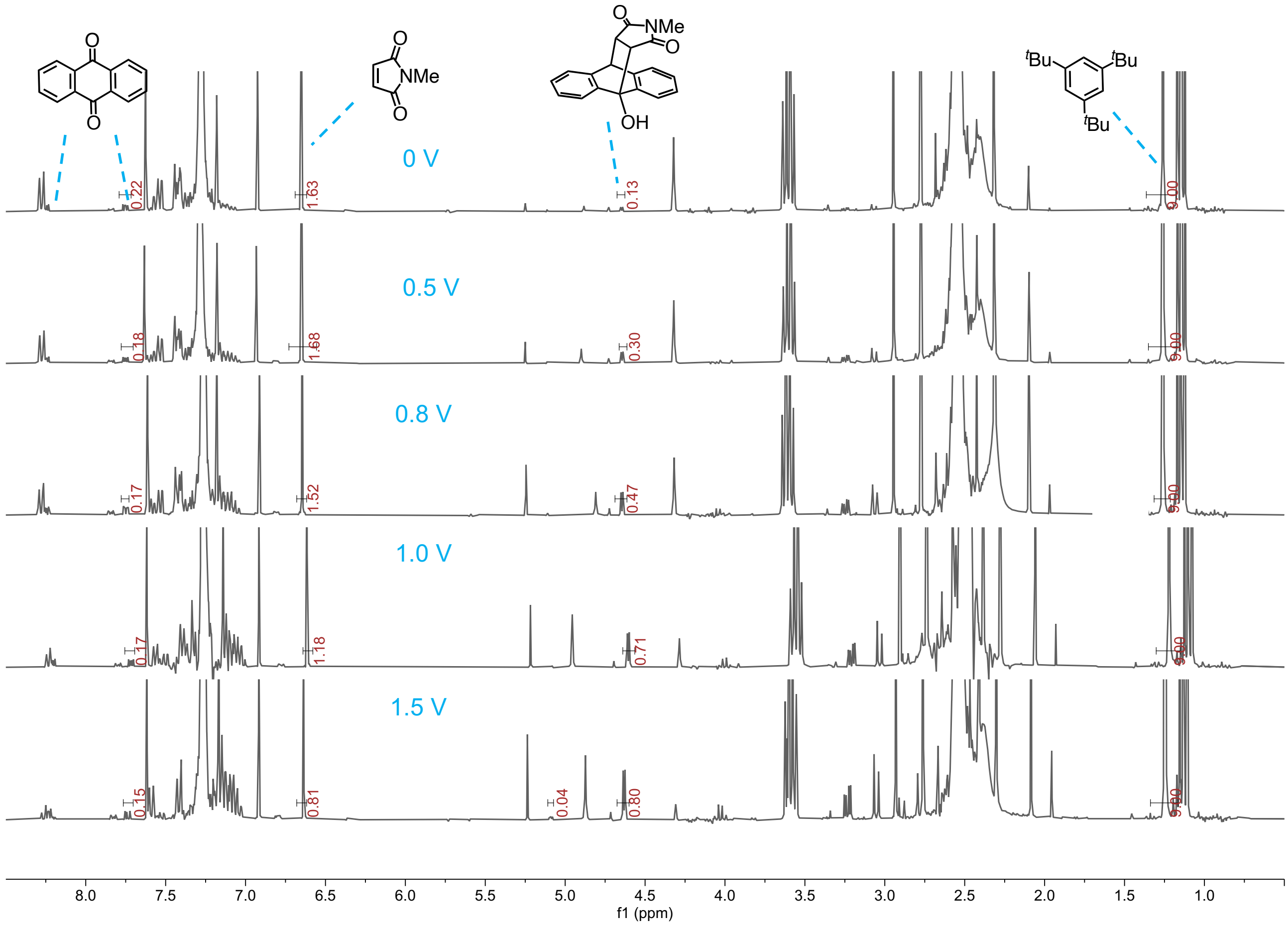


**Figure S10.** $^{1}$H NMR spectra (400 MHz, $CDCl_3$) of reaction mixtures obtained from **14** (50 mM), **15** (75 mM) and Helmholtz components **27** and $Cl^-$ (1 mM) in $CHCl_3$/DMSO 3:1 (4 mL) after one passage through the microfluidic capacitor at 5 µL/min with $V$ = 0.0, 0.5, 0.8, 1.0 and 1.5 V (from top to bottom) applied, at rt.

**Table S3.** EFC with substrates **14** (50 mM), **15** (75 mM) and Helmholtz components **27** and $Cl^-$ (1 mM) in $CHCl_3$/DMSO 3:1.[a]

| Entry | $V$ (V)[b] | $I$ (A)[c] | $Y_m^{\mathbf{18}}$ (%)[d] | $Y_m^{\mathbf{21}}$ (%)[e] | $Y_m^{\mathbf{24}}$ (%)[f] | *ee* (%)[g] |
|---|---|---|---|---|---|---|
| 1 | 0 | 0.000 | 13 | 0 | 5.5 | - |
| 2 | 0.5 | 0.000 | 30 | 0 | 4.5 | - |
| 3 | 0.8 | 0.000 | 47 | 0 | 4.3 | |
| 4 | 1.0 | 0.000 | 71 | 0 | 4.3 | 7 |
| 5 | 1.5 | 0.000 | 80 | 4 | 3.8 | 7 |

[a]In microfluidic capacitor, flow rate 5 µL/min, 100 µm foil, graphite electrodes, with 1,3,5-tri-*tert*-butylbenzene as internal standard (IS), rt, flowrate 5 µL/min. [b]Applied voltage, in volt. [c]Steady-state current measured, in ampere. [d]Microfluidic yield of DA product **18**, in percent. [e]Microfluidic yield of Michael product **21**, in percent. [f]Microfluidic yield of oxidation product **24**, in percent. [g]The *ee* value of DA product **18**, in percent.

$Y_m^{max}$ = 80%

$V_c$ = 0.4

$n_g$ = 3.0

*ee* = 7%

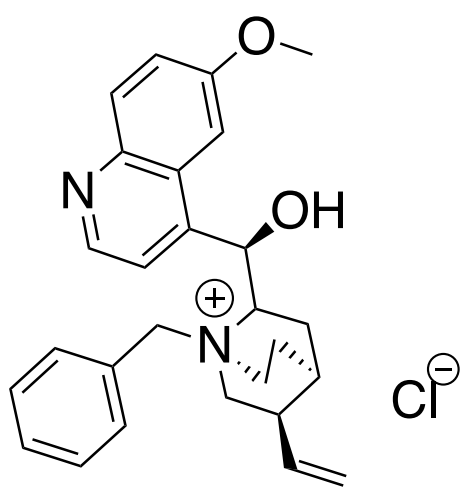


**27**

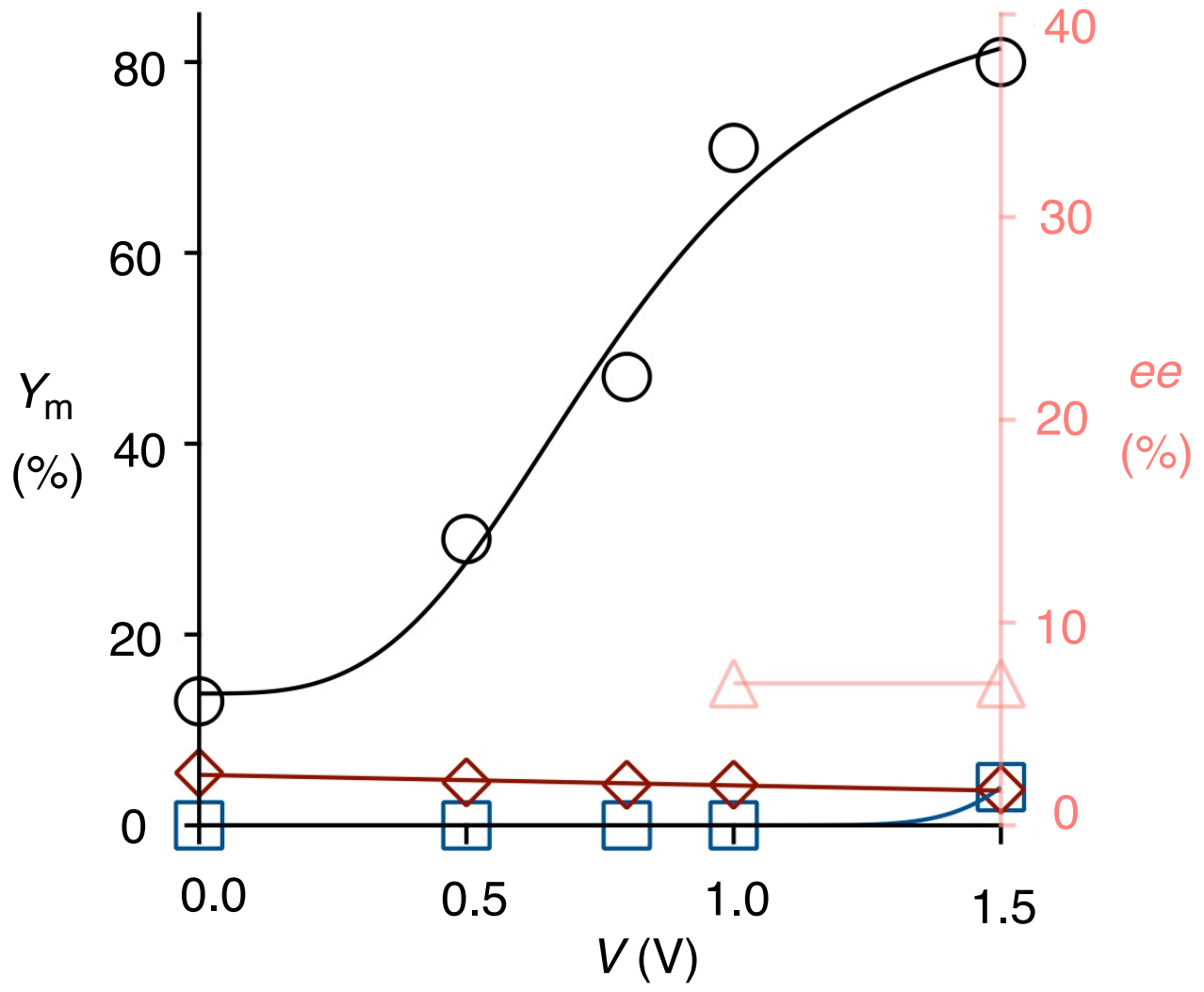


**Figure S11**. Dependence of microfluidic yield $Y_m$ (black, left, **18**: circles, **21**: squares, **24**: diamonds) and *ee* (right, red, **18**: triangles) on the applied voltage *V* for substrates **14** (50 mM), **15** (75 mM) with Helmholtz components **27** and $Cl^-$ (1 mM, 2 mol%) in $CHCl_3$/DMSO 3:1, flow rate 5 µL/min, 100 µm foil, graphite electrodes, rt, with curve fit to Hill equation (circles only) and indication of $Y_m^{max}$ as maximal microfluidic yield, $V_c$ as critical voltage, $n_g$ as gating coefficient and *ee* as enantioselectivity for **18**.

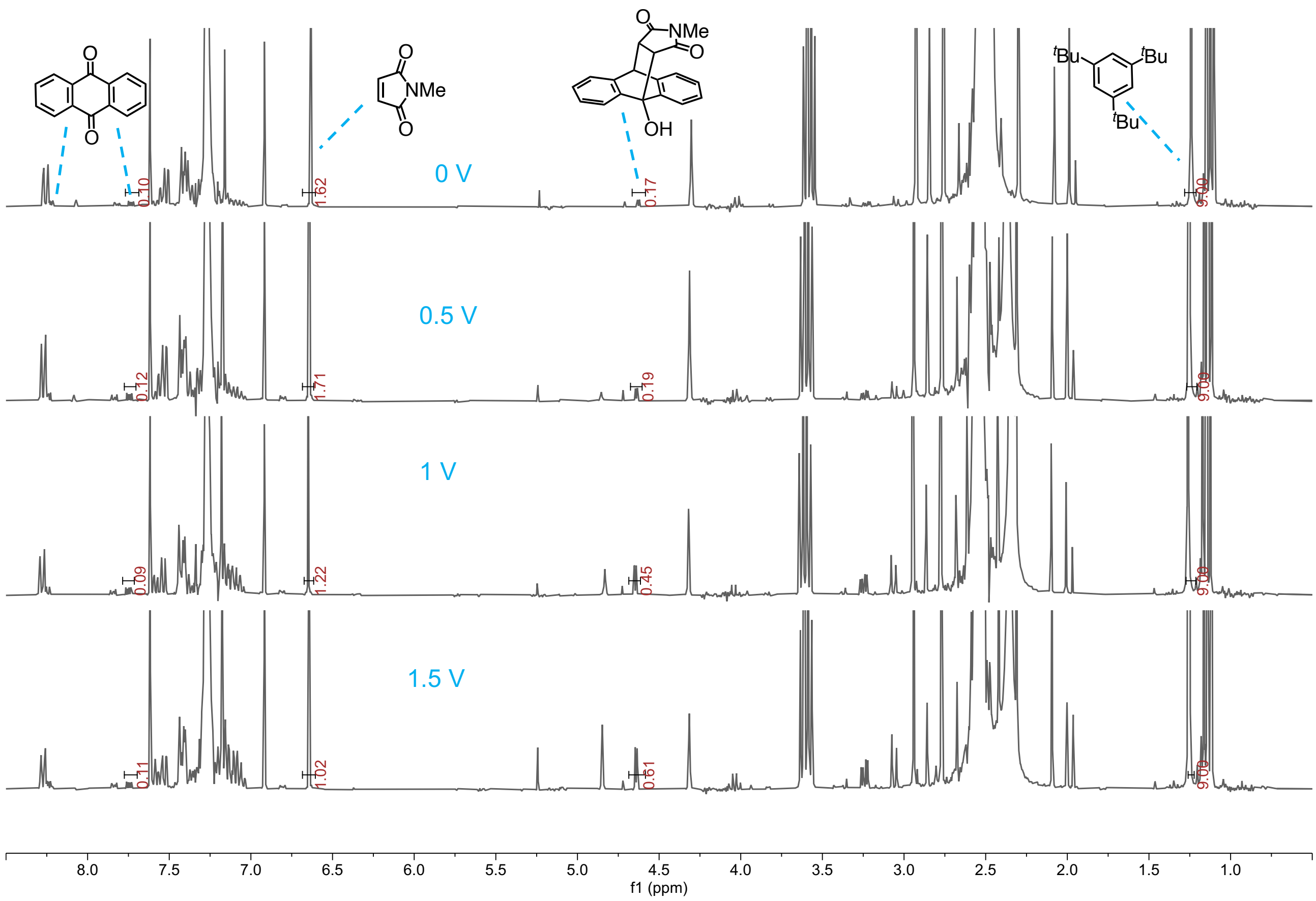


**Figure S12.** $^1$H NMR spectra (400 MHz, $CDCl_3$) of reaction mixtures obtained from **14** (50 mM), **15** (75 mM), Helmholtz components **27** and $Cl^-$ (1 mM) and **28** (2.5 mM) in $CHCl_3$/DMSO 3:1 (4 mL) after one passage through the microfluidic capacitor at 5 µL/min with $V$ = 0.0, 0.5, 1.0 and 1.5 V (from top to bottom) applied, at rt.

**Table S4.** EFC with substrates **14** (50 mM), **15** (75 mM), Helmholtz components **27** and $Cl^-$ (1 mM) and ion-pair breaker **28** (2.5 mM) in $CHCl_3$/DMSO 3:1.[a]

| Entry | *V* (V)[b] | *I* (A)[c] | $Y_m^{\mathbf{18}}$ (%)[d] | $Y_m^{\mathbf{21}}$ (%)[e] | $Y_m^{\mathbf{24}}$ (%)[f] | *ee* (%)[g] |
|---|---|---|---|---|---|---|
| 1 | 0 | 0.000 | 17 | 0 | 5.5 | - |
| 2 | 0.5 | 0.000 | 19 | 0 | 4.5 | - |
| 3 | 0.8 | 0.000 | 45 | 0 | 4.3 | |
| 4 | 1.0 | 0.000 | 61 | 0 | 4.3 | 7 |

[a]In microfluidic capacitor, flow rate 5 µL/min, 100 µm foil, graphite electrodes, with 1,3,5-tri-*tert*-butylbenzene as internal standard (IS), rt, flowrate 5 µL/min. [b]Applied voltage, in volt. [c]Steady-state current measured, in ampere. [d]Microfluidic yield of DA product **18**, in percent. [e]Microfluidic yield of Michael product **21**, in percent. [f]Microfluidic yield of oxidation product **24**, in percent. [g]The *ee* value of DA product **18**, in percent.

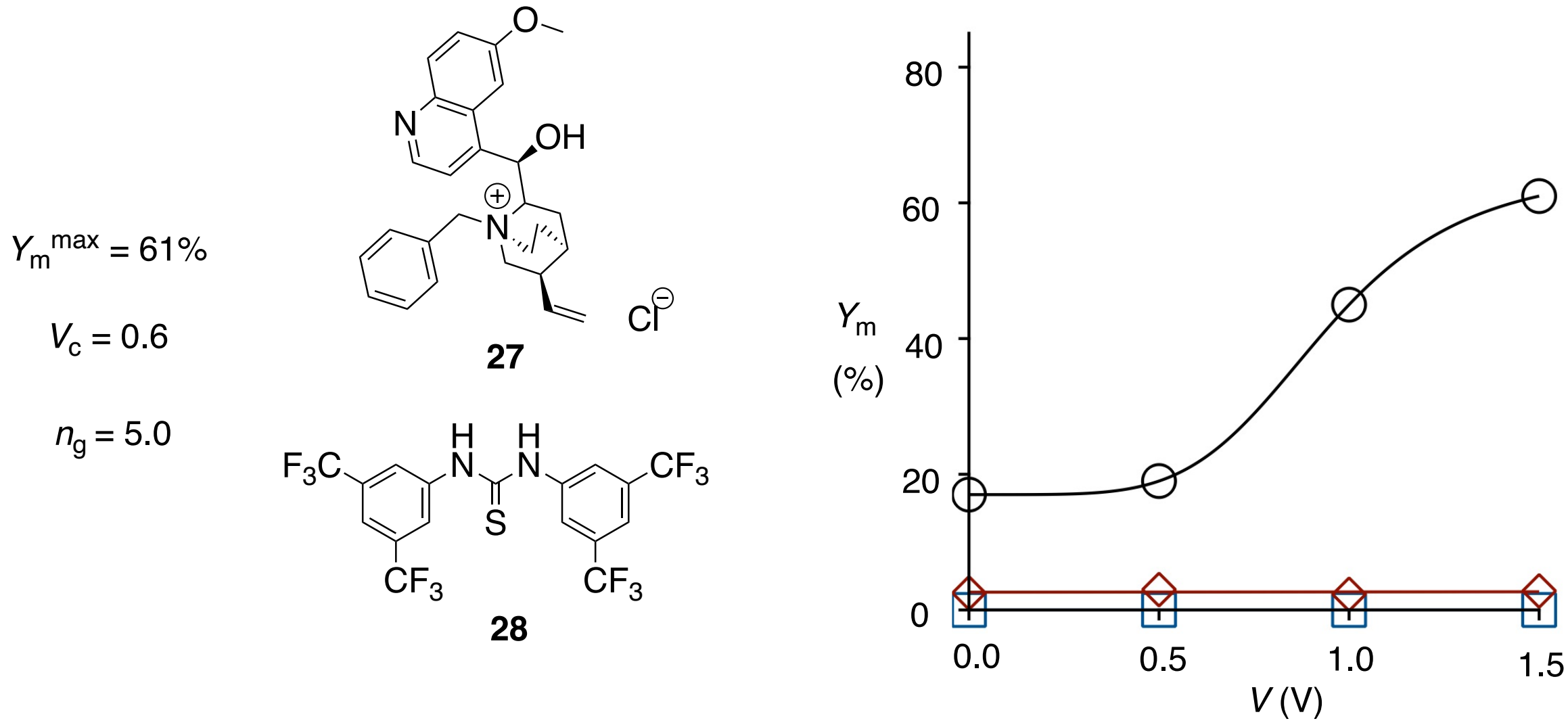


**Figure S13**. Dependence of microfluidic yield $Y_m$ (**18**: circles, **21**: squares, **24**: diamonds) and on the applied voltage $V$ for substrates **14** (50 mM), **15** (75 mM), Helmholtz components **27** and $Cl^-$ (1 mM, 2 mol%) and ion-pair breaker **28** (2.5 mM) in $CHCl_3$/DMSO 3:1, flow rate 5 µL/min, 100 µm foil, graphite electrodes, rt, with curve fit to Hill equation (circles only) and indication of $Y_m^{max}$ as maximal microfluidic yield, $V_c$ as critical voltage, $n_g$ as gating coefficient.

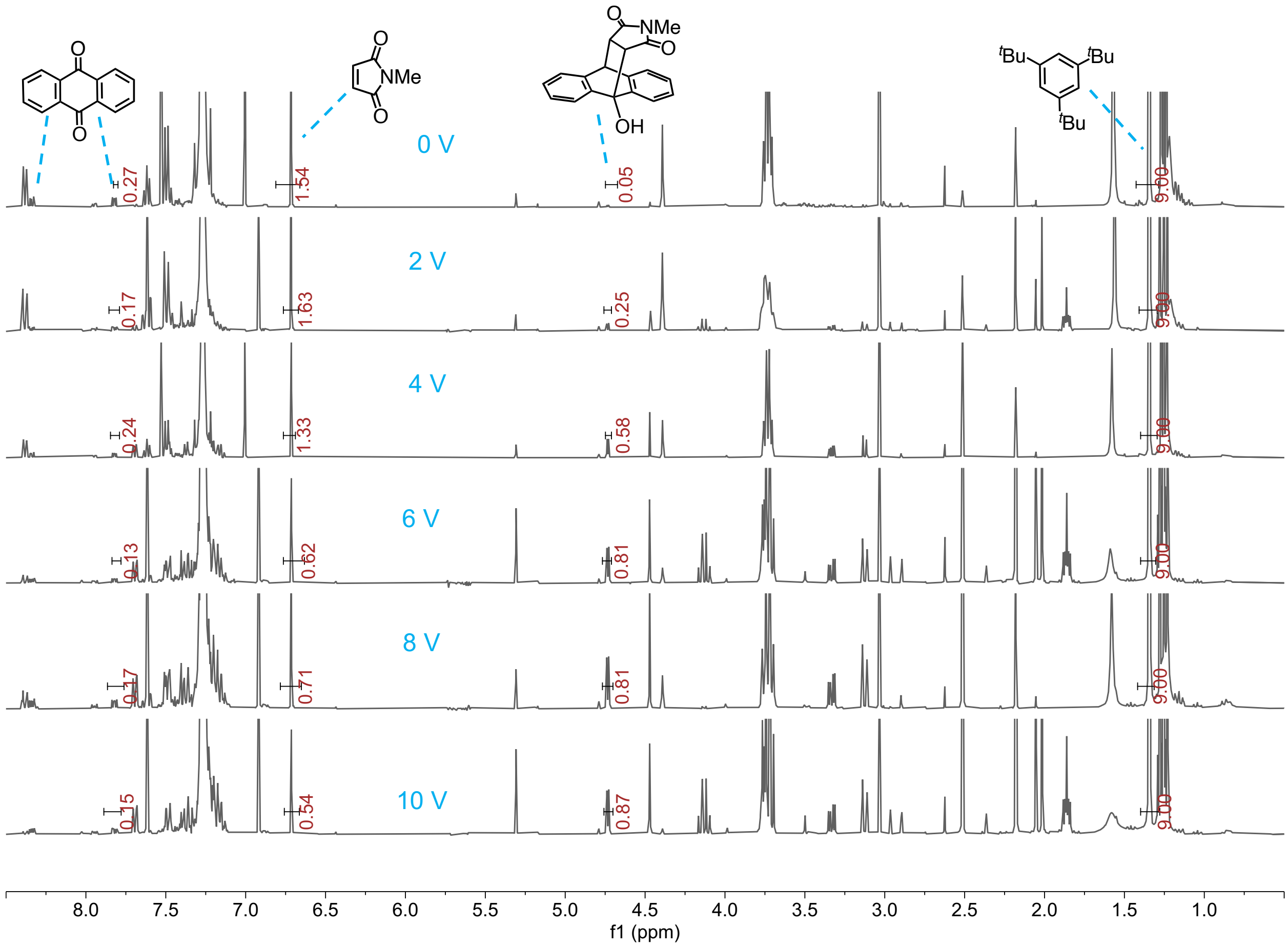


**Figure S14.** $^1$H NMR spectra (400 MHz, $CDCl_3$) of reaction mixtures obtained from **14** (50 mM), **15** (75 mM) and Helmholtz components **27** and $Cl^-$ (1 mM) in $CHCl_3$ (4 mL) after one passage through the microfluidic capacitor at 5 µL/min with $V$ = 0, 2, 4, 6, 8 and 10 V (from top to bottom) applied, at rt.

**Table S5**. EFC with substrates **14** (50 mM), **15** (75 mM) and Helmholtz components **27** and $Cl^-$ (1 mM) in $CHCl_3$ (4 mL).[a]

| Entry | $V$ (V)[b] | $I$ (A)[c] | $Y_m^{\mathbf{18}}$ (%)[d] | $Y_m^{\mathbf{21}}$ (%)[e] | $Y_m^{\mathbf{24}}$ (%)[f] | *ee* (%)[g] |
|---|---|---|---|---|---|---|
| 1 | 0 | 0.000 | 5 | 0 | 6.7 | - |
| 2 | 2 | 0.000 | 25 | 0 | 4.3 | - |
| 3 | 4 | 0.000 | 58 | 0 | 6.0 | 26 |
| 4 | 6 | 0.000 | 81 | 0 | 3.3 | 26 |
| 5 | 8 | 0.000 | 81 | 4 | 4.3 | 26 |
| 6 | 10 | 0.000 | 87 | 18 | 3.8 | 26 |

[a]In microfluidic capacitor, flow rate 5 µL/min, 100 µm foil, graphite electrodes, with 1,3,5-tri-*tert*-butylbenzene as internal standard (IS), rt, flowrate 5 µL/min. [b]Applied voltage, in volt. [c]Steady-state current measured, in ampere. [d]Microfluidic yield of DA product **18**, in percent. [e]Microfluidic yield of Michael product **21**, in percent. [f]Microfluidic yield of oxidation product **24**, in percent. [g]The *ee* value of DA product **18**, in percent.

$Y_m^{max}$ = 87%

$V_c$ = 1.5

$n_g$ = 3.0

*ee* = 26%

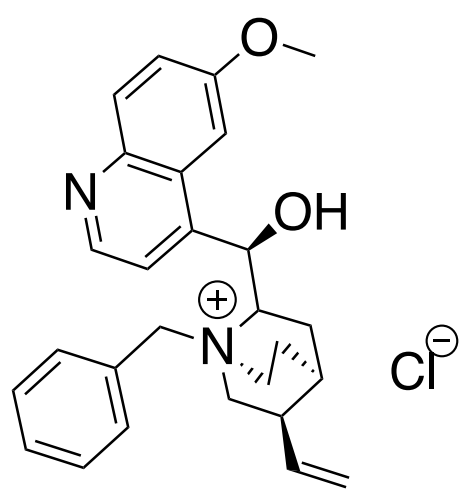


**27**

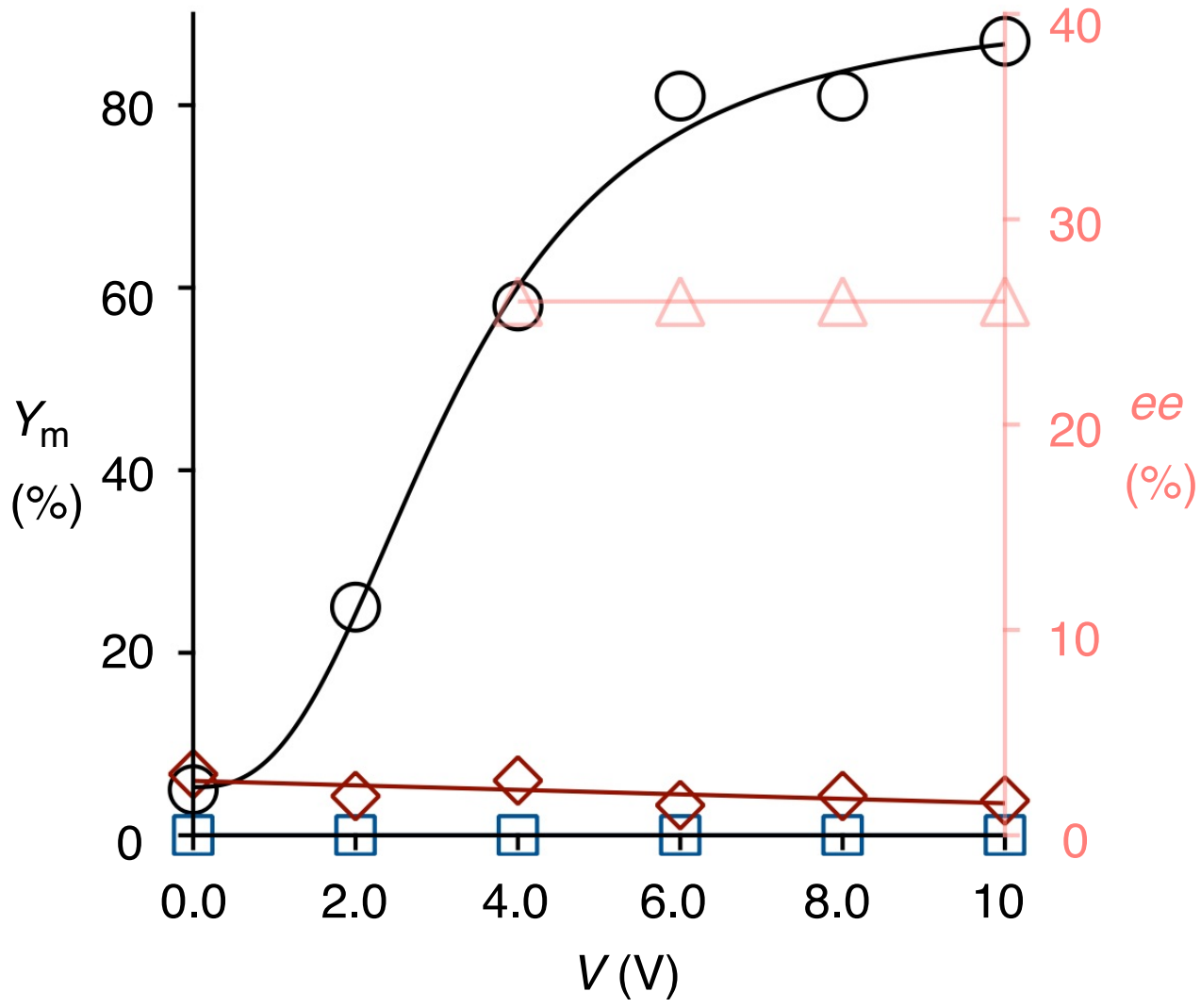


**Figure S15**. Dependence of microfluidic yield $Y_m$ (black, left, **18**: circles, **21**: squares, **24**: diamonds) and *ee* (right, red, **18**: triangles) on the applied voltage $V$ for substrates **14** (50 mM), **15** (75 mM), Helmholtz components **27** and $Cl^-$ (1 mM, 2 mol%) in $CHCl_3$ (4 mL), flow rate 5 µL/min, 100 µm foil, graphite electrodes, rt, with curve fit to Hill equation (circles only) and indication of $Y_m^{max}$ as maximal microfluidic yield, $V_c$ as critical voltage, $n_g$ as gating coefficient, and *ee* as enantioselectivity for **18**.

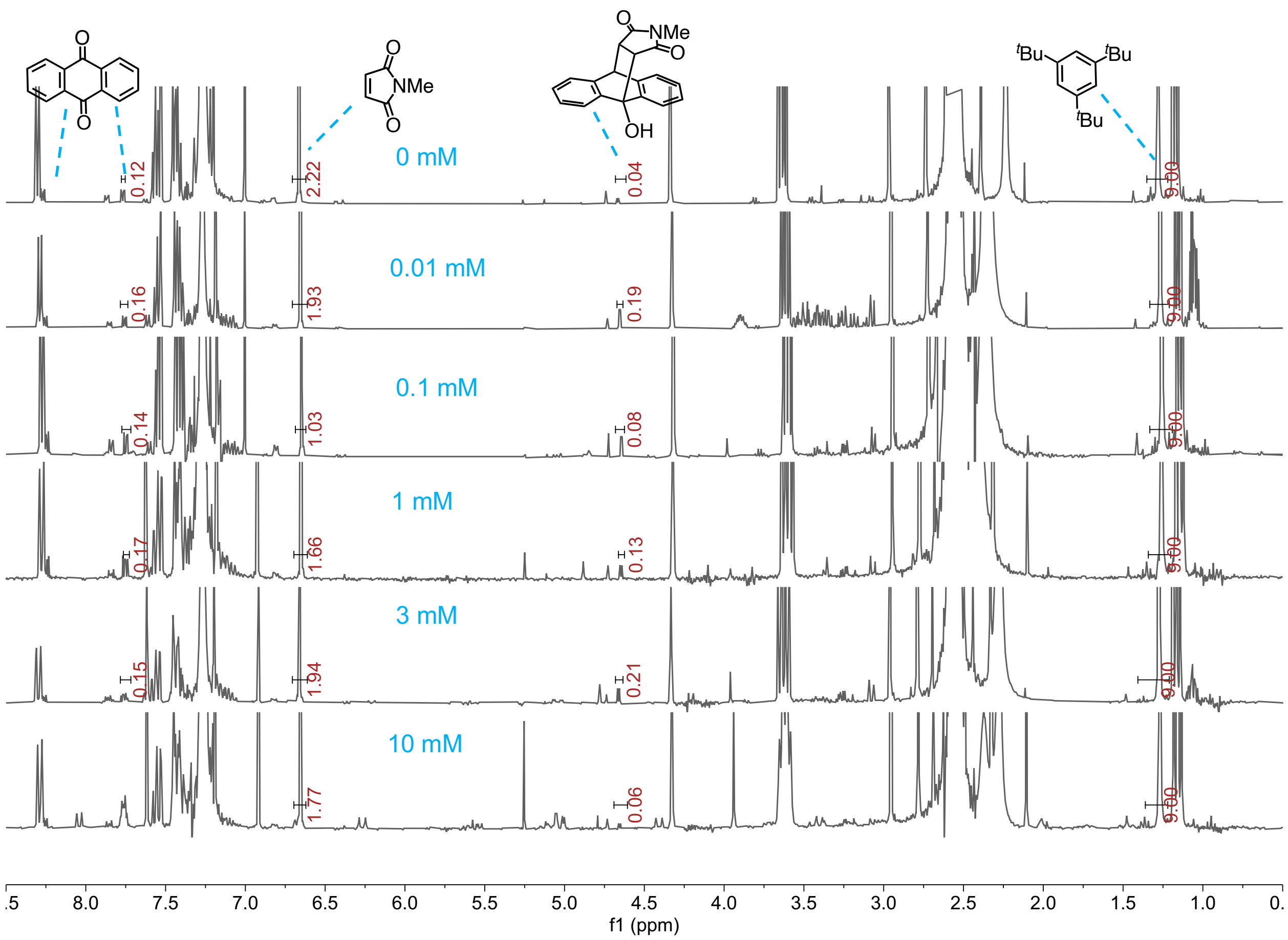


**Figure S16.** $^{1}$H NMR spectra (400 MHz, $CDCl_3$) of reaction mixtures obtained from **14** (50 mM), **15** (75 mM) and Helmholtz components **27** and $Cl^-$ (0, 0.01, 0.1, 1, 3, 10 mM, from top to bottom) in $CHCl_3$/DMSO 3:1 (4 mL) after one passage through the microfluidic capacitor at 5 µL/min with $V$ = 0.0 V applied, at rt.

**Table S6**. EFC with substrates **14** (50 mM), **15** (75mM) and Helmholtz components **27** and $Cl^-$ in $CHCl_3$/DMSO 3:1 at 0.0 V.[a]

| Entry | *c* (mM)[b] | *I* (A)[c] | $Y_m^{\mathbf{18}}$ (%)[d] | $Y_m^{\mathbf{21}}$ (%)[e] | $Y_m^{\mathbf{24}}$ (%)[f] | *ee* (%)[g] |
|---|---|---|---|---|---|---|
| 1 | 0 | 0.000 | 4 | 0 | 3.0 | - |
| 2 | 0.01 | 0.000 | 19 | 0 | 4.0 | - |
| 3 | 0.1 | 0.000 | 8 | 0 | 3.5 | - |
| 4 | 1 | 0.000 | 13 | 0 | 4.3 | - |
| 5 | 3 | 0.000 | 21 | 4 | 3.8 | - |
| 6 | 10 | 0.000 | 6 | 18 | 0 | - |

[a]In microfluidic capacitor, flow rate 5 µL/min, 100 µm foil, graphite electrodes, with 1,3,5-tri-*tert*-butylbenzene as internal standard (IS), rt, flowrate 5 µL/min. [b]Applied voltage, in volt. [c]Steady-state current measured, in ampere. [d]Microfluidic yield of DA product **18**, in percent. [e]Microfluidic yield of Michael product **21**, in percent. [f]Microfluidic yield of oxidation product **24**, in percent. [g]The *ee* value of DA product **18**, in percent.

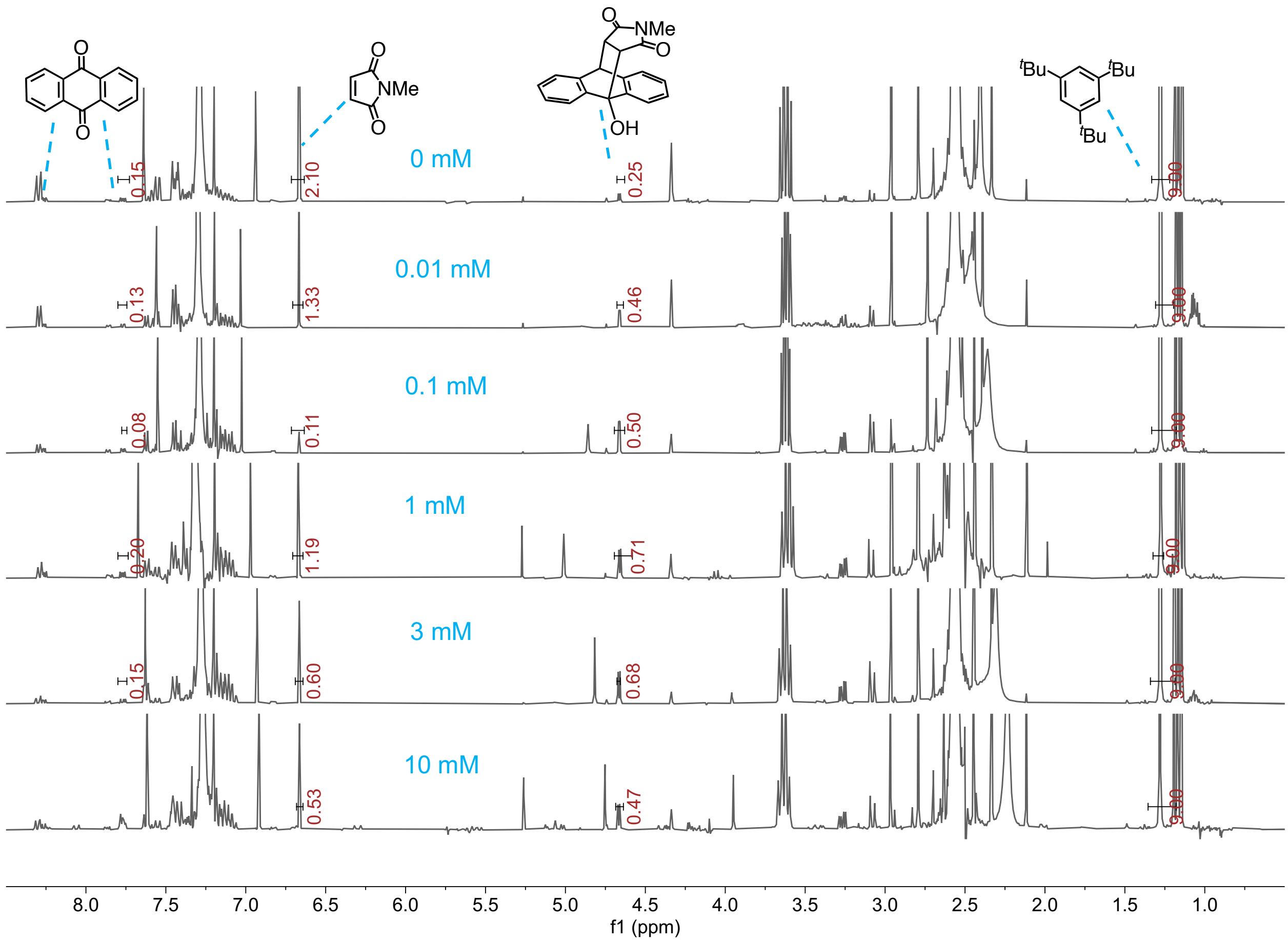


**Figure S17.** $^1$H NMR spectra (400 MHz, $CDCl_3$) of reaction mixtures obtained from **14** (50 mM), **15** (75 mM) and Helmholtz components **27** and $Cl^-$ (0, 0.01, 0.1, 1, 3, 10 mM, from top to bottom) in $CHCl_3$/DMSO 3:1 (4 mL) after one passage through the microfluidic capacitor at 5 µL/min with $V$ = 1.0 V applied, at rt.

**Table S7**. EFC with substrates **14** (50 mM), **15** (75 mM) and Helmholtz components **27** and $Cl^-$ in $CHCl_3$/DMSO 3:1 at 1.0 V.[a]

| Entry | *c* (mM)[b] | *I* (A)[c] | $Y_m^{\mathbf{18}}$ (%)[d] | $Y_m^{\mathbf{21}}$ (%)[e] | $Y_m^{\mathbf{24}}$ (%)[f] | *ee* (%)[g] |
|---|---|---|---|---|---|---|
| 1 | 0 | 0.000 | 25 | 0 | 3.8 | - |
| 2 | 0.01 | 0.000 | 46 | 0 | 3.3 | - |
| 3 | 0.1 | 0.000 | 50 | 0 | 2.0 | - |
| 4 | 1 | 0.000 | 71 | 0 | 5.0 | - |
| 5 | 3 | 0.000 | 68 | 0 | 3.8 | - |
| 6 | 10 | 0.000 | 47 | 0 | 0 | - |

[a]In microfluidic capacitor, flow rate 5 µL/min, 100 µm foil, graphite electrodes, with 1,3,5-tri-*tert*-butylbenzene as internal standard (IS), rt, flowrate 5 µL/min. [b]Applied voltage, in volt. [c]Steady-state current measured, in ampere. [d]Microfluidic yield of DA product **18**, in percent. [e]Microfluidic yield of Michael product **21**, in percent. [f]Microfluidic yield of oxidation product **24**, in percent. [g]The *ee* value of DA product **18**, in percent.

*EC*$_{50}$= 100 µM

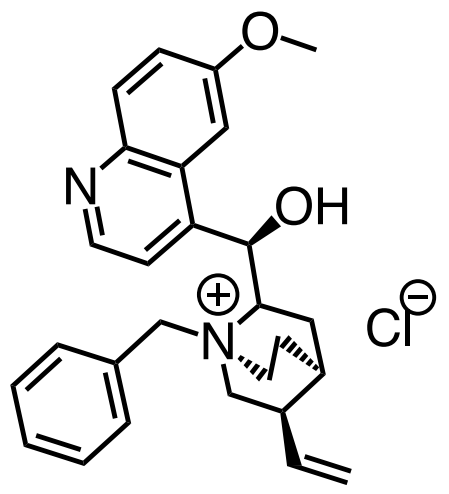

**27**·Cl

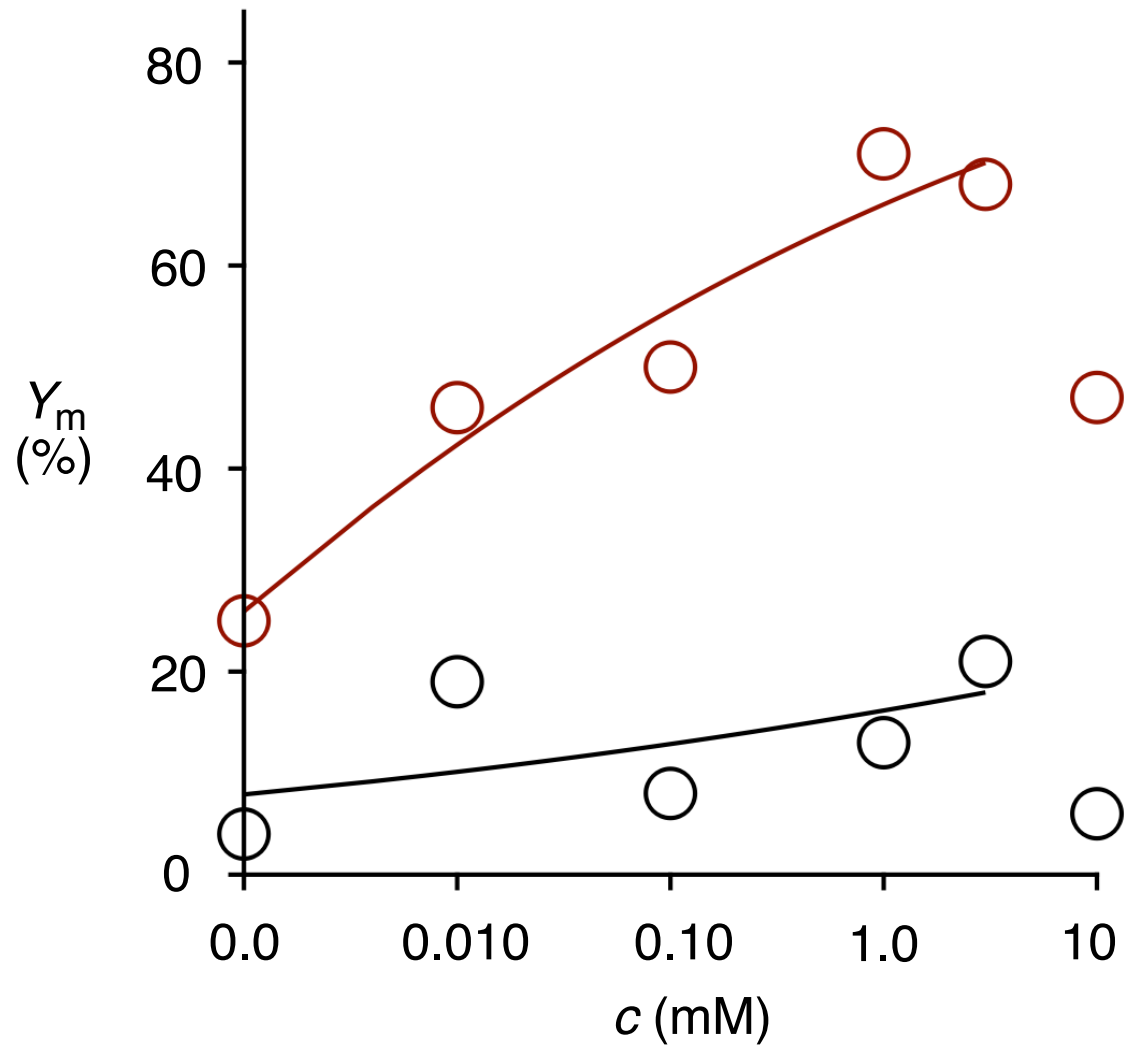


**Figure S18**. Dependence of microfluidic yield $Y_m$ (**18**: circles) on the applied voltage 0.0 and 1.0 V for substrates **14** (50 mM), **15** (75 mM), Helmholtz components **27** and $Cl^-$ (1 mM, 2 mol%) in $CHCl_3$/DMSO 3:1, flow rate 5 µL/min, 100 µm foil, graphite electrodes, rt, with curve fit to Hill equation and indication of $Y_m^{max}$ as maximal microfluidic yield.

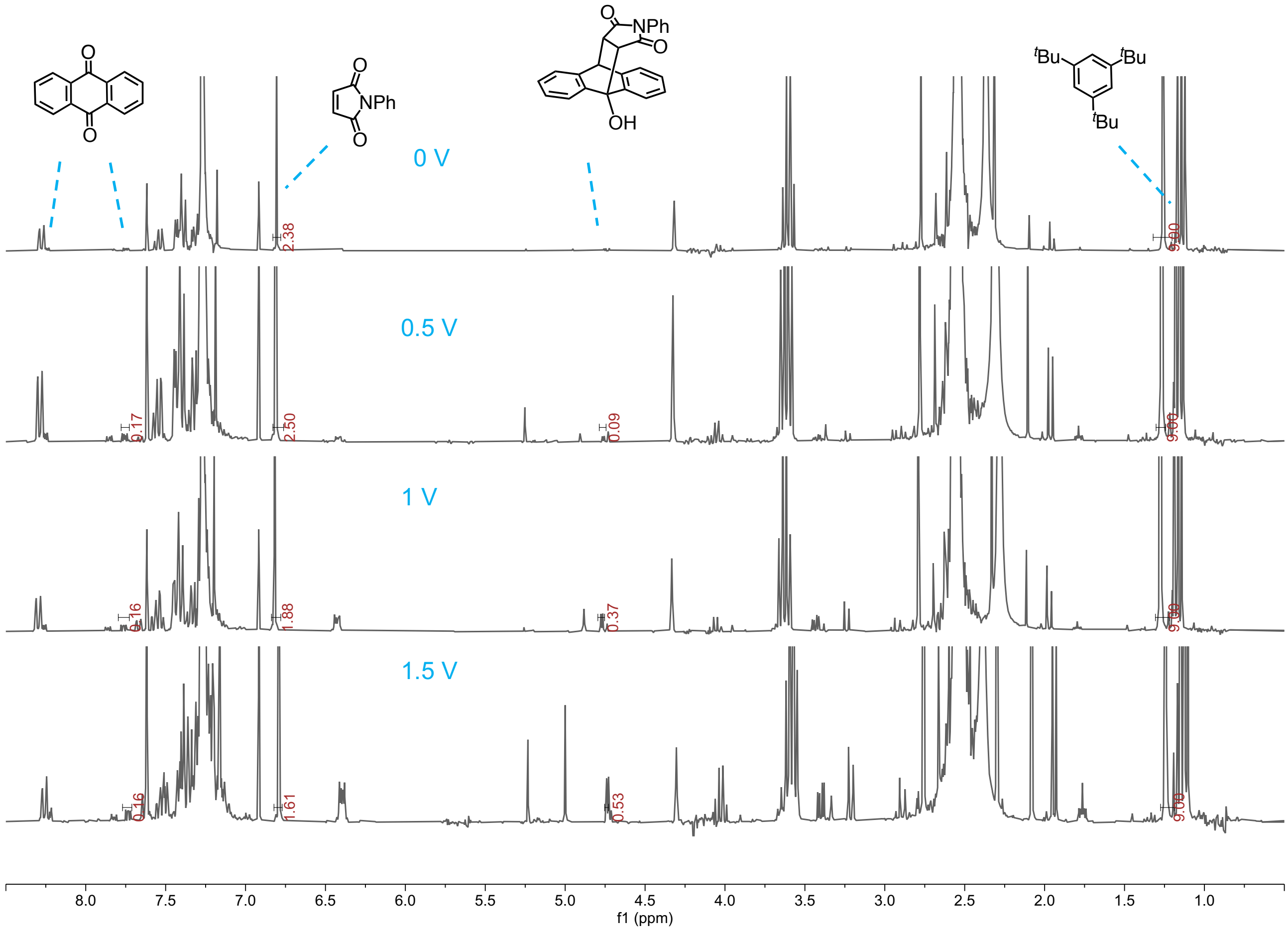


**Figure S19.** $^{1}$H NMR spectra (400 MHz, $CDCl_3$) of reaction mixtures obtained from **14** (50 mM), **17** (75 mM), Helmholtz components **27** and $Cl^-$ (1 mM) in $CHCl_3$/DMSO 3:1 (4 mL) after one passage through the microfluidic capacitor at 5 μL/min with $V$ = 0.0, 0.5, 1.0 and 1.5 V (from top to bottom) increasing voltage applied, at rt.

**Table S8.** EFC with **14** (50 mM), **17** (75 mM) and Helmholtz components **27** and $Cl^-$ (1 mM) in $CHCl_3$/DMSO 3:1.[a]

| Entry | *V* (V)[b] | *I* (A)[c] | $Y_m$**20** (%)[d] | $Y_m$**23** (%)[e] | $Y_m$**24** (%)[f] | *ee* (%)[g] |
|---|---|---|---|---|---|---|
| 1 | 0 | 0.000 | 0 | 0 | 0 | - |
| 2 | 0.5 | 0.000 | 9 | 0 | 4.3 | - |
| 3 | 1.0 | 0.000 | 37 | 0 | 4 | 11 |
| 4 | 1.5 | 0.000 | 53 | 0 | 4 | 10 |

[a]In microfluidic capacitor, flow rate 5 µL/min, 100 µm foil, graphite electrodes, with 1,3,5-tri-*tert*-butylbenzene as internal standard (IS), rt, flowrate 5 µL/min. [b]Applied voltage, in volt. [c]Steady-state current measured, in ampere. [d]Microfluidic yield of DA product **20**, in percent. [e]Microfluidic yield of Michael product **23**, in percent. [f]Microfluidic yield of oxidation product **24**, in percent. [g]The *ee* value of DA product **20**, in percent.

$Y_m^{max}$ = 53%

$V_c$ = 0.5

$n_g$ = 3.0

*ee* = 13%

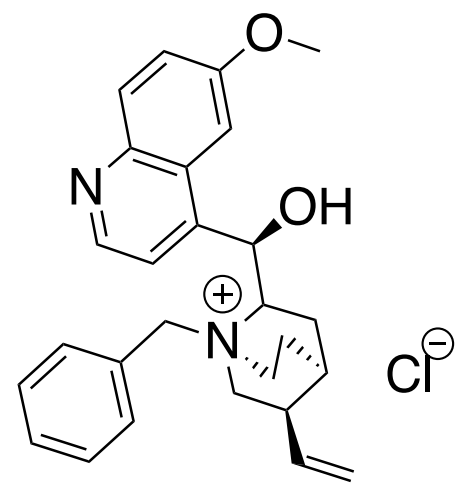


**27**

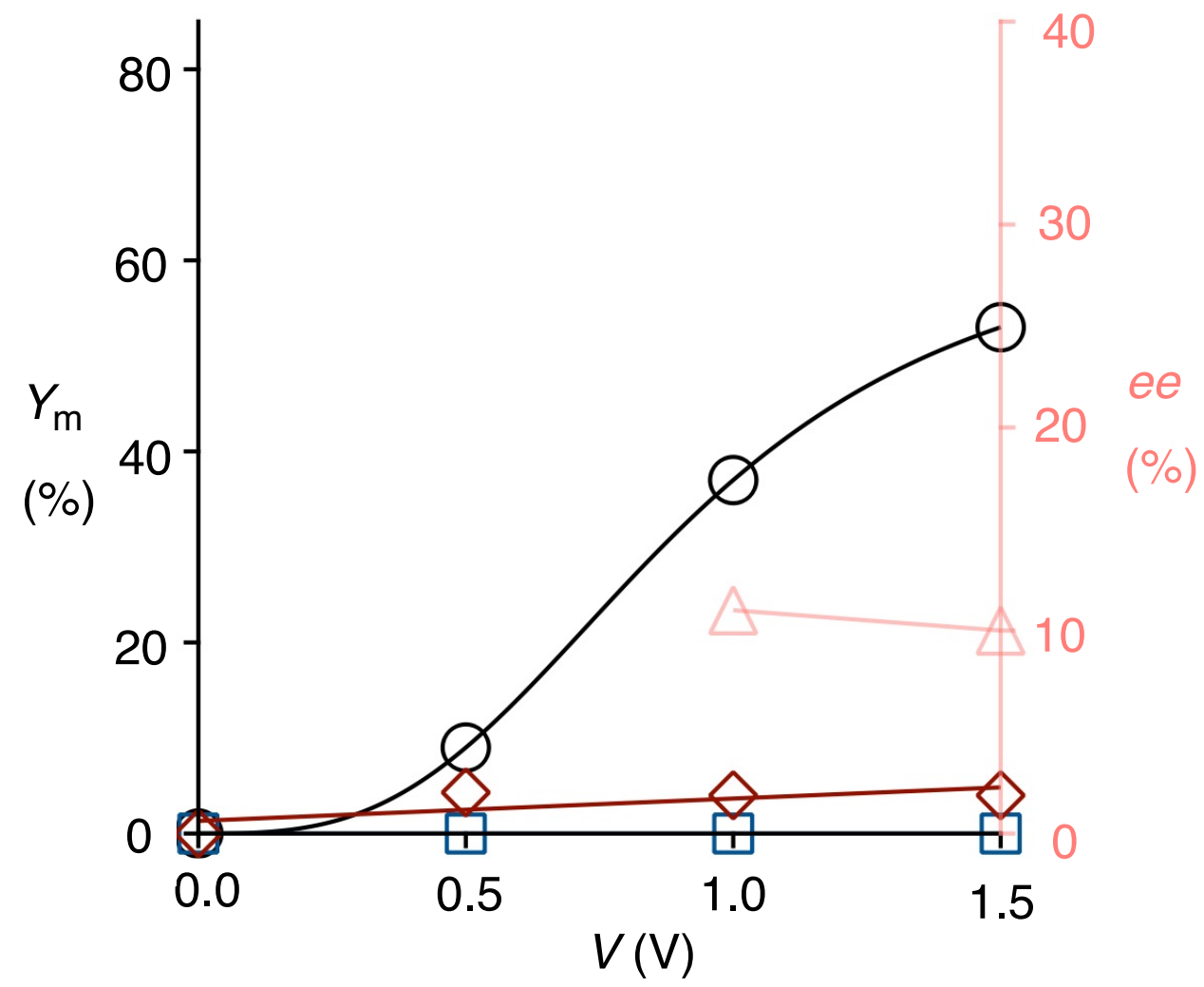


**Figure S20**. Dependence of microfluidic yield $Y_m$ (black, left, **20**: circles, **23**: squares, **24**: diamonds) and *ee* (right, red, **18**: triangles) on the applied voltage *V* for substrates **14** (50 mM), **17** (75 mM) with Helmholtz components **27** and $Cl^-$ (1 mM, 2 mol%) in $CHCl_3$/DMSO 3:1, flow rate 5 µL/min, 100 µm foil, graphite electrodes, rt, with curve fit to Hill equation (circles only) and indication of $Y_m^{max}$ as maximal microfluidic yield, $V_c$ as critical voltage, $n_g$ as gating coefficient, and *ee* as enantioselectivity for **20**.

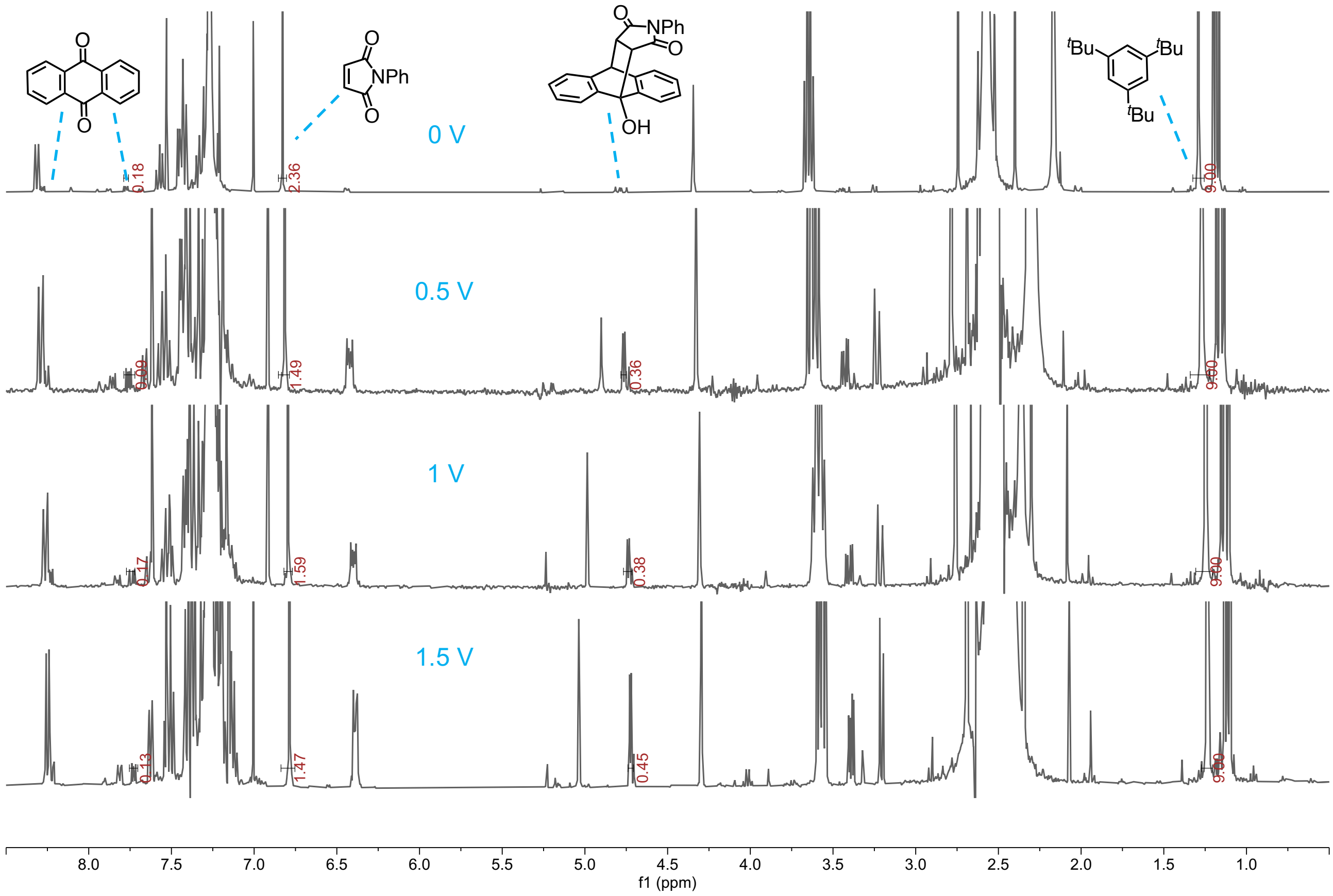


**Figure S21.** $^1$H NMR spectra (400 MHz, $CDCl_3$) of reaction mixtures obtained from **14** (50 mM), **17** (75 mM), Helmholtz components **27** and $Cl^-$ (1 mM) and **28** (2.5 mM) in $CHCl_3$/DMSO 3:1 (4 mL) after one passage through the microfluidic capacitor at 5 µL/min with $V$ = 0.0, 0.5, 1.0 and 1.5 V (from top to bottom) applied, at rt.

**Table S9.** EFC with **14** (50 mM), **17** (75 mM) and Helmholtz components **27** and $Cl^-$ (1 mM) and **28** (2.5 mM) in $CHCl_3$/DMSO 3:1.[a]

| Entry | *V* (V)[b] | *I* (A)[c] | $Y_m^{\mathbf{20}}$ (%)[d] | $Y_m^{\mathbf{23}}$ (%)[e] | $Y_m^{\mathbf{24}}$ (%)[f] | *ee* (%)[g] |
|---|---|---|---|---|---|---|
| 1 | 0 | 0.000 | 0 | 0 | 4.5 | - |
| 2 | 0.5 | 0.000 | 36 | 0 | 2.3 | - |
| 3 | 1.0 | 0.000 | 38 | 0 | 4.3 | - |
| 4 | 1.5 | 0.000 | 45 | 0 | 3.3 | 13 |

[a]In microfluidic capacitor, flow rate 5 µL/min, 100 µm foil, graphite electrodes, with 1,3,5-tri-*tert*-butylbenzene as internal standard (IS), rt, flowrate 5 µL/min. [b]Applied voltage, in volt. [c]Steady-state current measured, in ampere. [d]Microfluidic yield of DA product **20**, in percent. [e]Microfluidic yield of Michael product **23**, in percent. [f]Microfluidic yield of oxidation product **24**, in percent. [g]The *ee* value of DA product **20**, in percent.

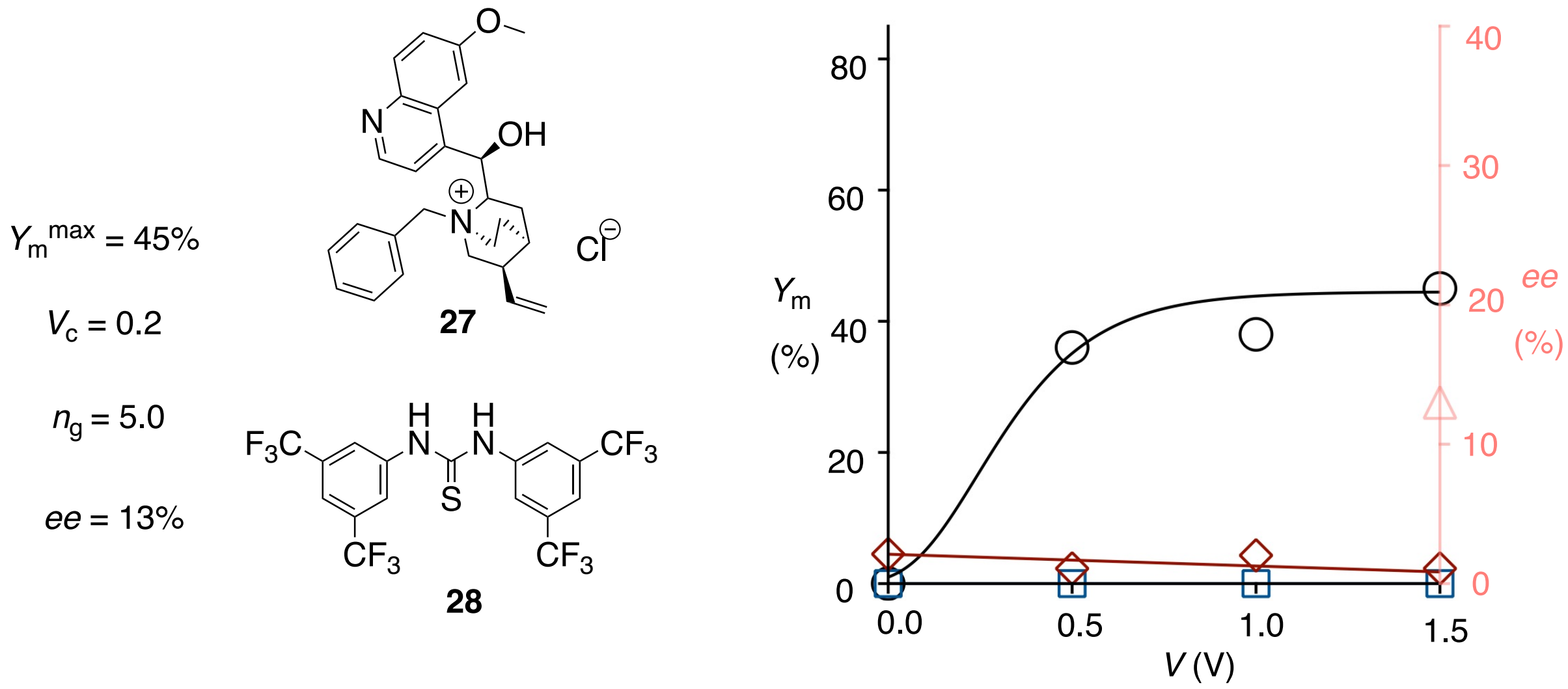


**Figure S22**. Dependence of microfluidic yield $Y_m$ (black, left, **20**: circles, **23**: squares, **24**: diamonds) on the applied voltage $V$ for substrates **14** (50 mM) and **17** (75 mM) in $CHCl_3$/DMSO 3:1, flow rate 5 µL/min, 100 µm foil, graphite electrodes, rt, with curve fit to Hill equation (circles only) and indication of $Y_m^{max}$ as maximal microfluidic yield, $V_c$ as critical voltage, $n_g$ as gating coefficient and *ee* as enantioselectivity for **20**.

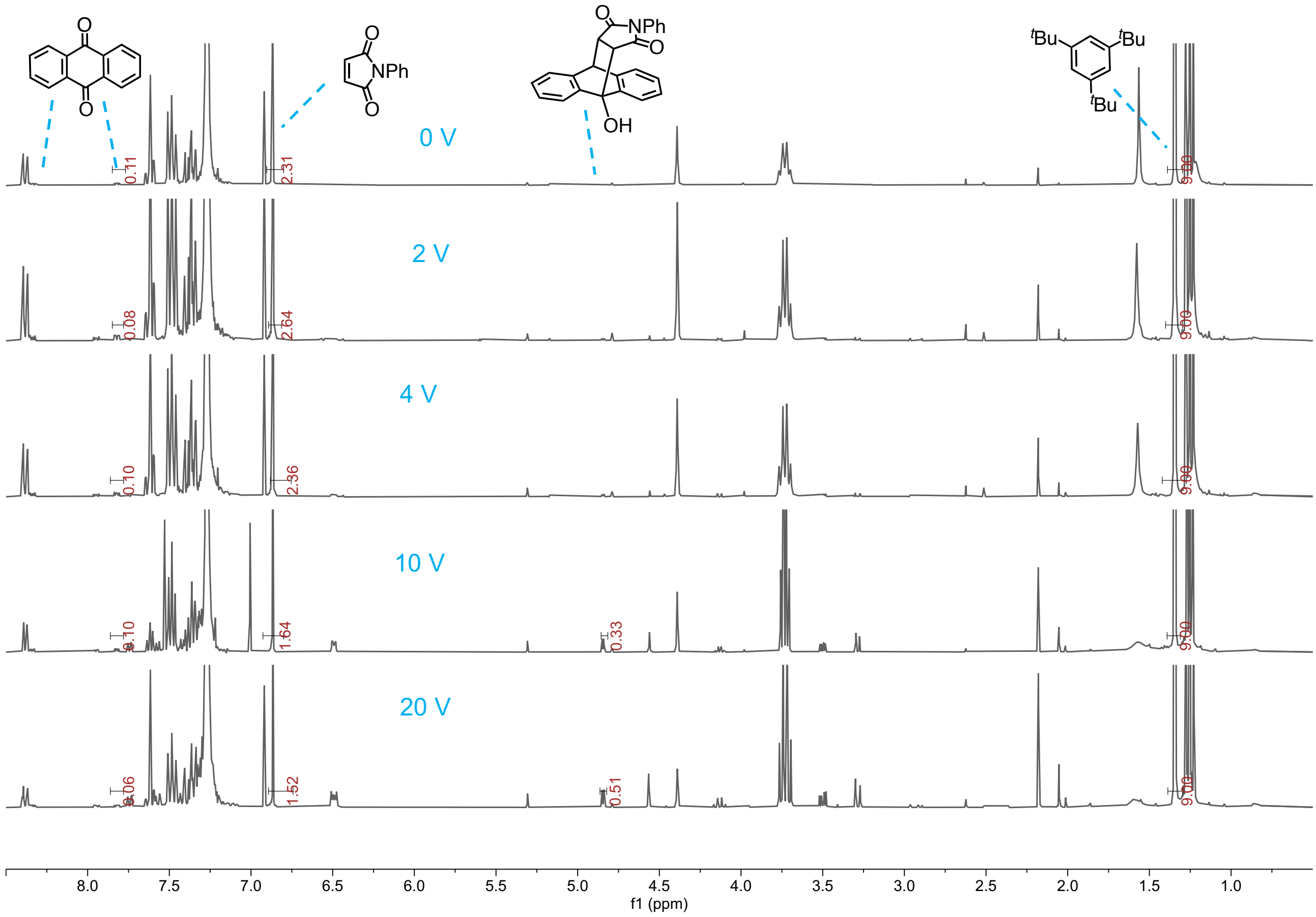


**Figure S23.** $^1$H NMR spectra (400 MHz, $CDCl_3$) of reaction mixtures obtained from **14** (50 mM), **17** (75 mM) and Helmholtz components **27** and $Cl^-$ (1 mM) in $CHCl_3$ (4 mL) after one passage through the microfluidic capacitor at 5 µL/min with $V$ = 0, 2, 4, 10 and 20 V (from top to bottom) applied, at rt.

**Table S10.** EFC with substrates **14** (50 mM), **17** (75 mM) and Helmholtz components **27** and $Cl^-$ in $CHCl_3$.[a]

| Entry | *V* (V)[b] | *I* (A)[c] | $Y_m^{\mathbf{20}}$ (%)[d] | $Y_m^{\mathbf{23}}$ (%)[e] | $Y_m^{\mathbf{24}}$ (%)[f] | *ee* (%)[g] |
|---|---|---|---|---|---|---|
| 1 | 0 | 0.000 | 0 | 0 | 2.8 | - |
| 2 | 2 | 0.000 | 0 | 0 | 2 | - |
| 3 | 4 | 0.000 | 0 | 0 | 2.5 | - |
| 4 | 10 | 0.000 | 33 | 0 | 2.5 | 31 |
| 5 | 20 | 0.000 | 51 | 0 | 1.5 | 28 |

[a]In microfluidic capacitor, flow rate 5 µL/min, 100 µm foil, graphite electrodes, with 1,3,5-tri-*tert*-butylbenzene as internal standard (IS), rt, flowrate 5 µL/min. [b]Applied voltage, in volt. [c]Steady-state current measured, in ampere. [d]Microfluidic yield of DA product **20**, in percent. [e]Microfluidic yield of Michael product **23**, in percent. [f]Microfluidic yield of oxidation product **24**, in percent. [g]The *ee* value of DA product **20**, in percent.

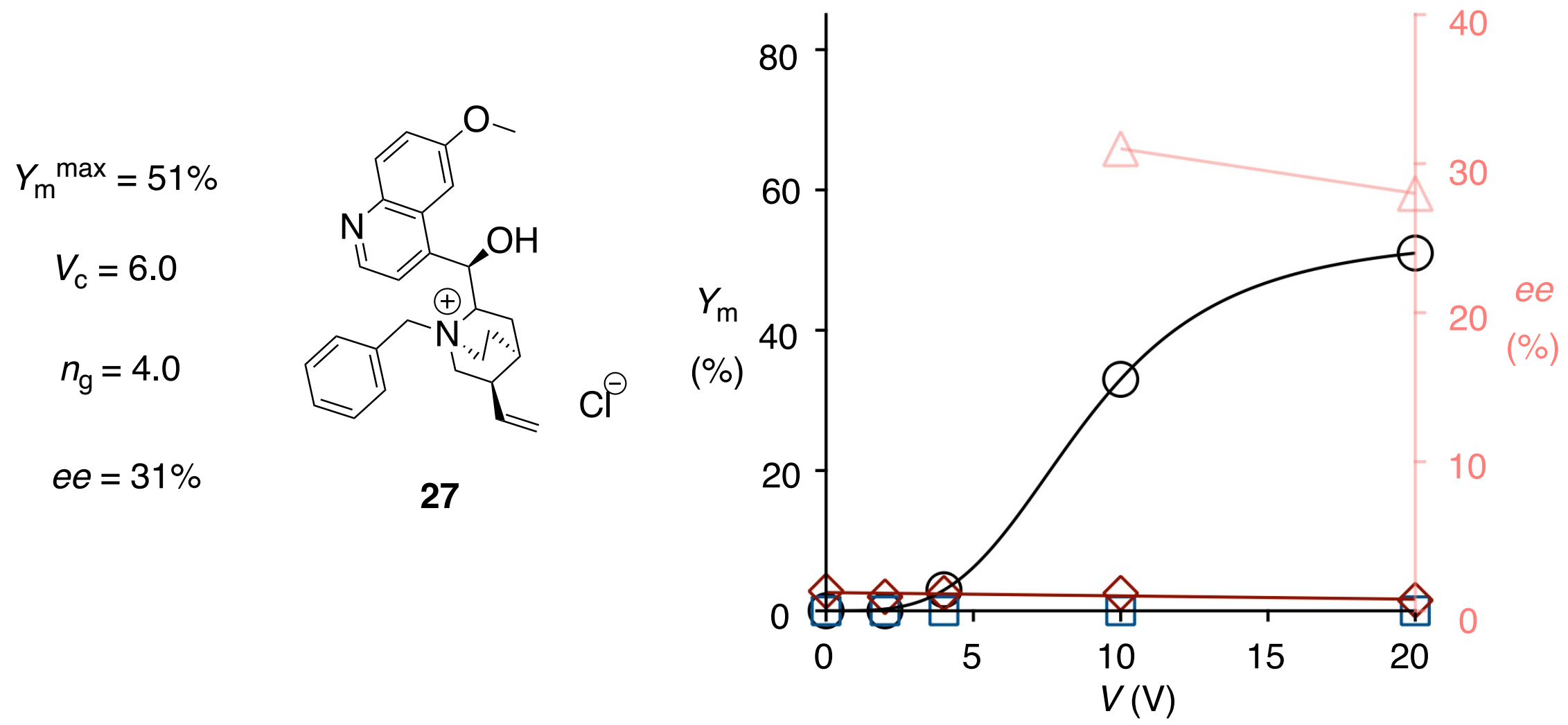


**Figure S24**. Dependence of microfluidic yield $Y_\mathrm{m}$ (black, left, **20**: circles, **23**: squares, **24**: diamonds) and *ee* (right, red, **20**: triangles) on the applied voltage $V$ for substrates **14** (50 mM), **17** (75 mM), Helmholtz components **27** and $Cl^-$ (1 mM, 2 mol%) in $CHCl_3$ (4 mL), flow rate 5 µL/min, 100 µm foil, graphite electrodes, rt, with curve fit to Hill equation (circles only) and indication of $Y_\mathrm{m}^\mathrm{max}$ as maximal microfluidic yield, $V_\mathrm{c}$ as critical voltage, $n_\mathrm{g}$ as gating coefficient and *ee* as enantioselectivity for **20**.

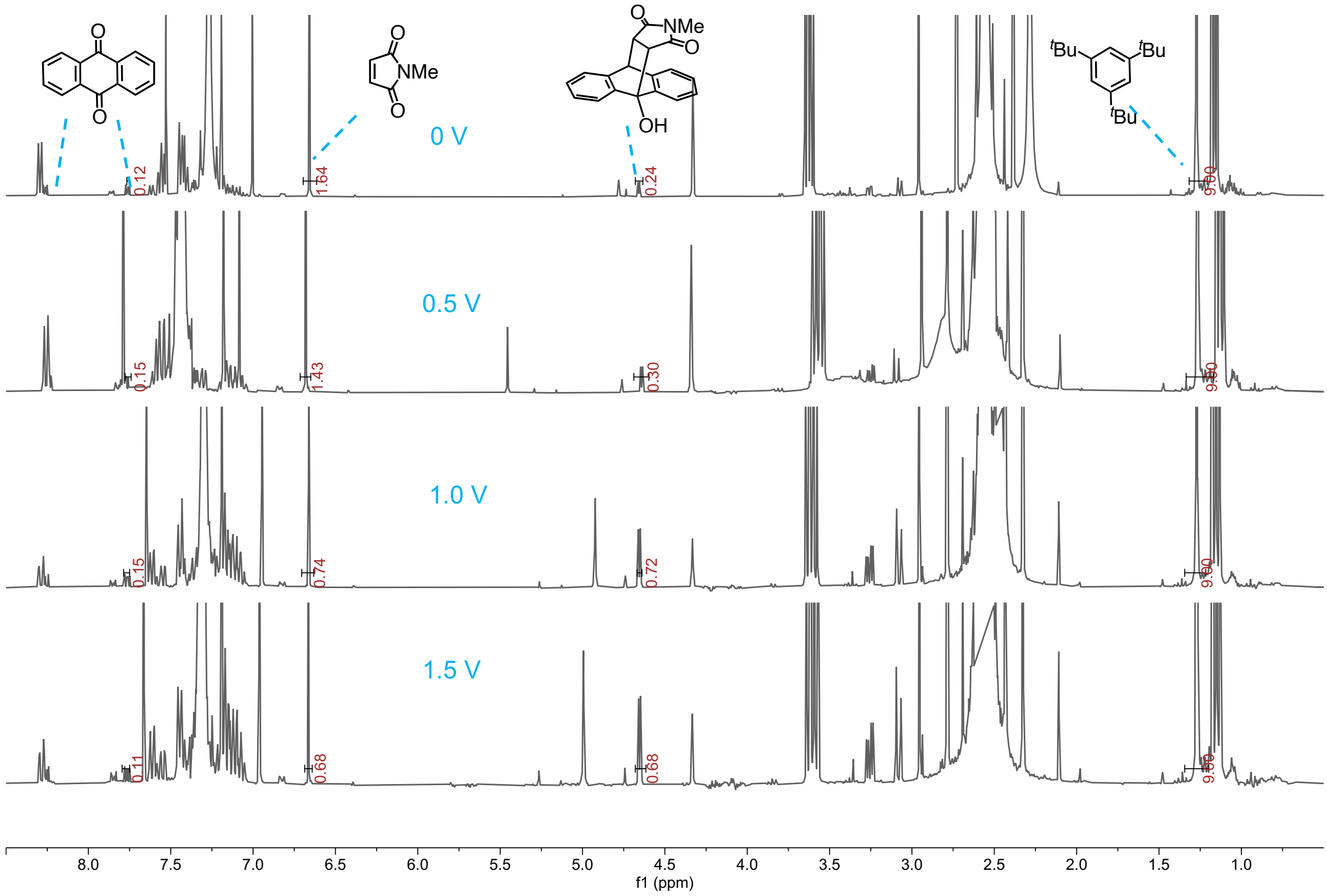


**Figure S25.** $^{1}$H NMR spectra (400 MHz, $CDCl_3$) of reaction mixtures obtained from **14** (50 mM), **15** (75 mM), Helmholtz components **25** (1 mM) and **29** (1 mM) in $CHCl_3$/DMSO 3:1 (4 mL) after one passage through the microfluidic capacitor at 5 µL/min with $V$ = 0.0, 0.5, 1.0 and 1.5 V (from top to bottom) applied, at rt.

**Table S11.** EFC with **14** (50 mM), **15** (75 mM) and Helmholtz components **25** (1 mM) and **29** (1 mM) in $CHCl_3$/DMSO 3:1.[a]

| Entry | *V* (V)[b] | *I* (A)[c] | $Y_m$**18** (%)[d] | $Y_m$**21** (%)[e] | $Y_m$**24** (%)[f] | *ee* (%)[g] |
|---|---|---|---|---|---|---|
| 1 | 0 | 0.000 | 24 | 0 | 3.0 | - |
| 2 | 0.5 | 0.000 | 30 | 0 | 3.8 | - |
| 3 | 1.0 | 0.000 | 72 | 0 | 3.8 | 15 |
| 4 | 1.5 | 0.000 | 68 | 0 | 2.8 | 13 |

[a]In microfluidic capacitor, flow rate 5 µL/min, 100 µm foil, graphite electrodes, with 1,3,5-tri-*tert*-butylbenzene as internal standard (IS), rt, flowrate 5 µL/min. [b]Applied voltage, in volt. [c]Steady-state current measured, in ampere. [d]Microfluidic yield of DA product **18**, in percent. [e]Microfluidic yield of Michael product **21**, in percent. [f]Microfluidic yield of oxidation product **24**, in percent. [g]The *ee* value of DA product **18**, in percent.

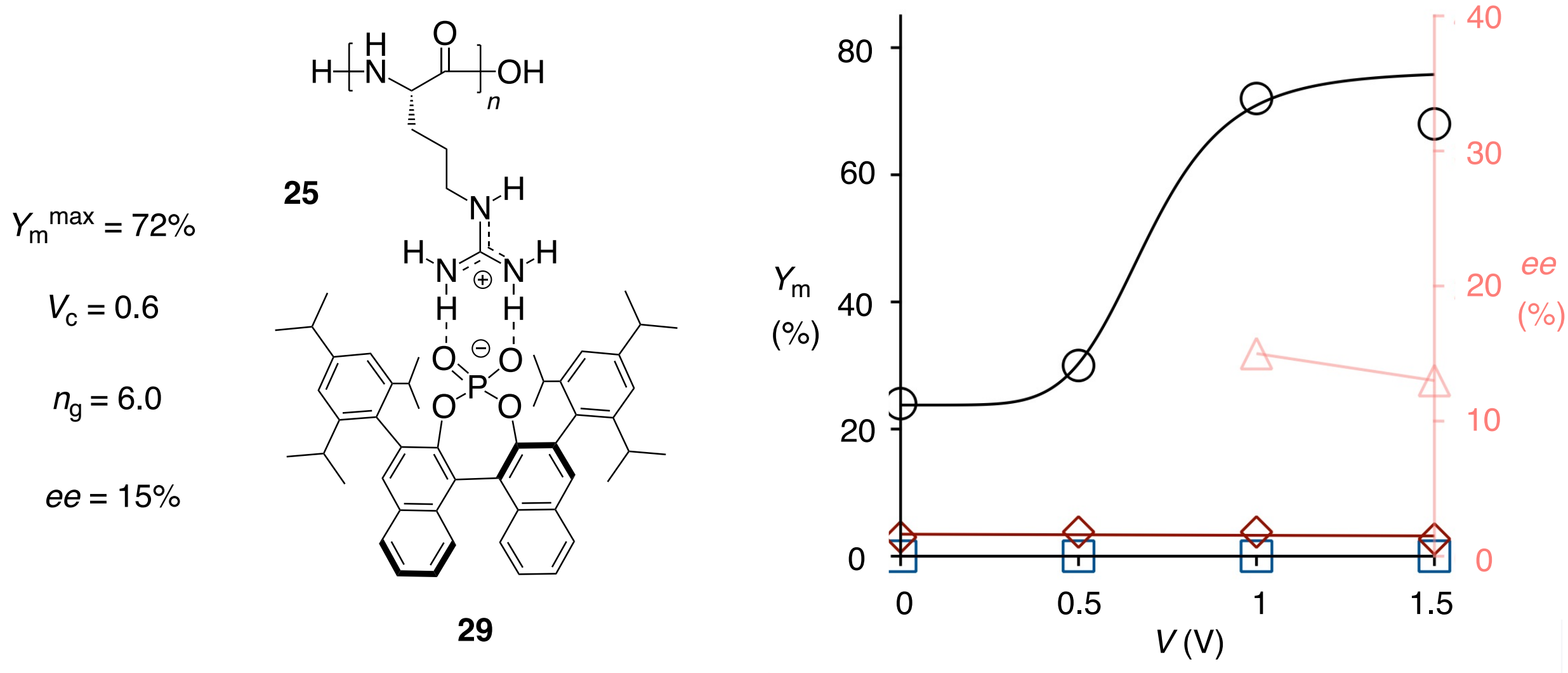


**Figure S26**. Dependence of microfluidic yield $Y_m$ (black, left, **18**: circles, **21**: squares, **24**: diamonds) and *ee* (right, red, **18**: triangles) on the applied voltage $V$ for substrates **14** (50 mM), **15** (75 mM), Helmholtz components **25** and **29** (1 mM, 2 mol%) in $CHCl_3$/DMSO 3:1, flow rate 5 µL/min, 100 µm foil, graphite electrodes, rt, with curve fit to Hill equation (circles only) and indication of $Y_m^{max}$ as maximal microfluidic yield, $V_c$ as critical voltage, $n_g$ as gating coefficient, and *ee* as enantioselectivity for **18**.

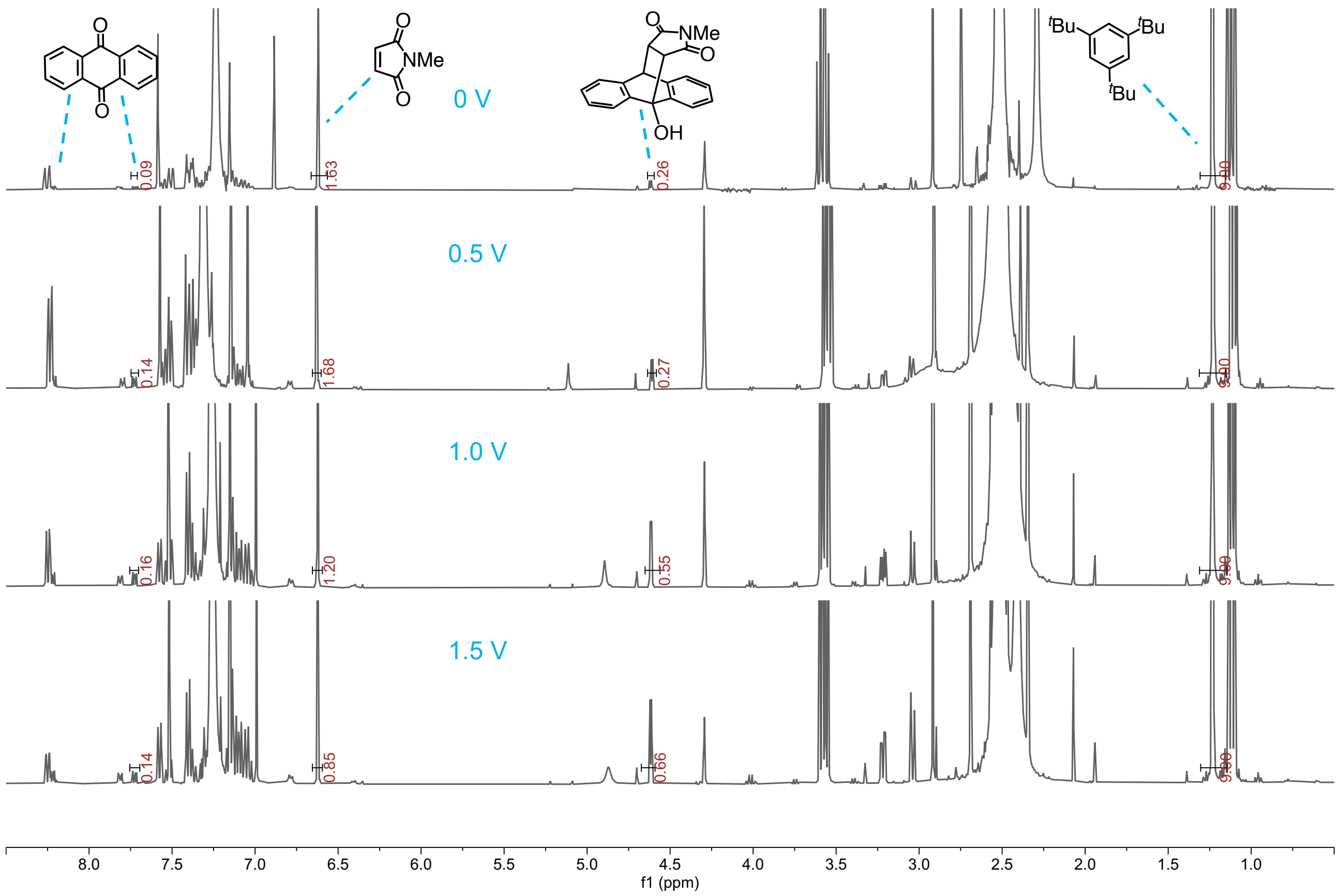


**Figure S27.** $^{1}H$ NMR spectra (400 MHz, $CDCl_3$) of reaction mixtures obtained from **14** (50 mM), **15** (75 mM), **25** (1 mM) and **30** (1 mM) in $CHCl_3$/DMSO 3:1 (4 mL) after one passage through the microfluidic capacitor at 5 µL/min with $V$ = 0.0, 0.5, 1.0 and 1.5 V (from top to bottom) applied, at rt.

**Table S12.** EFC with **14** (50 mM) and **15** (75 mM) and Helmholtz components **25** (1 mM) and **30** (1 mM) in $CHCl_3$/DMSO 3:1.[a]

| Entry | $V$ (V)[b] | $I$ (A)[c] | $Y_m^{\mathbf{18}}$ (%)[d] | $Y_m^{\mathbf{21}}$ (%)[e] | $Y_m^{\mathbf{24}}$ (%)[f] | *ee* (%)[g] |
|---|---|---|---|---|---|---|
| 1 | 0 | 0.000 | 26 | 0 | 2.5 | - |
| 2 | 0.5 | 0.000 | 27 | 0 | 3.5 | 10 |
| 3 | 1.0 | 0.000 | 55 | 0 | 4.0 | 15 |
| 4 | 1.5 | 0.000 | 66 | 0 | 3.5 | 8 |

[a]In microfluidic capacitor, flow rate 5 µL/min, 100 µm foil, graphite electrodes, with 1,3,5-tri-*tert*-butylbenzene as internal standard (IS), rt, flowrate 5 µL/min. [b]Applied voltage, in volt. [c]Steady-state current measured, in ampere. [d]Microfluidic yield of DA product **18**, in percent. [e]Microfluidic yield of Michael product **21**, in percent. [f]Microfluidic yield of oxidation product **24**, in percent. [g]The *ee* value of DA product **18**, in percent.

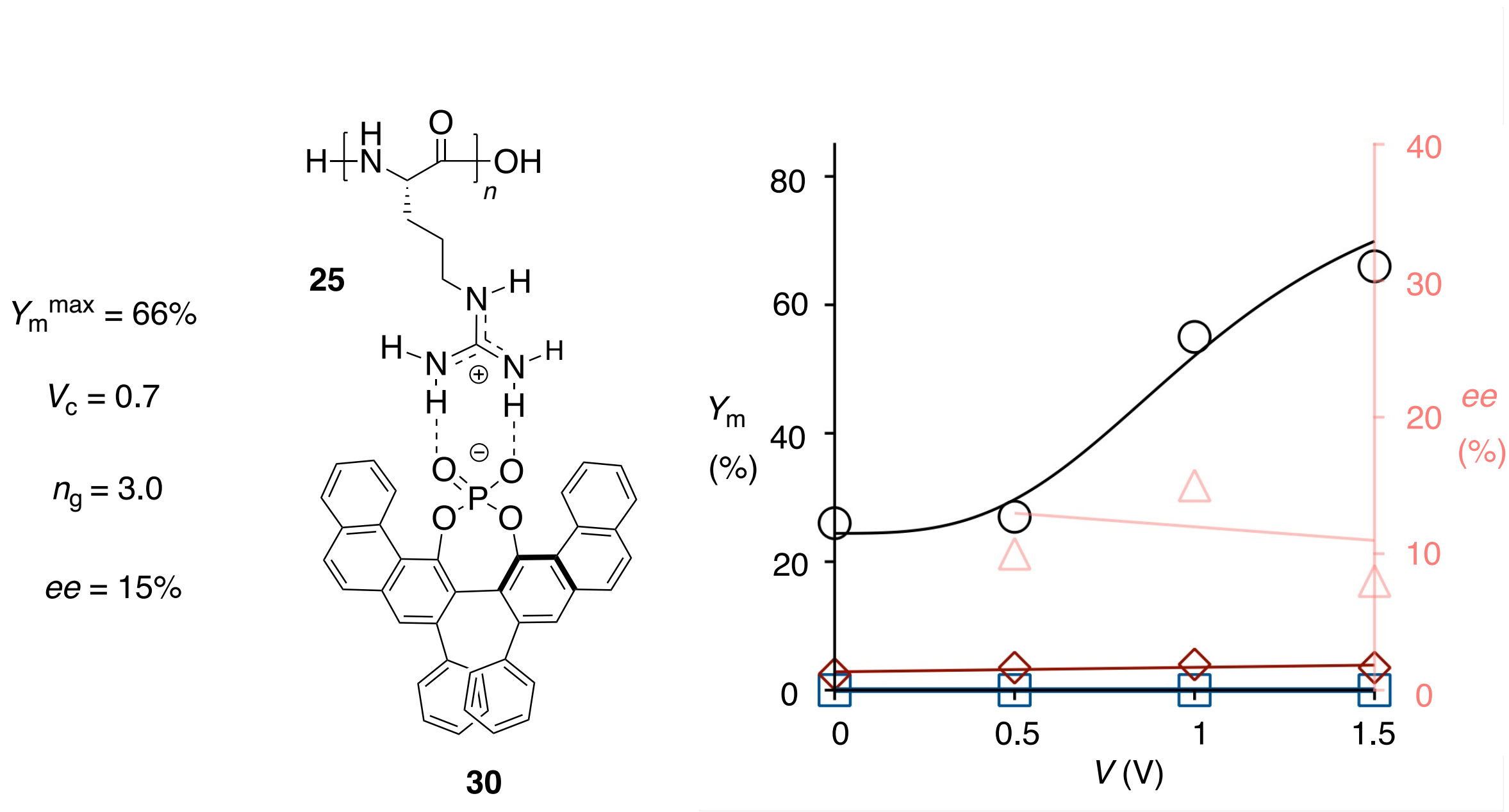


**Figure S28.** Dependence of microfluidic yield $Y_m$ (black, left, **18**: circles, **21**: squares, **24**: diamonds) and *ee* (right, red, **18**: triangles) on the applied voltage $V$ for substrates **14** (50 mM) and **15** (75 mM) with Helmholtz components **25** and **30** (1 mM, 2 mol%) in $CHCl_3$/DMSO 3:1, flow rate 5 µL/min, 100 µm foil, graphite electrodes, rt, with curve fit to Hill equation (circles only) and indication of $Y_m^{max}$ as maximal microfluidic yield, $V_c$ as critical voltage, $n_g$ as gating coefficient, and *ee* as enantioselectivity for **18**.

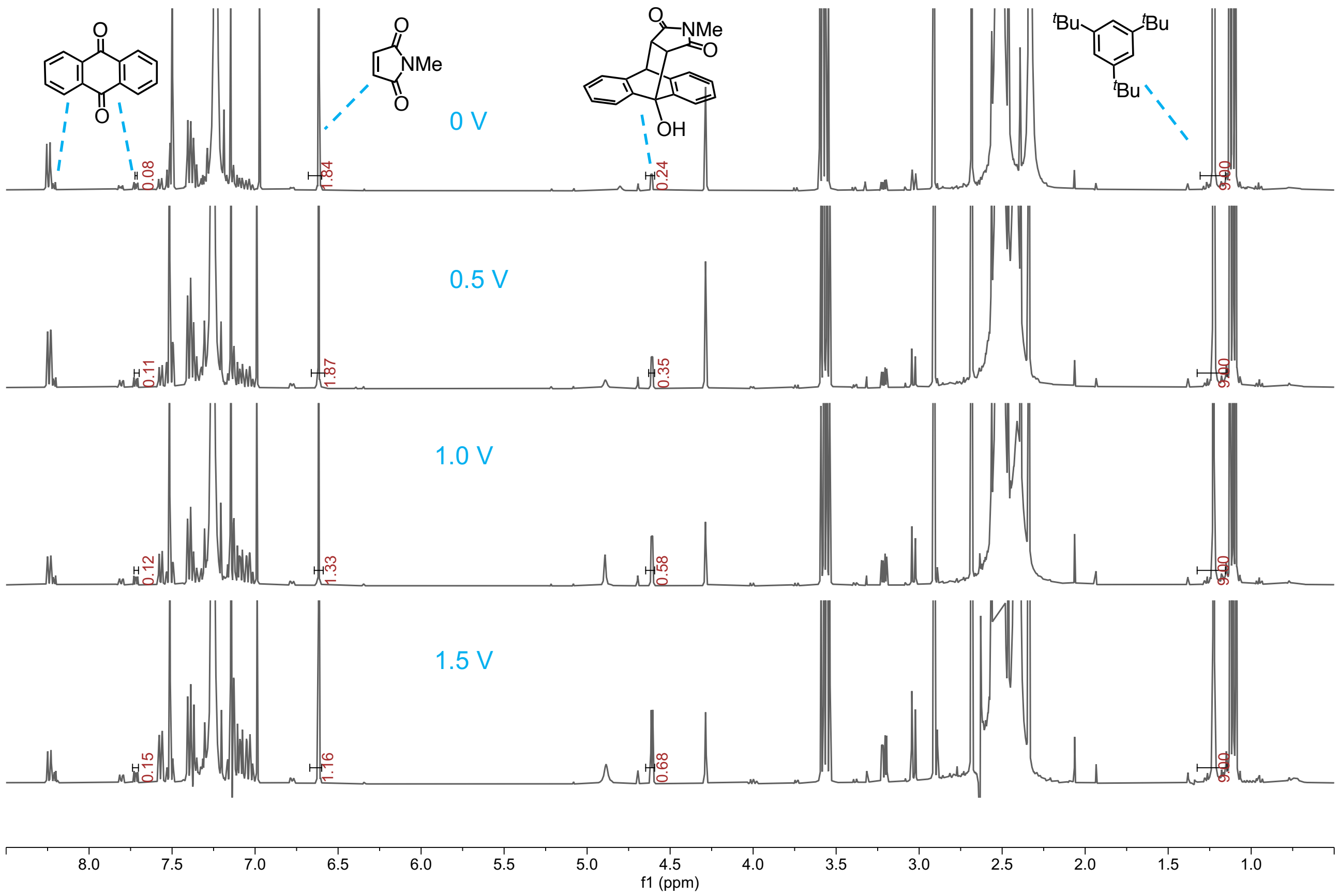


**Figure S29.** $^{1}$H NMR spectra (400 MHz, $CDCl_3$) of reaction mixtures obtained from **14** (50 mM), **15** (75 mM), **25** (1 mM) and **31** (1 mM) in $CHCl_3$/DMSO 3:1 (4 mL) after one passage through the microfluidic capacitor at 5 µL/min with $V$ = 0.0, 0.5, 1.0 and 1.5 V (from top to bottom) applied, at rt.

**Table S13.** EFC with **14** (50 mM) and **15** (75 mM) and Helmholtz components **25** (1 mM) and **31** (1 mM) in $CHCl_3$/DMSO 3:1.[a]

| Entry | *V* (V)[b] | *I* (A)[c] | $Y_m^{\mathbf{18}}$ (%)[d] | $Y_m^{\mathbf{21}}$ (%)[e] | $Y_m^{\mathbf{24}}$ (%)[f] | *ee* (%)[g] |
|---|---|---|---|---|---|---|
| 1 | 0 | 0.000 | 24 | 0 | 2.0 | - |
| 2 | 0.5 | 0.000 | 35 | 0 | 2.8 | 4 |
| 3 | 1.0 | 0.000 | 58 | 0 | 3.0 | 7 |
| 4 | 1.5 | 0.000 | 68 | 0 | 3.8 | 7 |

[a]In microfluidic capacitor, flow rate 5 µL/min, 100 µm foil, graphite electrodes, with 1,3,5-tri-*tert*-butylbenzene as internal standard (IS), rt, flowrate 5 µL/min. [b]Applied voltage, in volt. [c]Steady-state current measured, in ampere. [d]Microfluidic yield of DA product **18**, in percent. [e]Microfluidic yield of Michael product **21**, in percent. [f]Microfluidic yield of oxidation product **24**, in percent. [g]The *ee* value of DA product **18**, in percent.

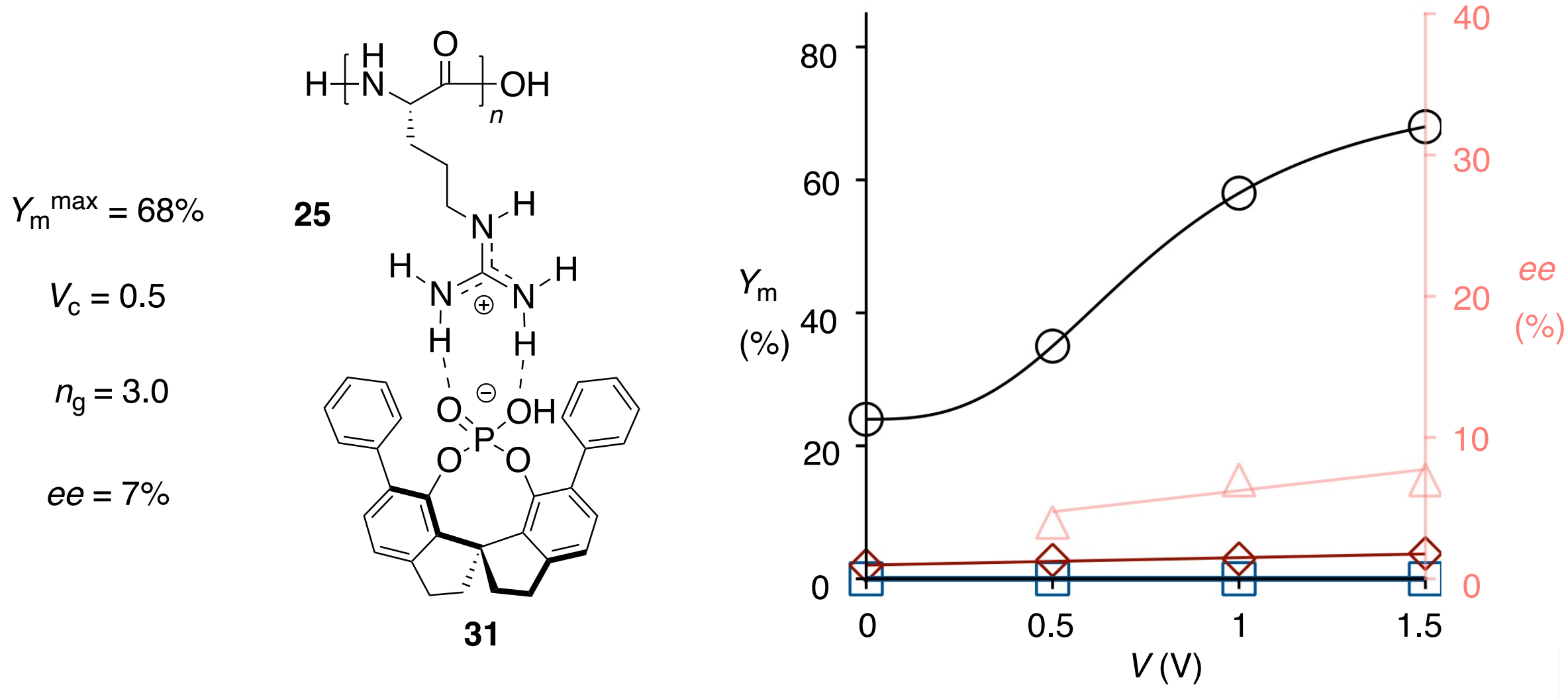


**Figure S30.** Dependence of microfluidic yield $Y_m$ (black, left, **18**: circles, **21**: squares, **24**: diamonds) and *ee* (right, red, **18**: triangles) on the applied voltage $V$ for substrates **14** (50 mM) and **15** (75 mM) with Helmholtz components **25** and **31** (1 mM, 2 mol%) in $CHCl_3$/DMSO 3:1, flow rate 5 µL/min, 100 µm foil, graphite electrodes, rt, with curve fit to Hill equation (circles only) and indication of $Y_m^{max}$ as maximal microfluidic yield, $V_c$ as critical voltage, $n_g$ as gating coefficient, and *ee* as enantioselectivity for **18**.

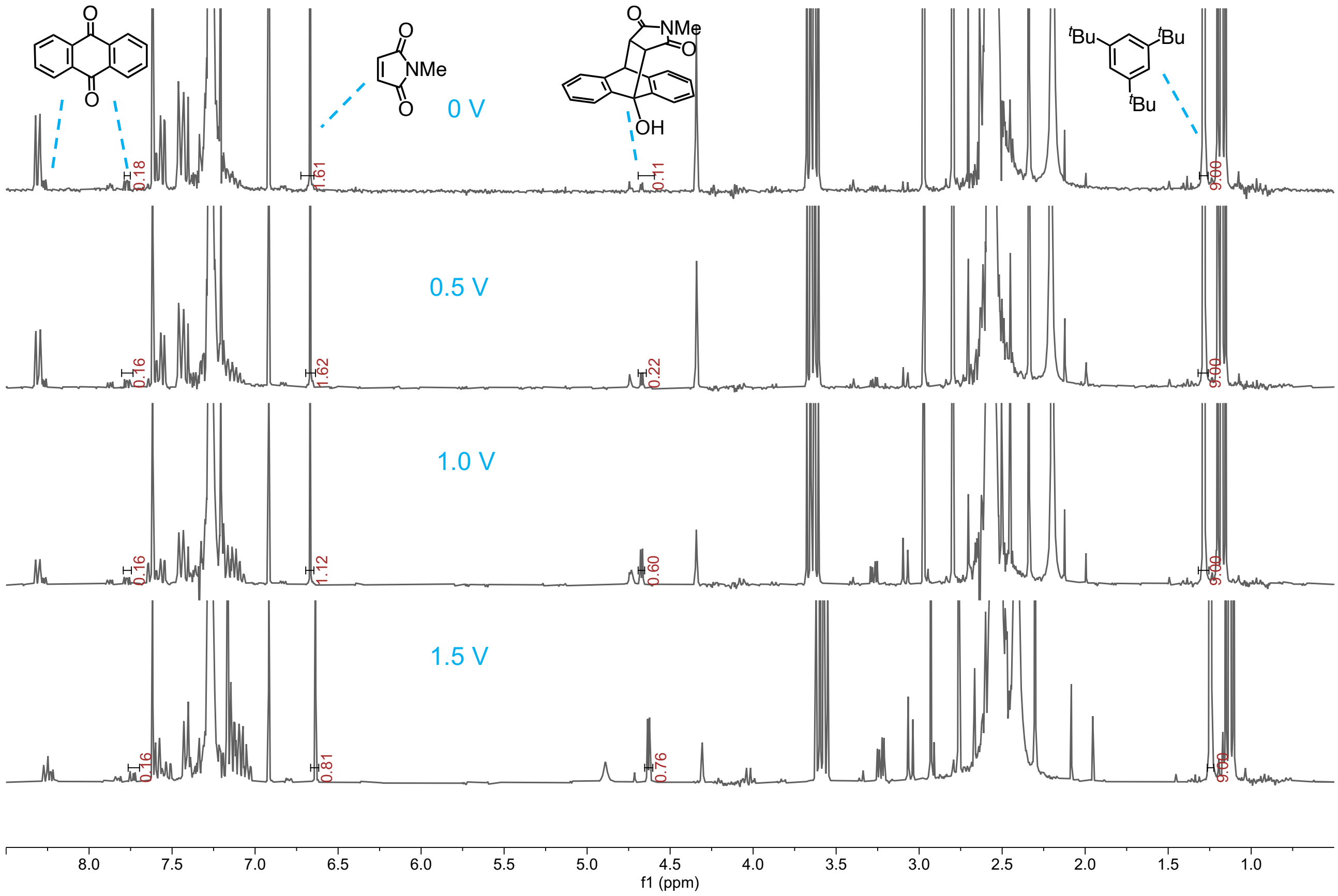


**Figure S31.** $^{1}$H NMR spectra (400 MHz, $CDCl_3$) of reaction mixtures obtained from **14** (50 mM), **15** (75 mM), Helmholtz components **25** (1 mM) and **32** (1 mM) in $CHCl_3$/DMSO 3:1 (4 mL) after one passage through the microfluidic capacitor at 5 µL/min with $V$ = 0.0, 0.5, 1.0 and 1.5 V (from top to bottom) applied, at rt.

**Table S14.** EFC with **14** (50 mM) and **15** (75 mM) and Helmholtz components **25** (1 mM) and **32** (1 mM) in $CHCl_3$/DMSO 3:1.[a]

| Entry | *V* (V)[b] | *I* (A)[c] | $Y_m^{\mathbf{18}}$ (%)[d] | $Y_m^{\mathbf{21}}$ (%)[e] | $Y_m^{\mathbf{24}}$ (%)[f] | *ee* (%)[g] |
|---|---|---|---|---|---|---|
| 1 | 0 | 0.000 | 11 | 0 | 4.0 | - |
| 2 | 0.5 | 0.000 | 22 | 0 | 4.0 | 0 |
| 3 | 1.0 | 0.000 | 60 | 0 | 4.0 | 0 |
| 4 | 1.5 | 0.000 | 76 | 0 | 4.0 | 0 |

[a]In microfluidic capacitor, flow rate 5 µL/min, 100 µm foil, graphite electrodes, with 1,3,5-tri-*tert*-butylbenzene as internal standard (IS), rt, flowrate 5 µL/min. [b]Applied voltage, in volt. [c]Steady-state current measured, in ampere. [d]Microfluidic yield of DA product **18**, in percent. [e]Microfluidic yield of Michael product **21**, in percent. [f]Microfluidic yield of oxidation product **24**, in percent. [g]The *ee* value of DA product **18**, in percent.

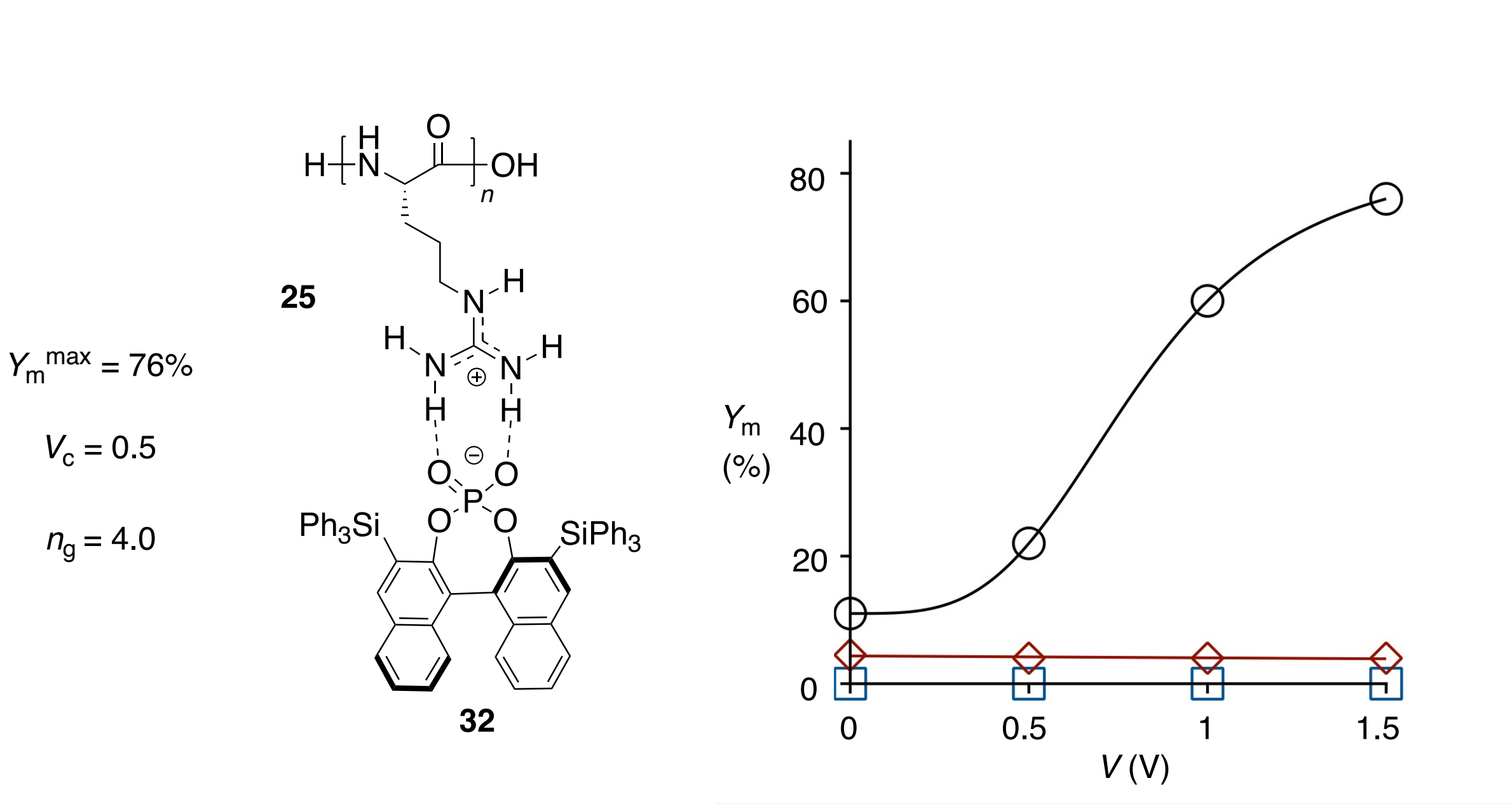


**Figure S32**. Dependence of microfluidic yield $Y_m$ (black, left, **18**: circles, **21**: squares, **24**: diamonds) on the applied voltage $V$ for substrates **14** (50 mM) and **15** (75 mM) with Helmholtz components **25** (1 mM) and **32** (1 mM) in $CHCl_3$/DMSO 3:1, flow rate 5 µL/min, 100 µm foil, graphite electrodes, rt, with curve fit to Hill equation (circles only) and indication of $Y_m^{max}$ as maximal microfluidic yield, $V_c$ as critical voltage, $n_g$ as gating coefficient.

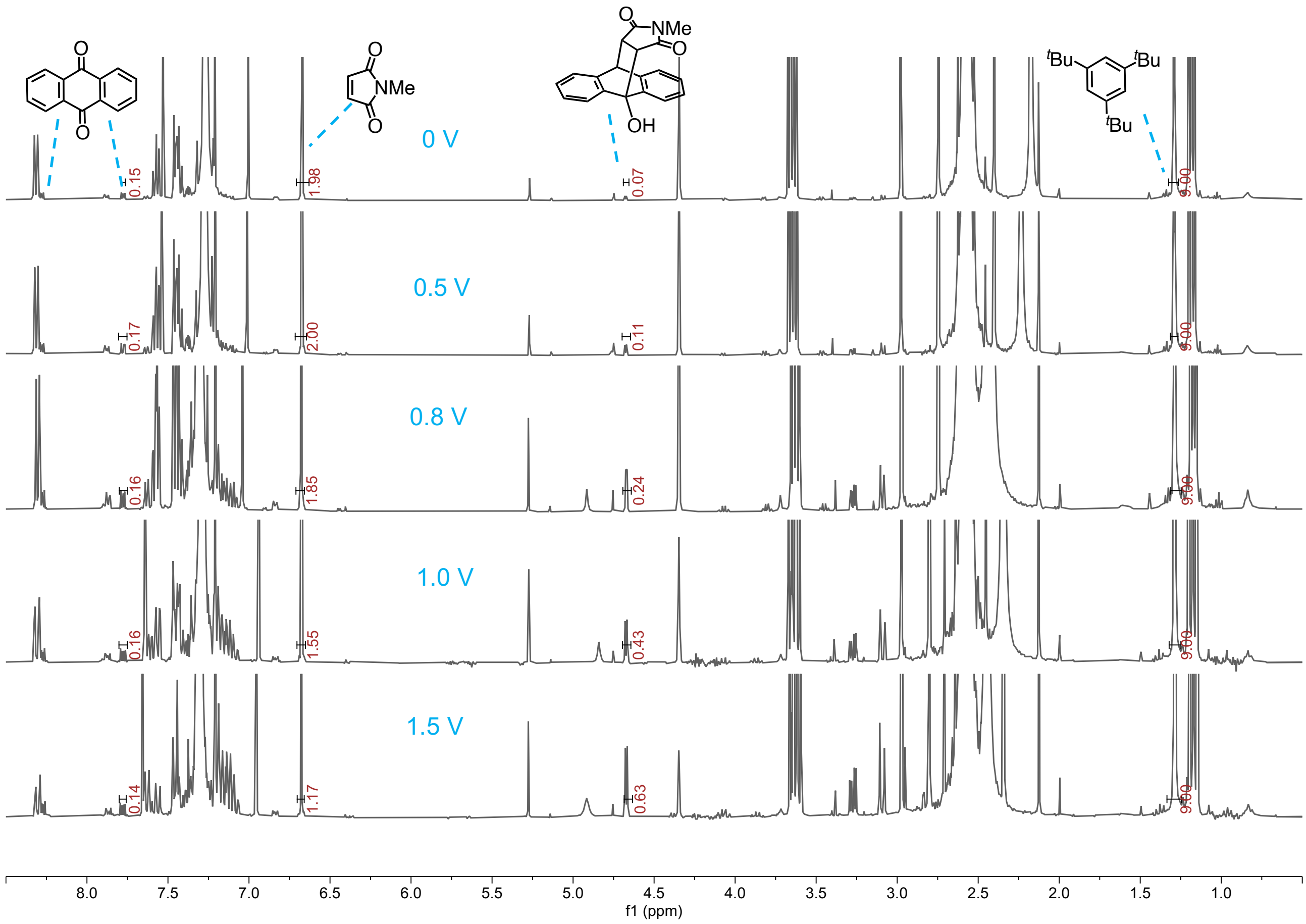


**Figure S33.** $^{1}$H NMR spectra (400 MHz, $CDCl_3$) of reaction mixtures obtained from **14** (50 mM), **15** (75 mM), **25** (1 mM) and **33** (1 mM) in $CHCl_3$/DMSO 3:1 (4 mL) after one passage through the microfluidic capacitor at 5 µL/min with $V$ = 0.0, 0.5, 0.8, 1.0 and 1.5 V (from top to bottom) applied, at rt.

**Table S15.** EFC with **14** (50 mM) and **15** (75 mM) and Helmholtz components **25** (1 mM) and **33** (1 mM) in $CHCl_3$/DMSO 3:1.[a]

| Entry | *V* (V)[b] | *I* (A)[c] | $Y_m^{\mathbf{18}}$ (%)[d] | $Y_m^{\mathbf{21}}$ (%)[e] | $Y_m^{\mathbf{24}}$ (%)[f] | *ee* (%)[g] |
|---|---|---|---|---|---|---|
| 1 | 0 | 0.000 | 7 | 0 | 3.7 | - |
| 2 | 0.5 | 0.000 | 11 | 0 | 4.3 | - |
| 3 | 0.8 | 0.000 | 24 | 0 | 4.0 | - |
| 4 | 1.0 | 0.000 | 43 | 0 | 4.0 | - |
| 5 | 1.5 | 0.000 | 63 | 0 | 3.5 | 0 |

[a]In microfluidic capacitor, flow rate 5 µL/min, 100 µm foil, graphite electrodes, with 1,3,5-tri-*tert*-butylbenzene as internal standard (IS), rt, flowrate 5 µL/min. [b]Applied voltage, in volt. [c]Steady-state current measured, in ampere. [d]Microfluidic yield of DA product **18**, in percent. [e]Microfluidic yield of Michael product **21**, in percent. [f]Microfluidic yield of oxidation product **24**, in percent. [g]The *ee* value of DA product **18**, in percent.

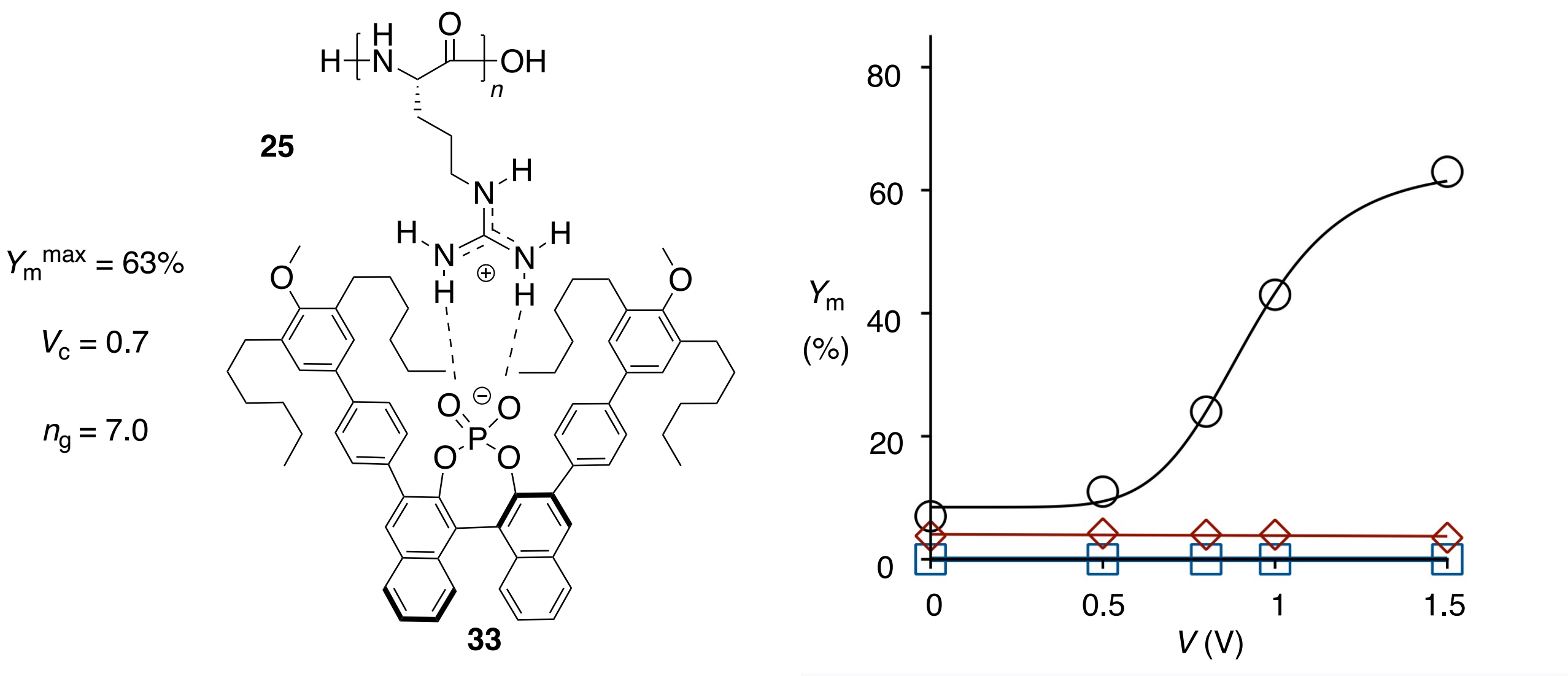


**Figure S34**. Dependence of microfluidic yield $Y_m$ (**18**: circles, **21**: squares, **24**: diamonds) on the applied voltage $V$ for substrates **14** (50 mM) and **15** (75 mM) with Helmholtz components **25** (1 mM) and **33** (1 mM)in $CHCl_3$/DMSO 3:1, flow rate 5 µL/min, 100 µm foil, graphite electrodes, rt, with curve fit to Hill equation (circles only) and indication of $Y_m^{max}$ as maximal microfluidic yield, $V_c$ as critical voltage, $n_g$ as gating coefficient.

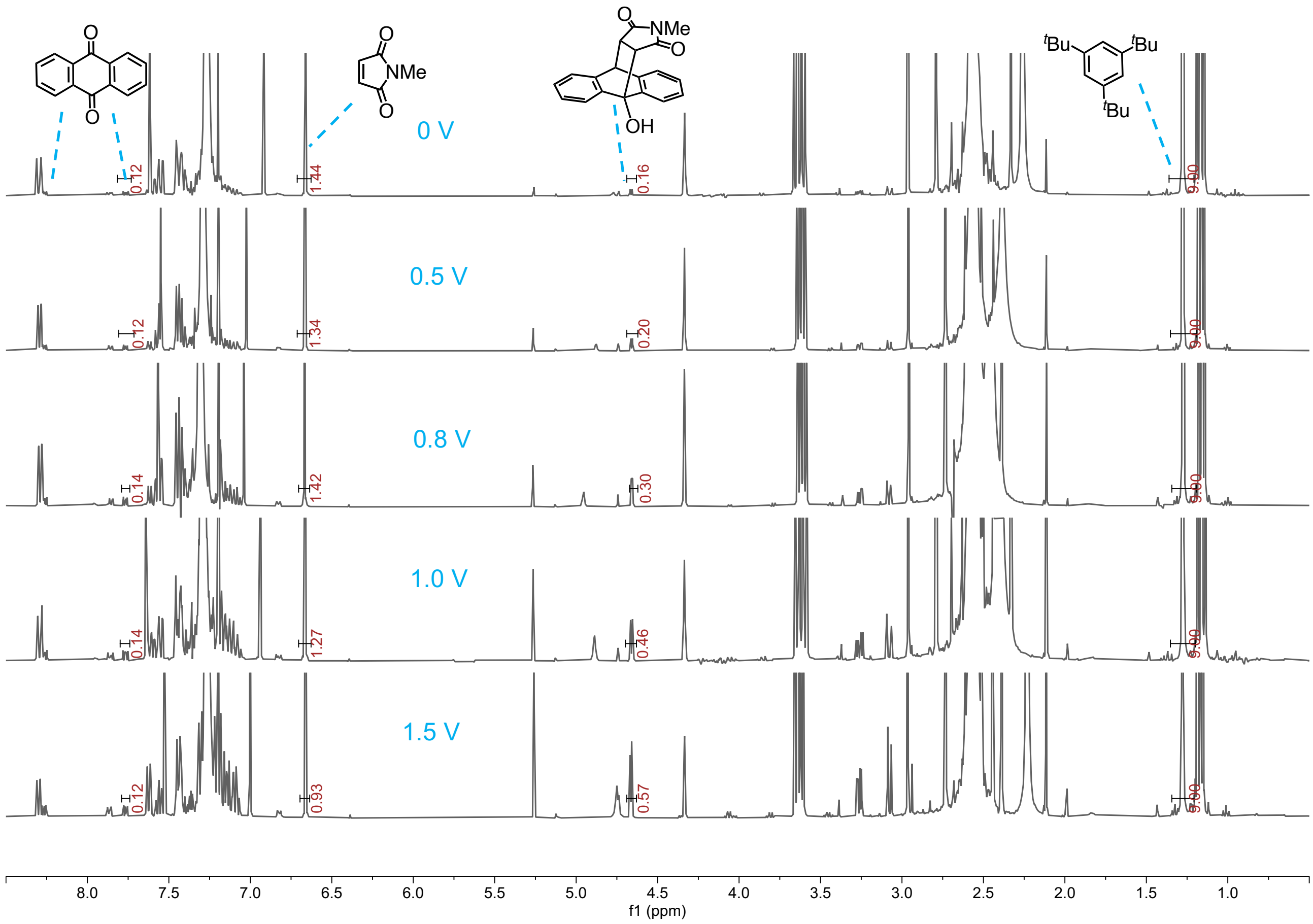


**Figure S35.** $^1$H NMR spectra (400 MHz, $CDCl_3$) of reaction mixtures obtained from **14** (50 mM), **15** (75 mM), **25** (1 mM) and **34** (1 mM) in $CHCl_3$/DMSO 3:1 (4 mL) after one passage through the microfluidic capacitor at 5 µL/min with $V$ = 0.0, 0.5, 0.8, 1.0 and 1.5 V (from top to bottom) applied, at rt.

**Table S16.** EFC with **14** (50 mM) and **15** (75 mM) and Helmholtz components **25** (1 mM) and **34** (1 mM) in $CHCl_3$/DMSO 3:1.[a]

| Entry | *V* (V)[b] | *I* (A)[c] | $Y_m^{\mathbf{18}}$ (%)[d] | $Y_m^{\mathbf{21}}$ (%)[e] | $Y_m^{\mathbf{24}}$ (%)[f] | *ee* (%)[g] |
|---|---|---|---|---|---|---|
| 1 | 0 | 0.000 | 16 | 0 | 3.0 | - |
| 2 | 0.5 | 0.000 | 20 | 0 | 3.0 | - |
| 3 | 0.8 | 0.000 | 30 | 0 | 3.5 | - |
| 4 | 1.0 | 0.000 | 46 | 0 | 3.5 | - |
| 5 | 1.5 | 0.000 | 57 | 0 | 3.0 | 0 |

[a]In microfluidic capacitor, flow rate 5 µL/min, 100 µm foil, graphite electrodes, with 1,3,5-tri-*tert*-butylbenzene  as internal standard (IS), rt, flowrate 5 µL/min. [b]Applied voltage, in volt. [c]Steady-state current measured, in ampere. [d]Microfluidic yield of DA product **18**, in percent. [e]Microfluidic yield of Michael product **21**, in percent. [f]Microfluidic yield of oxidation product **24**, in percent. [g]The *ee* value of DA product **18**, in percent.

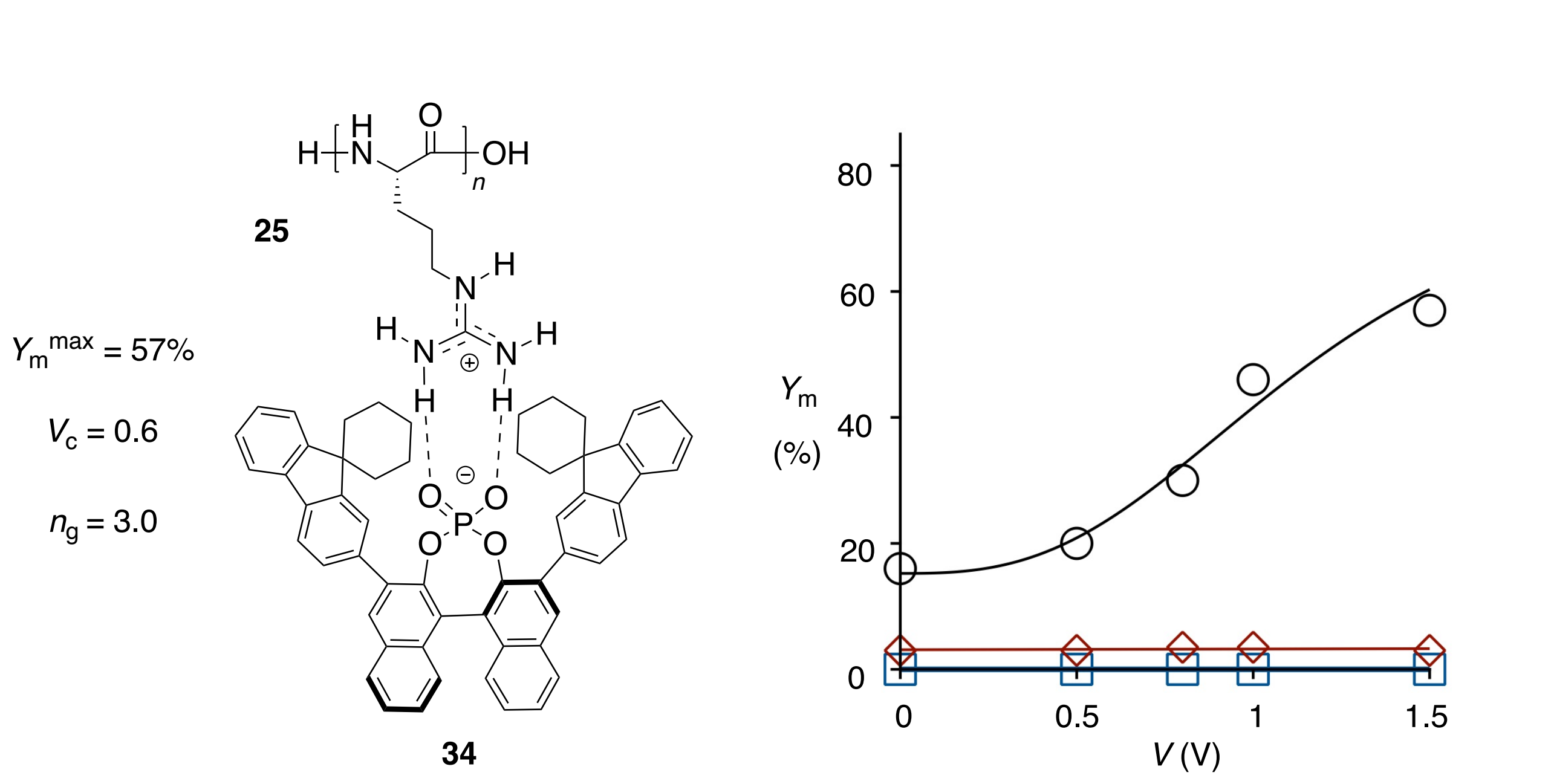


**Figure S36**. Dependence of microfluidic yield $Y_m$ (**18**: circles, **21**: squares, **24**: diamonds) on the applied voltage *V* for substrates **14** (50 mM) and **15** (75 mM) with Helmholtz components **25** (1 mM) and **34** (1 mM) in $CHCl_3$/DMSO 3:1, flow rate 5 µL/min, 100 µm foil, graphite electrodes, rt, with curve fit to Hill equation (circles only) and indication of $Y_m^{max}$ as maximal microfluidic yield, $V_c$ as critical voltage, $n_g$ as gating coefficient.

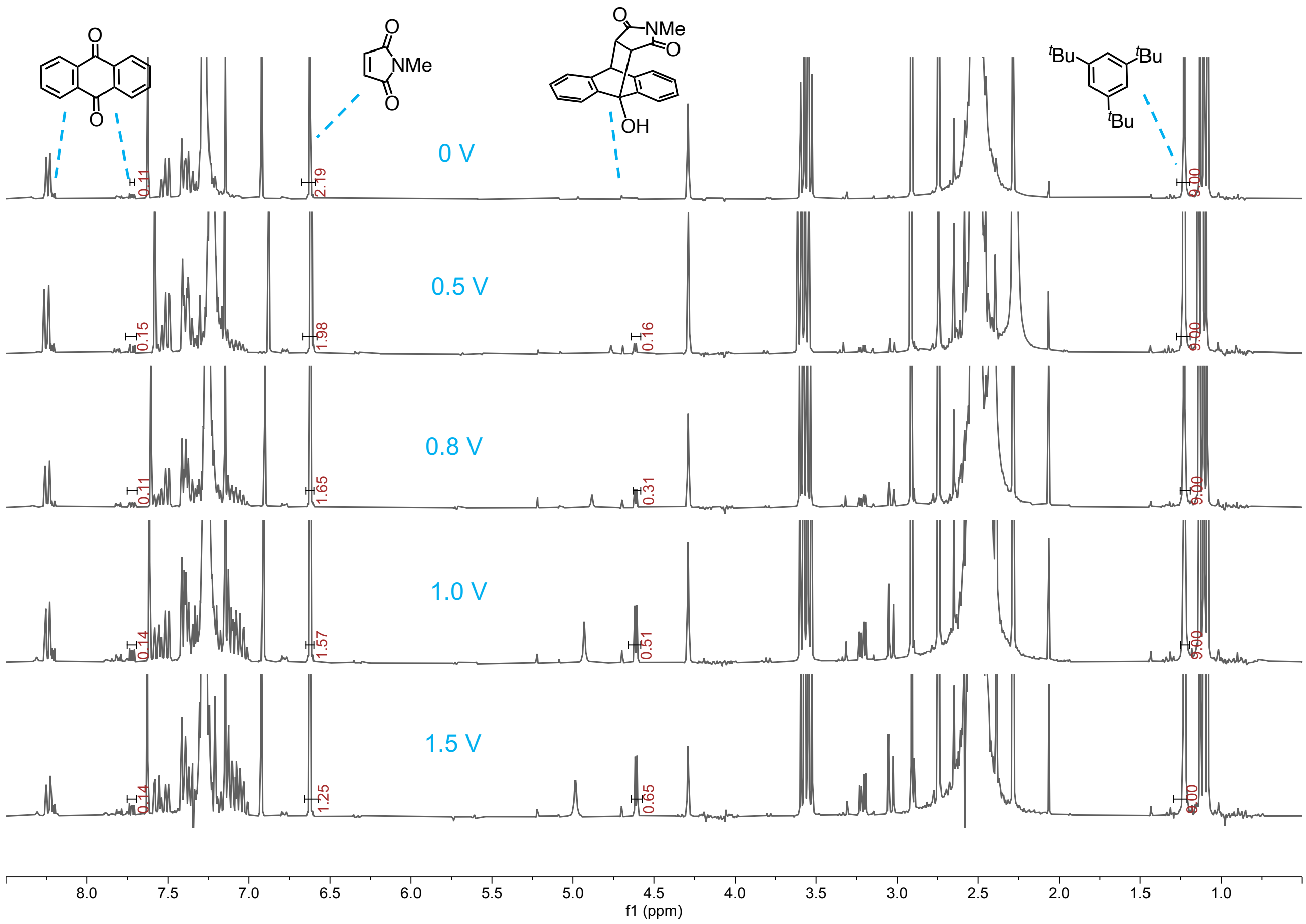


**Figure S37.** $^1$H NMR spectra (400 MHz, $CDCl_3$) of reaction mixtures obtained from **14** (50 mM), **15** (75 mM), **25** (1 mM) and **35** (1 mM) in $CHCl_3$/DMSO 3:1 (4 mL) after one passage through the microfluidic capacitor at 5 µL/min with $V$ = 0.0, 0.5, 0.8, 1.0 and 1.5 V (from top to bottom) applied, at rt.

**Table S17.** EFC with **14** (50 mM) and **15** (75 mM) and Helmholtz components **25** (1 mM) and **35** (1 mM) in $CHCl_3$/DMSO 3:1.[a]

| Entry | $V$ (V)[b] | $I$ (A)[c] | $Y_m^{\mathbf{18}}$ (%)[d] | $Y_m^{\mathbf{21}}$ (%)[e] | $Y_m^{\mathbf{24}}$ (%)[f] | *ee* (%)[g] |
|---|---|---|---|---|---|---|
| 1 | 0 | 0.000 | 0 | 0 | 2.8 | - |
| 2 | 0.5 | 0.000 | 16 | 0 | 3.8 | - |
| 3 | 0.8 | 0.000 | 31 | 0 | 2.8 | - |
| 4 | 1.0 | 0.000 | 51 | 0 | 3.5 | - |
| 5 | 1.5 | 0.000 | 65 | 0 | 3.5 | 0 |

[a]In microfluidic capacitor, flow rate 5 µL/min, 100 µm foil, graphite electrodes, with 1,3,5-tri-*tert*-butylbenzene as internal standard (IS), rt, flowrate 5 µL/min. [b]Applied voltage, in volt. [c]Steady-state current measured, in ampere. [d]Microfluidic yield of DA product **18**, in percent. [e]Microfluidic yield of Michael product **21**, in percent. [f]Microfluidic yield of oxidation product **24**, in percent. [g]The *ee* value of DA product **18**, in percent.

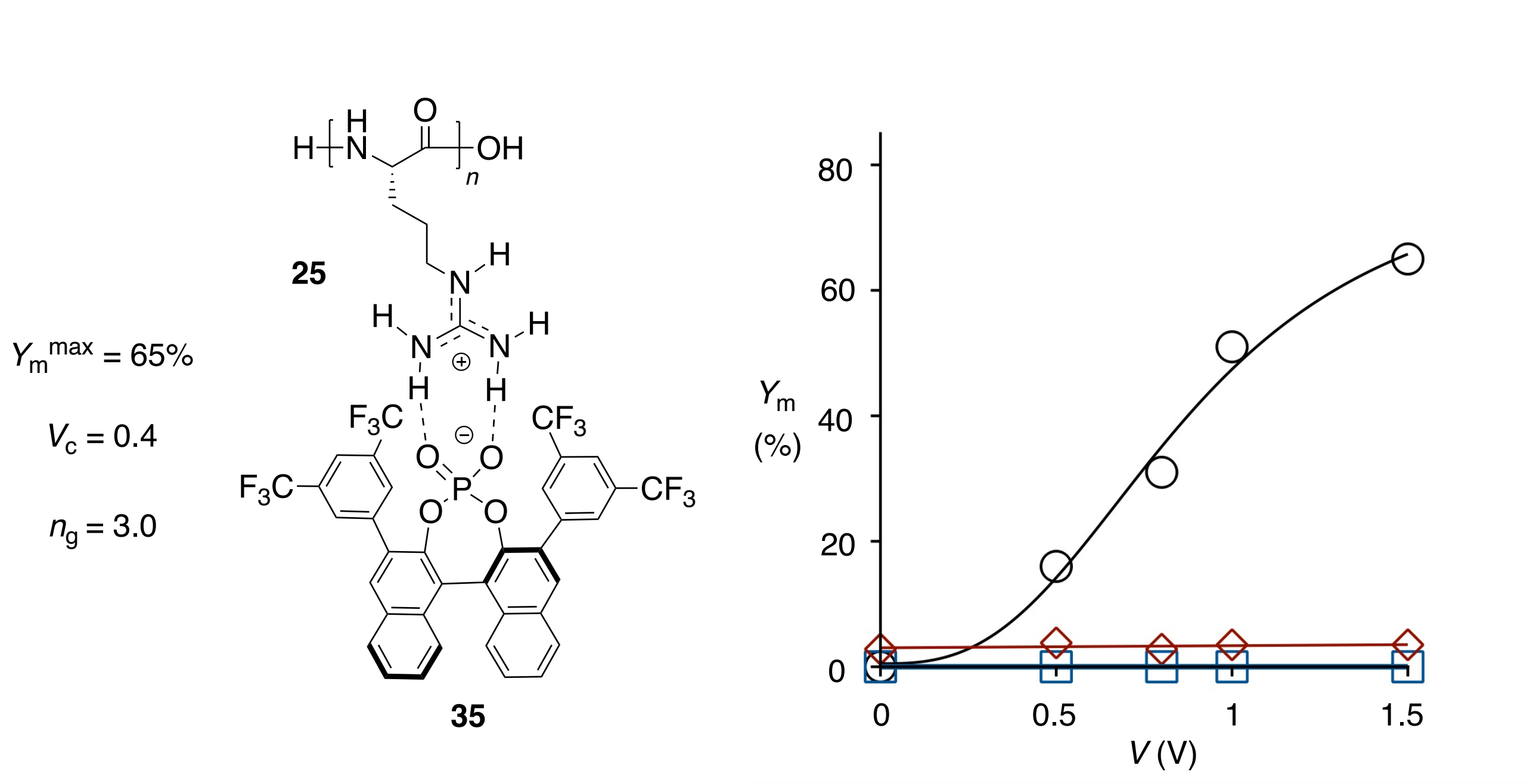


**Figure S38**. Dependence of microfluidic yield $Y_m$ (**18**: circles, **21**: squares, **24**: diamonds) on the applied voltage $V$ for substrates **14** (50 mM) and **15** (75 mM) with Helmholtz components **25** (1 mM) and **35** (1 mM) in $CHCl_3$/DMSO 3:1, flow rate 5 µL/min, 100 µm foil, graphite electrodes, rt, with curve fit to Hill equation (circles only) and indication of $Y_m^{max}$ as maximal microfluidic yield, $V_c$ as critical voltage, $n_g$ as gating coefficient.

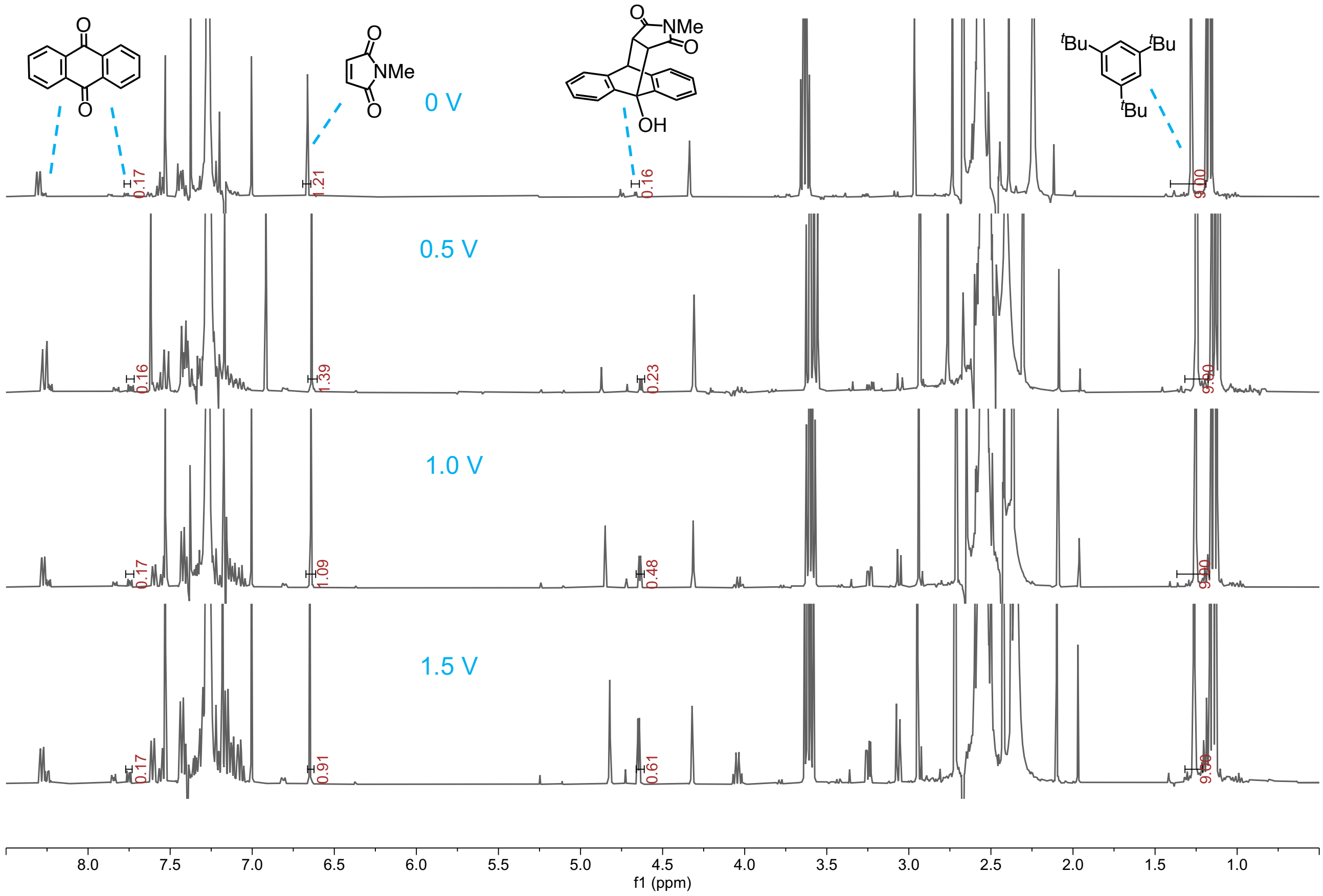


**Figure S39.** $^{1}$H NMR spectra (400 MHz, $CDCl_3$) of reaction mixtures obtained from **14** (50 mM), **15** (75 mM) and **29**·Na (1 mM) in $CHCl_3$/DMSO 3:1 (4 mL) after one passage through the microfluidic capacitor at 5 µL/min with $V$ = 0.0, 0.5, 1.0 and 1.5 V (from top to bottom) applied, at rt.

**Table S18.** EFC with **14** (50 mM) and **15** (75 mM) and Helmholtz components **29**·Na (1 mM) in $CHCl_3$/DMSO 3:1.[a]

| Entry | $V$ (V)[b] | $I$ (A)[c] | $Y_m^{\mathbf{18}}$ (%)[d] | $Y_m^{\mathbf{21}}$ (%)[e] | $Y_m^{\mathbf{24}}$ (%)[f] | *ee* (%)[g] |
|---|---|---|---|---|---|---|
| 1 | 0 | 0.000 | 16 | 0 | 4.3 | - |
| 2 | 0.5 | 0.000 | 23 | 0 | 4.0 | - |
| 3 | 1.0 | 0.000 | 48 | 0 | 4.3 | - |
| 4 | 1.5 | 0.000 | 61 | 0 | 4.3 | - |

[a]In microfluidic capacitor, flow rate 5 µL/min, 100 µm foil, graphite electrodes, with 1,3,5-tri-*tert*-butylbenzene as internal standard (IS), rt, flowrate 5 µL/min. [b]Applied voltage, in volt. [c]Steady-state current measured, in ampere. [d]Microfluidic yield of DA product **18**, in percent. [e]Microfluidic yield of Michael product **21**, in percent. [f]Microfluidic yield of oxidation product **24**, in percent. [g]The *ee* value of DA product **18**, in percent.

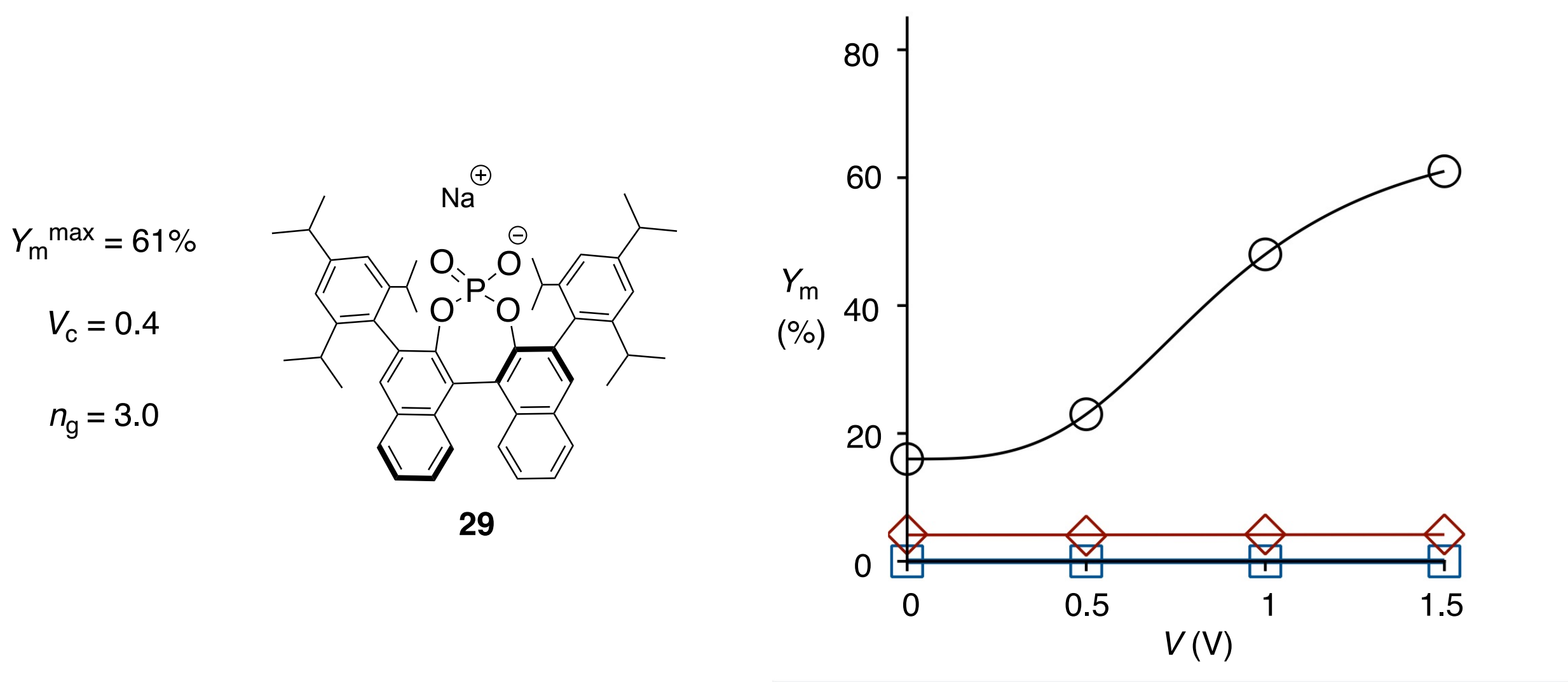


**Figure S40**. Dependence of microfluidic yield $Y_\mathrm{m}$ (**18**: circles, **21**: squares, **24**: diamonds) on the applied voltage *V* for substrates **14** (50 mM) and **15** (75 mM) with Helmholtz components **29**·Na (1 mM) in $CHCl_3$/DMSO 3:1, flow rate 5 µL/min, 100 µm foil, graphite electrodes, rt, with curve fit to Hill equation (circles only) and indication of $Y_\mathrm{m}^{\mathrm{max}}$ as maximal microfluidic yield, $V_\mathrm{c}$ as critical voltage, $n_\mathrm{g}$ as gating coefficient.

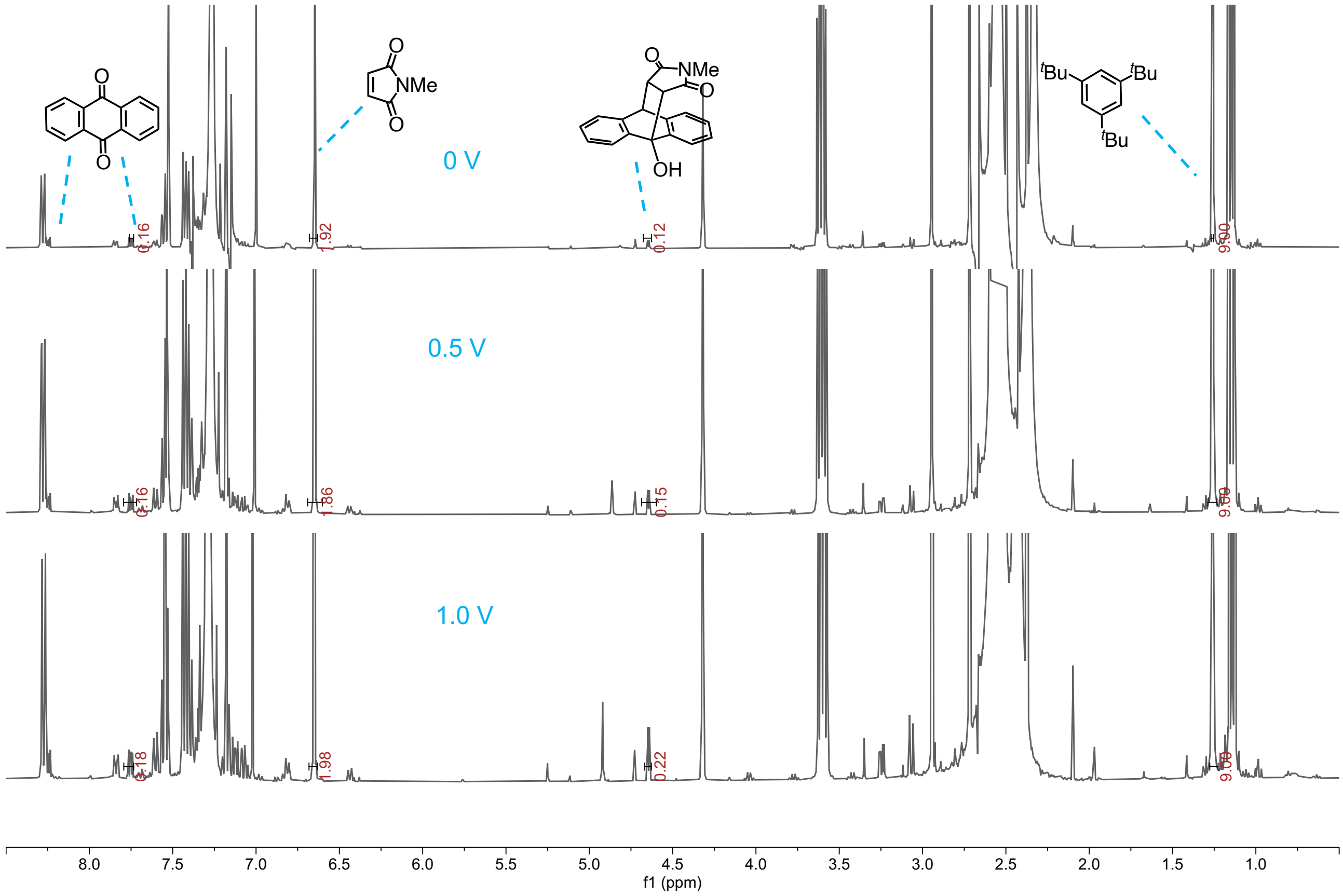


**Figure S41.** $^{1}$H NMR spectra (400 MHz, $CDCl_3$) of reaction mixtures obtained from **14** (50 mM), **15** (75 mM), **30** (1 mM) and **36** (1 mM) in $CHCl_3$/DMSO 3:1 (4 mL) after one passage through the microfluidic capacitor at 5 µL/min with $V$ = 0.0, 0.5 and 1.0 V (from top to bottom) applied, at rt.

**Table S19.** EFC with **14** (50 mM) and **15** (75 mM) and Helmholtz components **30** (1 mM) and **36** (1 mM) in $CHCl_3$/DMSO 3:1.[a]

| Entry | $V$ (V)[b] | $I$ (A)[c] | $Y_m^{\mathbf{18}}$ (%)[d] | $Y_m^{\mathbf{21}}$ (%)[e] | $Y_m^{\mathbf{24}}$ (%)[f] | *ee* (%)[g] |
|---|---|---|---|---|---|---|
| 1 | 0 | 0.000 | 12 | 0 | 4 | - |
| 2 | 0.5 | 0.000 | 15 | 0 | 4 | - |
| 3 | 1.0 | 0.000 | 22 | 0 | 4.5 | - |

[a]In microfluidic capacitor, flow rate 5 µL/min, 100 µm foil, graphite electrodes, with 1,3,5-tri-*tert*-butylbenzene as internal standard (IS), rt, flowrate 5 µL/min. [b]Applied voltage, in volt. [c]Steady-state current measured, in ampere. [d]Microfluidic yield of DA product **18**, in percent. [e]Microfluidic yield of Michael product **21**, in percent. [f]Microfluidic yield of oxidation product **24**, in percent. [g]The *ee* value of DA product **18**, in percent.

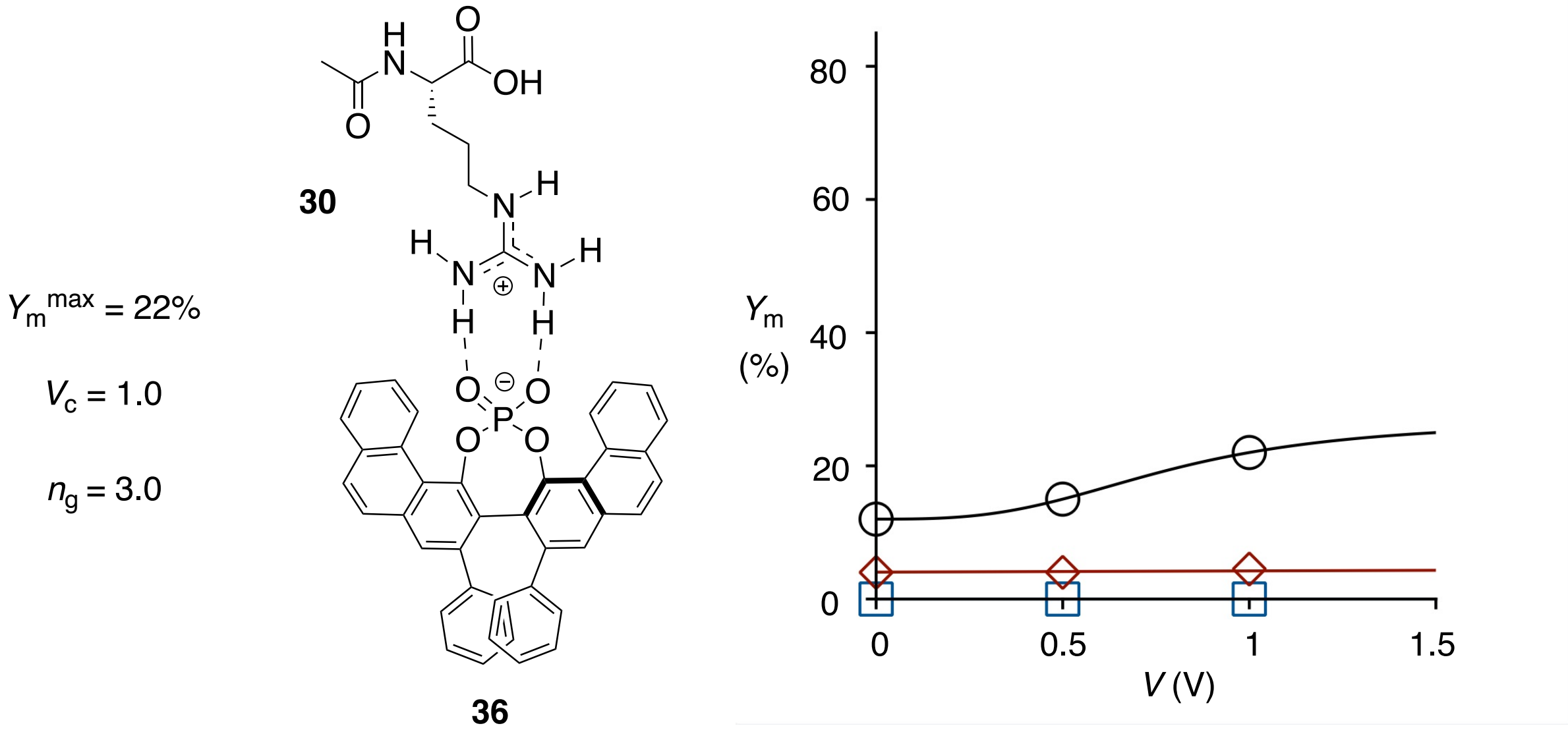


**Figure S42**. Dependence of microfluidic yield $Y_\mathrm{m}$ (**18**: circles, **21**: squares, **24**: diamonds) on the applied voltage $V$ for substrates **14** (50 mM) and **15** (75 mM) with Helmholtz components **30** (1 mM) and **36** (1 mM) in $CHCl_3$/DMSO 3:1, flow rate 5 µL/min, 100 µm foil, graphite electrodes, rt, with curve fit to Hill equation (circles only) and indication of $Y_\mathrm{m}^\mathrm{max}$ as maximal microfluidic yield, $V_\mathrm{c}$ as critical voltage, $n_\mathrm{g}$ as gating coefficient.

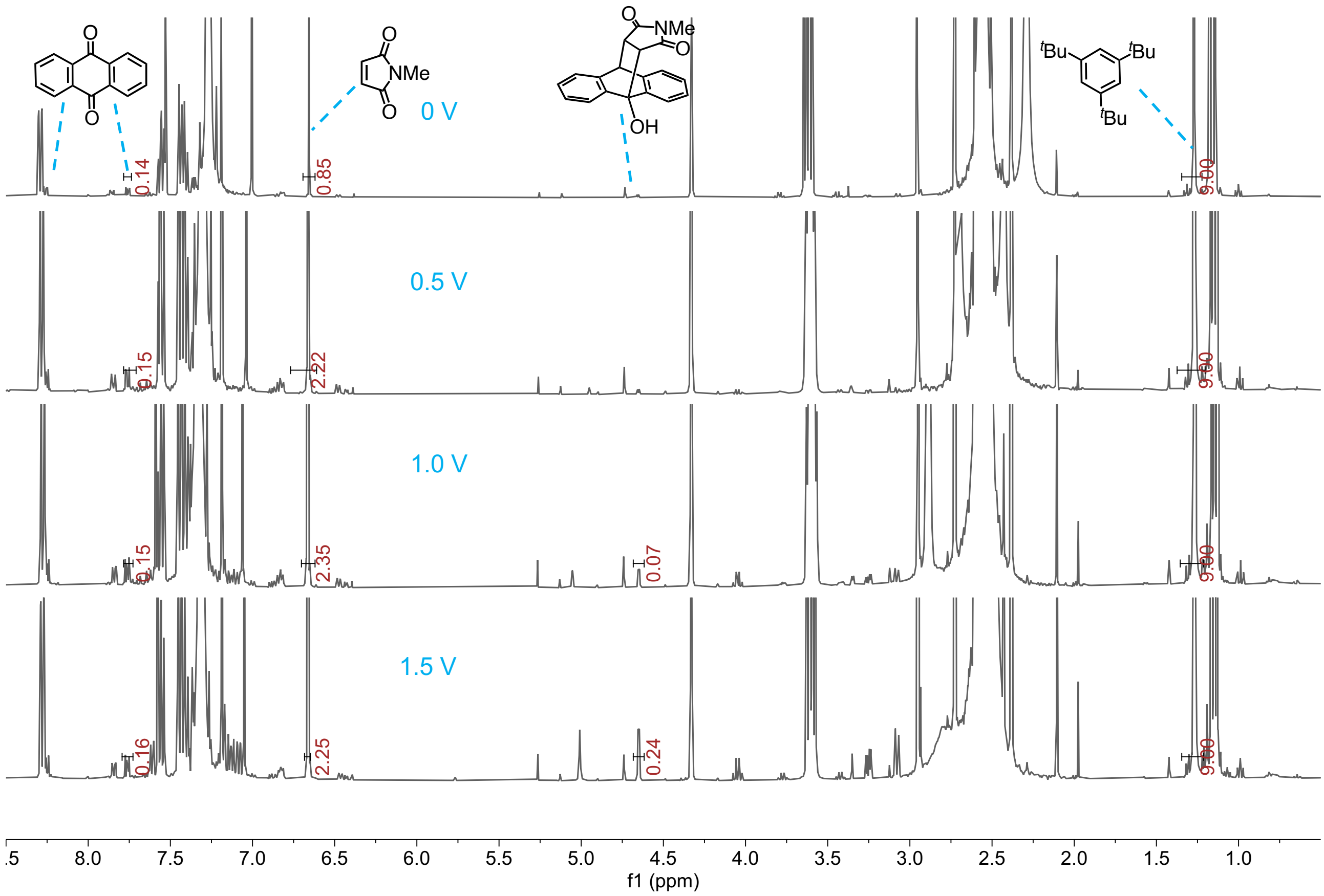


**Figure S43.** $^1$H NMR spectra (400 MHz, $CDCl_3$) of reaction mixtures obtained from **14** (50 mM), **15** (75 mM), **30** (1 mM) and **37** (1 mM) in $CHCl_3$/DMSO 3:1 (4 mL) after one passage through the microfluidic capacitor at 5 µL/min with $V$ = 0.0, 0.5, 1.0 and 1.5 V (from top to bottom) applied, at rt.

**Table S20.** EFC with **14** (50 mM) and **15** (75 mM) and Helmholtz components **30** (1 mM) and **37** (1 mM) in $CHCl_3$/DMSO 3:1.[a]

| Entry | $V$ (V)[b] | $I$ (A)[c] | $Y_m$**18** (%)[d] | $Y_m$**21** (%)[e] | $Y_m$**24** (%)[f] | *ee* (%)[g] |
|---|---|---|---|---|---|---|
| 1 | 0 | 0.000 | 0 | 0 | 3.5 | - |
| 2 | 0.5 | 0.000 | 0 | 0 | 3.8 | - |
| 3 | 1.0 | 0.000 | 7 | 0 | 3.8 | - |
| 4 | 1.5 | 0.000 | 24 | 0 | 4 | - |

[a]In microfluidic capacitor, flow rate 5 µL/min, 100 µm foil, graphite electrodes, with 1,3,5-tri-*tert*-butylbenzene as internal standard (IS), rt, flowrate 5 µL/min. [b]Applied voltage, in volt. [c]Steady-state current measured, in ampere. [d]Microfluidic yield of DA product **18**, in percent. [e]Microfluidic yield of Michael product **21**, in percent. [f]Microfluidic yield of oxidation product **24**, in percent. [g]The *ee* value of DA product **18**, in percent.

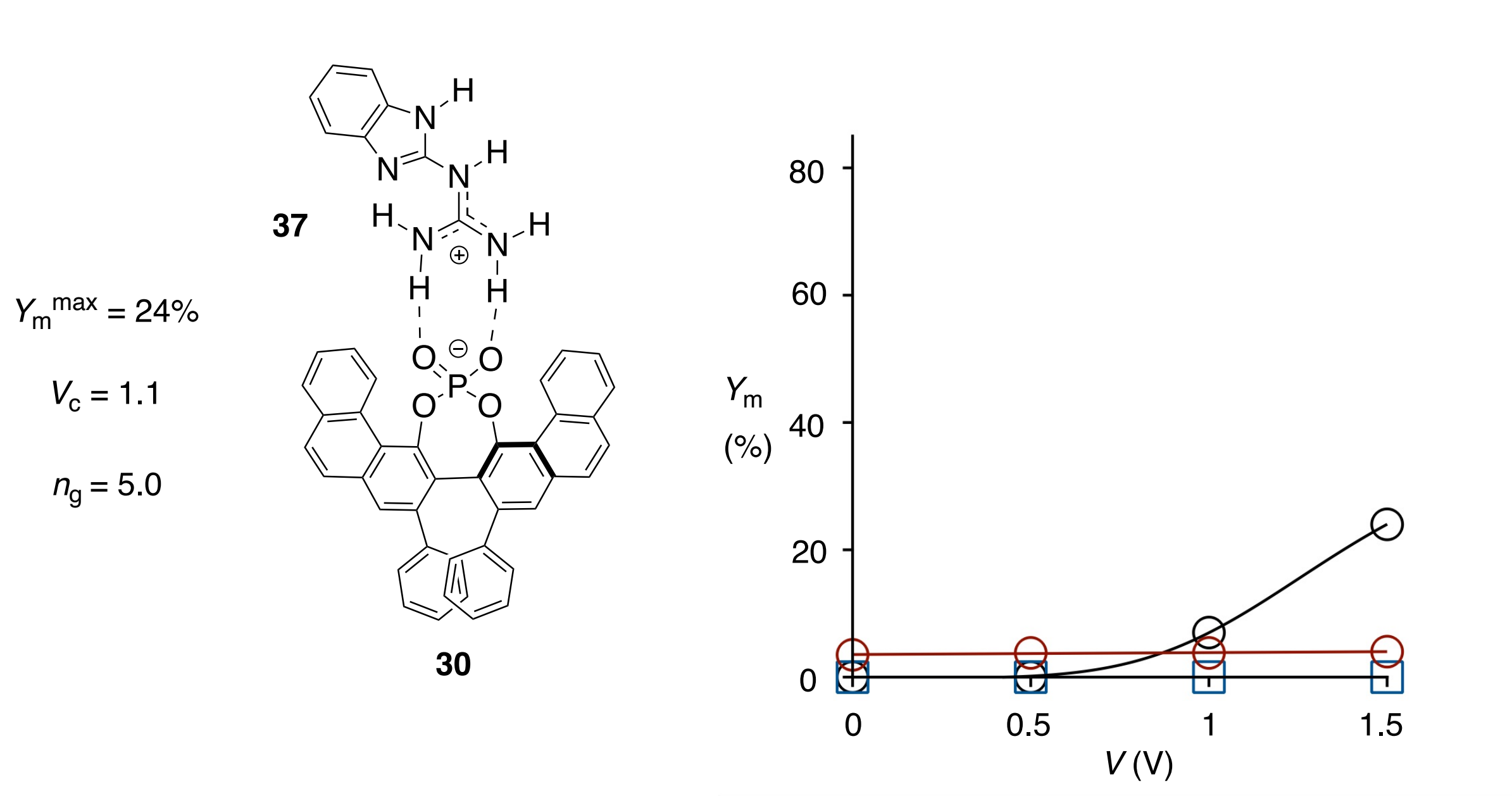


**Figure S44.** Dependence of microfluidic yield $Y_m$ (**18**: circles, **21**: squares, **24**: diamonds) on the applied voltage $V$ for substrates **14** (50 mM) and **15** (75 mM) with **30** (1 mM) and **37** (1 mM) in $CHCl_3$/DMSO 3:1, flow rate 5 µL/min, 100 µm foil, graphite electrodes, rt, with curve fit to Hill equation (circles only) and indication of $Y_m^{max}$ as maximal microfluidic yield, $V_c$ as critical voltage, $n_g$ as gating coefficient.

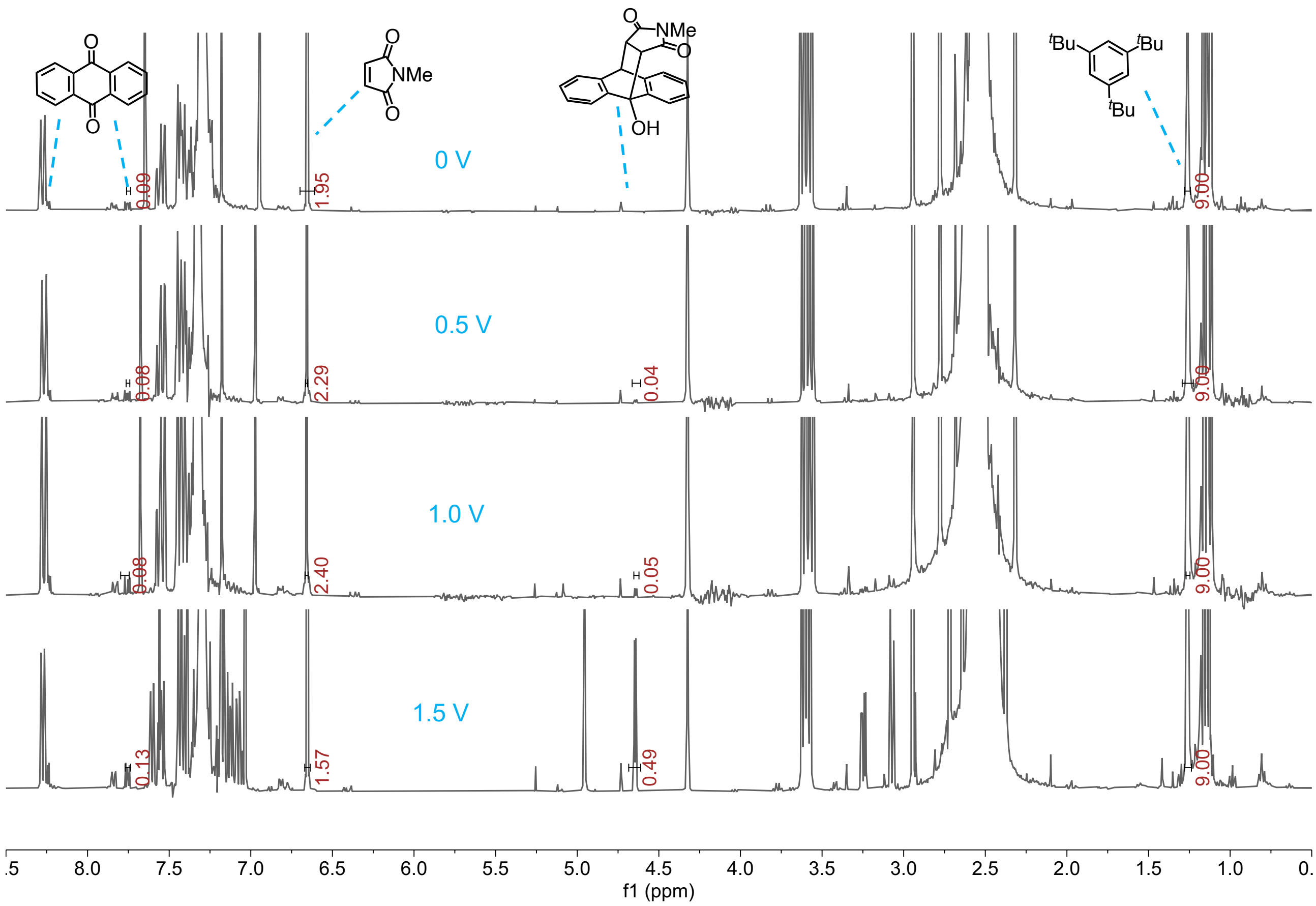


**Figure S45.** $^{1}$H NMR spectra (400 MHz, $CDCl_3$) of reaction mixtures obtained from **14** (50 mM), **15** (75 mM), **38** (1 mM) and **39** (1 mM) in $CHCl_3$/DMSO 3:1 (4 mL) after one passage through the microfluidic capacitor at 5 µL/min with $V$ = 0.0, 0.5, 1.0 and 1.5 V (from top to bottom) applied, at rt.

**Table S21.** EFC with **14** (50 mM) and **15** (75 mM) and Helmholtz components **38** (1 mM) and **39** (1 mM) in $CHCl_3$/DMSO 3:1.[a]

| Entry | *V* (V)[b] | *I* (A)[c] | $Y_m^{\mathbf{18}}$ (%)[d] | $Y_m^{\mathbf{21}}$ (%)[e] | $Y_m^{\mathbf{24}}$ (%)[f] | *ee* (%)[g] |
|---|---|---|---|---|---|---|
| 1 | 0 | 0.000 | 0 | 0 | 2.3 | - |
| 2 | 0.5 | 0.000 | 4 | 0 | 2.0 | - |
| 3 | 1.0 | 0.000 | 5 | 0 | 2.0 | - |
| 4 | 1.5 | 0.000 | 49 | 0 | 3.3 | - |

[a]In microfluidic capacitor, flow rate 5 µL/min, 100 µm foil, graphite electrodes, with 1,3,5-tri-*tert*-butylbenzene as internal standard (IS), rt, flowrate 5 µL/min. [b]Applied voltage, in volt. [c]Steady-state current measured, in ampere. [d]Microfluidic yield of DA product **18**, in percent. [e]Microfluidic yield of Michael product **21**, in percent. [f]Microfluidic yield of oxidation product **24**, in percent. [g]The *ee* value of DA product **18**, in percent.

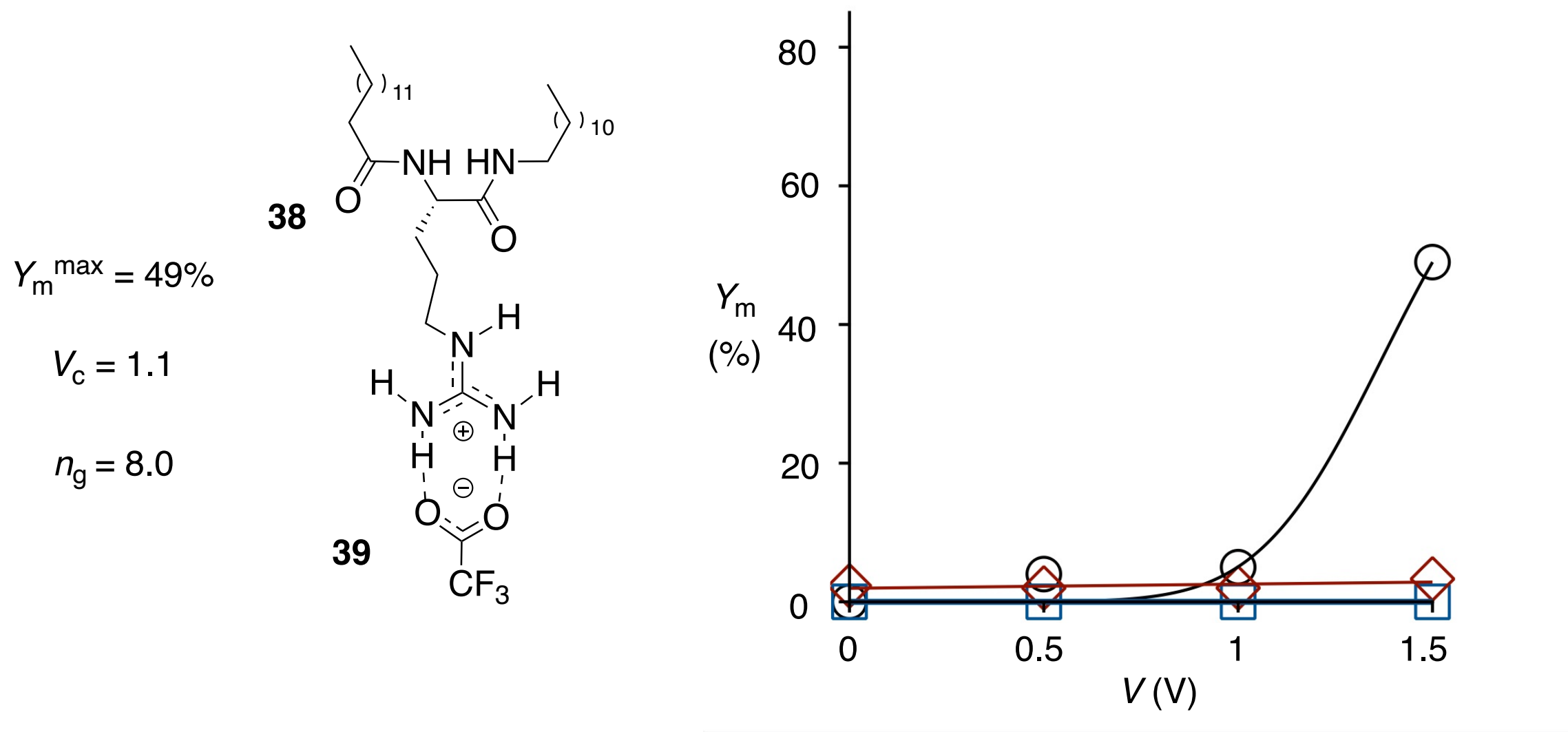


**Figure S46**. Dependence of microfluidic yield $Y_m$ (**18**: circles, **21**: squares, **24**: diamonds) on the applied voltage $V$ for substrates **14** (50 mM) and **15** (75 mM) Helmholtz components **38** (1 mM) and **39** (1 mM) in $CHCl_3$/DMSO 3:1, flow rate 5 µL/min, 100 µm foil, graphite electrodes, rt, with curve fit to Hill equation (circles only) and indication of $Y_m^{max}$ as maximal microfluidic yield, $V_c$ as critical voltage, $n_g$ as gating coefficient.

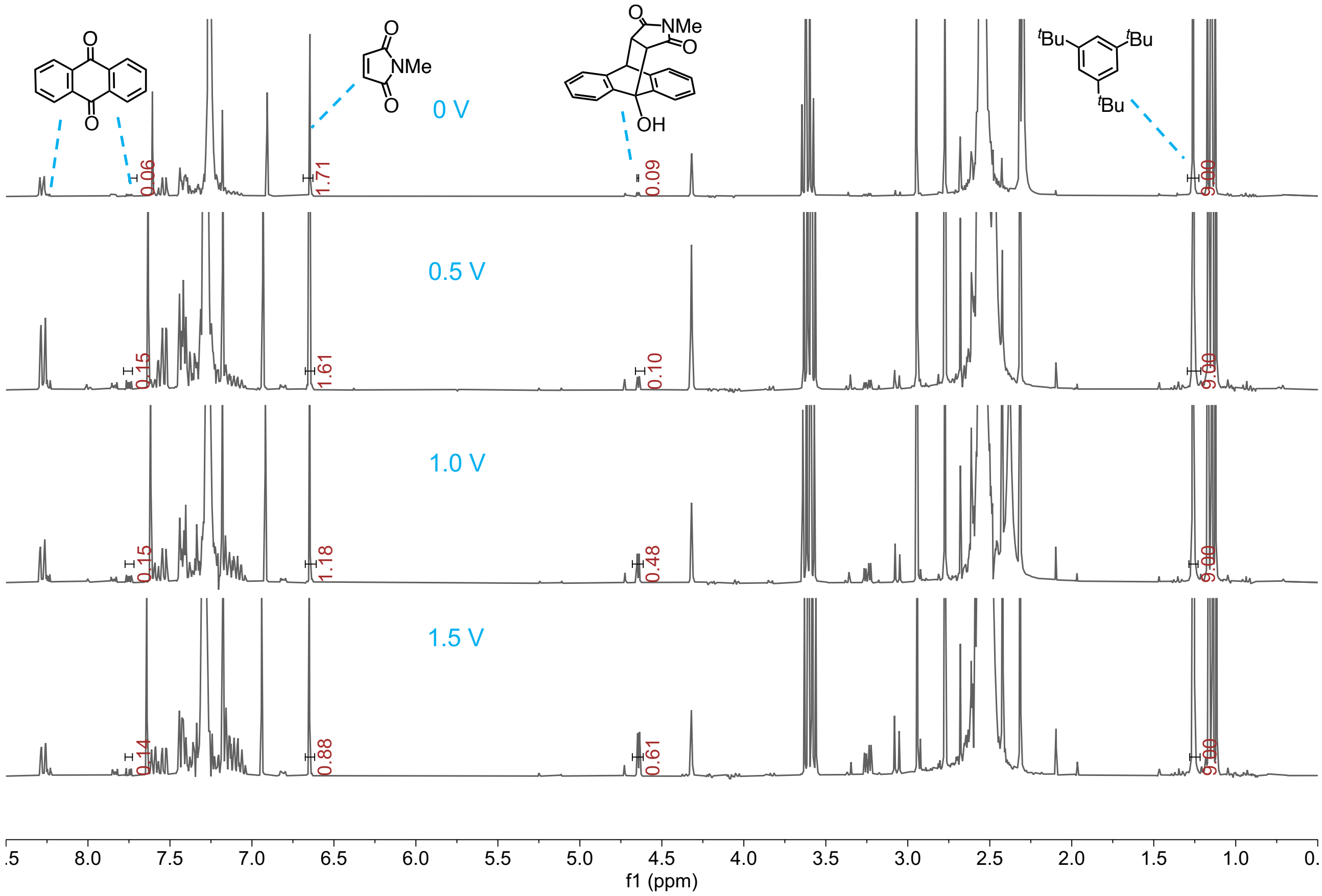


**Figure S47.** $^{1}$H NMR spectra (400 MHz, $CDCl_3$) of reaction mixtures obtained from **14** (50 mM), **15** (75 mM) and Helmholtz components **40** and $Br^-$ (1 mM) in $CHCl_3$/DMSO 3:1 (4 mL) after one passage through the microfluidic capacitor at 5 µL/min with $V$ = 0.0, 0.5, 1.0 and 1.5 V (from top to bottom) applied, at rt.

**Table S22.** EFC with **14** (50 mM) and **15** (75 mM) and Helmholtz components **40**·Br (1 mM) in $CHCl_3$/DMSO 3:1.[a]

| Entry | *V* (V)[b] | *I* (A)[c] | $Y_m^{\mathbf{18}}$ (%)[d] | $Y_m^{\mathbf{21}}$ (%)[e] | $Y_m^{\mathbf{24}}$ (%)[f] | *ee* (%)[g] |
|---|---|---|---|---|---|---|
| 1 | 0 | 0.000 | 9 | 0 | 1.5 | - |
| 2 | 0.5 | 0.000 | 10 | 0 | 3.8 | - |
| 3 | 1.0 | 0.000 | 48 | 0 | 3.8 | 10 |
| 4 | 1.5 | 0.000 | 61 | 0 | 3.5 | 11 |

[a]In microfluidic capacitor, flow rate 5 µL/min, 100 µm foil, graphite electrodes, with 1,3,5-tri-*tert*-butylbenzene as internal standard (IS), rt, flowrate 5 µL/min. [b]Applied voltage, in volt. [c]Steady-state current measured, in ampere. [d]Microfluidic yield of DA product **18**, in percent. [e]Microfluidic yield of Michael product **21**, in percent. [f]Microfluidic yield of oxidation product **24**, in percent. [g]The *ee* value of DA product **18**, in percent.

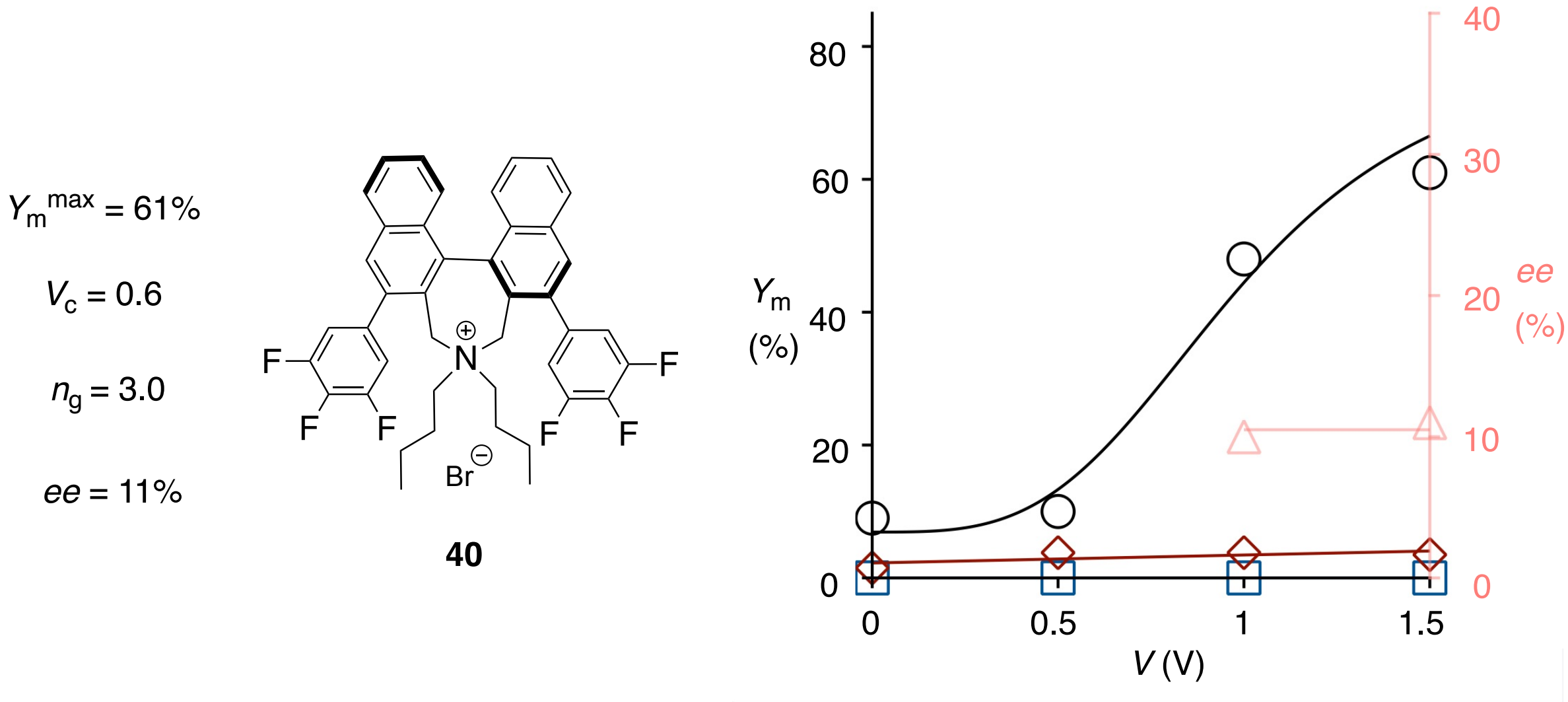


**Figure S48.** Dependence of microfluidic yield $Y_m$ (black, left, **18**: circles, **21**: squares, **24**: diamonds) and *ee* (right, red, **18**: triangles) on the applied voltage *V* for substrates **14** (50 mM) and **15** (75 mM) with Helmholtz components **40** and $Br^-$ (1 mM, 2 mol%) in $CHCl_3$/DMSO 3:1, flow rate 5 µL/min, 100 µm foil, graphite electrodes, rt, with curve fit to Hill equation (circles only) and indication of $Y_m^{max}$ as maximal microfluidic yield, $V_c$ as critical voltage, $n_g$ as gating coefficient and *ee* as enantioselectivity for **18**.

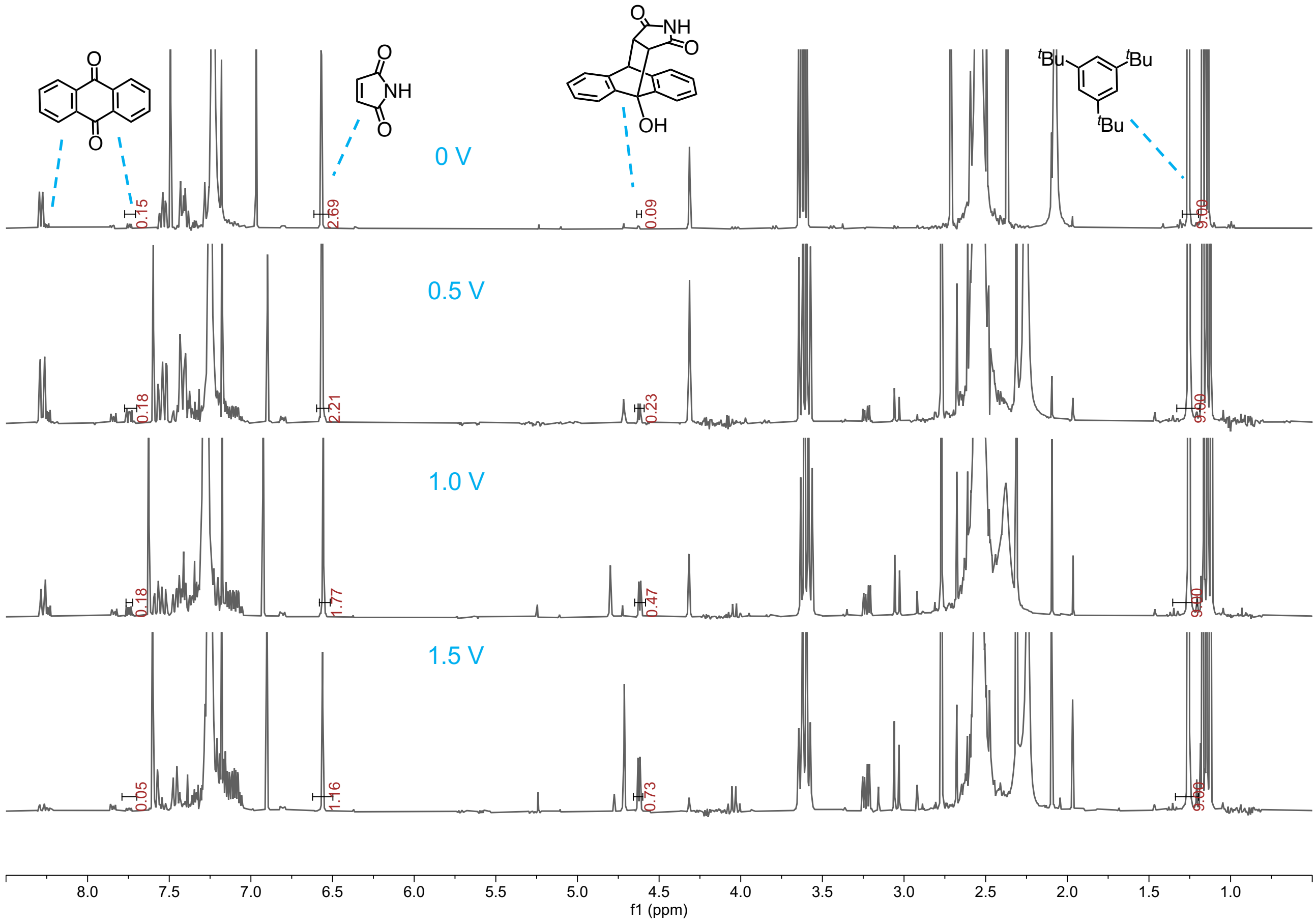


**Figure S49.** $^{1}$H NMR spectra (400 MHz, $CDCl_3$) of reaction mixtures obtained from **14** (50 mM), **16** (75 mM) and Helmholtz components **27** and $Cl^-$ (1 mM, 2 mol%) in $CHCl_3$/DMSO 3:1 (4 mL) after one passage through the microfluidic capacitor at 5 μL/min with $V$ = 0.0, 0.5, 1.0 and 1.5 V (from top to bottom) applied, at rt.

**Table S23.** EFC with **14** (50 mM) and **16** (75 mM) and Helmholtz components **27** and $Cl^-$ (1 mM) in $CHCl_3$/DMSO 3:1.[a]

| Entry | *V* (V)[b] | *I* (A)[c] | $Y_m^{\mathbf{19}}$ (%)[d] | $Y_m^{\mathbf{22}}$ (%)[e] | $Y_m^{\mathbf{24}}$ (%)[f] | *ee* (%)[g] |
|---|---|---|---|---|---|---|
| 1 | 0 | 0.000 | 9 | 0 | 3.8 | - |
| 2 | 0.5 | 0.000 | 23 | 0 | 4.5 | - |
| 3 | 1.0 | 0.000 | 47 | 0 | 4.5 | - |
| 4 | 1.5 | 0.000 | 73 | 0 | 1.3 | - |

[a]In microfluidic capacitor, flow rate 5 µL/min, 100 µm foil, graphite electrodes, with 1,3,5-tri-*tert*-butylbenzene as internal standard (IS), rt, flowrate 5 µL/min. [b]Applied voltage, in volt. [c]Steady-state current measured, in ampere. [d]Microfluidic yield of DA product **19**, in percent. [e]Microfluidic yield of Michael product **22**, in percent. [f]Microfluidic yield of oxidation product **24**, in percent. [g]The *ee* value of DA product **19**, in percent.

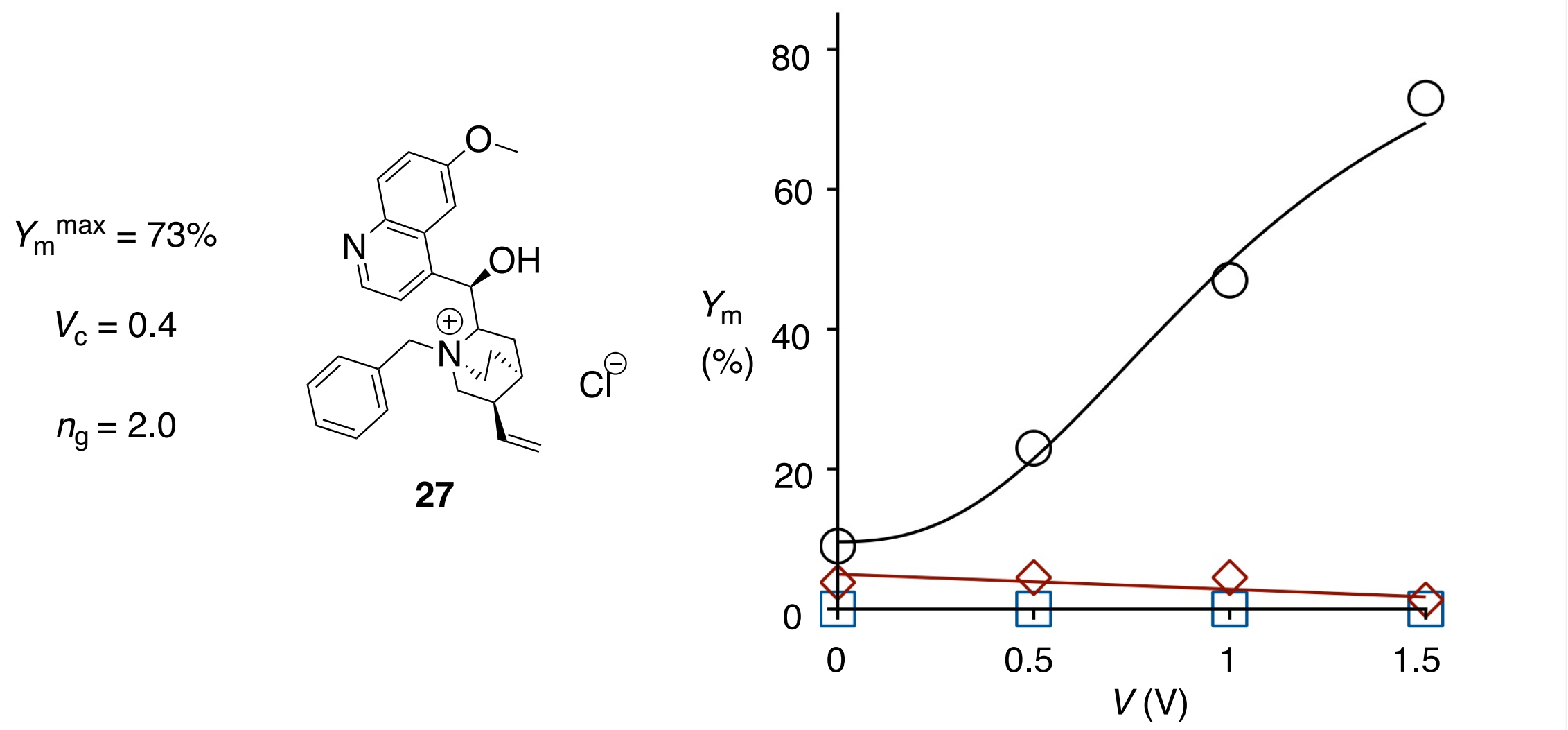


**Figure S50.** Dependence of microfluidic yield $Y_m$ (**19**: circles, **22**: squares, **24**: diamonds) on the applied voltage $V$ for substrates **14** (50 mM) and **16** (75 mM) with Helmholtz components **27** and $\mathbf{Cl^-}$ (1 mM, 2 mol%) in $CHCl_3$/DMSO 3:1, flow rate 5 μL/min, 100 μm foil, graphite electrodes, rt, with curve fit to Hill equation (circles only) and indication of $Y_m^{max}$ as maximal microfluidic yield, $V_c$ as critical voltage, $n_g$ as gating coefficient.

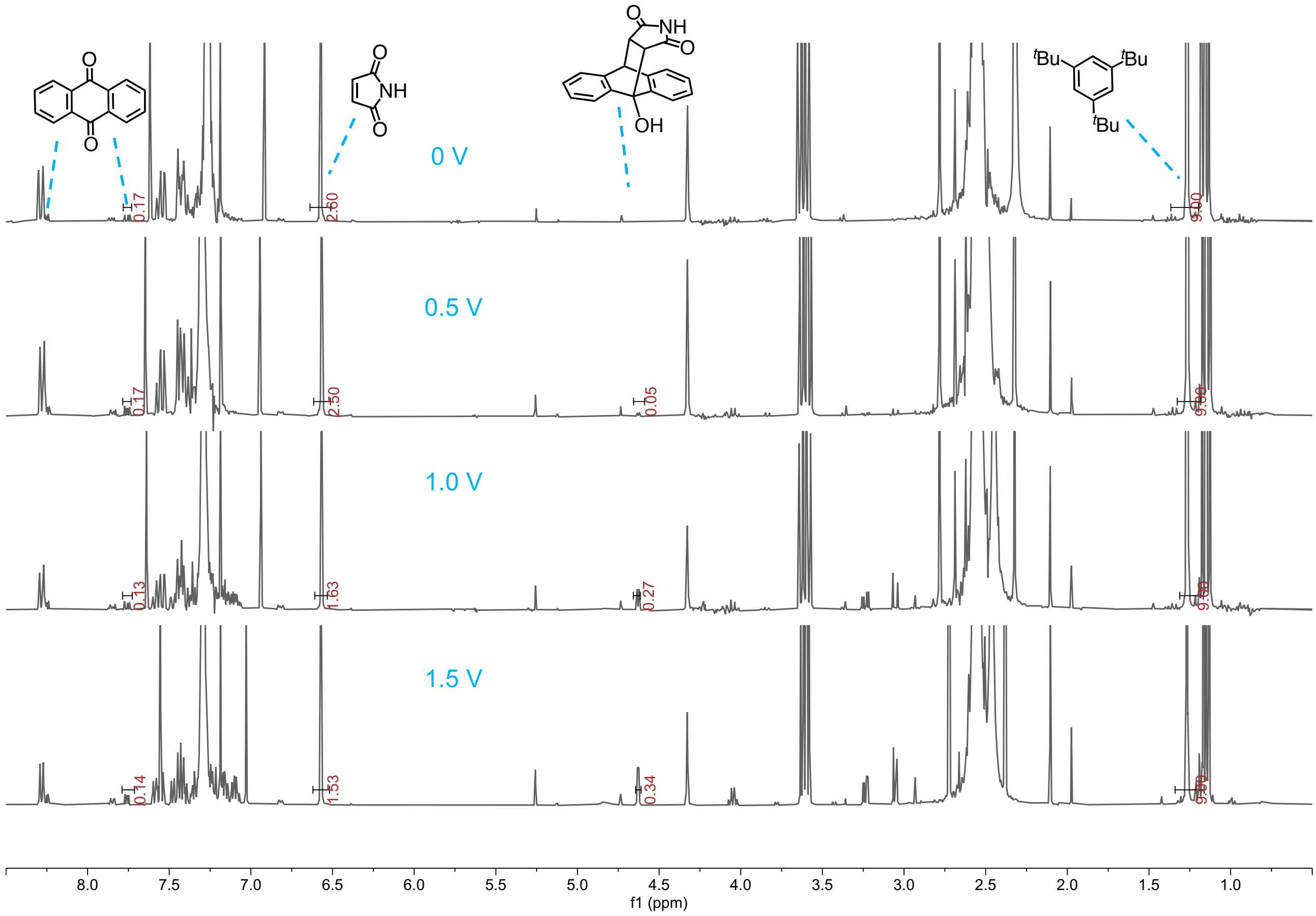


**Figure S51.** $^{1}H$ NMR spectra (400 MHz, $CDCl_3$) of reaction mixtures obtained from **14** (50 mM) and **16** (75 mM) in $CHCl_3$/DMSO 3:1 (4 mL) after one passage through the microfluidic capacitor at 5 μL/min with $V$ = 0.0, 0.5, 1.0 and 1.5 V (from top to bottom) applied, at rt.

**Table S24.** EFC with **14** (50 mM) and **16** (75 mM).[a]

| Entry | *V* (V)[b] | *I* (A)[c] | $Y_m^{\mathbf{19}}$ (%)[d] | $Y_m^{\mathbf{22}}$ (%)[e] | $Y_m^{\mathbf{24}}$ (%)[f] | *ee* (%)[g] |
|---|---|---|---|---|---|---|
| 1 | 0 | 0.000 | 0 | 0 | 4.3 | - |
| 2 | 0.5 | 0.000 | 5 | 0 | 4.3 | - |
| 3 | 1.0 | 0.000 | 27 | 0 | 4.5 | - |
| 4 | 1.5 | 0.000 | 34 | 0 | 1.3 | - |

[a]In microfluidic capacitor, flow rate 5 µL/min, 100 µm foil, graphite electrodes, with 1,3,5-tri-*tert*-butylbenzene as internal standard (IS), rt, flowrate 5 µL/min. [b]Applied voltage, in volt. [c]Steady-state current measured, in ampere. [d]Microfluidic yield of DA product **19**, in percent. [e]Microfluidic yield of Michael product **22**, in percent. [f]Microfluidic yield of oxidation product **24**, in percent. [g]The *ee* value of DA product **19**, in percent.

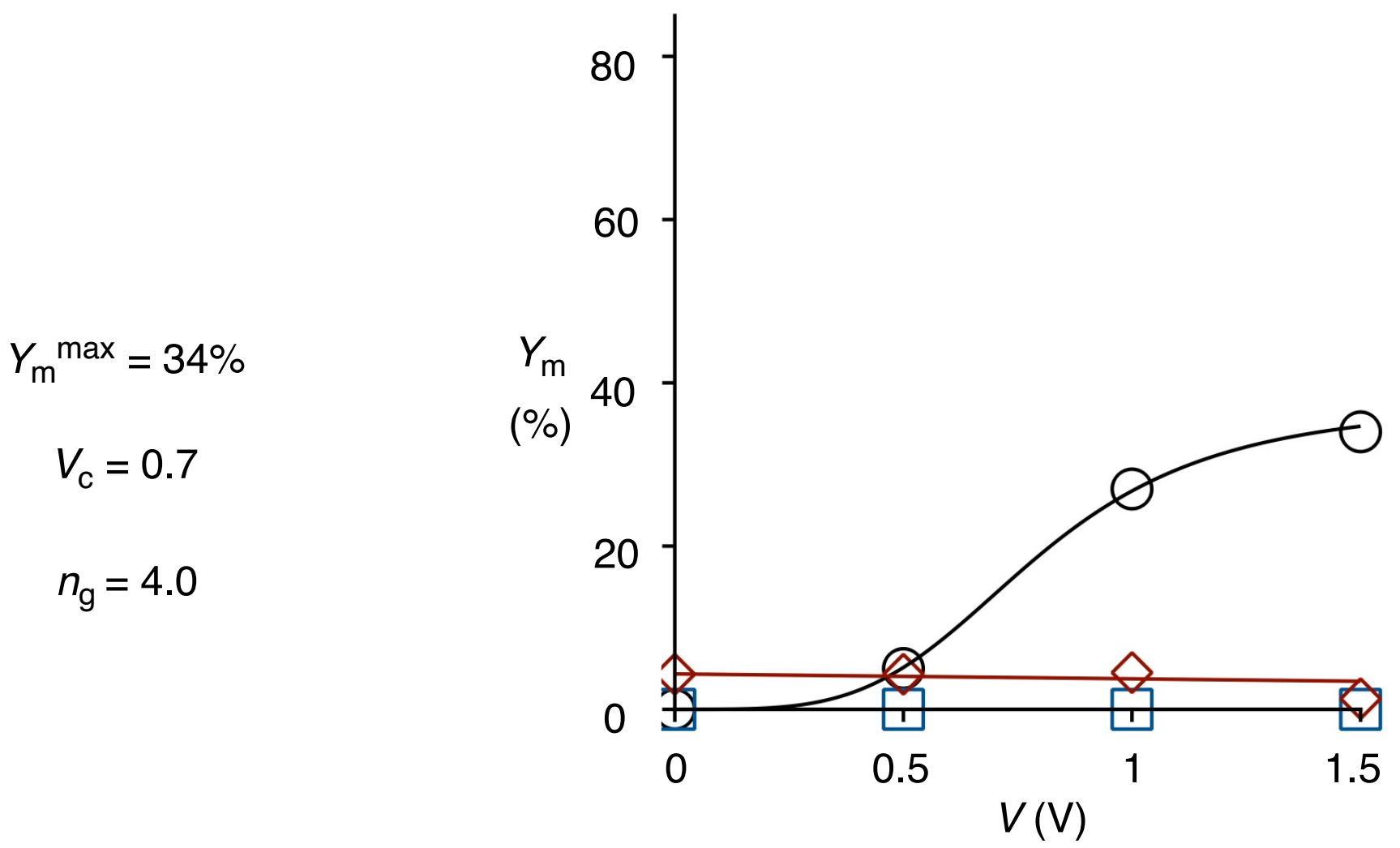


**Figure S52**. Dependence of microfluidic yield $Y_m$ (**19**: circles, **22**: squares, **24**: diamonds) on the applied voltage $V$ for substrates **14** (50 mM) and **16** (75 mM) in $CHCl_3$/DMSO 3:1, flow rate 5 µL/min, 100 µm foil, graphite electrodes, rt, with curve fit to Hill equation (circles only) and indication of $Y_m^{max}$ as maximal microfluidic yield, $V_c$ as critical voltage, $n_g$ as gating coefficient.

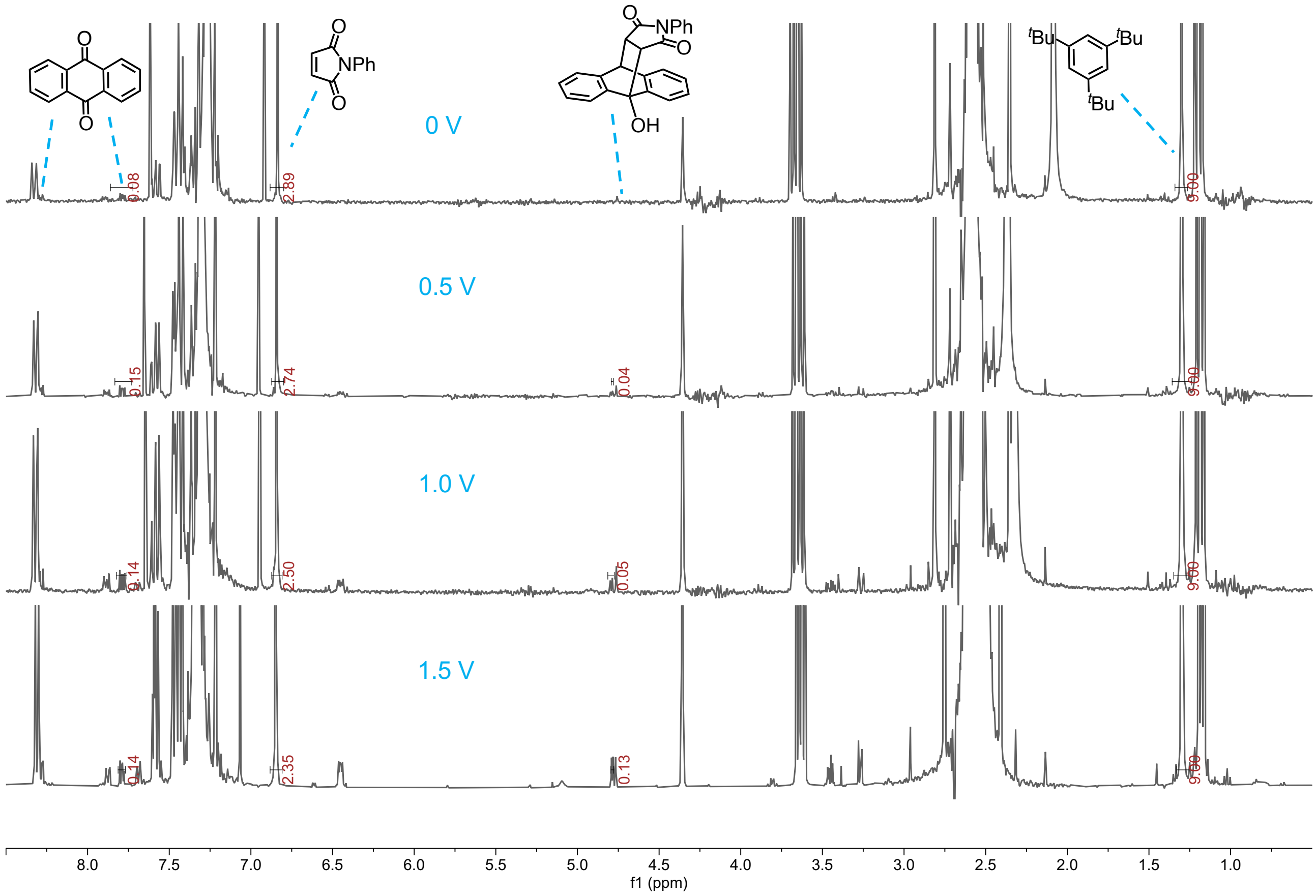


**Figure S53.** $^{1}$H NMR spectra (400 MHz, $CDCl_3$) of reaction mixtures obtained from **14** (50 mM) and **17** (75 mM) in $CHCl_3$/DMSO 3:1 (4 mL) after one passage through the microfluidic capacitor at 5 μL/min with $V$ = 0.0, 0.5, 1.0 and 1.5 V (from top to bottom) applied, at rt.

**Table S25.** EFC with **14** (50 mM) and **17** (75 mM).[a]

| Entry | *V* (V)[b] | *I* (A)[c] | $Y_m$**20** (%)[d] | $Y_m$**23** (%)[e] | $Y_m$**24** (%)[f] | *ee* (%)[g] |
|---|---|---|---|---|---|---|
| 1 | 0 | 0.000 | 0 | 0 | 2.0 | - |
| 2 | 0.5 | 0.000 | 4 | 0 | 3.8 | - |
| 3 | 1.0 | 0.000 | 5 | 0 | 3.5 | - |
| 4 | 1.5 | 0.000 | 13 | 0 | 3.5 | - |

[a]In microfluidic capacitor, flow rate 5 µL/min, 100 µm foil, graphite electrodes, with 1,3,5-tri-*tert*-butylbenzene as internal standard (IS), rt, flowrate 5 µL/min. [b]Applied voltage, in volt. [c]Steady-state current measured, in ampere. [d]Microfluidic yield of DA product **20**, in percent. [e]Microfluidic yield of Michael product **23**, in percent. [f]Microfluidic yield of oxidation product **24**, in percent. [g]The *ee* value of DA product **20**, in percent.

$Y_m^{max}$ = 13%

$V_c$ = 1.3

$n_g$ = 2.0

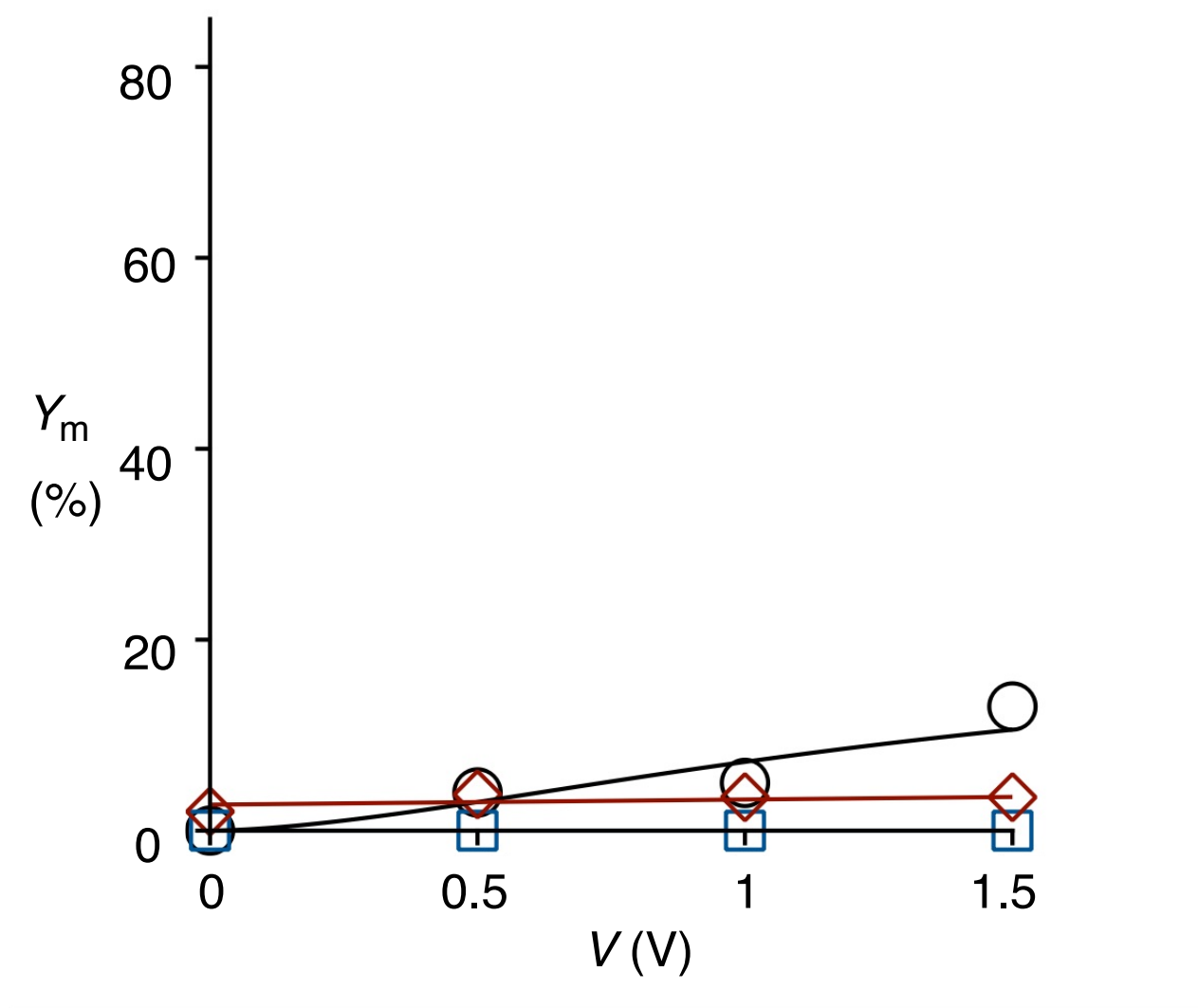


**Figure S54**. Dependence of microfluidic yield $Y_m$ (**20**: circles, **23**: squares, **24**: diamonds) on the applied voltage $V$ for substrates **14** (50 mM) and **17** (75 mM) in $CHCl_3$/DMSO 3:1, flow rate 5 μL/min, 100 μm foil, graphite electrodes, rt, with curve fit to Hill equation (circles only) and indication of $Y_m^{max}$ as maximal microfluidic yield, $V_c$ as critical voltage, $n_g$ as gating coefficient.

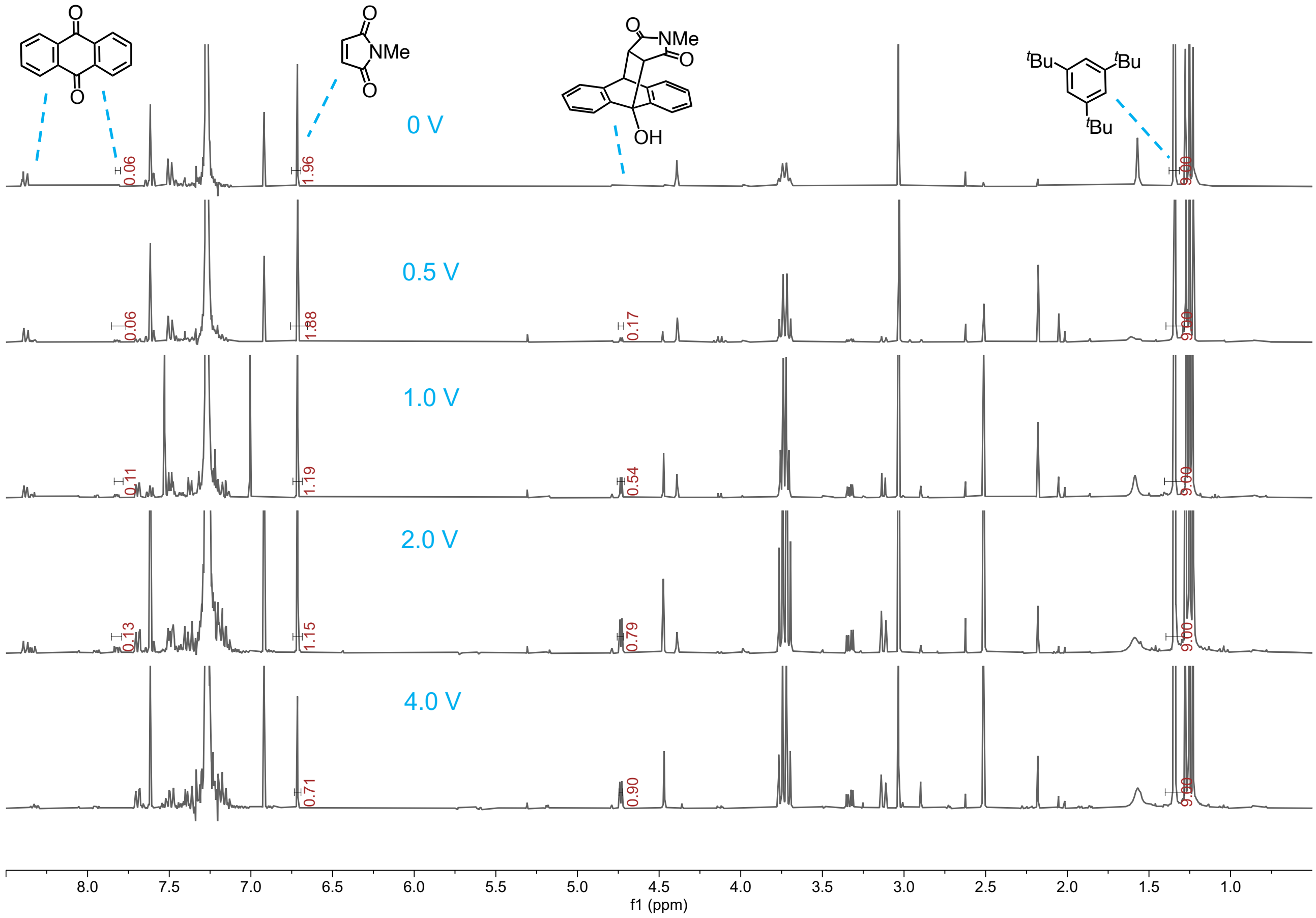


**Figure S55.** $^{1}H$ NMR spectra (400 MHz, $CDCl_3$) of reaction mixtures obtained from **14** (50 mM), **15** (75 mM) and Helmholtz components **40** and $Br^-$ (1 mM) in $CHCl_3$ (4 mL) after one passage through the microfluidic capacitor at 5 μL/min with $V$ = 0.0, 0.5, 1.0, 2.0 and 4.0 V (from top to bottom) applied, at rt.

**Table S26.** EFC with substrates **14** (50 mM) and **15** (75 mM) and Helmholtz components **40** and $Br^-$ in $CHCl_3$.[a]

| Entry | $V$ (V)[b] | $I$ (A)[c] | $Y_m^{\mathbf{18}}$ (%)[d] | $Y_m^{\mathbf{21}}$ (%)[e] | $Y_m^{\mathbf{24}}$ (%)[f] | *ee* (%)[g] |
|---|---|---|---|---|---|---|
| 1 | 0 | 0.000 | 0 | 0 | 1.5 | - |
| 2 | 0.5 | 0.000 | 17 | 0 | 1.5 | - |
| 3 | 1.0 | 0.000 | 54 | 0 | 2.8 | 15 |
| 4 | 2.0 | 0.000 | 79 | 0 | 3.2 | 15 |
| 5 | 4.0 | 0.000 | 90 | 0 | 0 | 15 |

[a]In microfluidic capacitor, flow rate 5 µL/min, 100 µm foil, graphite electrodes, with 1,3,5-tri-*tert*-butylbenzene as internal standard (IS), rt, flowrate 5 µL/min. [b]Applied voltage, in volt. [c]Steady-state current measured, in ampere. [d]Microfluidic yield of DA product **18**, in percent. [e]Microfluidic yield of Michael product **21**, in percent. [f]Microfluidic yield of oxidation product **24**, in percent. [g]The *ee* value of DA product **18**, in percent.

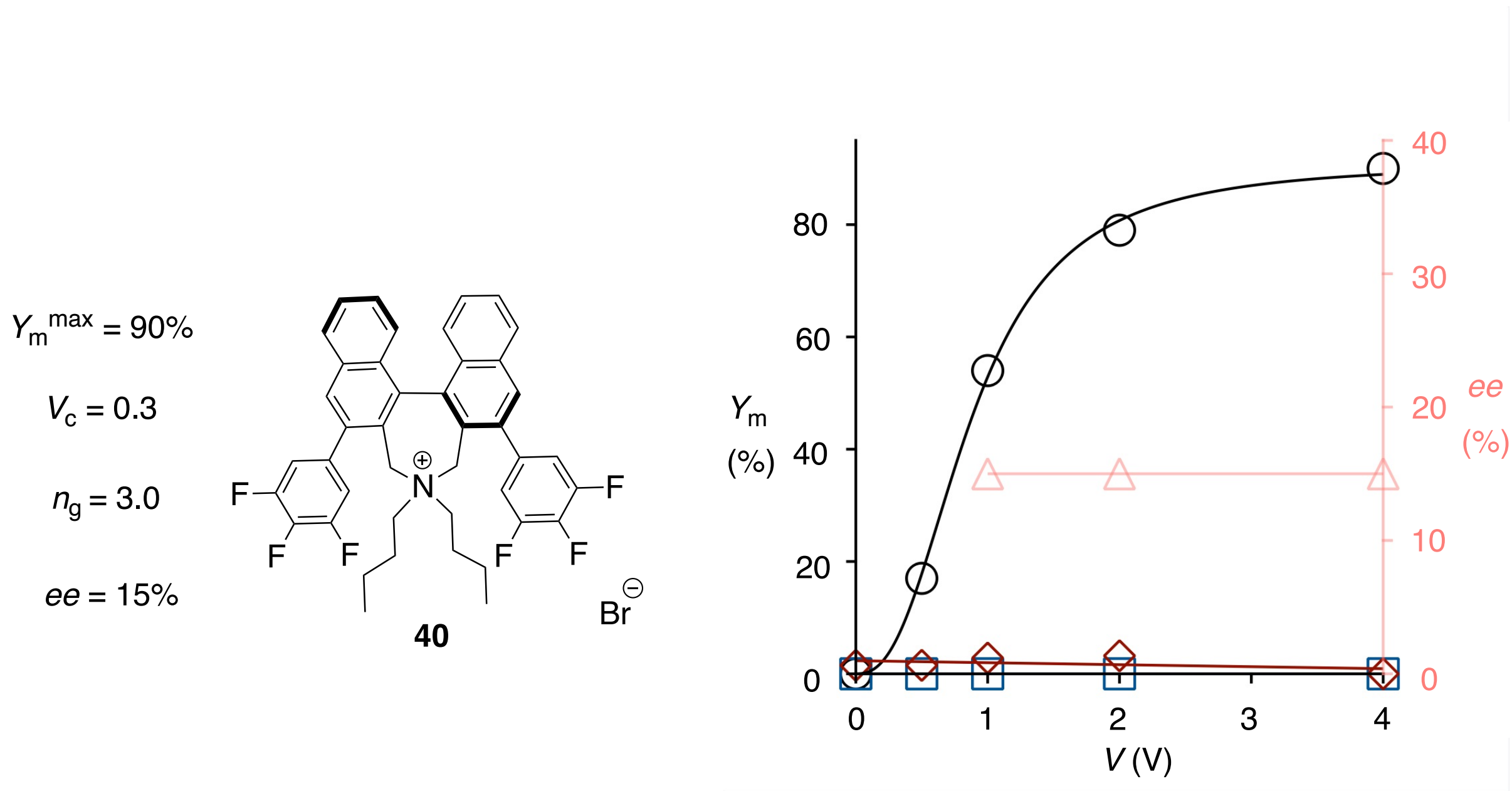


**Figure S56**. Dependence of microfluidic yield $Y_m$ (black, left, **18**: circles, **21**: squares, **24**: diamonds) and *ee* (right, red, **18**: triangles) on the applied voltage $V$ for substrates **14** (50 mM) and **15** (75 mM) with Helmholtz components **40** and $Br^-$ (1 mM, 2 mol%) in $CHCl_3$ (4 mL), flow rate 5 µL/min, 100 µm foil, graphite electrodes, rt, with curve fit to Hill equation (circles only) and indication of $Y_m^{max}$ as maximal microfluidic yield, $V_c$ as critical voltage, $n_g$ as gating coefficient and *ee* as enantioselectivity for **18**.

### 3.6. Chronoamperometry

In order to quantify the EEFs generated within the EDLs, CA measurements were performed. Under standard EFC conditions, one of the two graphite electrodes was configured as working electrode, while the other served as both the counter and reference electrode. The system was set up as described above. After the system reached a steady state, defined by a stable, uninterrupted, and bubble-free outflow of the reaction solution from the microfluidic reactor, CA measurements were carried out while maintaining continuous flow. A step potential was applied by imposing 1 V across the electrodes for 40 s to charge the EDL capacitor, followed by 0 V for 40 s to discharge it.

The charging/discharging process of the EDL capacitor can be modelled as an equivalent RC circuit (Figures S57-S60). For this circuit applies

$$V_{applied} = V_R + V_c = I(t)R + \frac{Q(t)}{C} = \frac{dQ}{dt}R + \frac{Q(t)}{C} \quad \text{(Eq S2)}$$

where $V_{\text{applied}}$ is the applied voltage, $V_{\text{R}}$ is the voltage across the resistance, $V_{\text{C}}$ is the voltage across the capacitor, *I(t)* is the current at time, *R* is the solution resistance, *Q(t)* is the accumulated charge, and *C* is the capacitance of the EDL.[S11]

Solving this differential equation Eq S2 yields

$$Q(t) = CV_{applied}\left(1 - e^{-\frac{t}{RC}}\right) \quad \text{(Eq S3)}$$

$$I(t) = \frac{dQ}{dt} = \frac{V_{applied}}{R}e^{-\frac{t}{RC}} \quad \text{(Eq S4)}$$

The transient decay curves of the *I*–*t* plots were fitted to the exponential function. Although multi-exponential decay is expected due to different EDL formation kinetics of multiple components in the mixture, only the initial, thus the most prominent, decay was selected for fitting

$$I(t) = ae^{-bt} \quad \text{(Eq S5)}$$

where $a = V_{\text{applied}}/R$, $b = 1/RC$. Thus, the capacitance can be determined from

$$C = \frac{a}{bV_{applied}} \quad \text{(Eq S6)}$$

when the charging process is complete ($t \to \infty$), the maximum stored charge could be obtained by Equation S3. The electric fields $E$ can be obtained by the Gauss's Law

$$E = \frac{Q_{max}}{\varepsilon_0 \varepsilon_r A} = \frac{CV_{applied}}{\varepsilon_0 \varepsilon_r A} \quad \text{(Eq S7)}$$

where $A$ is the surface of the posed to the microfluidic channel (12 $cm^2$), $\varepsilon_0$ is the dielectric constant of vacuum ($8.85 \times 10^{-12}$ F/m), $\varepsilon_r$ is the dielectric constant of the local medium.[S10]

Notably, the dielectric constant in the EDLs is expected to decrease under strong electric fields.[S11-S14] It has been reported that the saturated constants of various solvents become comparable under high-field conditions.[S12] Nevertheless, we used here the constants of $CHCl_3$ and DMSO in bulk solution: $CHCl_3$ = 4.8; DMSO = 47;[S15] the mixed = 15.35.

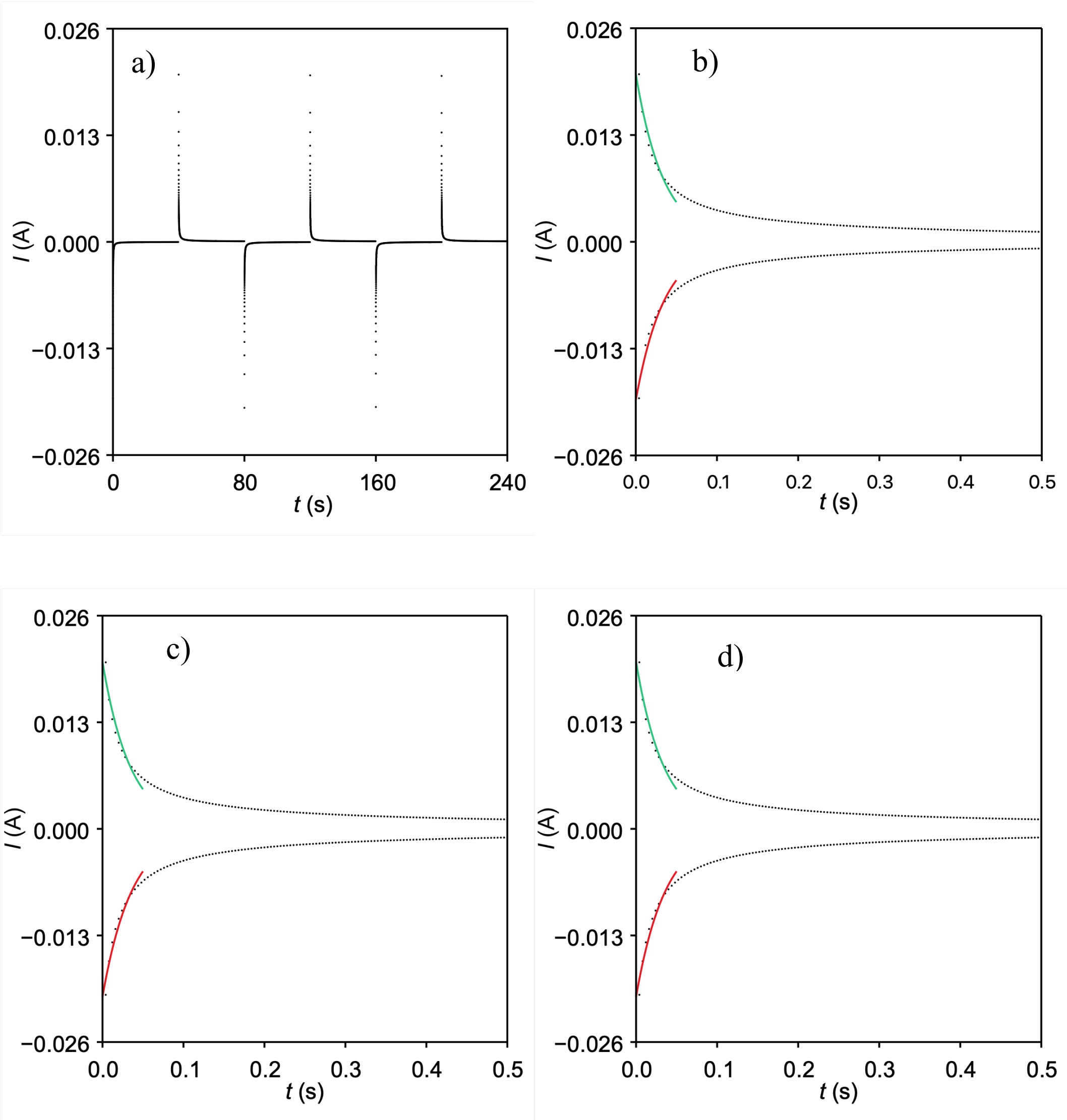


**Figure S57**. Chronoamperograms for **14** (50 mM), **15** (75 mM) and **27**-Cl (1 mM) in $CHCl_3$/DMSO 3:1 (4 mL) flowing through the microfluidic capacitor at 5 µL/min. a) Total 6 charge/discharge kinetics with pulse widths of 40 s. b) The first charge/discharge kinetics with fit to Equation S5. c) Same for the second charge/discharge kinetics. d) Same for the third charge/discharge kinetics.

**Table S27**. Chronoamperometry data for substrates **14** (50 mM) and **15** (75 mM) and Helmholtz components **27**-Cl (1 mM) in $CHCl_3$/DMSO 3:1 for charge (top) and discharge (bottom).[a]

| $a^b$ | $b^b$ | $C$ (mF)[c] | $E$ (V/nm)[d] | $C_{av}$ (mF)[e] | $E_{av}$ (V/nm)[f] |
|---|---|---|---|---|---|
| 19.3 ± 0.86 ×10$^{-3}$ | 28.4 ± 2.2 | 0.68 ± 0.05 | 4.2 ± 0.03 | charge: | charge: |
| 20.5 ± 0.84 ×10$^{-3}$ | 27.5 ± 2.0 | 0.75 ± 0.05 | 4.6 ± 0.03 | 0.72 ± 0.04 | 4.4± 0.10 |
| 20.3 ± 0.83 ×10$^{-3}$ | 27.3 ± 2.0 | 0.74 ± 0.05 | 4.5 ± 0.03 | | |
| 20.5 ± 0.97 ×10$^{-3}$ | 28.9 ± 2.3 | 0.71 ± 0.05 | 4.4 ± 0.03 | discharge: | discharge: |
| 20.4 ± 0.96 ×10$^{-3}$ | 28.8 ± 2.4 | 0.71 ± 0.05 | 4.4 ± 0.03 | 0.71 ± 0.01 | 4.4 ± 0.10 |
| 20.2 ± 0.96 ×10$^{-3}$ | 28.7 ± 2.3 | 0.70 ± 0.05 | 4.3 ± 0.03 | | |

[a]Analysis results for chronoamperogram in Figure S57. [b]Values ± uncertainties from curve fits to exponential decay. [c]Capacitance ± uncertainties, calculated using Equation S6. [d]Electric field ± uncertainties, calculated using Equation S7. [e]Average capacitance ± errors from three charge/discharge kinetics. [f]Average electric field ± errors from three charge or discharge kinetics.

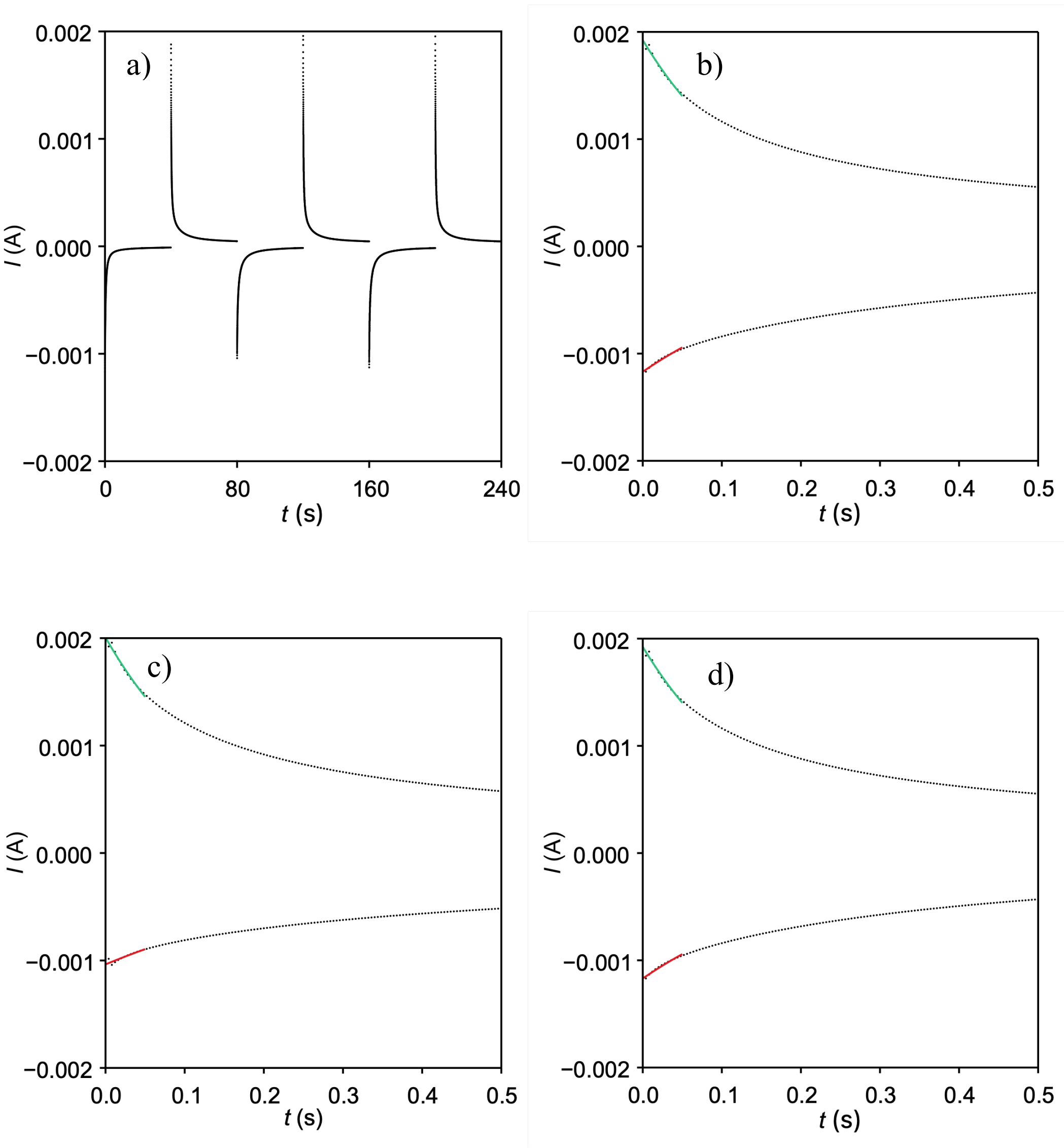


**Figure S58**. Chronoamperograms for **14** (50 mM), **15** (75 mM) in $CHCl_3$/DMSO 3:1 (4 mL) flowing through the microfluidic capacitor at 5 μL/min. a) Total 6 charge/discharge kinetics with pulse widths of 40 s. b) The first charge/discharge kinetics with fit to Equation S5. c) Same for the second charge/discharge kinetics. d) Same for the third charge/discharge kinetics.

**Table S28**. Chronoamperometry data for substrates **14** (50 mM) and **15** (75 mM) in $CHCl_3$/DMSO 3:1 for charge (top) and discharge (bottom).[a]

| $a$[b] | $b$[b] | $C$ (mF)[c] | $E$ (V/nm)[d] | $C_{av}$ (mF)[e] | $E_{av}$ (V/nm)[f] |
|---|---|---|---|---|---|
| 1.2 ± 0.01 $\times 10^{-3}$ | 4.3 ± 0.19 | 0.28 ± 0.01 | 1.7 ± 0.01 | charge: | charge: |
| 1.0 ± 0.01 $\times 10^{-3}$ | 2.9 ± 0.36 | 0.34 ± 0.04 | 2.0 ± 0.02 | 0.32 ± 0.04 | 1.9 ± 0.20 |
| 1.1 ± 0.01 $\times 10^{-3}$ | 3.2 ± 0.35 | 0.34 ± 0.04 | 2.0 ± 0.02 | | |
| 1.9 ± 0.02 $\times 10^{-3}$ | 6.3 ± 0.29 | 0.30 ± 0.01 | 1.8 ± 0.01 | discharge: | discharge: |
| 2.0 ± 0.01 $\times 10^{-3}$ | 6.4 ± 0.28 | 0.31 ± 0.01 | 1.9 ± 0.01 | 0.31 ± 0.01 | 1.9 ± 0.10 |
| 2.1 ± 0.02 $\times 10^{-3}$ | 6.6 ± 0.28 | 0.32 ± 0.01 | 1.9 ± 0.01 | | |

[a]Analysis results for chronoamperometry data in Figure S58. [b]Values ± uncertainties from curve fits to exponential decay. [c]Capacitance ± uncertainties, calculated using Equation S6. [d]Electric field ± uncertainties, calculated using Equation S7. [e]Average capacitance ± errors from three charge/discharge kinetics. [f]Average electric field ± errors from three charge or discharge kinetics.

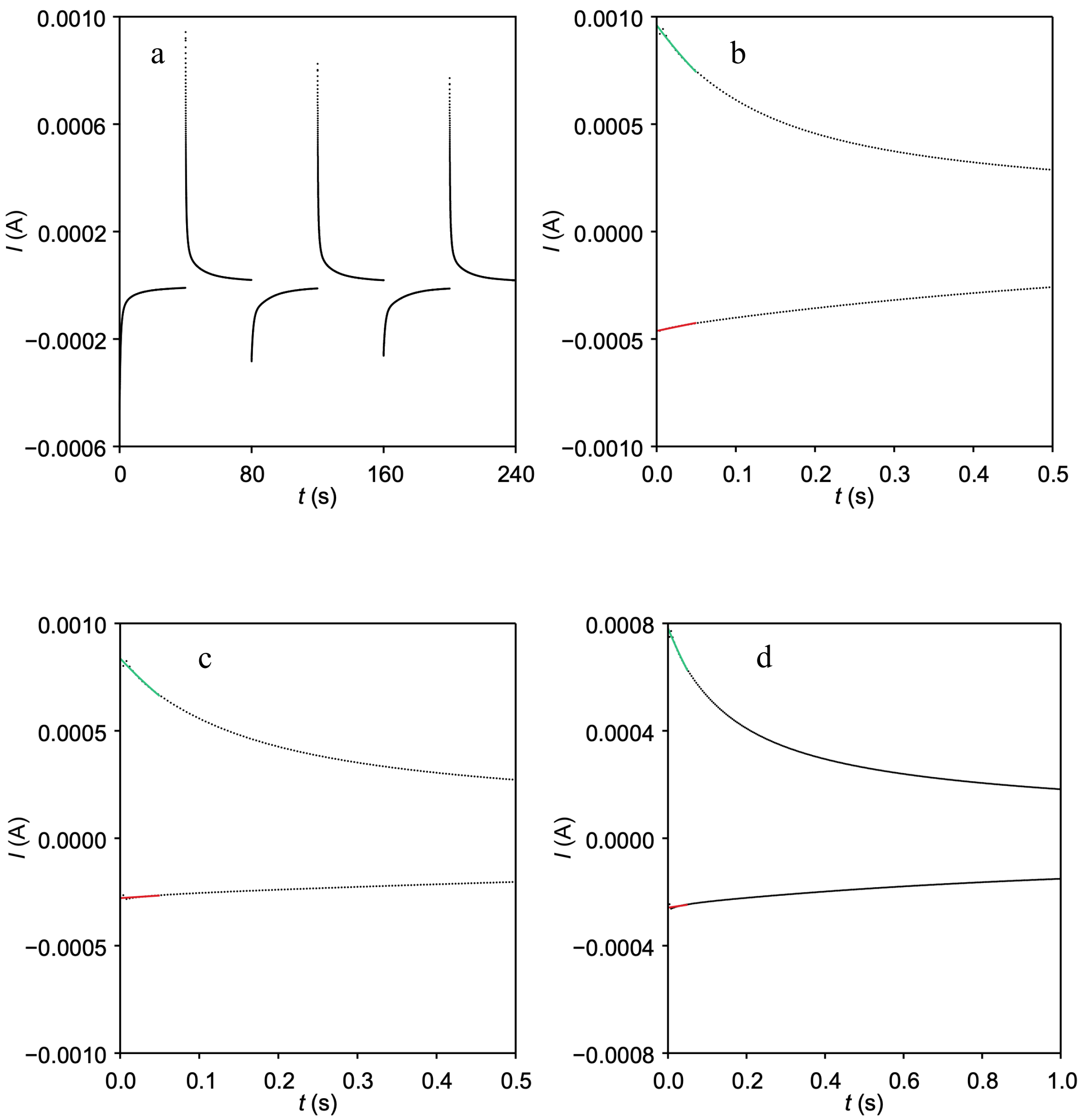


**Figure S59**. Chronoamperograms for $CHCl_3$/DMSO 3:1 (4 mL) flowing through the microfluidic capacitor at 5 μL/min. a) Total 6 charge/discharge kinetics with pulse widths of 40 s. b) The first charge/discharge kinetics with fit to Equation S5. c) Same for the second charge/discharge kinetics. d) Same for the third charge/discharge kinetics.

**Table S29**. Chronoamperometry data for $CHCl_3$/DMSO 3:1 (4 mL) for charge (top) and discharge (bottom).[a]

| $a^b$ | $b^b$ | $C$ (mF) [c] | $E$ (V/nm)[d] | $C_{av}$ (mF)[e] | $E_{av}$ (V/nm)[f] |
|---|---|---|---|---|---|
| 4.6 ± 0.07 ×10$^{-4}$ | 1.7 ± 0.06 | 0.27 ± 0.01 | 1.6 ± 0.01 | charge: | charge: |
| 2.7 ± 0.03 ×10$^{-4}$ | 0.87 ± 0.37 | 0.31 ± 0.13 | 1.9 ± 0.01 | 0.29 ± 0.02 | 1.8 ± 0.10 |
| 2.6 ± 0.03 ×10$^{-4}$ | 0.85 ± 0.37 | 0.31 ± 0.13 | 1.9 ± 0.08 | | |
| 9.6 ± 0.07 ×10$^{-4}$ | 5.2 ± 0.25 | 0.18 ± 0.01 | 1.1 ± 0.01 | discharge: | discharge: |
| 8.4 ± 0.06 ×10$^{-4}$ | 4.7 ± 0.26 | 0.18 ± 0.01 | 1.1 ± 0.01 | 0.18 ± 0.01 | 1.1 ± 0.10 |
| 7.8 ± 0.06 ×10$^{-4}$ | 4.5 ± 0.26 | 0.17 ± 0.03 | 1.0 ± 0.02 | | |

[a]Analysis results for chronoamperometry data in Figure S59. [b]Values ± uncertainties from curve fits to exponential decay. [c]Capacitance ± uncertainties, calculated using Equation S6. [d]Electric field ± uncertainties, calculated using Equation S7. [e]Average capacitance ± errors from three charge/discharge kinetics. [f]Average electric field ± errors from three charge or discharge kinetics.

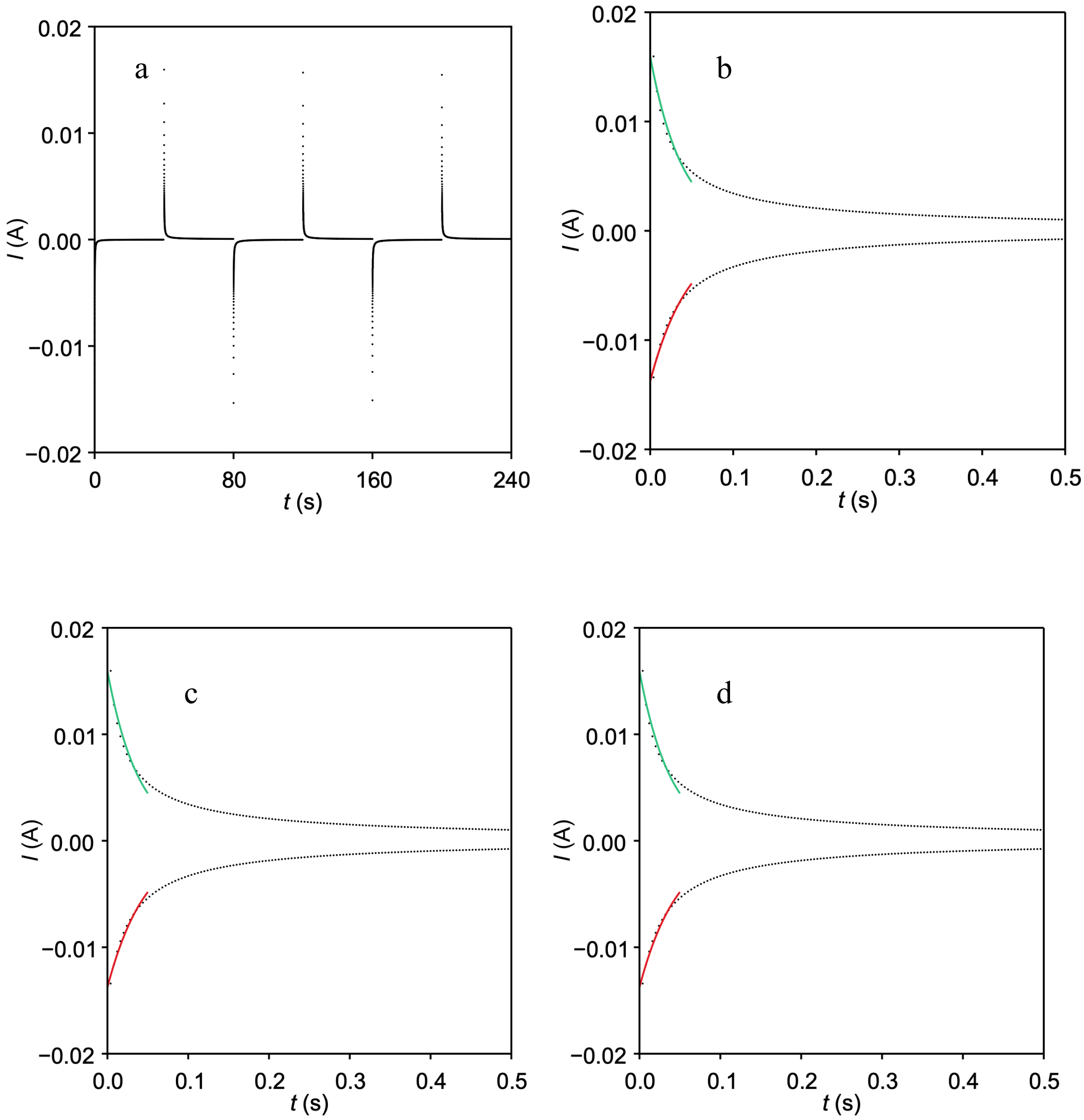


**Figure S60**. Chronoamperograms for **27**-Cl (1 mM) in $CHCl_3$/DMSO 3:1 (4 mL) flowing through the microfluidic capacitor at 5 µL/min. a) Total 6 charge/discharge kinetics with pulse widths of 40 s. b) The first charge/discharge kinetics with fit to Equation S5. c) Same for the second charge/discharge kinetics. d) Same for the third charge/discharge kinetics.

**Table S30**. Chronoamperometry data for Helmholtz components **27**-Cl (1 mM) in $CHCl_3$/DMSO 3:1 for charge (top) and discharge (bottom).[a]

| $a$[b] | $b$[b] | $C$ (mF)[c] | $E$ (V/nm)[d] | $C_{av}$ (mF)[e] | $E_{av}$ (V/nm)[f] |
|---|---|---|---|---|---|
| 13.7 ± 0.32 ×10$^{-3}$ | 21.0 ± 1.1 | 0.65 ± 0.03 | 4.0 ± 0.02 | charge: | charge: |
| 15.4 ± 0.54 ×10$^{-3}$ | 22.9 ± 1.6 | 0.67 ± 0.05 | 4.1 ± 0.03 | 0.66 ± 0.01 | 4.0 ± 0.10 |
| 15.1 ± 0.53 ×10$^{-3}$ | 22.8 ± 1.6 | 0.66 ± 0.05 | 4.0 ± 0.03 | | |
| 15.9 ± 0.03 ×10$^{-3}$ | 25.9 ± 1.9 | 0.62 ± 0.05 | 3.8 ± 0.03 | discharge: | discharge: |
| 15.6 ± 0.03 ×10$^{-3}$ | 25.3 ± 1.9 | 0.62 ± 0.05 | 3.8 ± 0.03 | 0.62 ± 0.01 | 3.8 ± 0.10 |
| 15.4 ± 0.02 ×10$^{-3}$ | 25.1 ± 1.9 | 0.61 ± 0.05 | 3.7 ± 0.03 | | |

[a]Analysis results for chronoamperometry data in Figure S60. [b]Values ± uncertainties from curve fits to exponential decay. [c]Capacitance ± uncertainties, calculated using Equation S6. [d]Electric field ± uncertainties, calculated using Equation S7. [e]Average capacitance ± errors from three charge/discharge kinetics. [f]Average electric field ± errors from three charge or discharge kinetics.

To evaluate whether the electrochemical redox reaction accounts for substrate transformation, the amount of electrons transferred during EFC and the resulting theoretical yield were estimated based on the experimentally measured current under 1 V. Assuming an ideal electrochemical redox process, in which all transferred charge is exclusively consumed by the target redox reaction without other contributions, such as competing redox reactions, electrolyte decomposition, capacitive charging, or mass transport limitations, the theoretical yield can be determined according to the formula:

$$Y = \frac{n_e}{n_{sub}} = \frac{\frac{Q}{F}}{C_{sub}^m V_m} = \frac{I\frac{V_m}{U}}{C_{sub}^m V_m F} = \frac{I}{C_{sub}^m U F} = \frac{24 \times 10^{-6}}{0.05 \times \frac{5}{60} \times 10^{-6} \times 96485} = 6\% \qquad \text{(Eq S8)}$$

where $I$ is the measured current, $I = 24$ μA, $Q$ is the total charge passed (C), $V_m$ is the reactor volume, $U$ is flow rate (5 μL/min), $C_{sub}^{m}$ is substrate concentration (50 mM), $n_e$ is the amount of electrons (mol), $n_{sub}$ is the amount of substrate (mol), $F$ is the Faraday constant (96485 C/mol). The obtained $Y$ = 6% is much lower than the experimental $Y_m$ of product **18** (71%), supporting the insignificant contribution of redox reaction to the product formation.

### 3.7. Radical Scavenger Control Experiments

A solution of **27**·Cl (1 mM), 1,3,5-tri-*tert*-butylbenzene as internal standard (IS), 2,6-di-*tert*-butyl-*p*-cresol **58** (50 mM), anthrone **14** (50 mM), and *N*-methylmaleimide **15** (75 mM) in $CHCl_3$/DMSO 3:1 was loaded in a syringe. The solution was infused at 5 μL/min using a syringe pump into the microfluidic capacitor under constant voltage. The initial 0.36 mL, corresponding to three reactor volumes, was discarded before collecting each sample to ensure that a steady state of the system had been reached at the desired voltage. Then, 150 μL samples were collected over 30 min. Samples were diluted with additional $CDCl_3$ and the $^1$H NMR spectrum was recorded right after every experiment. Yields ($Y_m$) were determined by comparing the integrals of the signal to that of the IS. All experiments were conducted under $I = 0.000$ A.

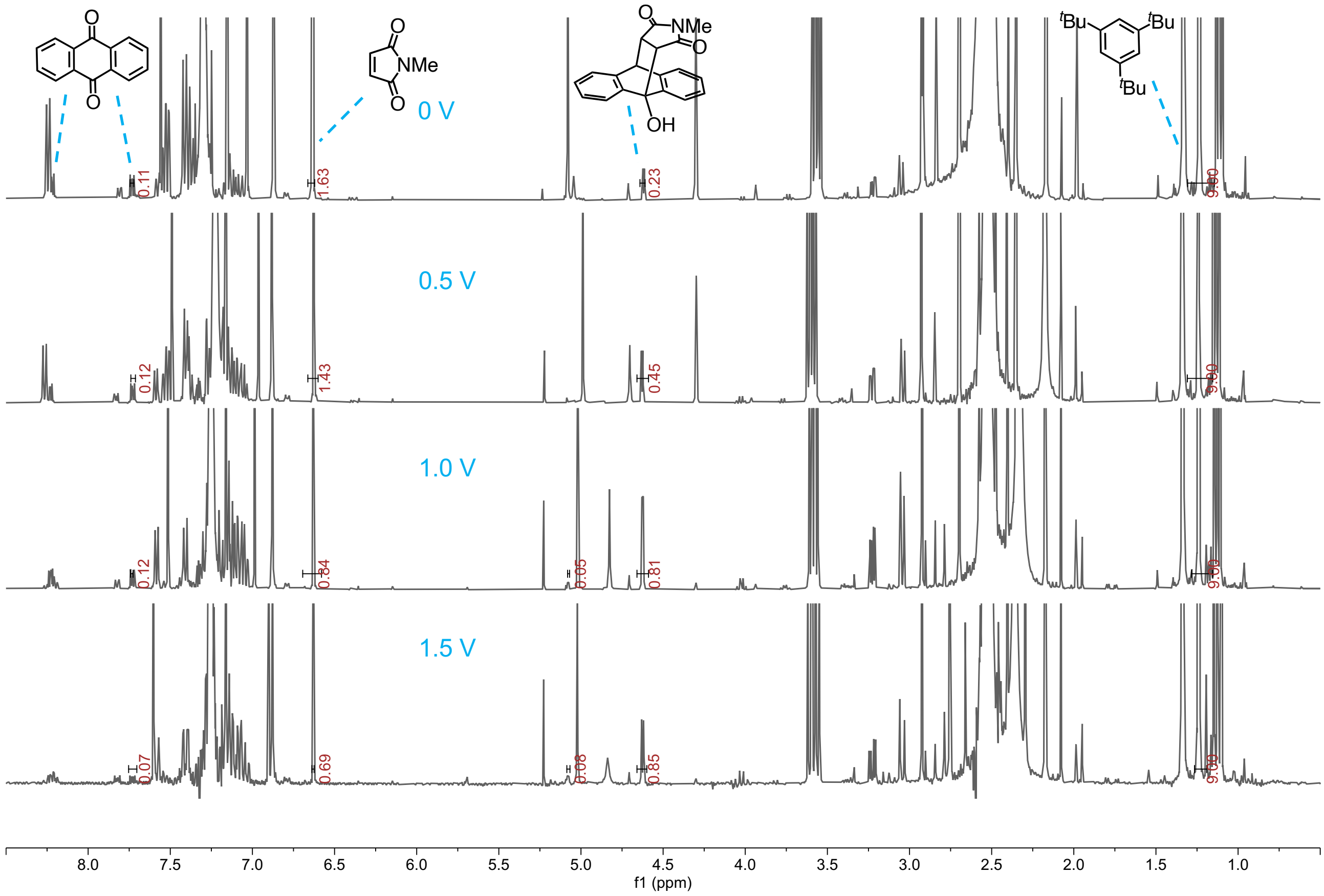


**Figure S61.** $^{1}$H NMR spectra (400 MHz, $CDCl_3$) of reaction mixtures obtained from **14** (50 mM), **15** (75 mM), **58** (50 mM), and Helmholtz components **27** and $Cl^-$ in $CHCl_3$/DMSO 3:1 (4 mL) after one passage through the microfluidic capacitor at 5 µL/min with $V$ = 0.0, 0.5, 1.0 and 1.5 V (from top to bottom) applied, at rt.

**Table S31.** EFC with **14** (50 mM), **15** (75 mM), **58** (50 mM) and Helmholtz components **27** and $Cl^-$ (1 mM) in $CHCl_3$/DMSO 3:1.[a]

| Entry | *V* (V)[b] | *I* (A)[c] | $Y_m$**18** (%)[d] | $Y_m$**21** (%)[e] | $Y_m$**24** (%)[f] | *ee* (%)[g] |
|---|---|---|---|---|---|---|
| 1 | 0 | 0.000 | 23 | 0 | 2.8 | - |
| 2 | 0.5 | 0.000 | 45 | 0 | 3.0 | - |
| 3 | 1.0 | 0.000 | 81 | 5 | 3.0 | - |
| 4 | 1.5 | 0.000 | 85 | 8 | 1.0 | - |

[a]In microfluidic capacitor, flow rate 5 µL/min, 100 µm foil, graphite electrodes, with 1,3,5-tri-*tert*-butylbenzene as internal standard (IS), rt, flowrate 5 µL/min. [b]Applied voltage, in volt. [c]Steady-state current measured, in ampere. [d]Microfluidic yield of DA product **18**, in percent. [e]Microfluidic yield of Michael product **21**, in percent. [f]Microfluidic yield of oxidation product **24**, in percent. [g]The *ee* value of DA product **18**, in percent.

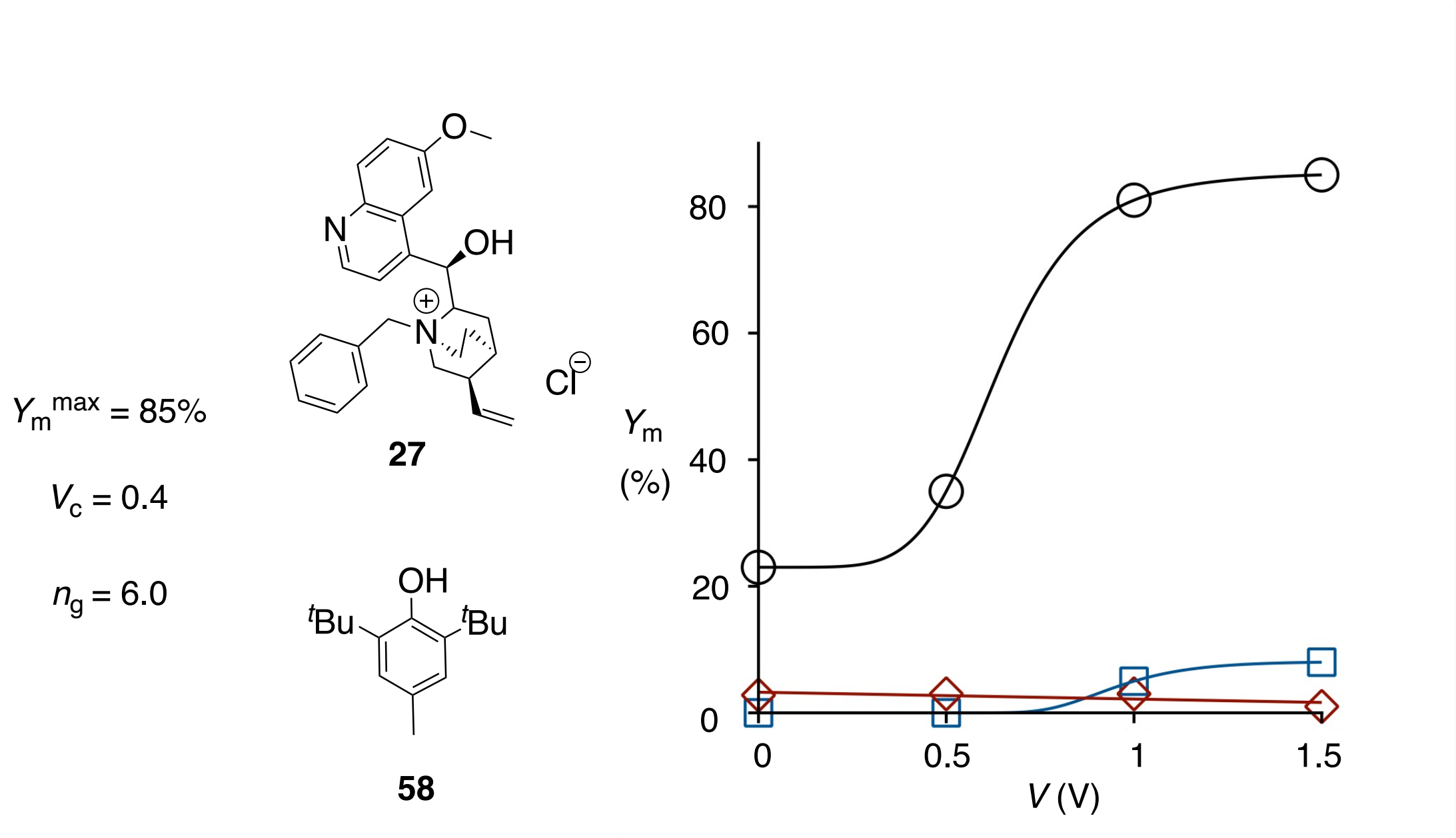


**Figure S62.** Dependence of microfluidic yield $Y_m$ (**18**: circles, **21**: squares, **24**: diamonds) on the applied voltage $V$ for substrates **14** (50 mM), **15** (75 mM) and **58** (50 mM) with Helmholtz components **27** and $Cl^-$ (1 mM, 2 mol%) in $CHCl_3$/DMSO 3:1, flow rate 5 µL/min, 100 µm foil, graphite electrodes, rt, with curve fit to Hill equation (circles only) and indication of $Y_m^{max}$ as maximal microfluidic yield, $V_c$ as critical voltage, $n_g$ as gating coefficient.

## 4. Supporting References


(S1) Paraja, M.; Matile, S. *JACS Au* **2026**, 6, 2337-2344.

(S2) Uemae, K.; Masuda, S.; Yamamoto, Y. *J. Chem. Soc., Perkin Trans.* **2001**, *1*, 1002-1006.

(S3) Byrne, A. J.; Bright, S. A.; McKeown, J. P.; O'Brien, J. E.; Twamley, B.; Fayne, D.; Williams, D. C.; Meegan, M. *J. Pharmaceuticals* **2020**, *13*, 16-74.

(S4) Chuard, N.; Gasparini, G.; Moreau, D.; Lörcher, S.; Palivan, C.; Meier, W.; Sakai, N.; Matile, S. *Angew. Chem. Int. Ed.* **2017**, *56*, 2947-2950.

(S5) Romanov-Michailidis, F.; Romanova-Michaelides, M.; Pupier, M.; Alexakis, A. *Chem. Eur. J.* **2015**, *21*, 5561-5583.

(S6) Saha, S.; Schneider, C. *Chem. Eur. J.* **2015**, *21*, 2348-235.

(S7) Zhang, P. L.; Tsuji, N.; Ouyang, J.; List, B. *J. Am. Chem. Soc.* **2021**, *143*, 675-680.

(S8) Yue, Y., Turlington, M., Yu, X. Q., Pu, L. *J. Org. Chem*. **2009**, *74*, 8681-8689.

(S9) Wu, T. R.; Shen, L. X.; Chong, J. M. *Org. Lett.* **2004**, *6*, 2701-2704.

(10). Sevim, S.; Sanchis-Gual, R.; Franco, C.; Aragonès, A. C.; Darwish, N.; Kim, D.; Picca, R. A.; Nelson, B. J.; Ruiz, E.; Pané, S.; Díez-Pérez, I.; Puigmartí-Luis, J. *Nat. Commun.* **2024**, *15*, 790.

(11) Schott, C. M.; Schneider, P. M.; Song, K.-T.; Yu, H.; Götz, R.; Haimerl, F.; Gubanova, E.; Zhou, J.; Schmidt, T. O.; Zhang, Q.; Alexandrov, V.; Bandarenka, A. S. *Chem. Rev.* **2024**, *124*, 12391-12462.

(12) Hou, Y.; Aoki, K. J.; Chen, J.; Nishiumi, T. *J. Phys. Chem. C* **2014**, *118*, 10153-10158.

(13) Wang, X.; Liu, K.; Wu, J. *J. Chem. Phys.* **2021**, *154*, 124701-124710.

(14) Shaik, S. *ACS Phys. Chem. Au.* **2024**, *4*, 191-201.

(15) Storm, K. R.; Skoruppa, E.; Pritzl, S. D.; Kolbeck, P. J.; Vanderlinden, W.; Schiessel, H.; Lipfert, J. *Biophys. J.* **2025**, *124*, 2639-2654.

(16) Klaper, M.; Wessig, P.; Linker, T. *Chem. Commun.* **2016**, *52*, 1210-1213.

## 5. NMR Spectra

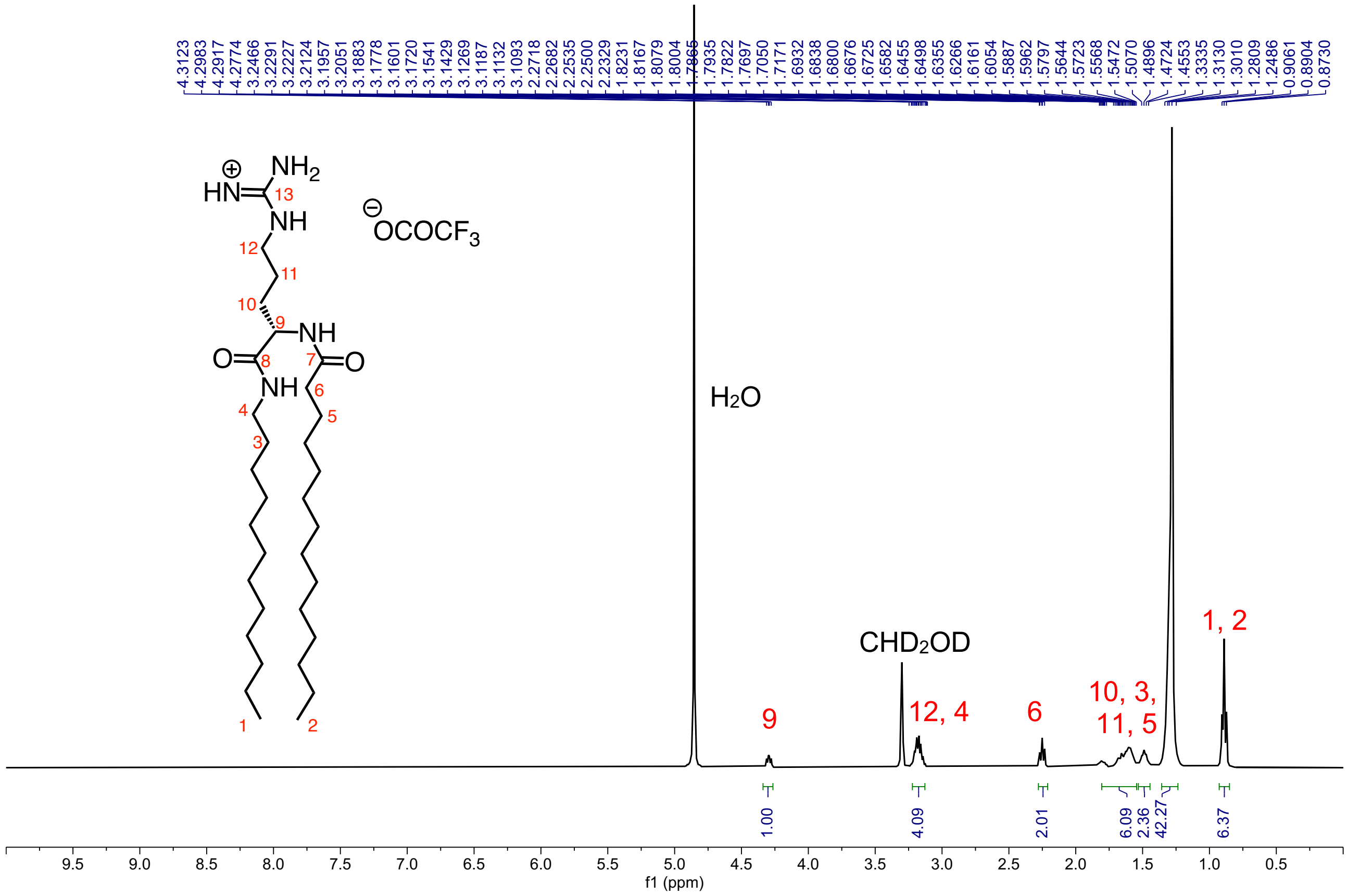

**Figure S63**. 400 MHz $^1$H NMR spectra of ion pair **38** and **39** in $CD_3OD$.

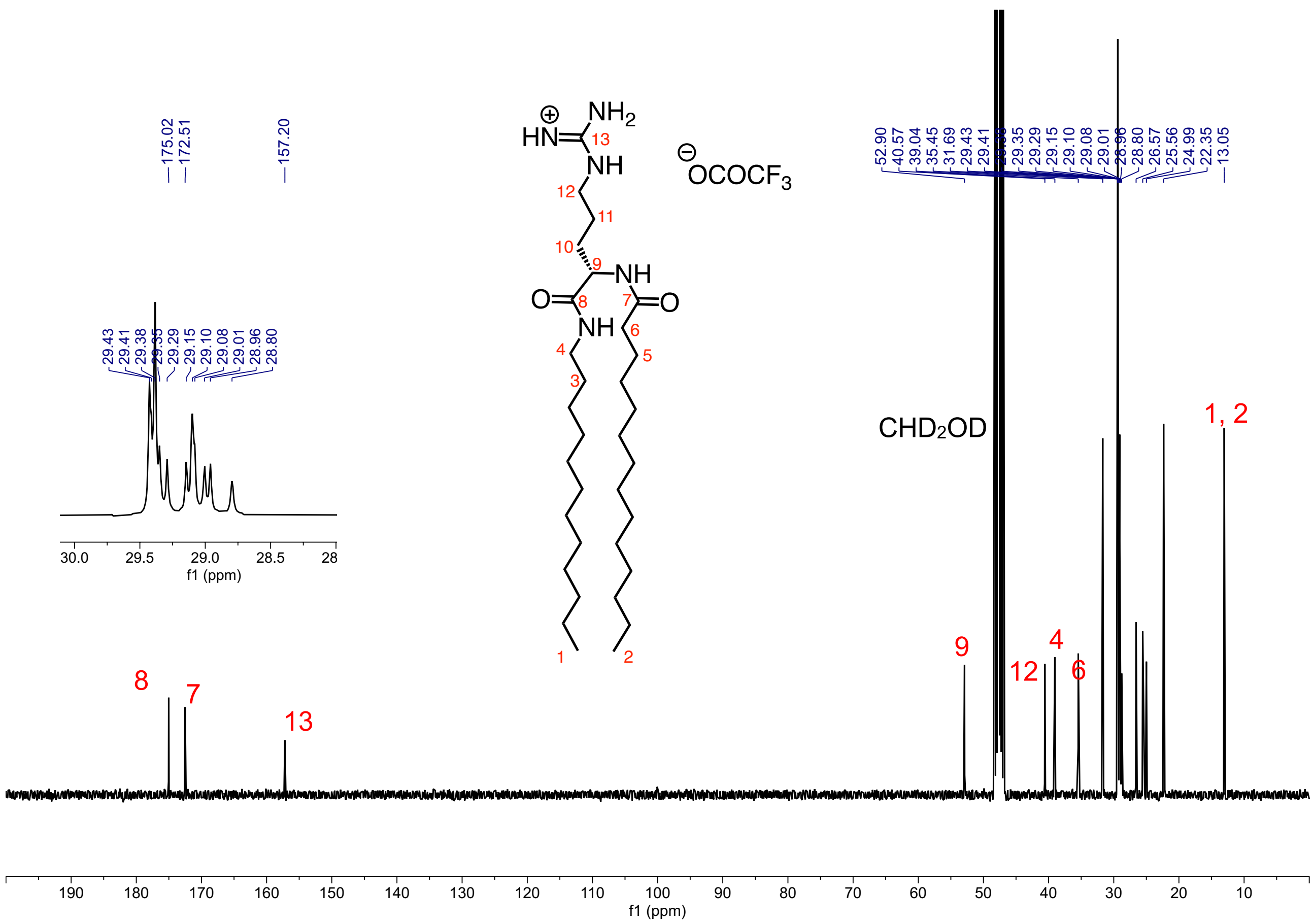


**Figure S64.** 101 MHz $^{13}C$ NMR spectra of ion pair **38** and **39** in $CD_3OD$.

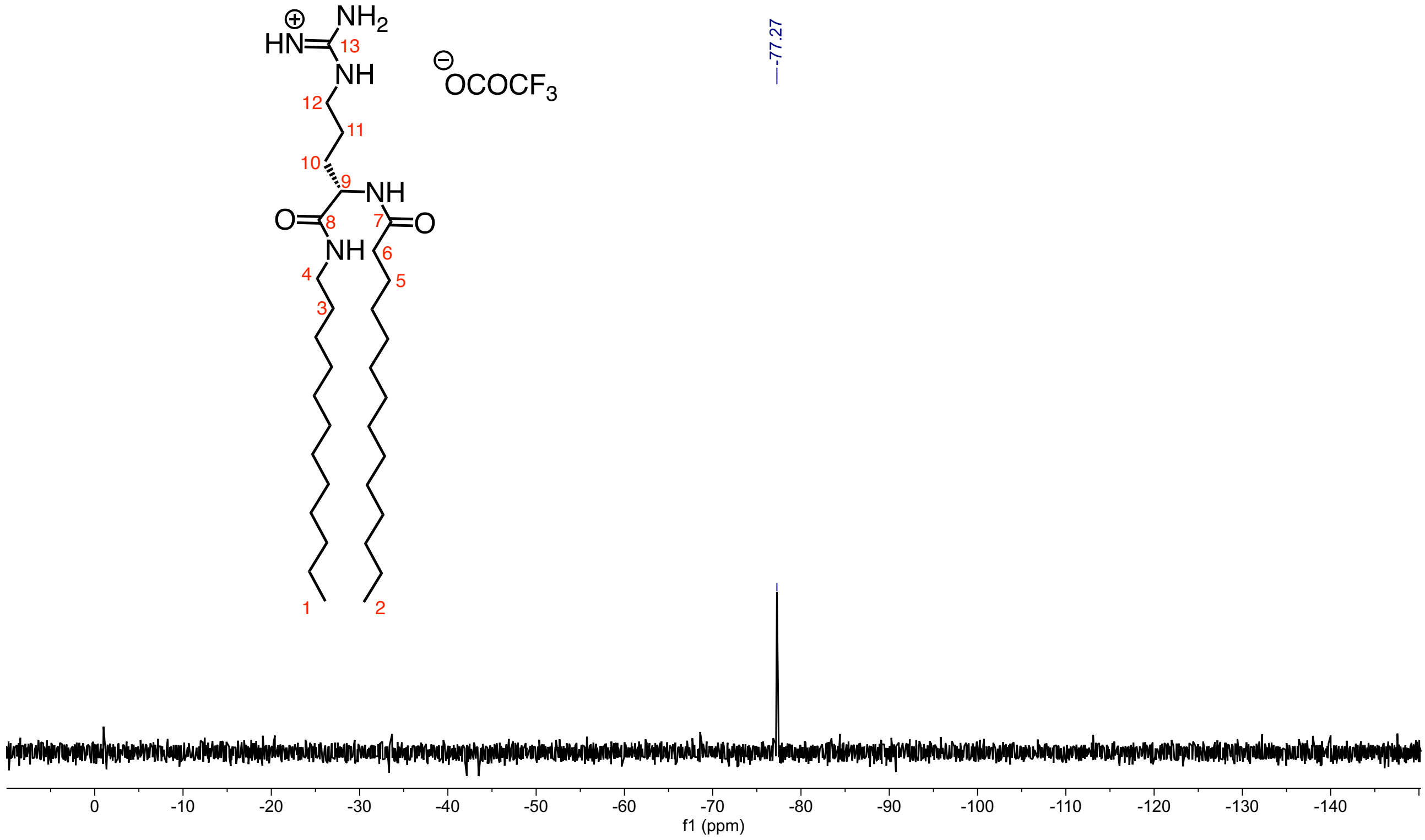


**Figure S65**. 282 MHz $^{19}F$ NMR spectra of ion pair **38** and **39** in $CD_3OD$**.**

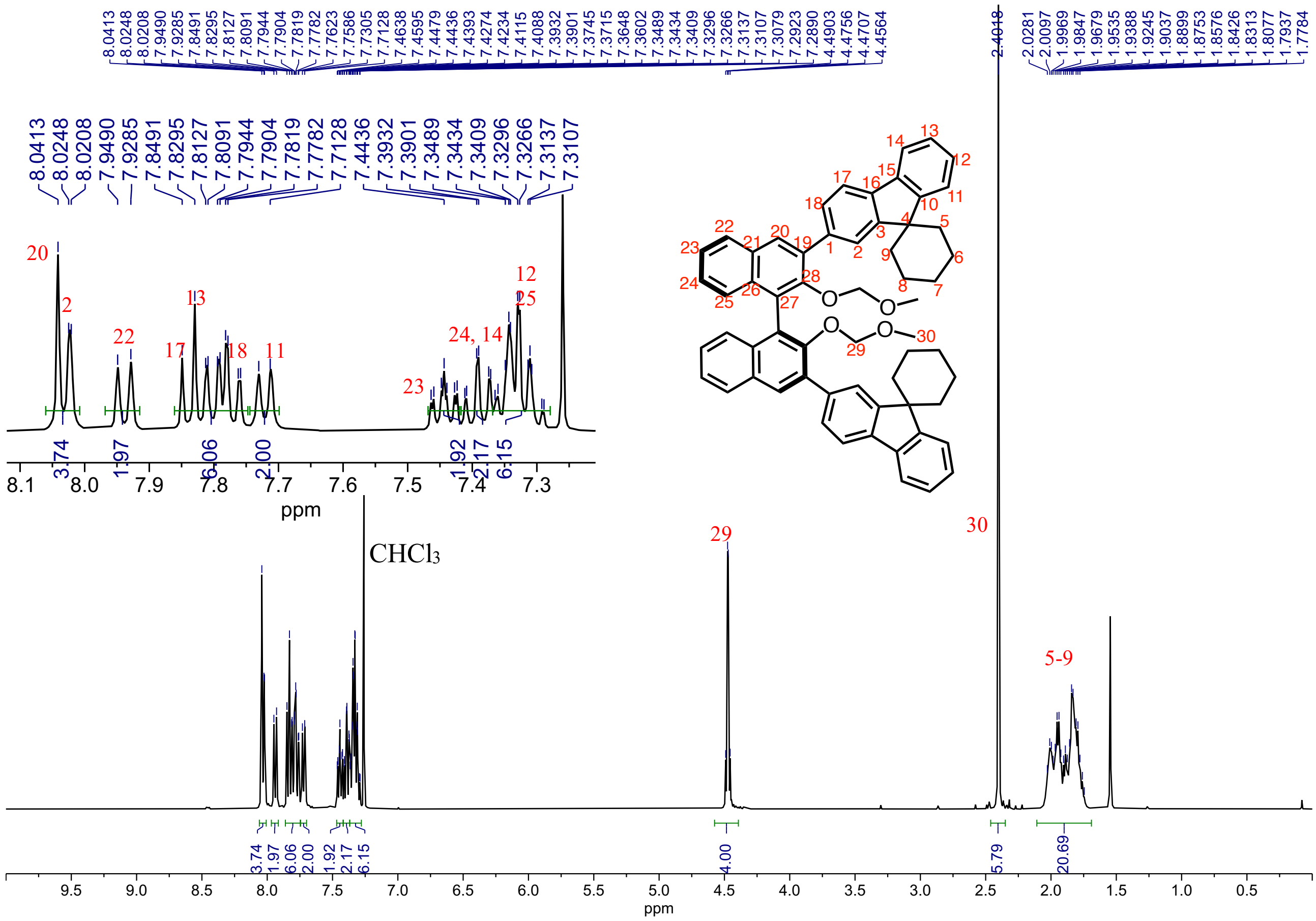

**Figure S66**. 400 MHz $^{1}H$ NMR spectra **52** in $CDCl_3$.

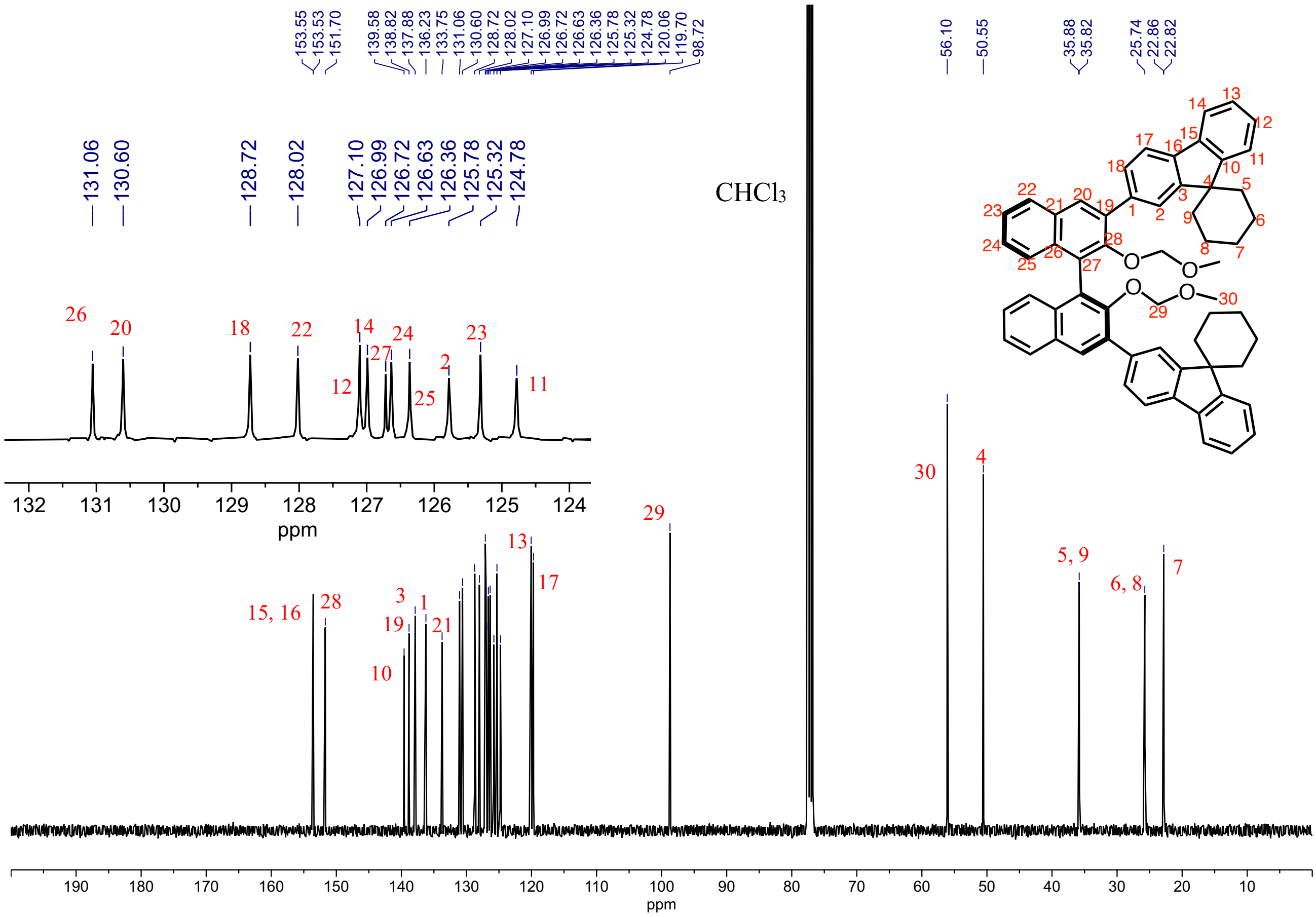


**Figure S67**. 101 MHz $^{13}C$ NMR spectra **52** in $CDCl_3$.

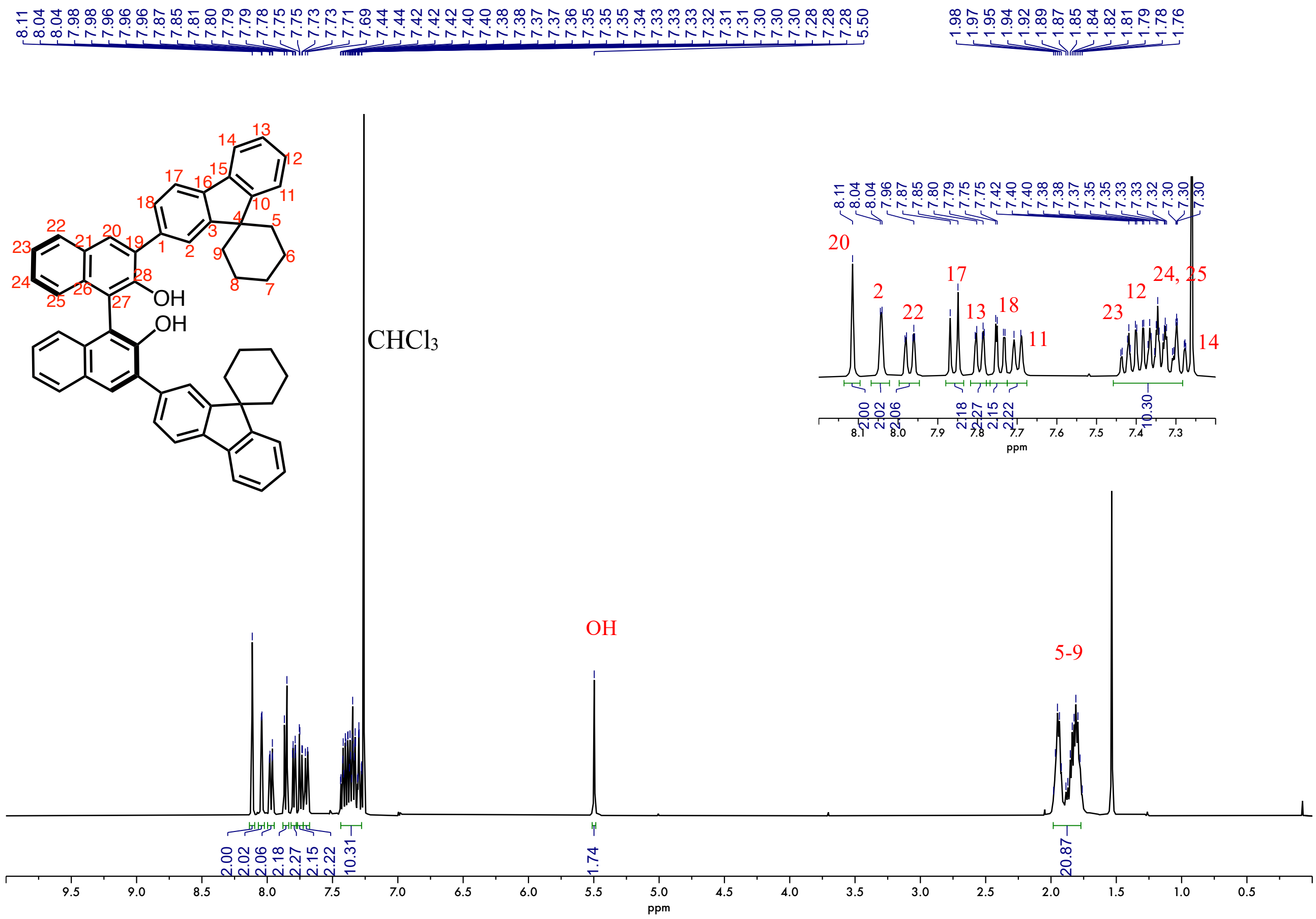

**Figure S68**. 400 MHz $^{1}$H NMR spectra **53** in $CDCl_3$.

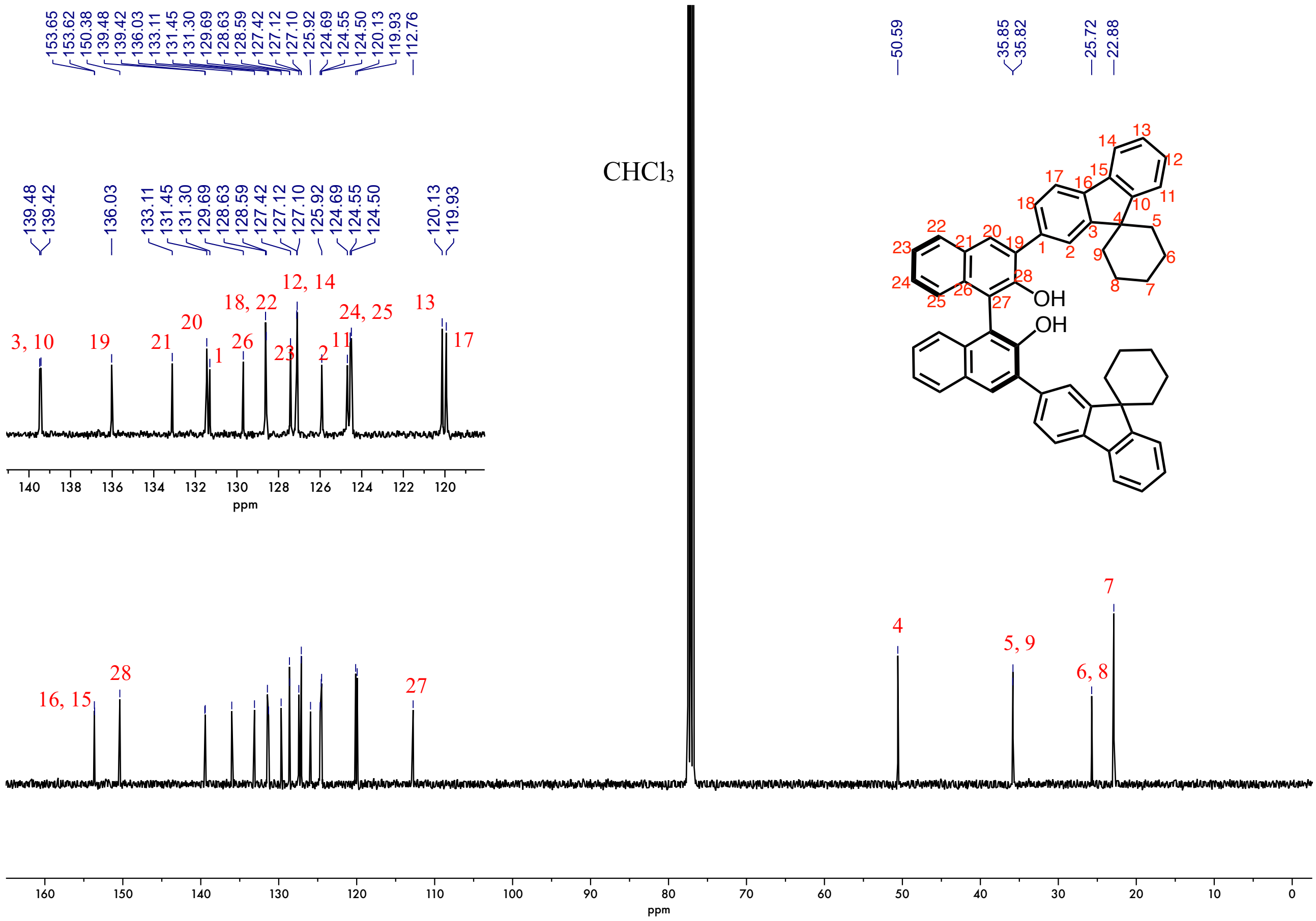


**Figure S69**. 101 MHz $^{13}$C NMR spectra **53** in $CDCl_3$.

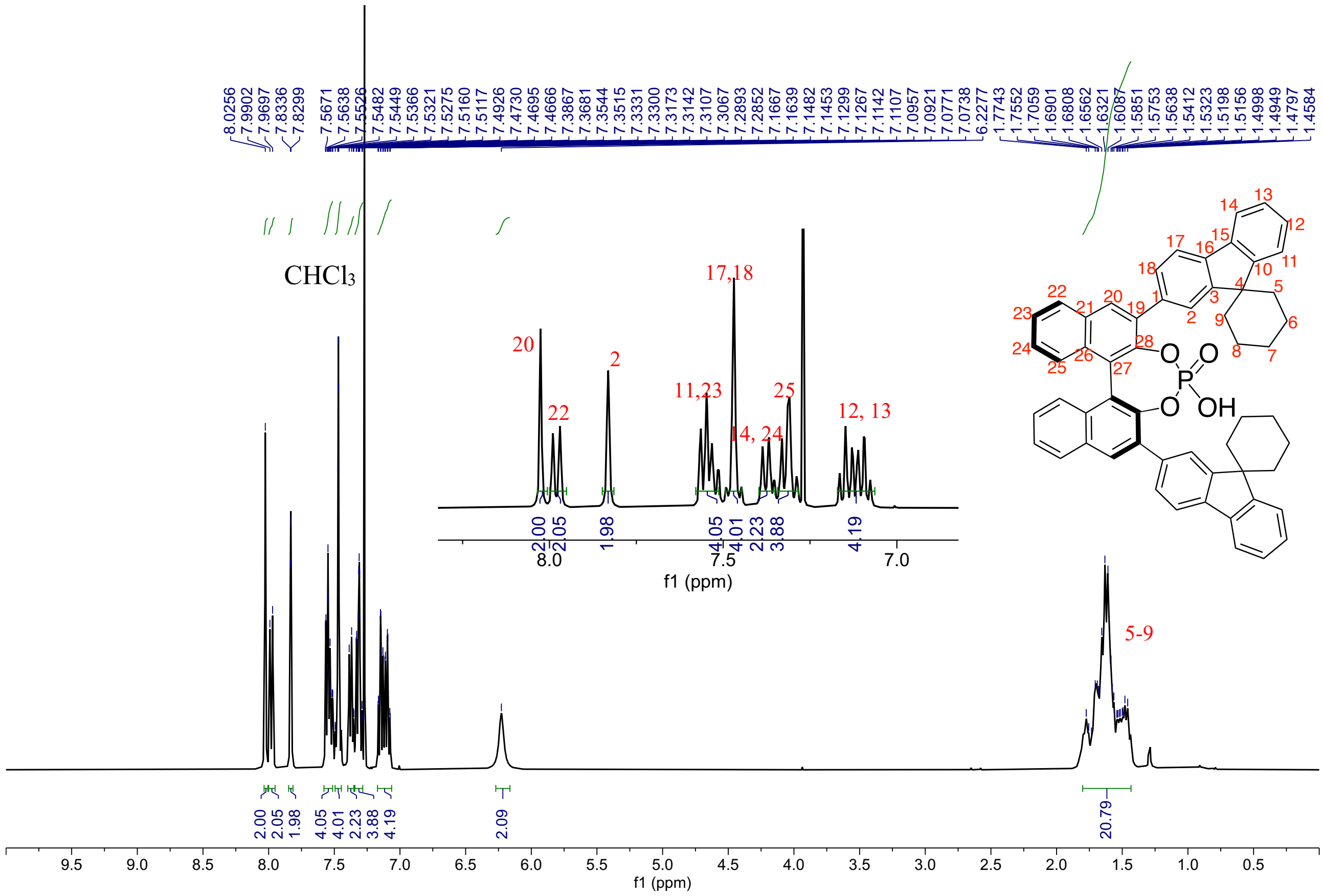

**Figure S70**. 400 MHz $^{1}$H NMR spectra **54** in $CDCl_3$.

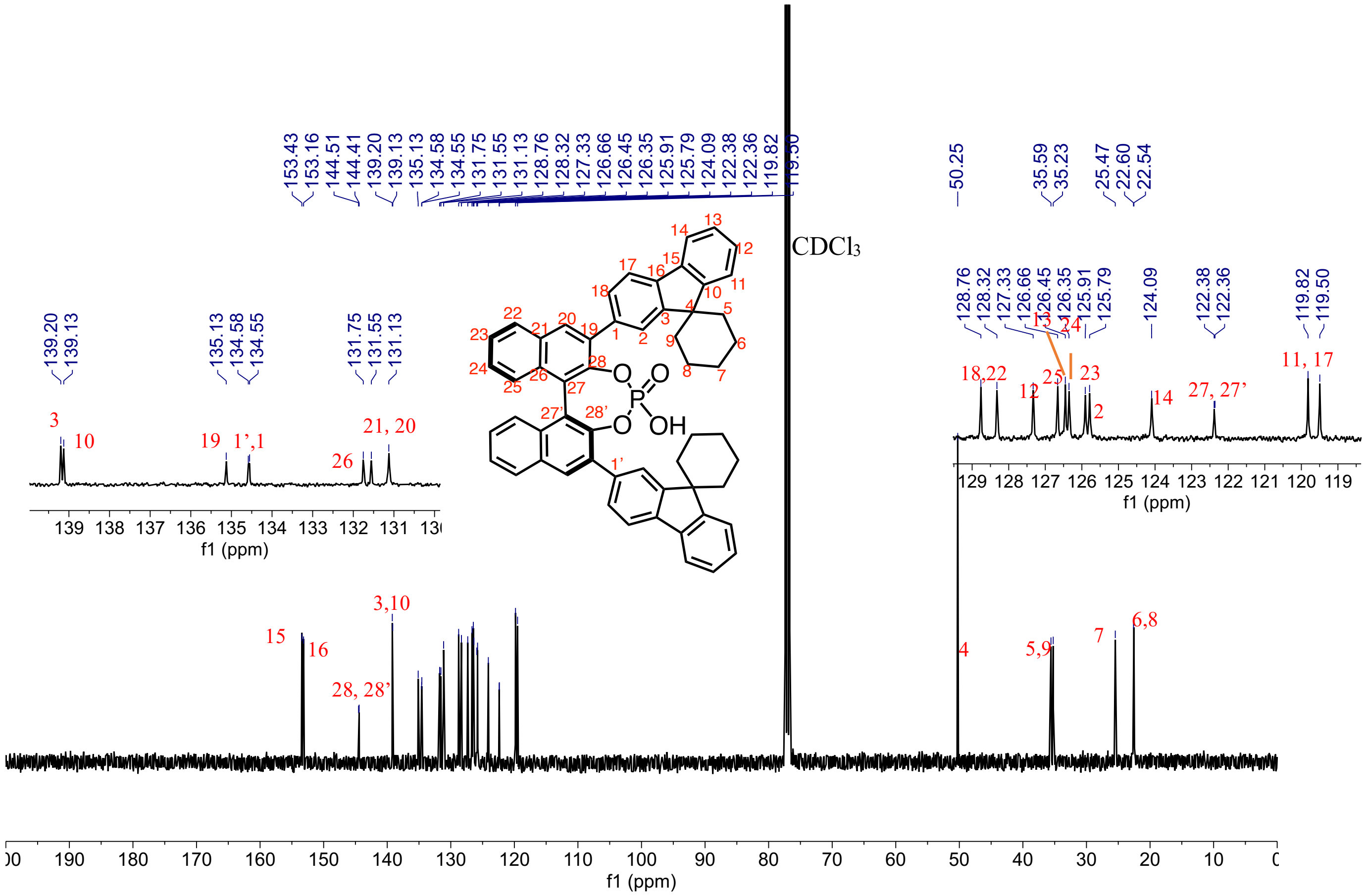

**Figure S71.** 101 MHz $^{13}C$ NMR spectra of **54** in $CDCl_3$.

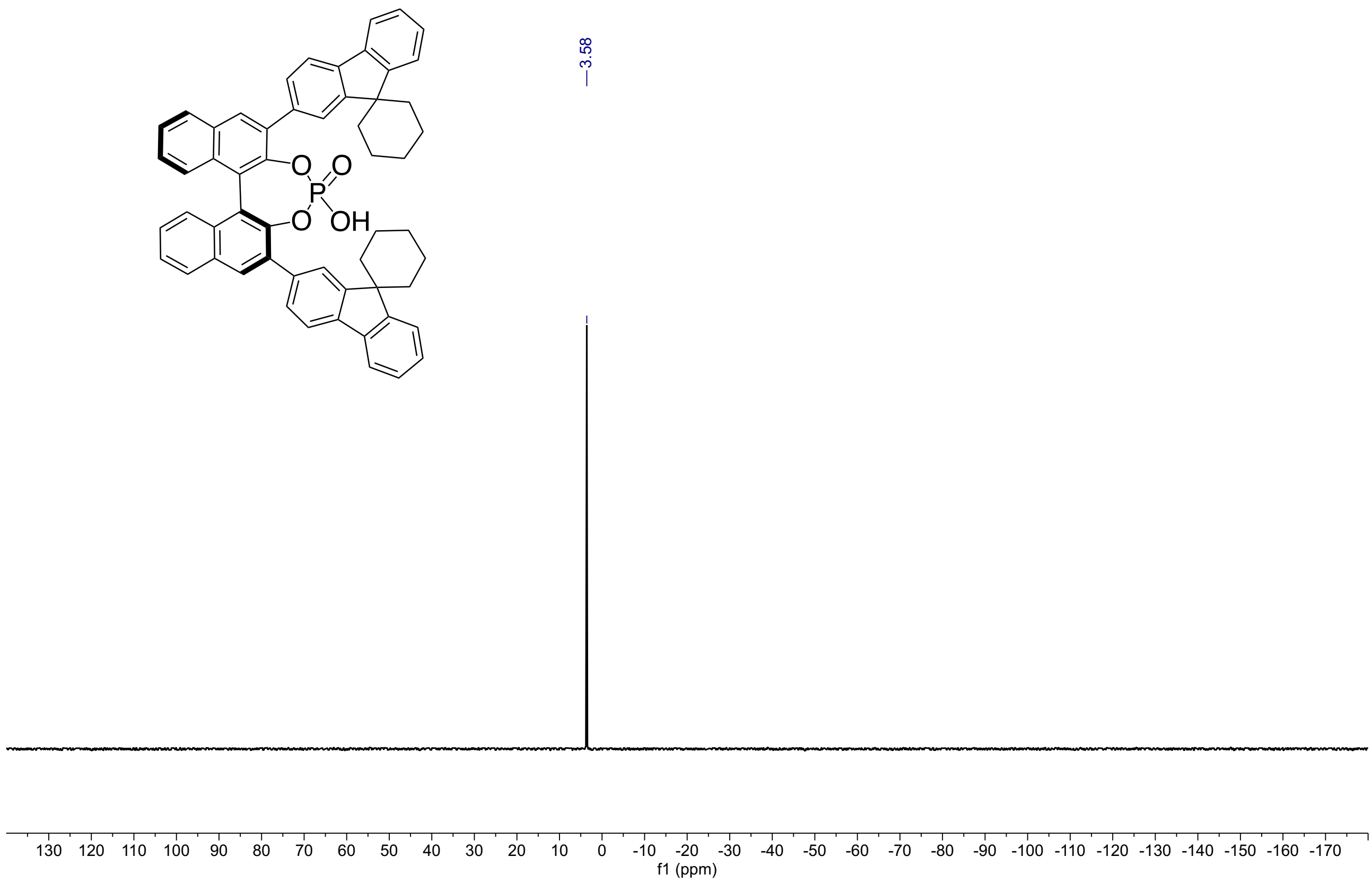


**Figure S72.** 162 MHz $^{31}$P NMR spectra of **54** in $CDCl_3$.

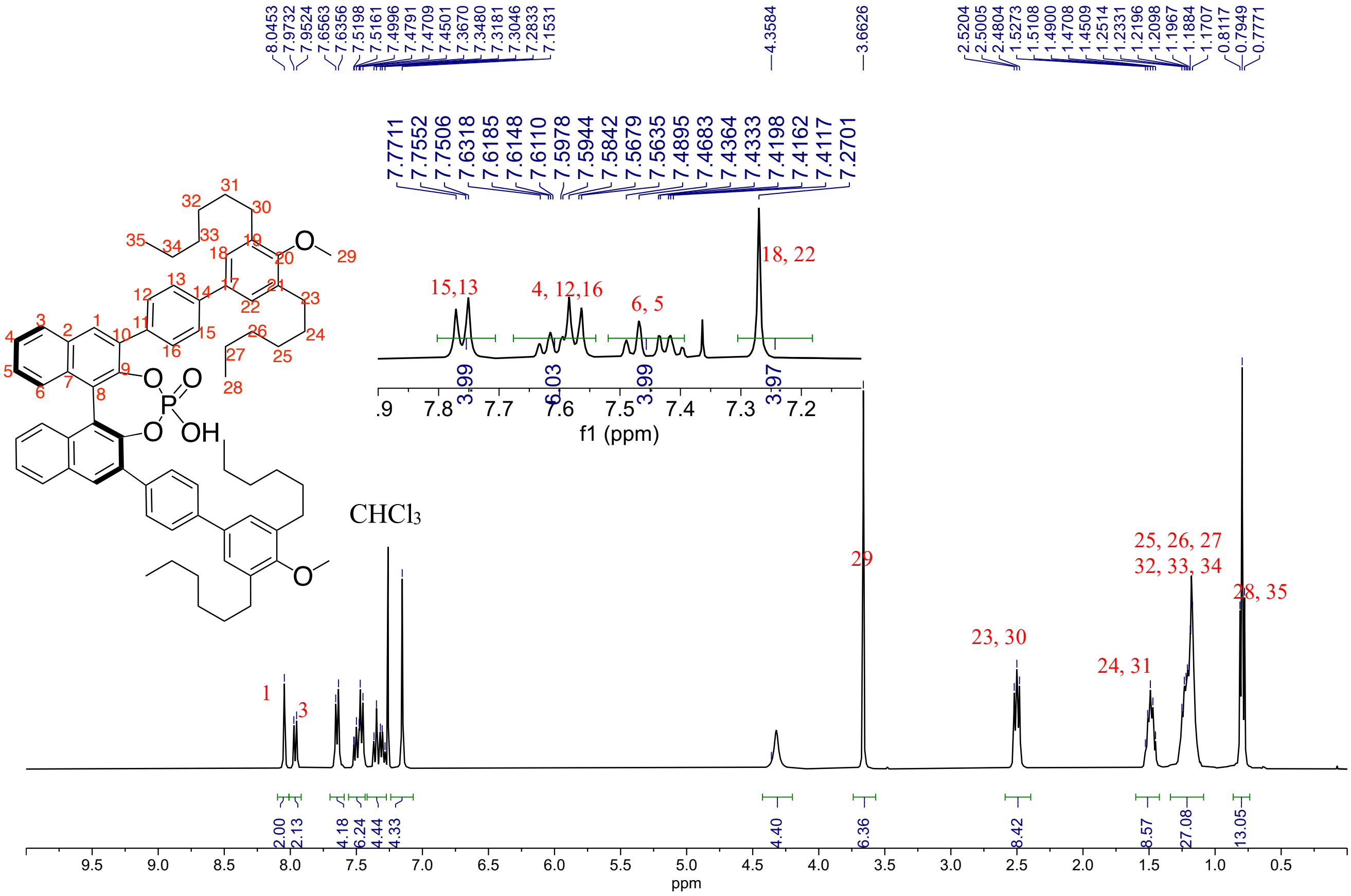

**Figure S73**. 500 MHz $^{1}$H NMR spectra of **49** in $CDCl_3$.

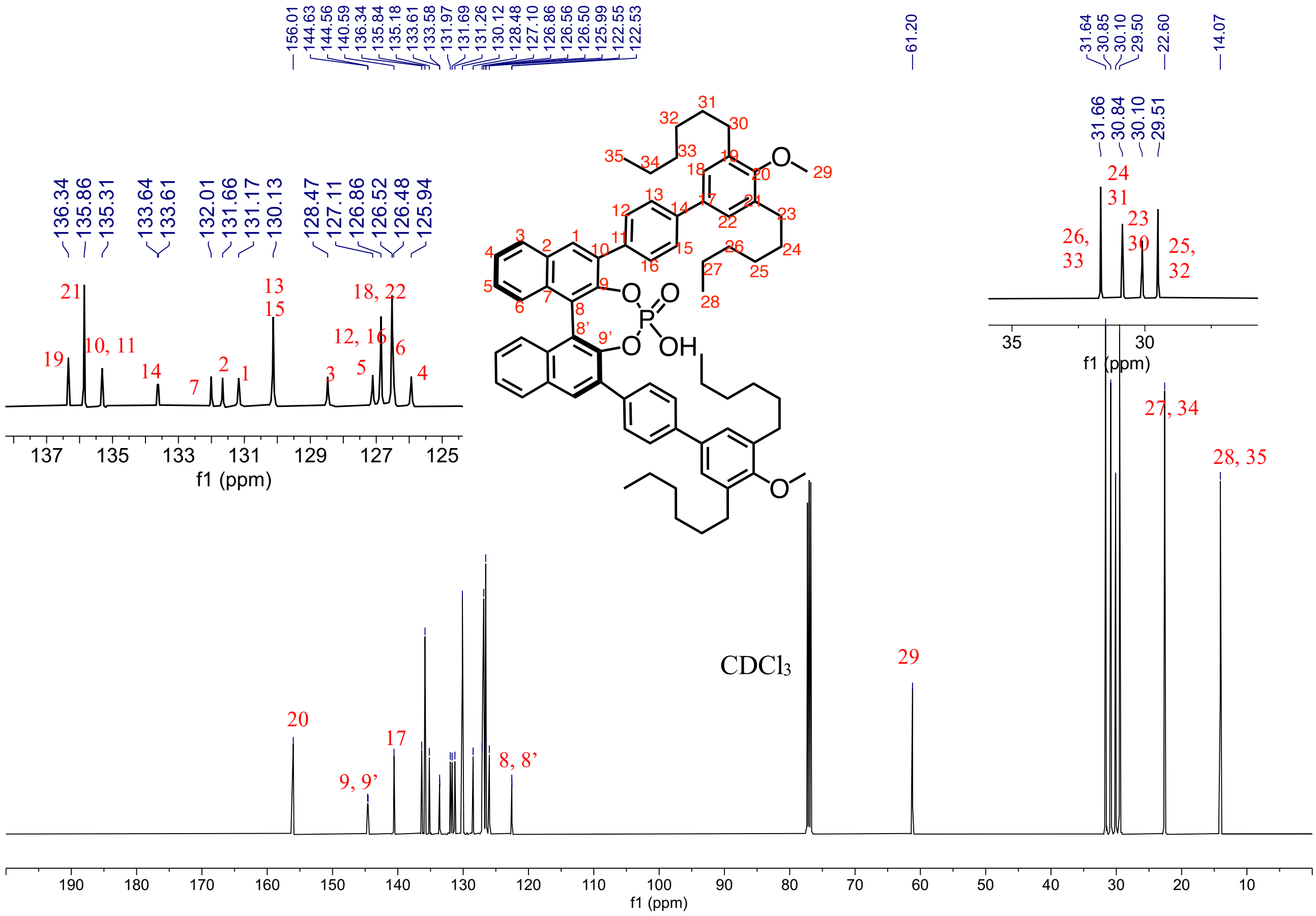

**Figure S74.** 262 MHz $^{13}C$ NMR spectra of **49** in $CDCl_3$.

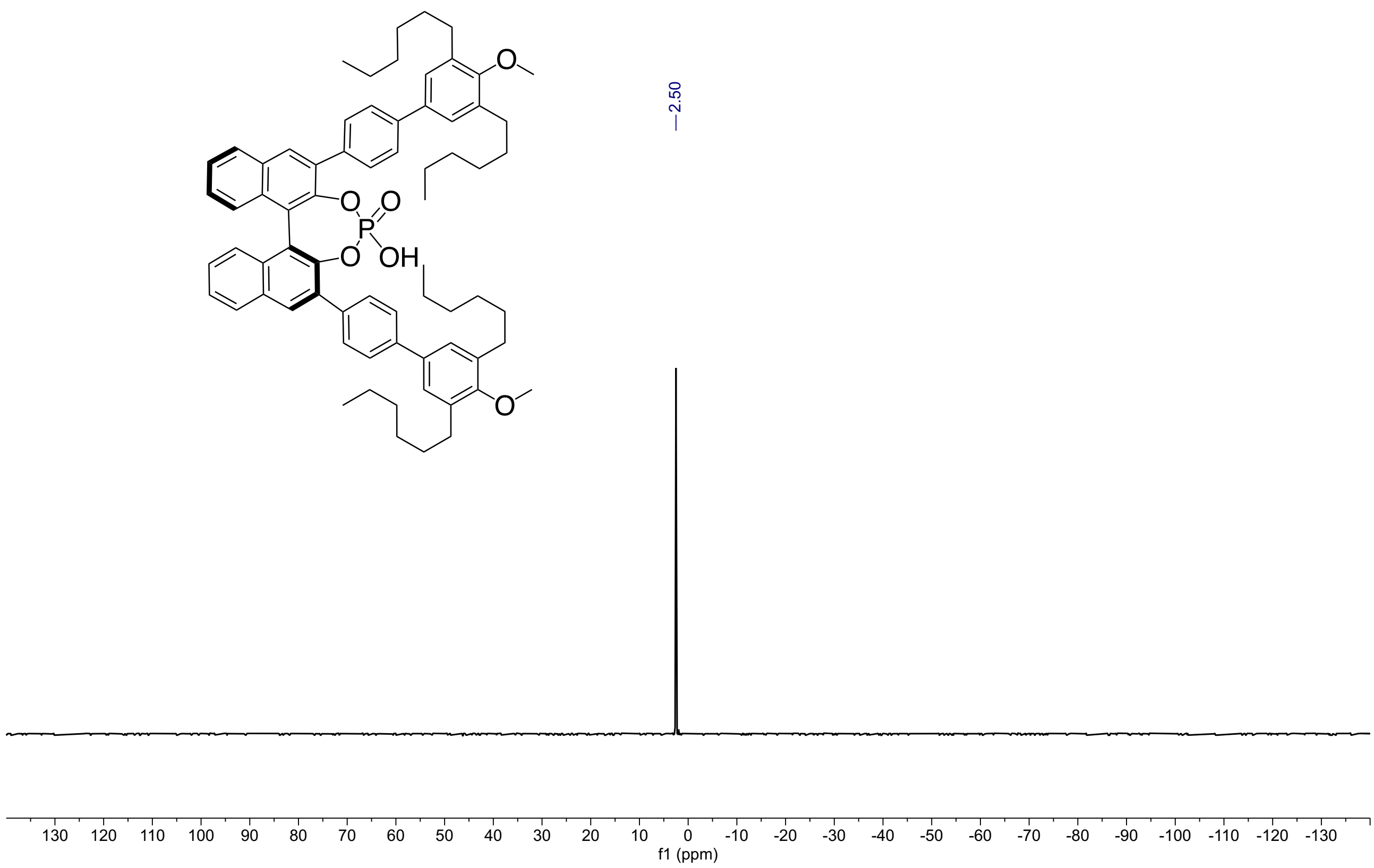


**Figure S75.** 162 MHz $^{31}P$ NMR spectra of **49** in $CDCl_3$.
